# Enantioselective Nazarov cyclization of indole enones cooperatively catalyzed by Lewis acids and chiral Brønsted acids†
†Electronic supplementary information (ESI) available: For CIF data of (*R*)-**3r**, experimental procedures, spectral data, and computational study results. CCDC 1539722. For ESI and crystallographic data in CIF or other electronic format see DOI: 10.1039/c7sc03183a



**DOI:** 10.1039/c7sc03183a

**Published:** 2017-08-29

**Authors:** Guo-Peng Wang, Meng-Qing Chen, Shou-Fei Zhu, Qi-Lin Zhou

**Affiliations:** a State Key Laboratory and Institute of Elemento-Organic Chemistry , College of Chemistry Nankai University , Tianjin 300071 , China . Email: sfzhu@nankai.edu.cn ; Email: qlzhou@nankai.edu.cn; b Collaborative Innovation Center of Chemical Science and Engineering (Tianjin) , Nankai University , Tianjin 300071 , China

## Abstract

Enantioselective control of the chirality of a tertiary α-carbon in the products of a Nazarov cyclization of enones is challenging because the reaction involves an enantioselective proton transfer process. We herein report the use of cooperative catalysis using Lewis acids and chiral Brønsted acids to control the stereochemistry of the tertiary α-carbon in the products of this reaction. Specifically, with ZnCl_2_ and a chiral spiro phosphoric acid as catalysts, we realized the first enantioselective construction of cyclopenta[*b*]indoles with chiral tertiary α-carbons *via* Nazarov cyclization of indole enone substrates with only one coordinating site. Mechanistic studies revealed that the chiral spiro phosphoric acid acts as a multifunctional catalyst: it co-catalyzes the cyclization of the dienone and enantioselectively catalyzes a proton transfer reaction of the enol intermediate. This new strategy of enantioselective control by means of cooperative catalysis may show utility for other challenging asymmetric cyclization reactions.

## Abstract 2

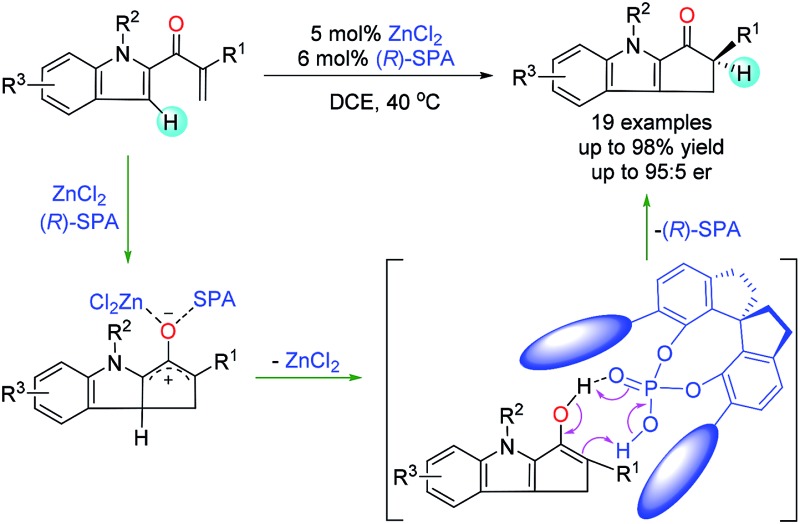
The first enantioselective Nazarov cyclization of substrates with only one coordinating site and with proton-transfer as an enantioselective-determining step was realized by means of cooperative catalysis with a Lewis acid and a chiral Brønsted acid.

## Introduction

**Fig. 1:**
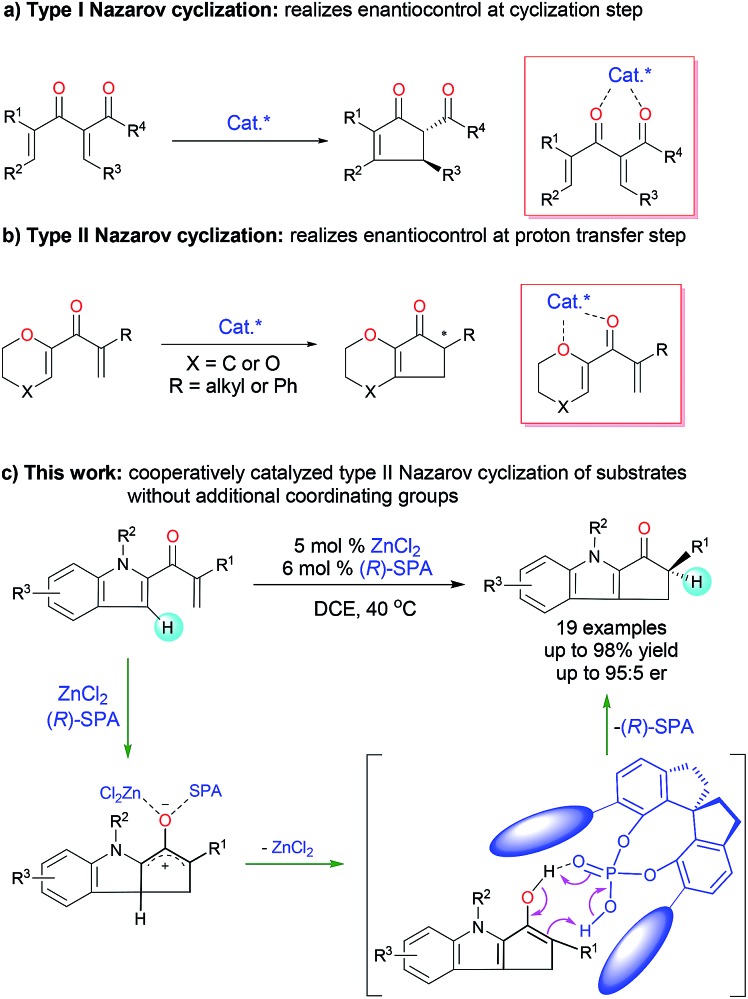
Catalytic asymmetric Nazarov cyclization.

The Nazarov cyclization, a 4π-electrocyclization of cross-conjugated dienones, is one of the most effective methods for constructing functionalized cyclopentenones and has been extensively investigated and widely utilized for the synthesis of cyclopentenoid natural products.^1^ Catalytic enantioselective Nazarov cyclization is a straightforward method for introducing chiral-center-containing cyclopentenones, which are versatile chiral synthons,^2^ and this strategy has attracted increasing attention in recent decades.^3^ The reported asymmetric Nazarov cyclization can be roughly classified into two types on the basis of the enantioselective control step: type I Nazarov cyclization affords products with a chiral center at the β-carbon by means of an enantioselective cyclization step (Fig. 1a), and type II Nazarov cyclization affords products with only one chiral center at the tertiary α-carbon by means of an enantioselective proton transfer step (Fig. 1b). Various chiral Lewis acid^4^ and Brønsted acid^5^ catalysts have been reported to afford high enantioselectivity in type I Nazarov cyclization reactions. In contrast, for type II Nazarov cyclization, the stereochemistry-determining step is a proton transfer reaction of an active enol or enolate intermediate generated during electrocyclization, and control of the enantioselectivity is much more difficult.^6^ To date, only one type II Nazarov cyclization was reported with reasonable enantioselectivity.^7^ In 2004, Liang and Trauner^7*a*^ achieved good to excellent enantioselectivity (up to 98.5 : 1.5 enantiomeric ratio [er]) in the asymmetric Nazarov cyclization of α-substituted divinyl ketones by using a Sc-Pybox catalyst (Fig. 1b). These investigators suggested that the catalyst chelates with the substrate in addition to mediating the enantioselective proton transfer reaction of the cyclized intermediate. In 2009, Rueping and Ieawsuwan^7*b*^ reported the use of a chiral Brønsted acid, typically a chiral *N*-triflylphosphoramide, to catalyze Nazarov cyclizations of the same substrates as those used by Liang and Trauner with moderate to good enantioselectivities. However, both of these seminal reactions require a dienone substrate with an additional coordinating group (an ether group) for high enantioselectivity. To our knowledge, enantioselective type II Nazarov cyclization of substrates without an additional coordinating group has not been realized, and strategies to address this challenge are highly desirable. In our recent studies of transition-metal-catalyzed enantioselective carbene insertion into heteroatom–hydrogen bonds,^8^ we developed a method for cooperative catalysis using achiral dirhodium complexes and chiral spiro phosphoric acids (SPAs) to accomplish asymmetric heteroatom–hydrogen bond insertion reactions with excellent enantioselectivity.^9^ Mechanistic studies revealed that the SPAs catalyze a proton transfer reaction of an ylide, enol, or enolate intermediate by acting as chiral proton transfer shuttles.^9b,10^ Inspired by these results, we investigated the type II Nazarov cyclization of substrates with only one coordinating group by means of cooperative catalysis with a Lewis acid and a chiral Brønsted acid to control the enantioselectivity of the proton transfer step (Fig. 1c).^11^ With the combination of ZnCl_2_ and a SPA, highly enantioselective Nazarov cyclization of indole enones was accomplished.

## Results and discussion

To identify suitable catalysts, we carried out the Nazarov cyclization of 2-methyl-1-(1-methyl-1*H*-indol-2-yl)prop-2-en-1-one (**2a**, Table 1). We began by evaluating Sc-Pybox and a chiral *N*-triflylphosphoramide, which has been previously used in the literature^7^ to catalyze the type II Nazarov cyclization of substrates with two coordinating groups. However, neither of these catalysts triggered the cyclization reaction of **2a**. To our delight though, the combination of FeCl_3_ (5 mol%) and chiral SPA (*R*)-**1a** (6 mol%) promoted the reaction at 25 °C in 1,2-dichloroethane (DCE), affording the desired cyclization product (**3a**) in 95% yield with 63 : 37 er (entry 1). Various other chiral SPAs were then evaluated (entries 2–7), and (*R*)-**1e**, which has 6,6′-di-(2,4,6-tri-isopropyl)phenyl substituents, gave the highest enantioselectivity (85 : 15 er, entry 5). Chiral phosphoric acid (*R*)-**4**, which has a binaphthyl scaffold, showed almost no enantioselectivity (entry 8). Different Lewis acids were then studied with (*R*)-**1e** as a co-catalyst. ZnCl_2_, Zn(OTf)_2_, AgClO_4_, Sc(OTf)_3_, and In(OTf)_3_ afforded good enantioselectivities (up to 92 : 8 er, entries 9–13), although Zn(OTf)_2_ and AgClO_4_ showed relatively low yields. Using ZnCl_2_ and (*R*)-**1e** as co-catalysts, we then optimized the reaction conditions. When the reaction was performed at 40 °C, the yield increased to 98%, and the high enantioselectivity was retained (entry 14). Increasing the temperature further (to 60 °C) increased the reaction rate (the reaction was complete after only 20 h) but slightly lowered the enantioselectivity (87 : 13 er, entry 15). The kinetic studies indicate that the reaction achieves 90% conversion within 6 hours, but becomes sluggish thereafter. The use of CHCl_3_ did not markedly alter the yield or enantioselectivity, whereas the nonpolar solvent cyclohexane lowered both the yield and enantioselectivity (entries 16 and 17).

**Table 1:** Enantioselective Nazarov cyclization of **2a** cooperatively catalyzed by Lewis acids and chiral phosphoric acids: optimization of reaction conditions^*a*^

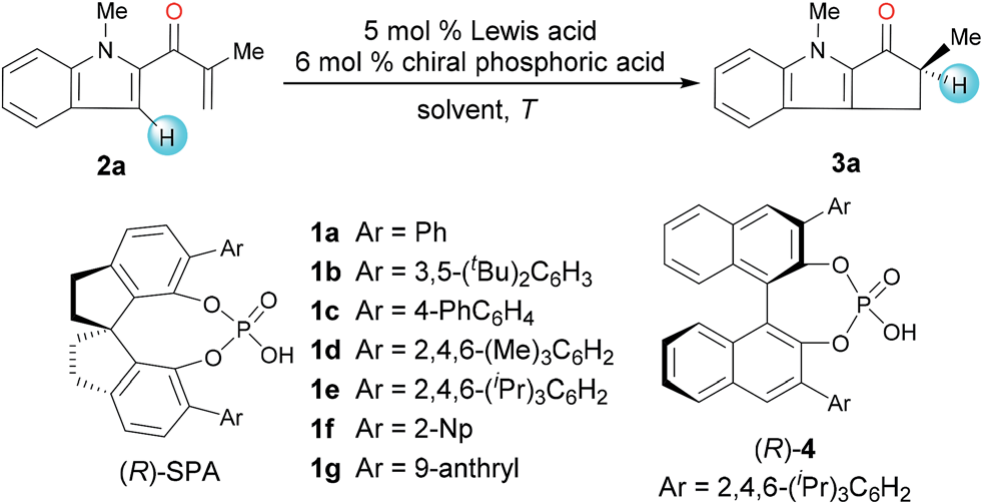						
Entry	Lewis acid	Phosphoric acid	*T* (°C)	Time (h)	Yield^*b*^ (%)	er^*c*^
1	FeCl_3_	(*R*)-**1a**	25	36	95	63 : 37
2	FeCl_3_	(*R*)-**1b**	25	30	83	46 : 54
3	FeCl_3_	(*R*)-**1c**	25	35	95	44 : 56
4	FeCl_3_	(*R*)-**1d**	25	48	95	63 : 37
5	FeCl_3_	(*R*)-**1e**	25	48	98	85 : 15
6	FeCl_3_	(*R*)-**1f**	25	48	93	52 : 48
7	FeCl_3_	(*R*)-**1g**	25	36	95	57 : 43
8	FeCl_3_	(*R*)-**4**	25	36	98	48 : 52
9	ZnCl_2_	(*R*)-**1e**	25	48	78	91 : 9
10	Zn(OTf)_2_	(*R*)-**1e**	25	48	48	90 : 10
11	AgClO_4_	(*R*)-**1e**	25	48	50	92 : 8
12	Sc(OTf)_3_	(*R*)-**1e**	25	48	73	91 : 9
13	In(OTf)_3_	(*R*)-**1e**	25	24	93	86 : 14
14	ZnCl_2_	(*R*)-**1e**	40	48	98	91 : 9
15	ZnCl_2_	(*R*)-**1e**	60	20	100	87 : 13
16^*d*^	ZnCl_2_	(*R*)-**1e**	40	48	95	90 : 10
17^*e*^	ZnCl_2_	(*R*)-**1e**	40	48	25	62 : 38
18^*f*^	ZnCl_2_	None	40	48	65	NA
19	None	(*R*)-**1e**	40–60	48	0	NA
20	ZnCl_2_	(*R*)-**1e**-Na	40	48	0	NA

^*a*^Reaction conditions: Lewis acid/phosphoric acid/**2** = 0.01 : 0.012 : 0.2 (mmol) in 3 mL DCE, 40 °C, 48 h.

^*b*^Isolated yield.

^*c*^Determined by HPLC using a Chiralpak AD-3 or Chiralcel OD-3 column.

^*d*^3 mL CHCl_3_ used as solvent.

^*e*^3 mL cyclohexane used as solvent.

^*f*^75% conversion of **2a**. NA = not analysed.

We performed a set of control experiments to elucidate the roles of ZnCl_2_ and the SPA. In the absence of SPA (*R*)-**1e**, ZnCl_2_ independently catalyzed the cyclization (entry 18), but the reaction rate was clearly slower than when both catalysts were used (entry 14). This result indicates that the SPA accelerated the ZnCl_2_-catalyzed cyclization reaction of **2a**. However, no reaction occurred in the presence of the SPA alone at 40 °C or even at 60 °C (entry 19). Moreover, when we used the sodium salt of the SPA, (*R*)-**1e**-Na, the Nazarov cyclization did not occur (entry 20). The anionic SPA’s lack of catalytic activity may have been due to the formation of zinc(ii) phosphate (*R*)-**1e**-Zn, which was inert under the reaction conditions.

Under the optimal reaction conditions, we evaluated indole enones with various groups (R^2^) on the N atom of the indole ring (Table 2). Substrates with either an N-alkyl or N-aryl group gave good to excellent yields (76–98%), but substrates with an N-aryl group showed the best enantioselectivities (91 : 9–92 : 8 er). The presence of an N-aryl group with a strongly electron-withdrawing CF_3_ group lowered the yield (entry 7).

**Table 2:** Enantioselective Nazarov cyclization of **2** cooperatively catalyzed by ZnCl_2_ and (*R*)-**1e**: influence of N-protecting groups^*a*^

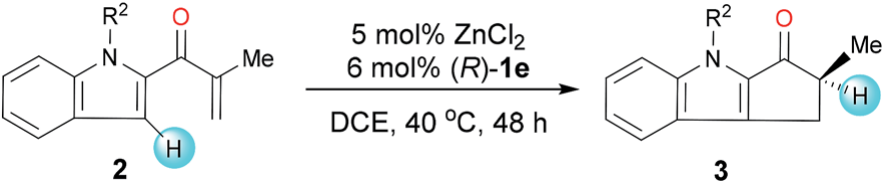				
Entry	R^2^	Product	Yield^*b*^ (%)	er^*c*^
1	Me (**2a**)	**3a**	98	91 : 9
2	^i^Pr (**2b**)	**3b**	98	90 : 10
3	Allyl (**2c**)	**3c**	93	89 : 11
4	Bn (**2d**)	**3d**	87	88 : 12
5	Ph (**2e**)	**3e**	90	92 : 8
6	An (**2f**)	**3f**	95	92 : 8
7	4-CF_3_C_6_H_4_ (**2g**)	**3g**	76	91 : 9
8	2-Np (**2h**)	**3h**	92	92 : 8

^*a*^Reaction conditions: ZnCl_2_/(*R*)-**1e**/**2** = 0.01 : 0.012 : 0.2 (mmol) in 3 mL DCE, 40 °C, 48 h.

^*b*^Isolated yield.

^*c*^Determined by HPLC using a Chiralpak AD-3 or Chiralcel OD-3 column.

Next, we determined the effect of the R^1^ group on the α-carbon of enone substrates **2** (Table 3). When R^1^ was alkyl, excellent yields and good enantioselectivities were obtained, with a bulky isopropyl group giving the highest enantioselectivity (**3j**, 95 : 5 er). When R^1^ was aryl, however, Zn(OTf)_2_ had to be used instead of ZnCl_2_ to obtain satisfactory enantioselectivities (**3k–3o**, 85 : 15–92 : 8 er). Substrates with a substituent at the 4- or 5-position of the indole ring (**3p–3s**) underwent the reaction smoothly and gave good yields (71–94%) and high enantioselectivities (92 : 8–95 : 5 er). The structure and absolute configuration of (*R*)-**3r** were determined using X-ray diffraction of a single crystal.^12^

**Table 3:** Enantioselective Nazarov cyclization of **2** cooperatively catalyzed by ZnCl_2_ or Zn(OTf)_2_ and (*R*)-**1e**: substrate scope^*a*^

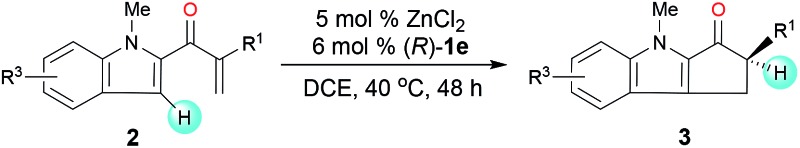
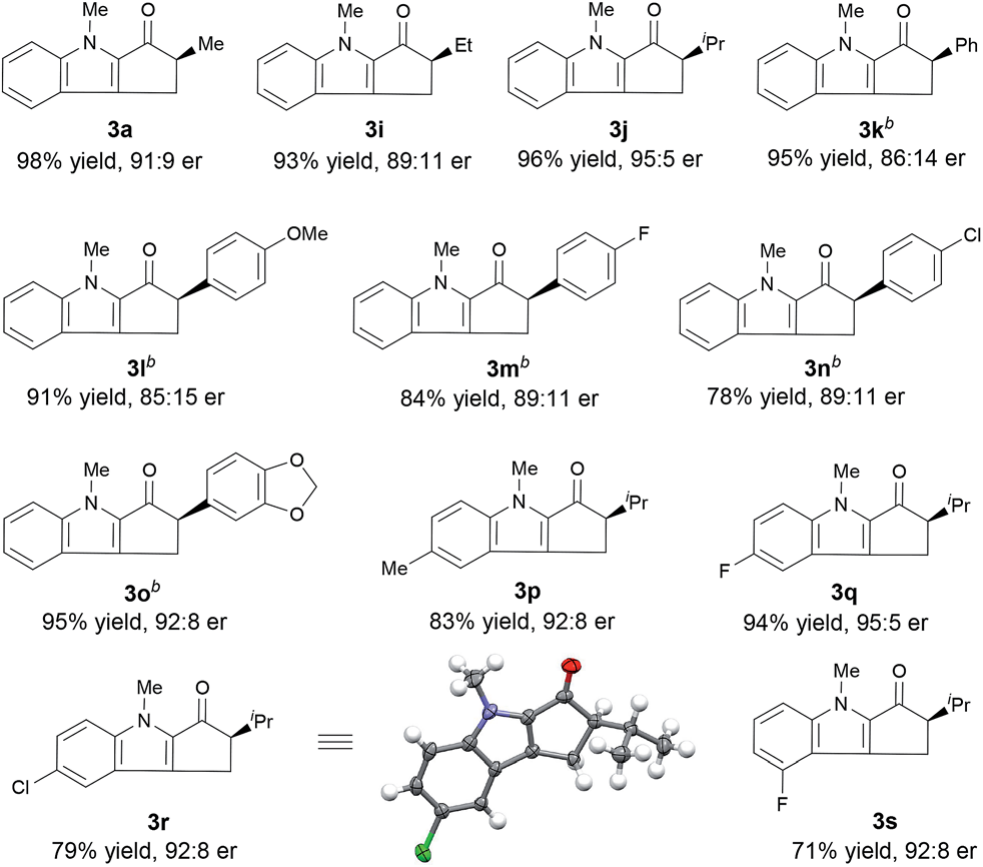

^*a*^Reaction conditions: ZnCl_2_/(*R*)-**1e**/**2** = 0.01 : 0.012 : 0.2 (mmol) in 3 mL DCE, 40 °C, 48 h. Isolated yields are given. The er values were determined by HPLC using a Chiralpak AD-3 or Chiralcel OD-3 column.

^*b*^Zn(OTf)_2_ used instead of ZnCl_2_.

**Scheme 1:**
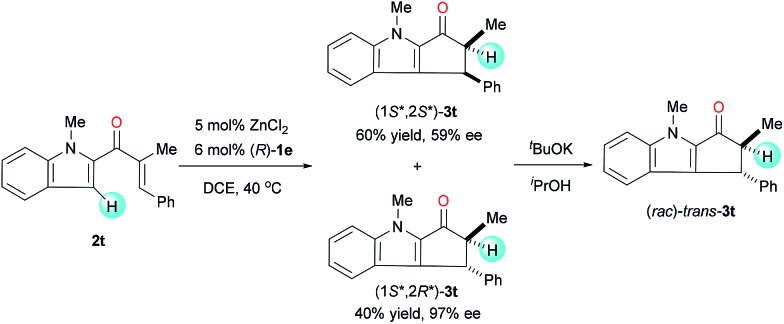
Nazarov cyclization of **2t** with a β-phenyl substitution.

Under the standard reaction conditions, the substrate **2t**, having a β-phenyl substitution, smoothly cyclized to give a mixture of diastereoisomers (1S*,2S*)-**3t** and (1S*,2R*)-**3t** with 1.5 : 1 dr (Scheme 1). The enantioselectivity of (1S*,2R*)-**3t** is very high (97% ee), though the enantioselectivity of (1S*,2S*)-**3t** is moderate (59% ee). When the mixture of cyclization products was treated with ^*t*^BuOK in ^i^PrOH, it transformed to a racemic single diastereoisomer through the epimerization of α-carbons. This experiment clearly implies that the enantio-control on the β-carbon is not achieved during the cyclization step and the enantioselectivity of the reaction is achieved during the protonation step.

**Scheme 2:**
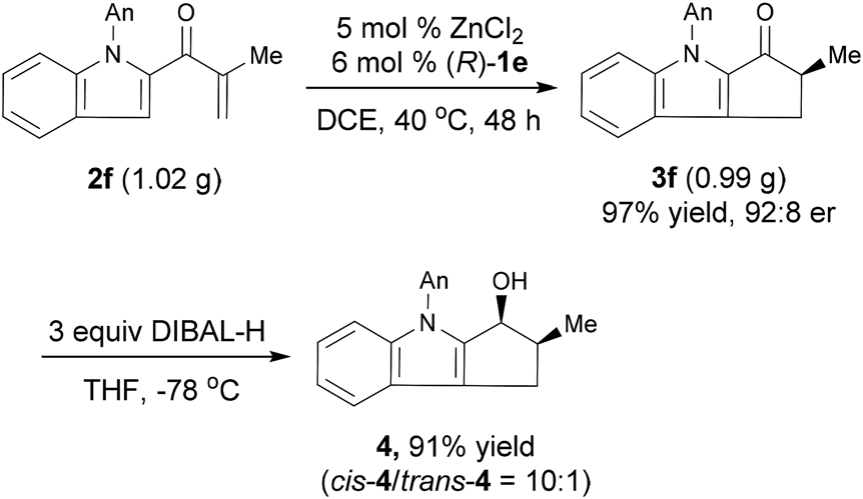
Gram-scale experiment and a transformation of product.

The Nazarov cyclization of **2** could readily be carried out on a gram scale. For example, the reaction of 1.02 g **2f** under the standard conditions smoothly produced chiral cyclopentanone[*b*]indole **3f** in 97% yield with 92 : 8 er (Scheme 2). Reduction of **3f** with diisobutylaluminum hydride (DIBAL-H) afforded the corresponding alcohol *cis*-**4** in 91% yield with a high diastereoselectivity (*cis*/*trans* = 10 : 1) and retained er. The relative configuration of *cis*-**4** was confirmed by a nuclear Overhauser enhancement spectroscopy experiment (see ESI for details†). Note that cyclopenta[*b*]indoles are common building blocks for indole alkaloids, such as (+)-dasyrachine,^13^ penitrems A–F,^14^ 15α-hydroxy-14,15-dihydrovindolinine,^15^ (+)-paxilline,^16^ and lolitrem B.^17^ Indeed, a similar non-enantioselective Nazarov cyclization was used in the total synthesis of yuehchukene analogues.^18^

**Fig. 2:**
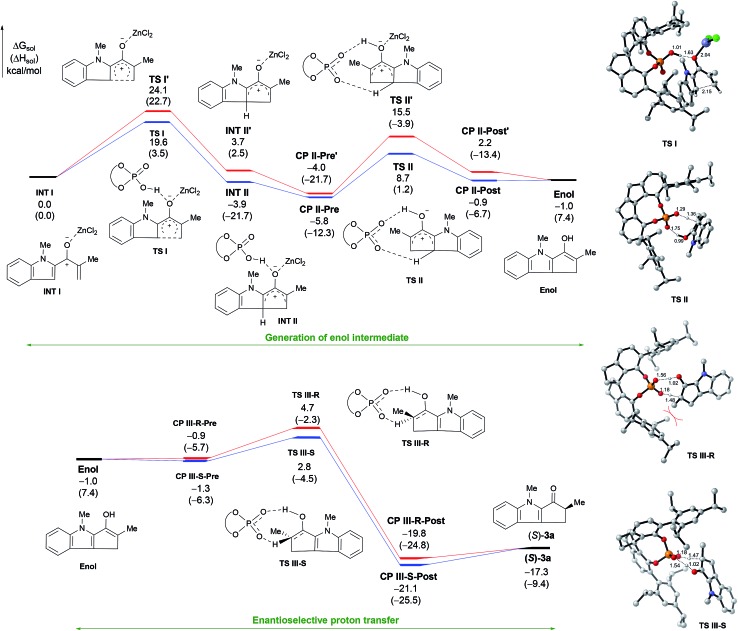
The computed energy surfaces for the Nazarov cyclization of **2a** cooperatively catalyzed by ZnCl_2_ and (*R*)-**1e**.

To obtain more insight into the mechanism, we carried out density functional theory calculations at the B3LYP/def2TZVP-SMD(DCE)//B3LYP/def2SVP level of theory using Gaussian 09 (see ESI for details†). The cyclization of **2a** by cooperative catalysis using ZnCl_2_ and (*R*)-**1e** was used as the model reaction (Fig. 2). The calculations indicate that the cyclization starts with the coordination of ZnCl_2_ to the carbonyl group of the substrate (**INT 1**). The SPA facilitates 4π-electrocyclization *via* **TS I** to afford intermediate **INT II**. In the absence of (*R*)-**1e**, that is, when ZnCl_2_ is the sole catalyst, the reaction proceeds *via* an alternative transition state (**TS I′**) with a higher (4.5 kcal mol^–1^) energy barrier. Like the control experiments, the calculation results support our contention that ZnCl_2_ and (*R*)-**1e** cooperatively catalyze the cyclization. The SPA then promotes a proton transfer reaction of **INT II** *via* **TS II** to generate the enol intermediate. The formation of **TS II** has an activation barrier of 12.6 kcal mol^–1^ in solution, which is not a problem when the reaction is carried out at 25 °C. The calculations also suggest that the Lewis acid ZnCl_2_ prefers to dissociate from **INT II** during the proton transfer reaction, because the energy barrier for the formation of **TS II′**, which contains ZnCl_2_, is 6.8 kcal mol^–1^ higher than that for **TS II**. The enol intermediate can form a complex (**CP III-R** or **CP III-S**) with (*R*)-**1e** and then undergo [1,3]-proton transfer to give the cyclization product **3a**.^19^ Our calculations indicate that (*R*)-**1e** catalyzes the [1,3]-proton transfer *via* an eight-membered transition state (**TS III-R** or **TS III-S**).^9*b*^ **TS III-S** is lower in energy than **TS III-R** by 1.9 kcal mol^–1^. This result suggests that a product mixture enriched with enantiomer (*S*)-**3a** (calcd er = 96 : 4) should be generated from **TS III-S**, which is consistent with the experimental result. In the energetically disfavored transition state (**TS III-R**), the Me group at the α-position of the substrate experiences more steric repulsion from the 2,4,6-triisopropyphenyl group of (*R*)-**1e** than the same Me group in the favored transition state (**TS III-S**). The steric repulsion may be induced by rotation of the indole plane to some extent in the chiral cavity. Other proton transfer processes of the enol intermediate with and without promoters were also calculated, but the energy barriers of all of these other processes were higher than the barrier for when the SPA was used as a catalyst (see Fig. S4†). Our calculations reveal that the SPA first acts as a Brønsted acid catalyst to promote the cyclization and later acts as a chiral proton transfer shuttle to promote an enantioselective proton transfer reaction of the enol intermediate, thus exerting enantioselectivity of the Nazarov cyclization.

## Conclusions

In conclusion, we have accomplished enantioselective Nazarov cyclizations of indole enones with only one coordinating site in high yield and good enantioselectivity by using ZnCl_2_ as a Lewis acid catalyst and a chiral SPA as a co-catalyst. Proton transfer is the stereochemistry-determining step of the process. The Lewis acid and the SPA promote the cyclization cooperatively. The SPA also promotes a proton transfer reaction of the enol intermediate and exerts enantioselective control over the tertiary α-carbon chiral center generated by the Nazarov cyclization. This new strategy of using cooperative catalysis for enantioselective control has great potential for application to other challenging asymmetric cyclization reactions.

## Conflicts of interest

There are no conflicts of interest to declare.
