# Supplementary material for: Enantioselective Nazarov cyclization of indole enones cooperatively catalyzed by Lewis acids and chiral Brønsted acids
Source: Chem Sci. 2017 Aug 29;8(10):7197–202. doi: 10.1039/c7sc03183a (PMC5633839; doi:10.1039/c7sc03183a)
Supplement: Supplementary file 1 [file SC-008-C7SC03183A-s001.pdf]

Electronic Supplementary Material (ESI) for Chemical Science.
This journal is © The Royal Society of Chemistry 2017

Supplementary Information for:

Enantioselective Nazarov cyclization of indole enones cooperatively catalyzed by Lewis acids and chiral Brønsted acids

Guo-Peng Wang,[a] Meng-Qing Chen,[a] Shou-Fei Zhu,*[,a] Qi-Lin Zhou,*[,a,b]

[a] *State Key Laboratory and Institute of Elemento-Organic Chemistry, College of Chemistry, Nankai University, Tianjin 300071, China*

[b] *Collaborative Innovation Center of Chemical Science and Engineering (Tianjin), Nankai University, Tianjin 300071, China*

Contents:

1. General Information......2

2. Synthesis and Analytical Data of Substituted Indole Enones......2

3. Typical Procedure for Nazarov Cyclization of Indole Enones......19

4. Analytical Data of Cyclization Products......19

5. X-Ray Diffraction Analysis of (*R*)-3r......29

6. Transformations of Cyclization Product......31

7. NMR Spectra of Intermediates and Indole Enones......33

8. NMR Spectra of Cyclization Products and Transformation Product......71

9. HPLC Charts of Cyclization Products and Transformation Product......92

10. References......112

1. General Information

Unless otherwise noted, all solvents used in the reactions were distilled from appropriate drying agents prior to use.[1] $ZnCl_2$ (99.99%) was purchased from Alfa Aesar Chemical Company and sublimated under high vacuum before use. $Zn(OTf)_2$ (98%) was purchased from Sigma-Aldrich Chemical Company and used without further purification. All reactions and manipulations which are sensitive to moisture or air were performed in an argon-filled glovebox (MBRAUN LABstar) or using standard Schlenk techniques. ^{1}H NMR and ^{13}C NMR spectra were recorded with a Brucker AV 400 spectrometer at 400 MHz (^{1}H NMR) and 101 MHz (^{13}C NMR) in $CDCl_3$. Chemical shifts were reported in ppm down field from internal Me_4Si. HRMS were recorded on an Agilent 6520 Q-TOF LC/MS spectrometer with ESI or MALDI-TOF resource. HPLC analysis was performed on an Agilent 1260 Infinity LC chromatography. Melting points were measured on a RY–I or SGW X-4 apparatus and uncorrected. Optical rotations were determined by a Rudolph Autopol VI polarimeter. Infrared spectra were obtained on a Nicolet MAGNA-IR 560 FT-IR spectrometer and peak values are reported in reciprocal centimeters (cm^{-1}).

2. Synthesis and Analytical Data of Substituted Indole Enones

2.1 Synthesis of indole enones

Indole enones were synthesized following the procedures below, while N-substituted indole-2-carbaldehyde, substituted vinyl bromide, α,β-unsaturated aldehyde and Weinreb amide were purchased or synthesized according to literatures. Substrates **2a**–**2i**, **2k**–**2o** were prepared according to route A; substrates **2j** and **2p** were prepared according to route B; substrates **2q**–**2s** were prepared according to route C.

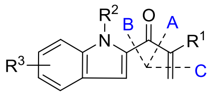

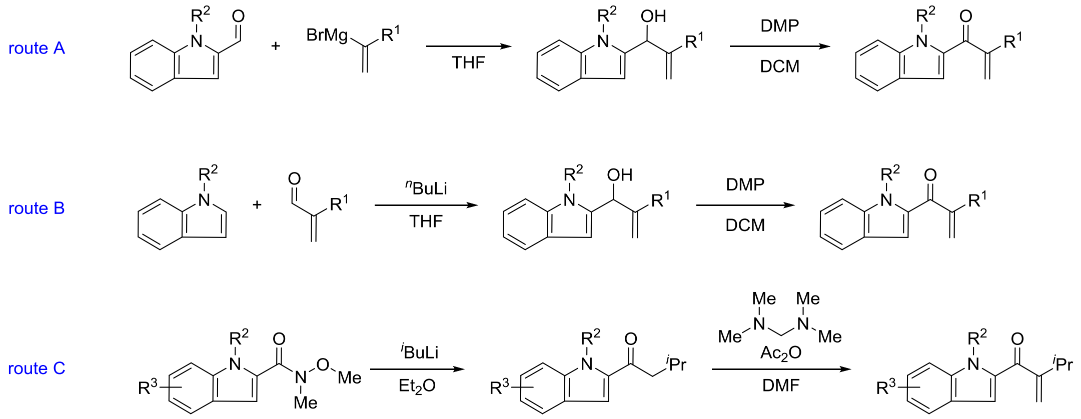

General Method of Route A[2,3,4]

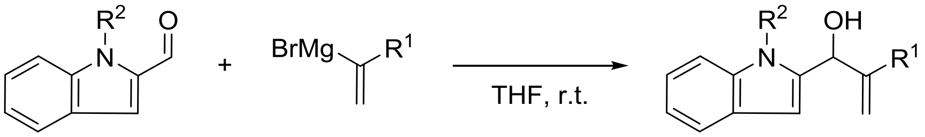

In an oven dried 100 mL four-necked flask, Mg turnings (1.09 g, 45 mmol) and a small amount of the solution of substituted vinyl bromide (30 mmol) in dry THF (40 mL) was added successively. After initiation automatically, the remaining solution was added alternately to keep the reaction refluxing slightly. After complete addition, the mixture was heated at 45~50 ^{o}C for 30 min. The resulting solution was transferred dropwise to a solution of N-substituted indole-2-carbaldehyde (20 mmol) in THF (20 mL). The reaction mixture was stirred for 30 min at room temperature. Saturated aq. NH_4Cl (10 mL) was added to quench the reaction. The resulting mixture was then extracted with ethyl acetate and the organic layer was combined and dried over anhydrous $MgSO_4$. The solvent was evaporated under reduced pressure and the residue was purified by column chromatography on silica gel to afford the desired indole enol.

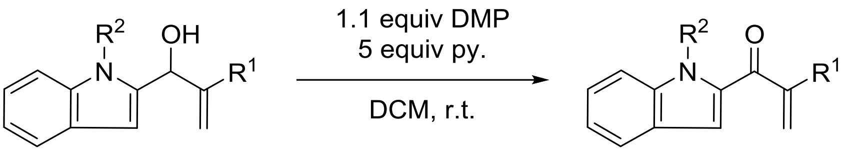

To a solution of the Dess-Martin periodinane (DMP, 9.26 g, 21.8 mmol) in DCM (40 mL), pyridine (8.0 mL, 99.3 mmol) was added. The resulting mixture was stirred for 5 min, then a solution of indole enol (19.9 mmol) in DCM (25 mL) was added dropwise. Stirring was continued for another 30 min and the reaction mixture was diluted and washed with the mixture of saturated $NaHCO_3$ and saturated $Na_2S_2O_3$ (1:1 v/v) for 3 times, then dried over anhydrous Na_2SO_4. The solvent was evaporated under reduced pressure and the residue was purified by column chromatography on silica gel to afford the desired indole enone.

General Method of Route B[5,6]

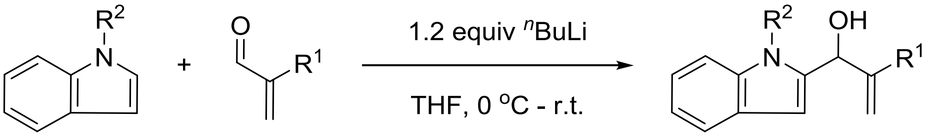

N-substituted indole (15 mmol) was dissolved in dry THF (25 ml) and then ^{n}BuLi (7.5 mL, 2.4 M, 18 mmol) was added at 0 ^oC. The mixture was stirred for 3 h at room temperature. Then the 2-methylene-aldehyde (16.5 mmol) was added slowly, and the resulting mixture was stirred for 12 h at room temperature. The mixture was carefully quenched with saturated aq. NH_4Cl at 0 °C. The organic layer was separated and aqueous layer was extracted with Et_2O twice. The combined organic layer was washed with brine and dried over anhydrous Na_2SO_4. The solvent was evaporated under reduced pressure and the residue was purified by column chromatography on silica gel to afford the desired indole enol, which subsequently been oxidized to corresponding ketone using the method described in Route A.

General Method of Route C[7,8]

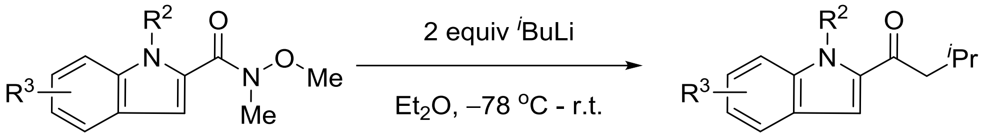

To a solution of substituted Weinreb amide (15.2 mmol) in dry Et_2O (50 mL) was added ^{i}BuLi (38 mL, 1.0 M, 38 mmol) at –78 °C over the course of about 30 min. Upon consumption of the starting material, monitored by TLC, the reaction was allowed to warm to about –50 °C, and saturated aq. NH_4Cl (15 mL) was added slowly to quench the reaction. After the reaction mixture was warmed to room temperature, the organic layer was separated and aqueous layer was extracted with Et_2O twice. The combined organic layer was dried over anhydrous Na_2SO_4. The solvent was evaporated under reduced pressure and the residue was purified by column chromatography on silica gel to afford the desired ketone.

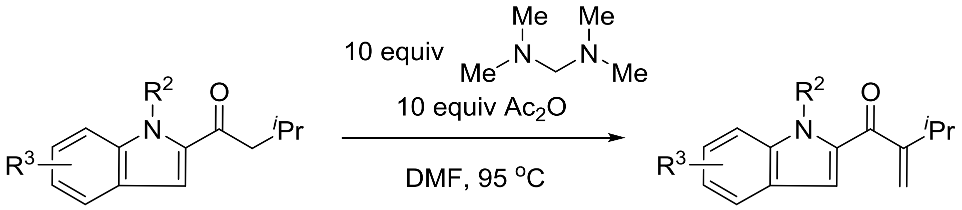

Bis(dimethylamino)methane (17 mL, 125 mmol) and acetic anhydride (12 mL, 125 mmol) was added successively to a solution of ketone (12.5 mmol) dissolved in anhydrous DMF (12 mL). The mixture was stirred at 95 °C until the consumption of starting material, monitored by TLC or ^{1}H NMR. After cooled to room temperature, the brown mixture was distilled under reduced pressure and the residue was purified by column chromatography on silica gel to afford the desired indole enone.

2.2 Analytical Data of Intermediates

2-methyl-1-(1-methyl-1H-indol-2-yl)prop-2-en-1-ol

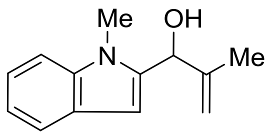

Light yellow solid, mp: 64–65 °C. TLC R_f = 0.25 (petroleum ether/ethyl acetate, PE/EA = 10:1, v/v), 93% yield. ^{1}H NMR (400 MHz, $CDCl_3$): δ 7.57 (d, *J* = 7.9 Hz, 1H), 7.30 (dd, *J* = 8.2, 0.5 Hz, 1H), 7.21 (ddd, *J* = 8.2, 5.6, 2.1 Hz, 1H), 7.09 (td, *J* = 7.5, 1.0 Hz, 1H), 6.42 (s, 1H), 5.30 (s, 1H), 5.22 (d, *J* = 0.7 Hz, 1H), 5.10 (dd, *J* = 2.7, 1.4 Hz, 1H), 3.75 (s, 3H), 2.02 (s, 1H), 1.75 (s, 3H); ^{13}C NMR (101 MHz, $CDCl_3$) δ 144.8, 139.7, 138.2, 127.1, 121.8, 120.7, 119.5, 111.9, 109.1, 100.9, 71.2, 30.1, 19.5. ESI-HRMS calcd for $[C_{13}H_{16}NO, M + H]^+$: 202.1226, Found 202.1224.

1-(1-isopropyl-1H-indol-2-yl)-2-methylprop-2-en-1-ol

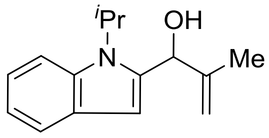

Colorless solid, mp: 40–42 °C. TLC R_f = 0.64 (PE/EA = 4:1, v/v), 95% yield. ^{1}H NMR (400 MHz, $CDCl_3$) δ 7.56–7.48 (m, 2H), 7.16–7.09 (m, 1H), 7.07–7.00 (m, 1H), 6.33 (s, 1H), 5.19 (d, *J* = 4.5 Hz, 1H), 5.17 (s, 1H), 5.06 (s, 1H), 4.88–4.76 (m, 1H), 2.39–2.24 (m, 1H), 1.69 (s, 3H), 1.58–1.53 (m, 6H); ^{13}C NMR (101 MHz, $CDCl_3$) δ 145.0, 139.1, 135.7, 128.2, 121.1, 121.0, 119.0, 112.1, 111.5, 101.0, 71.2, 47.4, 21.2, 21.1, 19.7. ESI-HRMS calcd for $[C_{15}H_{20}NO, M + H]^+$: 230.1539, Found 230.1540.

1-(1-allyl-1H-indol-2-yl)-2-methylprop-2-en-1-ol

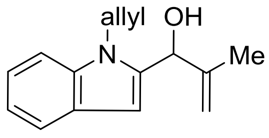

Colorless solid, mp: 66–67 °C. TLC R_f = 0.58 (PE/EA = 4:1, v/v), 88% yield. ^{1}H NMR (400 MHz, $CDCl_3$) δ 7.57 (d, *J* = 7.8 Hz, 1H), 7.25 (d, *J* = 8.2 Hz, 1H), 7.20–7.16 (m, 1H), 7.08 (t, *J* = 7.4 Hz, 1H), 6.42 (s, 1H), 5.97–5.86 (m, 1H), 5.23 (s, 1H), 5.20 (d, *J* = 4.6 Hz, 1H), 5.09 (d, *J* = 9.1 Hz, 2H), 4.88–4.71 (m, 3H), 2.22 (d, *J* = 4.8 Hz, 1H), 1.71 (s, 3H); ^{13}C NMR (101 MHz, $CDCl_3$) δ 144.8, 139.3, 137.5, 133.8, 127.2, 121.8, 120.8, 119.6, 116.0, 111.7, 109.6, 101.2, 70.7, 45.7, 19.5. ESI-HRMS calcd for $[C_{15}H_{18}NO, M + H]^+$: 228.1383, Found 228.1384.

1-(1-benzyl-1H-indol-2-yl)-2-methylprop-2-en-1-ol

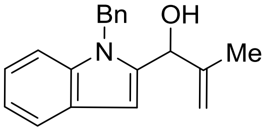

Yellow solid, mp: 79–80 °C. TLC R_f = 0.30 (PE/EA = 10:1, v/v), 93% yield. ^{1}H NMR (400 MHz, $CDCl_3$) δ 7.61 (d, *J* = 7.5 Hz, 1H), 7.27–7.19 (m, 4H), 7.17–7.08 (m, 2H), 6.95 (d, *J* = 6.9 Hz, 2H), 6.52 (s, 1H), 5.45 (dd, *J* = 39.7, 17.1 Hz, 2H), 5.20 (s, 1H), 5.18 (d, *J* = 3.7 Hz, 1H), 5.04 (d, *J* = 1.1 Hz, 1H), 1.96 (s, 1H), 1.68 (s, 3H); ^{13}C NMR (101 MHz, $CDCl_3$) δ 144.7, 139.7, 138.0, 137.9, 128.7, 127.3, 127.2, 125.8, 122.1, 120.8, 119.8, 111.9, 109.7, 101.4, 70.8, 46.8, 19.4. ESI-HRMS calcd for [$C_{19}H_{20}NO$, M + H]$^+$: 278.1539, Found 278.1542.

2-methyl-1-(1-phenyl-1H-indol-2-yl)prop-2-en-1-ol

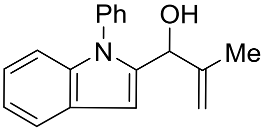

Light yellow solid, mp: 111–112 °C. TLC R_f = 0.47 (PE/EA = 8:1, v/v), 89% yield. ^{1}H NMR (400 MHz, $CDCl_3$) δ 7.65–7.63 (m, 1H), 7.54–7.45 (m, 5H), 7.18–7.08 (m, 3H), 6.64 (s, 1H), 5.08 (s, 1H), 5.01 (s, 1H), 4.94 (s, 1H), 1.92–1.73 (m, 1H), 1.69 (s, 3H); ^{13}C NMR (101 MHz, $CDCl_3$) δ 144.9, 141.1, 138.6, 137.4, 129.3, 128.4, 128.1, 127.3, 122.2, 120.7, 120.2, 111.7, 110.5, 101.2, 70.1, 19.0. ESI-HRMS calcd for [$C_{18}H_{18}NO$, M + H]$^+$: 264.1383, Found 264.1389.

1-(1-(4-methoxyphenyl)-1H-indol-2-yl)-2-methylprop-2-en-1-ol

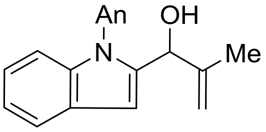

Light yellow solid, mp: 96–97 °C. TLC R_f = 0.48 (PE/EA = 4:1, v/v), 91% yield. ^{1}H NMR (400 MHz, $CDCl_3$) δ 7.63–7.57 (m, 1H), 7.29–7.19 (m, 2H), 7.14–7.09 (m, 2H), 7.07–7.02 (m, 1H), 6.96 (d, *J* = 8.1 Hz, 2H), 6.58 (s, 1H), 5.00 (d, *J* = 4.5 Hz, 1H), 4.97 (s, 1H), 4.90 (s, 1H), 3.84 (s, 3H), 2.12 (d, *J* = 5.0 Hz, 1H), 1.65 (s, 3H); ^{13}C NMR (101 MHz, $CDCl_3$) δ 159.2, 144.9, 141.4, 138.9, 129.9, 129.6, 127.2, 122.0, 120.6, 120.1, 114.4, 111.7, 110.4, 100.7, 70.2, 55.5, 19.0 . ESI-HRMS calcd for [$C_{19}H_{20}NO_2$, M + H]$^+$: 294.1489, Found 294.1492.

2-methyl-1-(1-(4-(trifluoromethyl)phenyl)-1H-indol-2-yl)prop-2-en-1-ol

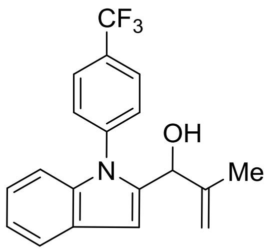

Light yellow solid, mp: 100–101 °C. TLC R_f = 0.41 (PE/EA = 8:1, v/v), 93% yield. ^{1}H NMR (400 MHz, $CDCl_3$) δ 7.76 (d, J = 8.5 Hz, 2H), 7.65–7.61 (m, 1H), 7.57–7.56 (m, 2H), 7.20–7.08 (m, 3H), 6.65 (s, 1H), 5.02 (s, 1H), 5.00 (s, 1H), 4.94 (d, J = 1.0 Hz, 1H), 2.11–2.02 (m, 1H), 1.65 (s, 3H); ^{13}C NMR (101 MHz, $CDCl_3$) δ 144.7, 140.8, 140.6, 138.4, 130.0 (q, J = 33.1 Hz), 128.7, 127.5, 126.5 (q, J = 3.5 Hz), 123.8 (q, J = 273.4 Hz), 122.8, 121.0, 120.8, 111.9, 110.2, 102.5, 70.1, 19.2. ESI-HRMS calcd for [$C_{19}H_{17}F_3NO$, M + H]$^+$: 332.1257, Found 332.1255.

2-methyl-1-(1-(naphthalen-2-yl)-1H-indol-2-yl)prop-2-en-1-ol

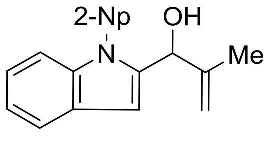

Yellow solid, mp: 136–137 °C. TLC R_f = 0.45 (PE/EA = 8:1, v/v), 94% yield. ^{1}H NMR (400 MHz, $CDCl_3$) δ 8.00–7.88 (m, 4H), 7.69–7.64 (m, 1H), 7.62–7.55 (m, 2H), 7.47 (s, 1H), 7.17–7.15 (m, 3H), 6.69 (s, 1H), 5.13 (d, J = 5.4 Hz, 1H), 5.02 (s, 1H), 4.94 (s, 1H), 1.91 (d, J = 5.4 Hz, 1H), 1.71 (s, 3H); ^{13}C NMR (101 MHz, $CDCl_3$) δ 145.0, 141.4, 138.8, 134.8, 133.5, 132.6, 129.3, 128.0, 127.8, 127.5, 126.9, 126.8, 126.7, 126.3, 122.3, 120.8, 120.4, 111.8, 110.5, 101.4, 70.2, 19.1. ESI-HRMS calcd for [$C_{22}H_{20}NO$, M + H]$^+$: 314.1539, Found 314.1538.

1-(1-methyl-1H-indol-2-yl)-2-methylenebutan-1-ol

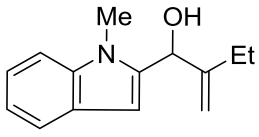

Yellow liquid, TLC R_f = 0.34 (PE/EA = 8:1, v/v), 95% yield. ^{1}H NMR (400 MHz, $CDCl_3$) δ 7.57 (d, J = 7.8 Hz, 1H), 7.29 (d, J = 8.2 Hz, 1H), 7.24 ‒ 7.07 (m, 1H), 7.10 ‒ 7.07 (m, 1H), 6.41 (s, 1H), 5.35 (d, J = 4.7 Hz, 1H), 5.26 (s, 1H), 5.11 (s, 1H), 3.74 (s, 3H), 2.15–1.96 (m, 3H), 1.05 (t, J = 7.4 Hz, 3H); ^{13}C NMR (101 MHz, $CDCl_3$) δ 150.7, 139.9, 138.2, 127.1, 121.8, 120.7, 119.5, 109.7, 109.0, 101.0, 70.6, 30.1, 25.6, 12.2. ESI-HRMS calcd for [$C_{14}H_{18}NO$, M + H]$^+$: 216.1383, Found 216.1380.

3-methyl-1-(1-methyl-1H-indol-2-yl)-2-methylenebutan-1-ol

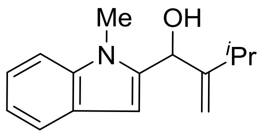

Yellow solid, mp: 61–62 °C. TLC R_f = 0.75 (PE/EA = 10:1, v/v), 88% yield. ^{1}H NMR (400 MHz, $CDCl_3$) δ 7.57 (d, *J* = 7.8 Hz, 1H), 7.29 (d, *J* = 8.0 Hz, 1H), 7.21 (dd, *J* = 10.4, 4.6 Hz, 1H), 7.09 (dd, *J* = 7.7, 7.1 Hz, 1H), 6.39 (s, 1H), 5.37 (d, *J* = 4.8 Hz, 1H), 5.25 (s, 1H), 5.15 (s, 1H), 3.71 (s, 3H), 2.30–2.19 (m, 1H), 2.08 (d, *J* = 5.2 Hz, 1H), 1.05 (dd, *J* = 9.4, 6.9 Hz, 6H); ^{13}C NMR (101 MHz, $CDCl_3$) δ 155.7, 140.1, 138.2, 127.0, 121.7, 120.7, 119.4, 109.0, 108.5, 101.1, 69.6, 30.6, 30.1, 23.0, 21.9. ESI-HRMS calcd for [$C_{15}H_{20}NO$, M + H]$^+$: 230.1539, Found 230.1545.

1-(1-methyl-1H-indol-2-yl)-2-phenylprop-2-en-1-ol

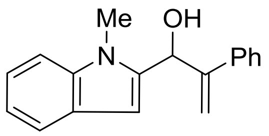

Yellow solid, mp: 96–97 °C. TLC R_f = 0.53 (PE/EA = 4:1, v/v), 98% yield. ^{1}H NMR (400 MHz, $CDCl_3$) δ 7.54 (d, *J* = 7.9 Hz, 1H), 7.40 (d, *J* = 7.4 Hz, 2H), 7.32–7.19 (m, 5H), 7.07 (t, *J* = 7.4 Hz, 1H), 6.43 (s, 1H), 5.88 (d, *J* = 5.4 Hz, 1H), 5.66 (s, 1H), 5.51 (s, 1H), 3.80 (s, 3H), 2.14 (d, *J* = 5.6 Hz, 1H); ^{13}C NMR (101 MHz, $CDCl_3$) δ 148.6, 139.9, 139.2, 138.1, 128.4, 127.8, 127.0, 126.5, 121.8, 120.9, 119.4, 114.6, 109.1, 101.7, 68.7, 30.0. ESI-HRMS calcd for [$C_{18}H_{18}NO$, M + H]$^+$: 264.1383, Found 264.1379.

2-(4-methoxyphenyl)-1-(1-methyl-1H-indol-2-yl)prop-2-en-1-ol

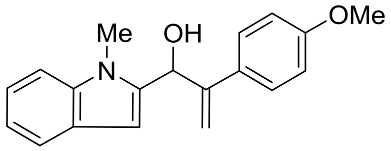

Light green solid, mp: 118–119 °C. TLC R_f = 0.39 (PE/EA = 4:1, v/v), 99% yield. ^{1}H NMR (400 MHz, $CDCl_3$) δ 7.53 (d, *J* = 7.8 Hz, 1H), 7.33–7.28 (m, 3H), 7.21 (dd, *J* = 13.2, 5.9 Hz, 1H), 7.07 (t, *J* = 7.4 Hz, 1H), 6.80 (d, *J* = 8.4 Hz, 2H), 6.41 (s, 1H), 5.81 (d, *J* = 5.3 Hz, 1H), 5.57 (s, 1H), 5.40 (s, 1H), 3.76–3.75 (m, 6H), 2.29–2.20 (m, 1H); ^{13}C NMR (101 MHz, $CDCl_3$) δ 159.3, 148.0, 140.1, 138.1, 131.7, 127.6, 127.1, 121.8, 120.8, 119.4, 113.8, 113.1, 109.1, 101.7, 68.9, 55.2, 30.1. ESI-HRMS calcd for [$C_{19}H_{20}NO_2$, M + H]$^+$: 294.1489, Found 294.1497.

2-(4-fluorophenyl)-1-(1-methyl-1H-indol-2-yl)prop-2-en-1-ol

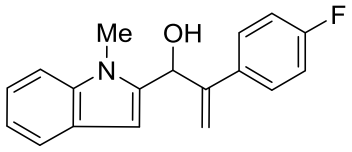

Yellow solid, mp: 109–110 °C. TLC R_f = 0.41 (PE/EA = 4:1, v/v), 98% yield. ^{1}H NMR (400 MHz, $CDCl_3$) δ 7.50 (d, J = 7.9 Hz, 1H), 7.30–7.23 (m, 3H), 7.21–7.16 (m, 1H), 7.08–7.03 (m, 1H), 6.95–6.88 (m, 2H), 6.34 (s, 1H), 5.67 (d, J = 5.4 Hz, 1H), 5.55 (s, 1H), 5.44 (s, 1H), 3.66 (s, 3H), 2.55 (d, J = 5.7 Hz, 1H); ^{13}C NMR (101 MHz, $CDCl_3$) δ 162.3 (d, J = 248.1 Hz), 147.5, 139.6, 138.1, 135.3 (d, J = 3.3 Hz), 128.1 (d, J = 8.0 Hz), 126.9, 121.9, 120.8, 119.5, 115.2 (d, J = 21.6 Hz), 114.5, 109.1, 101.7, 68.7, 29.9. ESI-HRMS calcd for [$C_{18}H_{17}FNO$, M + H]$^+$: 282.1289, Found 282.1284.

2-(4-chlorophenyl)-1-(1-methyl-1H-indol-2-yl)prop-2-en-1-ol

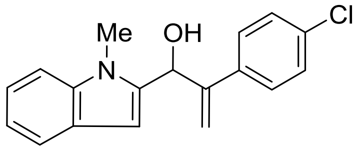

Yellow solid, mp: 130–131 °C. TLC R_f = 0.43 (PE/EA = 4:1, v/v), 99% yield. ^{1}H NMR (400 MHz, $CDCl_3$) δ 7.53 (d, J = 7.9 Hz, 1H), 7.32–7.28 (m, 3H), 7.25–7.19 (m, 3H), 7.07 (t, J = 7.4 Hz, 1H), 6.38 (s, 1H), 5.80 (d, J = 5.7 Hz, 1H), 5.64 (s, 1H), 5.53 (s, 1H), 3.77 (s, 3H), 2.19 (t, J = 4.7 Hz, 1H); ^{13}C NMR (101 MHz, $CDCl_3$) δ 147.5, 139.5, 138.1, 137.6, 133.6, 128.5, 127.8, 126.9, 122.0, 120.9, 119.6, 115.1, 109.1, 101.8, 68.7, 30.1. ESI-HRMS calcd for [$C_{18}H_{17}NOCl$, M + H]$^+$: 298.0993, Found 298.0997.

2-(benzo[d][1,3]dioxol-5-yl)-1-(1-methyl-1H-indol-2-yl)prop-2-en-1-ol

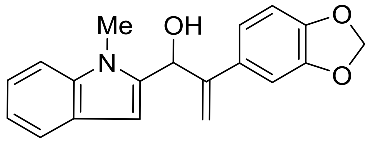

Yellow solid, mp: 116–117 °C. TLC R_f = 0.59 (PE/EA = 4:1, v/v), 99% yield. ^{1}H NMR (400 MHz, $CDCl_3$) δ 7.53 (d, J = 7.8 Hz, 1H), 7.28 (d, J = 8.2 Hz, 1H), 7.20 (t, J = 7.6 Hz, 1H), 7.07 (t, J = 7.4 Hz, 1H), 6.90 (s, 1H), 6.85 (d, J = 8.1 Hz, 1H), 6.70 (d, J = 8.1 Hz, 1H), 6.39 (s, 1H), 5.90 (s, 2H), 5.76 (d, J = 5.6 Hz, 1H), 5.55 (s, 1H), 5.40 (s, 1H), 3.74 (s, 3H), 2.27 (d, J = 5.1 Hz, 1H); ^{13}C NMR (101 MHz, $CDCl_3$) δ 148.1, 147.6, 147.2, 139.9, 138.1, 133.4, 127.0, 121.9, 120.8, 120.0, 119.5, 113.8, 109.1, 108.1, 107.1, 101.7, 101.0, 68.9, 30.1. ESI-HRMS calcd for [$C_{19}H_{18}NO_3$, M + H]$^+$: 308.1281, Found 308.1280.

1-(1,5-dimethyl-1H-indol-2-yl)-3-methyl-2-methylenebutan-1-ol

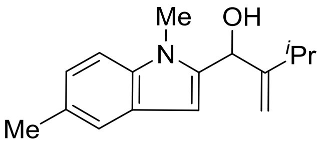

Yellow sticky oil. TLC R_f = 0.28 (PE/EA = 10:1, v/v), 92% yield. ^{1}H NMR (400 MHz, $CDCl_3$) δ 7.31 (d, J = 0.6 Hz, 1H), 7.12 (d, J = 8.7 Hz, 1H), 7.00 (d, J = 8.4 Hz, 1H), 6.24 (s, 1H), 5.23 (d, J = 5.1 Hz, 1H), 5.17 (s, 1H), 5.09 (s, 1H), 3.57 (s, 3H), 2.41–2.39 (m, 4H), 2.19 (dt, J = 13.6, 6.8 Hz, 1H), 1.00 (dd, J = 8.8, 6.9 Hz, 6H); ^{13}C NMR (101 MHz, $CDCl_3$) δ 155.6, 140.1, 136.5, 128.5, 127.2, 123.2, 120.2, 108.7, 108.4, 100.4, 69.5, 30.5, 29.9, 22.9, 21.9, 21.3. ESI-HRMS calcd for $[C_{16}H_{22}NO, M + H]^+$: 244.1696, Found 244.1698.

1-(5-fluoro-1-methyl-1H-indol-2-yl)-3-methylbutan-1-one

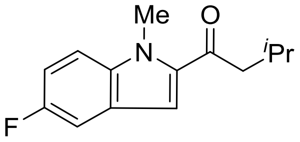

Light green solid, mp: 32–33 °C. TLC R_f = 0.53 (PE/EA = 30:1, v/v), 91% yield. ^{1}H NMR (400 MHz, $CDCl_3$) δ 7.34–7.28 (m, 2H), 7.21 (s, 1H), 7.13 (td, J = 9.1, 2.4 Hz, 1H), 4.05 (s, 3H), 2.80 (d, J = 7.0 Hz, 2H), 2.31 (dp, J = 13.5, 6.7 Hz, 1H), 1.01 (d, J = 6.7 Hz, 6H); ^{13}C NMR (101 MHz, $CDCl_3$) δ 194.3, 158.1(d, J = 237.8 Hz), 136.7, 136.5, 125.7 (d, J = 10.4 Hz), 114.8 (d, J = 27.2 Hz), 111.3 (d, J = 9.5 Hz), 110.7 (d, J = 5.5 Hz), 106.7 (d, J = 23.1 Hz), 49.0, 32.4, 25.9, 22.7. ESI-HRMS calcd for $[C_{14}H_{17}FNO, M + H]^+$: 234.1289, Found 234.1291.

1-(5-chloro-1-methyl-1H-indol-2-yl)-3-methylbutan-1-one

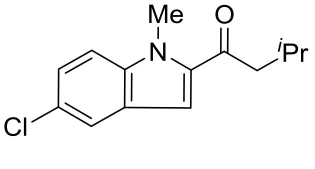

Light yellow solid, mp: 39–40 °C. TLC R_f = 0.47 (PE/EA = 15:1, v/v), 94% yield. ^{1}H NMR (400 MHz, $CDCl_3$) δ 7.63 (t, J = 1.6 Hz, 1H), 7.29 (d, J = 1.6 Hz, 2H), 7.18 (s, 1H), 4.04 (s, 3H), 2.80 (d, J = 7.0 Hz, 2H), 2.31 (dp, J = 13.5, 6.8 Hz, 1H), 1.01 (d, J = 6.8 Hz, 6H); ^{13}C NMR (101 MHz, $CDCl_3$) δ 194.3, 138.2, 136.1, 126.5, 126.2, 126.1, 121.8, 111.5, 110.3, 49.0, 32.4, 25.9, 22.7. ESI-HRMS calcd for $[C_{14}H_{17}ClNO, M + H]^+$: 250.0993, Found 250.0997.

1-(4-fluoro-1-methyl-1H-indol-2-yl)-3-methylbutan-1-one

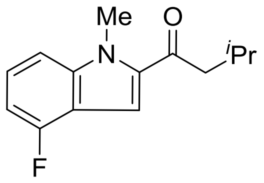

Light yellow solid, mp: 27–28 °C. TLC R_f = 0.41 (PE/EA = 40:1, v/v), 93% yield. ^{1}H NMR (400 MHz, $CDCl_3$) δ 7.33 (s, 1H), 7.31–7.24 (m, 1H), 7.14 (d, J = 8.4 Hz, 1H), 6.79 (dd, J = 9.9, 7.8 Hz, 1H), 4.06 (s, 3H), 2.82 (d, J = 7.1 Hz, 2H), 2.32 (dp, J = 13.5, 6.7 Hz, 1H), 1.02 (d, J = 6.7 Hz, 6H); ^{13}C NMR (101 MHz, $CDCl_3$) δ 194.2, 157.4 (d, J = 252.0 Hz), 142.1 (d, J = 10.0 Hz), 135.3, 126.2 (d, J = 7.9 Hz), 115.5 (d, J = 22.8 Hz), 107.0, 106.4 (d, J = 4.0 Hz), 104.8 (d, J = 18.6 Hz), 48.9, 32.7, 26.0, 22.7. ESI-HRMS calcd for $[C_{14}H_{17}FNO, M + H]^+$: 234.1289, Found 234.1286.

2.3 Analytical Data of Substituted Indole Enones

2-methyl-1-(1-methyl-1H-indol-2-yl)prop-2-en-1-one (2a)

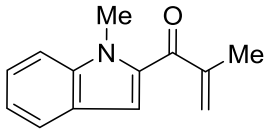

Greenish yellow solid, mp: 77–78 °C. TLC R_f = 0.80 (PE/EA = 10:1, v/v), 82% yield. ^{1}H NMR (400 MHz, $CDCl_3$) δ 7.67 (d, J = 8.0 Hz, 1H), 7.40–7.34 (m, 2H), 7.15 (ddd, J = 8.0, 5.6, 2.3 Hz, 1H), 7.09 (s, 1H), 5.85 (s, 1H), 5.83–5.81 (m, 1H), 4.01 (s, 3H), 2.09 (s, 3H); ^{13}C NMR (101 MHz, $CDCl_3$) δ 190.4, 145.2, 140.4, 134.8, 125.8, 125.6, 125.1, 122.9, 120.6, 113.5, 110.2, 31.8, 18.7. IR (film): $\tilde{\nu}$ 3122, 3050, 2962, 2924, 2848, 1629, 800, 753, 743 cm^{-1}. ESI-HRMS calcd for $[C_{13}H_{14}NO, M + H]^+$: 200.1070, Found 200.1070.

1-(1-isopropyl-1H-indol-2-yl)-2-methylprop-2-en-1-one (2b)

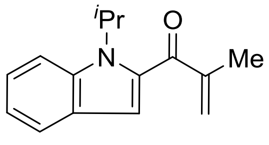

Yellow solid, mp: 31–32 °C. TLC R_f = 0.58 (PE/EA = 20:1, v/v), 84% yield. ^{1}H NMR (400 MHz, $CDCl_3$) δ 7.71 (d, J = 8.0 Hz, 1H), 7.65 (d, J = 8.6 Hz, 1H), 7.38–7.32 (m, 1H), 7.17 (t, J = 7.5 Hz, 1H), 7.03 (s, 1H), 5.93–5.92 (m, 2H), 5.38 (hept, J = 7.0 Hz, 1H), 2.14 (s, 3H), 1.70 (d, J = 7.0 Hz, 6H); ^{13}C NMR (101 MHz, $CDCl_3$) δ 190.6, 145.6, 138.4, 135.4, 126.6, 126.3, 124.7, 123.1, 120.1, 113.7, 112.9, 48.3, 21.5, 18.5. IR (film): $\tilde{\nu}$ 3003, 2975, 2934, 2878, 1638, 1623, 789, 757, 747 cm^{-1}. ESI-HRMS calcd for $[C_{15}H_{18}NO, M + H]^+$: 228.1383, Found 228.1385.

1-(1-allyl-1H-indol-2-yl)-2-methylprop-2-en-1-one (2c)

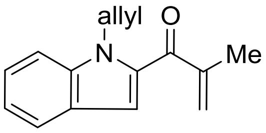

Greenish yellow liquid. TLC R_f = 0.42 (PE/EA = 20:1, v/v), 86% yield. ^{1}H NMR (400 MHz, $CDCl_3$) δ 7.69 (d, J = 8.0 Hz, 1H), 7.41–7.33 (m, 2H), 7.19–7.14 (m, 1H), 7.12 (s, 1H), 6.02 (ddt, J = 16.8, 10.2, 5.1 Hz, 1H), 5.84–5.82 (m, 2H), 5.15–5.08 (m, 3H), 4.93 (dd, J = 17.2, 1.1 Hz, 1H), 2.09 (s, 3H); ^{13}C NMR (101 MHz, $CDCl_3$) δ 190.2, 145.1, 139.8, 134.3, 134.0, 125.8, 125.7, 125.3, 122.9, 120.7, 116.0, 114.0, 110.7, 46.9, 18.7. IR (film): $\tilde{\nu}$ 3083, 3062, 3033, 2983, 2955, 2924, 2852, 1638, 1622, 793, 752, 738 cm^{-1}. ESI-HRMS calcd for [$C_{15}H_{16}NO$, M + H]$^+$: 226.1226, Found 226.1228.

1-(1-benzyl-1H-indol-2-yl)-2-methylprop-2-en-1-one (2d)

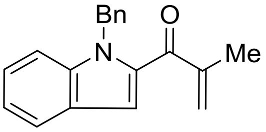

Yellow solid, mp: 62–63 °C. TLC R_f = 0.74 (PE/EA = 10:1, v/v), 88% yield. ^{1}H NMR (400 MHz, $CDCl_3$) δ 7.70 (d, J = 8.0 Hz, 1H), 7.37–7.29 (m, 2H), 7.26–7.14 (m, 5H), 7.05 (d, J = 7.3 Hz, 2H), 5.82 (d, J = 2.9 Hz, 2H), 5.76 (s, 2H), 2.06 (s, 3H); ^{13}C NMR (101 MHz, $CDCl_3$) δ 190.3, 145.1, 140.1, 138.3, 134.5, 128.5, 127.1, 126.4, 126.0, 125.9, 125.4, 122.9, 120.9, 114.3, 110.9, 48.0, 18.7. IR (film): $\tilde{\nu}$ 3101, 3084, 3061, 3030, 2952, 2921, 2850, 1635, 1621, 793, 753, 725, 694 cm^{-1}. ESI-HRMS calcd for [$C_{19}H_{18}NO$, M + H]$^+$: 276.1383, Found 276.1385.

2-methyl-1-(1-phenyl-1H-indol-2-yl)prop-2-en-1-one (2e)

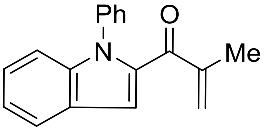

White solid, mp: 59–60 °C. TLC R_f = 0.59 (PE/EA = 20:1, v/v), 75% yield. ^{1}H NMR (400 MHz, $CDCl_3$) δ 7.73 (d, J = 8.0 Hz, 1H), 7.49 (t, J = 7.6 Hz, 2H), 7.41 (t, J = 7.3 Hz, 1H), 7.33–7.27 (m, 3H), 7.24–7.17 (m, 3H), 6.01 (s, 1H), 5.88 (s, 1H), 1.99 (s, 3H); ^{13}C NMR (101 MHz, $CDCl_3$) δ 188.9, 145.2, 140.6, 138.6, 135.7, 129.2, 127.6, 127.2, 126.1, 125.9, 125.9, 122.7, 121.3, 113.9, 111.4, 18.3. IR (film): $\tilde{\nu}$ 3109, 3057, 3029, 2986, 2957, 2918, 1973, 1907, 1873, 1790, 1639, 796, 763, 742, 696 cm^{-1}. ESI-HRMS calcd for [$C_{18}H_{16}NO$, M + H]$^+$: 262.1226, Found 262.1228.

1-(1-(4-methoxyphenyl)-1H-indol-2-yl)-2-methylprop-2-en-1-one (2f)

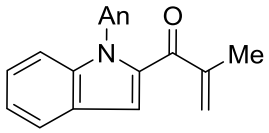

Light yellow solid, mp: 95–96 °C. TLC R_f = 0.50 (PE/EA = 10:1, v/v), 73% yield. ^{1}H NMR (400 MHz, $CDCl_3$) δ 7.72 (d, *J* = 8.3 Hz, 1H), 7.32–7.27 (m, 1H), 7.26–7.16 (m, 5H), 7.02–6.98 (m, 2H), 5.99 (s, 1H), 5.88 (s, 1H), 3.86 (s, 3H), 2.00 (s, 3H); ^{13}C NMR (101 MHz, $CDCl_3$) δ 188.9, 158.8, 145.1, 140.9, 135.6, 131.3, 128.3, 125.9, 125.8, 125.7, 122.6, 121.2, 114.3, 113.5, 111.4, 55.4, 18.3. IR (film): $\tilde{\nu}$ 3093, 3064, 2983, 2952, 2936, 2835, 1942, 1902, 1871, 1821, 1643, 1513, 799, 788, 748 cm^{-1}. ESI-HRMS calcd for [$C_{19}H_{18}NO_2$, M + H]$^+$: 292.1332, Found 292.1335.

2-methyl-1-(1-(4-(trifluoromethyl)phenyl)-1H-indol-2-yl)prop-2-en-1-one (2g)

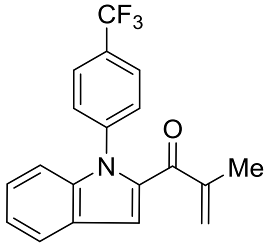

White solid, mp: 144–145 °C. TLC R_f = 0.58 (PE/EA = 50:1, v/v), 74% yield. ^{1}H NMR (400 MHz, $CDCl_3$) δ 7.82–7.79 (m, 3H), 7.49 (d, *J* = 8.2 Hz, 2H), 7.41–7.36 (m, 1H), 7.32–7.26 (m, 3H), 6.11 (s, 1H), 5.99 (s, 1H), 2.07 (s, 3H); ^{13}C NMR (101 MHz, $CDCl_3$) δ 188.6, 144.9, 141.8, 140.3, 135.4, 129.5 (q, *J* = 32.9 Hz), 127.5, 126.5, 126.4 (q, *J* = 4.0 Hz), 126.4, 126.2, 123.9 (q, *J* = 273.2 Hz), 123.0, 121.8, 115.1, 111.0, 18.3. IR (film): $\tilde{\nu}$ 3134, 3094, 3064, 1947, 1930, 1869, 1640, 1122, 1104, 1070, 827, 790, 749, 719 cm^{-1}. ESI-HRMS calcd for [$C_{19}H_{15}F_3NO$, M + H]$^+$: 330.1100, Found 330.1104.

2-methyl-1-(1-(naphthalen-2-yl)-1H-indol-2-yl)prop-2-en-1-one (2h)

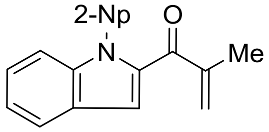

Light yellow solid, mp: 116–117 °C. TLC R_f = 0.42 (PE/EA = 15:1, v/v), 73% yield. ^{1}H NMR (400 MHz, $CDCl_3$) δ 7.93 (d, *J* = 8.7 Hz, 1H), 7.91–7.87 (m, 1H), 7.83 (dd, *J* = 5.7, 3.2 Hz, 1H), 7.80 (s, 1H), 7.74 (d, *J* = 7.9 Hz, 1H), 7.51 (dd, *J* = 6.1, 3.2 Hz, 2H), 7.38 (dd, *J* = 8.6, 1.7 Hz, 1H), 7.31–7.17 (m, 4H), 6.05 (s, 1H), 5.88 (s, 1H), 1.97 (s, 3H); ^{13}C NMR (101 MHz, $CDCl_3$) δ 188.8, 145.1, 140.7, 136.1, 135.8, 133.4, 132.5, 129.1, 128.0, 127.8, 126.6, 126.3, 126.2, 126.0, 125.8, 125.2, 122.8, 121.4, 114.1, 111.4,

18.3. IR (film): $\tilde{\nu}$ 3106, 3059, 3028, 2961, 2921, 1954, 1910, 1825, 1787, 1638, 817, 756 cm^{-1}. ESI-HRMS calcd for [$C_{22}H_{18}NO$, M + H]$^+$: 312.1383, Found 312.1385.

1-(1-methyl-1H-indol-2-yl)-2-methylenebutan-1-one (2i)

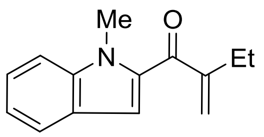

Yellow liquid, TLC R_f = 0.81 (PE/EA = 20:1, v/v), 83% yield. ^{1}H NMR (400 MHz, $CDCl_3$) δ 7.67 (d, J = 8.0 Hz, 1H), 7.41–7.34 (m, 2H), 7.15 (ddd, J = 8.0, 5.4, 2.4 Hz, 1H), 7.09 (s, 1H), 5.78 (s, 1H), 5.73 (d, J = 1.2 Hz, 1H), 4.03 (s, 3H), 2.51 (q, J = 7.4 Hz, 2H), 1.14 (t, J = 7.4 Hz, 3H); ^{13}C NMR (101 MHz, $CDCl_3$) δ 190.7, 151.2, 140.4, 135.3, 125.7, 125.7, 122.9, 122.3, 120.6, 113.8, 110.3, 31.8, 25.4, 12.5. IR (film): $\tilde{\nu}$ 3058, 2966, 2935, 2874, 1637, 1619, 792, 753, 740 cm^{-1}. ESI-HRMS calcd for [$C_{14}H_{16}NO$, M + H]$^+$: 214.1226, Found 214.1227.

3-methyl-1-(1-methyl-1H-indol-2-yl)-2-methylenebutan-1-one (2j)

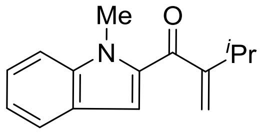

Yellow solid, mp: 50–51 °C. TLC R_f = 0.59 (PE/EA = 50:1, v/v), 93% yield. ^{1}H NMR (400 MHz, $CDCl_3$) δ 7.67 (d, J = 8.0 Hz, 1H), 7.40–7.33 (m, 2H), 7.14 (ddd, J = 7.9, 5.0, 2.8 Hz, 1H), 7.08 (s, 1H), 5.69 (s, 1H), 5.64 (s, 1H), 4.05 (s, 3H), 3.05 (hept, J = 6.8 Hz, 1H), 1.15 (d, J = 6.9 Hz, 6H); ^{13}C NMR (101 MHz, $CDCl_3$) δ 191.3, 155.8, 140.4, 135.7, 125.8, 125.6, 122.9, 120.6, 119.8, 114.1, 110.2, 31.9, 29.9, 21.4. IR (film): $\tilde{\nu}$ 3090, 3049, 2969, 2950, 2868, 1632, 1617, 791, 773, 752, 728 cm^{-1}. ESI-HRMS calcd for [$C_{15}H_{18}NO$, M + H]$^+$: 228.1383, Found 228.1388.

1-(1-methyl-1H-indol-2-yl)-2-phenylprop-2-en-1-one (2k)

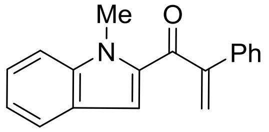

Yellow solid, mp: 80–81 °C. TLC R_f = 0.53 (PE/EA = 10:1, v/v), 79% yield. ^{1}H NMR (400 MHz, $CDCl_3$) δ 7.65 (d, J = 8.1 Hz, 1H), 7.47 (dd, J = 8.0, 1.4 Hz, 2H), 7.41–7.31 (m, 5H), 7.18 (s, 1H), 7.15 (ddd, J = 7.9, 5.3, 2.5 Hz, 1H), 6.06 (s, 1H), 5.79 (s, 1H), 4.14 (s, 3H); ^{13}C NMR (101 MHz, $CDCl_3$) δ 190.1, 149.1, 140.7, 137.4, 135.3, 128.5, 128.3, 127.2, 126.3, 125.7, 123.2, 120.8, 120.7, 115.4, 110.4, 32.1. IR (film): $\tilde{\nu}$ 3093, 3040,

3032, 2949, 1641, 787, 746, 729, 713, 701 cm^{-1}. ESI-HRMS calcd for $[C_{18}H_{16}NO, M + H]^+$: 262.1226, Found 262.1228.

2-(4-methoxyphenyl)-1-(1-methyl-1H-indol-2-yl)prop-2-en-1-one (2l)

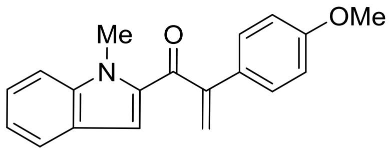

Yellow solid, mp: 74–75 °C. TLC R_f = 0.39 (PE/EA = 20:1, v/v), 77% yield. ^{1}H NMR (400 MHz, $CDCl_3$) δ 7.65 (d, *J* = 8.0 Hz, 1H), 7.41–7.39 (m, 4H), 7.18 (s, 1H), 7.14 (t, *J* = 6.6 Hz, 1H), 6.89 (d, *J* = 8.4 Hz, 2H), 5.98 (s, 1H), 5.69 (s, 1H), 4.13 (s, 3H), 3.80 (s, 3H); ^{13}C NMR (101 MHz, $CDCl_3$) δ 190.4, 159.7, 148.4, 140.6, 135.3, 129.9, 128.4, 126.2, 125.7, 123.2, 120.7, 119.0, 115.3, 113.9, 110.3, 55.3, 32.1. IR (film): $\tilde{\nu}$ 3123, 3084, 3009, 2966, 2947, 2836, 1630, 1606, 1509, 846, 805, 788, 742 cm^{-1}. ESI-HRMS calcd for $[C_{19}H_{18}NO_2, M + H]^+$: 292.1332, Found 292.1333.

2-(4-fluorophenyl)-1-(1-methyl-1H-indol-2-yl)prop-2-en-1-one (2m)

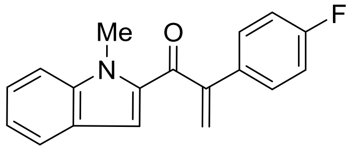

Bright yellow solid, mp: 65–66 °C. TLC R_f = 0.44 (PE/EA = 20:1, v/v), 80% yield. ^{1}H NMR (400 MHz, $CDCl_3$) δ 7.66 (dd, *J* = 8.1, 0.7 Hz, 1H), 7.47–7.38 (m, 4H), 7.19–7.11 (m, 2H), 7.05 (t, *J* = 8.5 Hz, 2H), 6.02 (s, 1H), 5.80 (s, 1H), 4.12 (s, 3H); ^{13}C NMR (101 MHz, $CDCl_3$) δ 189.7, 162.8 (d, *J* = 248.9 Hz), 148.0, 140.7, 135.1, 133.5 (d, *J* = 3.2 Hz), 129.0 (d, *J* = 8.2 Hz), 126.4, 125.7, 123.2, 120.9, 120.9, 115.5 (d, *J* = 21.7 Hz), 115.4, 110.4, 32.1. IR (film): $\tilde{\nu}$ 3105, 3060, 2951, 1632, 1601, 1505, 1464, 981, 843, 754, 739 cm^{-1}. ESI-HRMS calcd for $[C_{18}H_{15}FNO, M + H]^+$: 280.1132, Found 280.1135.

2-(4-chlorophenyl)-1-(1-methyl-1H-indol-2-yl)prop-2-en-1-one (2n)

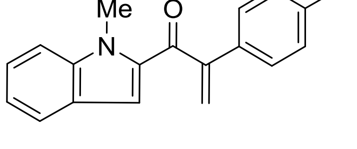

Yellow solid, mp: 101–102 °C. TLC R_f = 0.47 (PE/EA = 20:1, v/v), 79% yield. ^{1}H NMR (400 MHz, $CDCl_3$) δ 7.65 (d, *J* = 8.1 Hz, 1H), 7.43–7.39 (m, 4H), 7.33 (d, *J* = 8.5 Hz, 2H), 7.18–7.12 (m, 2H), 6.05 (s, 1H), 5.82 (s, 1H), 4.12 (s, 3H); ^{13}C NMR (101 MHz, $CDCl_3$) δ 189.5, 147.9, 140.8, 135.8, 135.1, 134.3, 128.7, 128.6, 126.5,

125.7, 123.2, 121.3, 120.9, 115.5, 110.4, 32.1. IR (film): ṽ 3065, 2951, 1639, 1614, 987, 915, 843, 748, 729, 647 cm^{-1}. ESI-HRMS calcd for $[C_{18}H_{15}ClNO, M + H]^+$: 296.0837, Found 296.0828.

2-(benzo[d][1,3]dioxol-5-yl)-1-(1-methyl-1H-indol-2-yl)prop-2-en-1-one (2o)

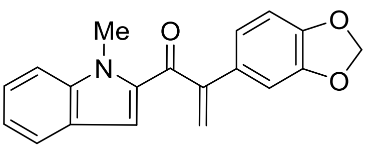

Colorless solid, mp: 117–118 °C. TLC R_f = 0.61 (PE/EA = 10:1, v/v), 76% yield. ^1H NMR (400 MHz, $CDCl_3$) δ 7.65 (d, *J* = 8.1 Hz, 1H), 7.42–7.36 (m, 2H), 7.17 (s, 1H), 7.14 (ddd, *J* = 8.0, 4.9, 3.0 Hz, 1H), 6.97 (d, *J* = 1.7 Hz, 1H), 6.93 (dd, *J* = 8.1, 1.8 Hz, 1H), 6.78 (d, *J* = 8.1 Hz, 1H), 5.95 (s, 2H), 5.93 (s, 1H), 5.68 (s, 1H), 4.13 (s, 3H); ^{13}C NMR (101 MHz, $CDCl_3$) δ 190.0, 148.7, 147.9, 147.8, 140.7, 135.2, 131.6, 126.3, 125.7, 123.2, 121.3, 120.8, 119.2, 115.4, 110.3, 108.3, 107.5, 101.2, 32.0. IR (film): ṽ 3134, 3087, 2897, 2788, 1622, 1609, 1501, 1486, 894, 870, 743, 691 cm^{-1}. ESI-HRMS calcd for $[C_{19}H_{16}NO_3, M + H]^+$: 306.1125, Found 306.1126.

1-(1,5-dimethyl-1H-indol-2-yl)-3-methyl-2-methylenebutan-1-one (2p)

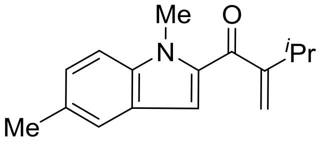

Greenish yellow oil. TLC R_f = 0.66 (PE/EA = 20:1, v/v), 87% yield. ^1H NMR (400 MHz, $CDCl_3$) δ 7.43 (s, 1H), 7.28 (d, *J* = 8.6 Hz, 1H), 7.21 (d, *J* = 8.5 Hz, 1H), 7.00 (s, 1H), 5.68 (s, 1H), 5.63 (s, 1H), 4.03 (s, 3H), 3.04 (hept, *J* = 6.8 Hz, 1H), 2.44 (s, 3H), 1.14 (d, *J* = 6.9 Hz, 6H); ^{13}C NMR (101 MHz, $CDCl_3$) δ 191.4, 155.8, 139.1, 135.7, 129.9, 127.8, 125.8, 122.1, 119.6, 113.6, 110.0, 31.9, 29.9, 21.4, 21.3. IR (film): ṽ 3092, 3021, 2962, 2871, 1636, 1621, 793 cm^{-1}. ESI-HRMS calcd for $[C_{16}H_{20}NO, M + H]^+$: 242.1539, Found 242.1541.

1-(5-fluoro-1-methyl-1H-indol-2-yl)-3-methyl-2-methylenebutan-1-one (2q)

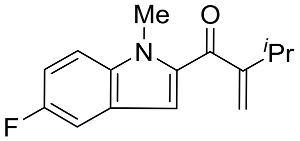

Green liquid. TLC R_f = 0.54 (PE/EA = 20:1, v/v), 68% yield. ^1H NMR (400 MHz, $CDCl_3$) δ 7.34–7.27 (m, 2H), 7.13 (td, *J* = 9.1, 2.2 Hz, 1H), 7.01 (s, 1H), 5.71 (s, 1H), 5.67 (s, 1H), 4.03 (s, 3H), 3.04 (hept, *J* = 6.4 Hz, 1H), 1.14 (d, *J* = 6.9 Hz, 6H); ^{13}C NMR (101

MHz, $CDCl_3$) δ 191.1, 158.0 (d, *J* = 237.7 Hz), 155.6, 137.0, 136.8, 125.5 (d, *J* = 10.2 Hz), 120.3, 114.7 (d, *J* = 27.1 Hz), 113.3 (d, *J* = 5.5 Hz), 111.2 (d, *J* = 9.5 Hz), 106.8 (d, *J* = 23.1 Hz), 32.0, 29.8, 21.3. IR (film): $\tilde{\nu}$ 3095, 3070, 2963, 2872, 1642, 1516, 1470, 1391, 1193, 986, 953, 925, 858, 790, 768 cm^{-1}. ESI-HRMS calcd for $[C_{15}H_{17}FNO, M + H]^+$: 246.1289, Found 246.1294.

1-(5-chloro-1-methyl-1H-indol-2-yl)-3-methyl-2-methylenebutan-1-one (2r)

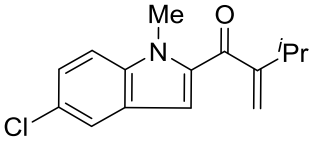

Light green solid, mp: 54–55 °C. TLC R_f = 0.47 (PE/EA = 15:1, v/v), 66% yield. ^1H NMR (400 MHz, $CDCl_3$) δ 7.63 (s, 1H), 7.32 (s, 2H), 6.99 (s, 1H), 5.71 (s, 1H), 5.69 (d, *J* = 0.8 Hz, 1H), 4.03 (s, 3H), 3.04 (hept, *J* = 6.8 Hz, 1H), 1.14 (d, *J* = 6.9 Hz, 6H); ^{13}C NMR (101 MHz, $CDCl_3$) δ 191.2, 155.6, 138.6, 136.6, 126.4, 126.2, 126.1, 121.9, 120.5, 112.9, 111.4, 32.1, 29.8, 21.4. IR (film): $\tilde{\nu}$ 3133, 2961, 2922, 2864, 1638, 1507, 1460, 912, 788, 746, 700 cm^{-1}. ESI-HRMS calcd for $[C_{15}H_{17}ClNO, M + H]^+$: 262.0993, Found 262.0996.

1-(4-fluoro-1-methyl-1H-indol-2-yl)-3-methyl-2-methylenebutan-1-one (2s)

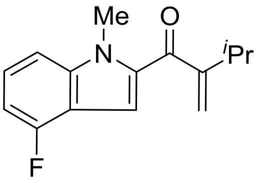

Colorless solid, mp: 68–69 °C. R_f = 0.41 (PE/EA = 40:1, v/v), 87% yield. ^1H NMR (400 MHz, $CDCl_3$) δ 7.32–7.25 (m, 1H), 7.17–7.14 (m, 2H), 6.80 (dd, *J* = 10.0, 7.8 Hz, 1H), 5.73 (s, 1H), 5.69 (d, *J* = 0.9 Hz, 1H), 4.05 (s, 3H), 3.04 (hept, *J* = 6.8 Hz, 1H), 1.15 (d, *J* = 6.9 Hz, 6H); ^{13}C NMR (101 MHz, $CDCl_3$) δ 191.0, 157.4 (d, *J* = 252.1 Hz), 155.5, 142.5 (d, *J* = 10.0 Hz), 135.7, 126.2 (d, *J* = 8.0 Hz), 120.5, 115.4 (d, *J* = 22.8 Hz), 109.6, 106.3 (d, *J* = 4.0 Hz), 104.9 (d, *J* = 18.6 Hz), 32.4, 29.8, 21.3. IR (film): $\tilde{\nu}$ 3090, 2957, 2871, 1633, 1622, 1573, 1513, 1468, 1230, 1136, 974, 763, 723, 708 cm^{-1}. ESI-HRMS calcd for $[C_{15}H_{17}FNO, M + H]^+$: 246.1289, Found 246.1289.

3. Typical Procedure for Nazarov Cyclization of Indole Enones

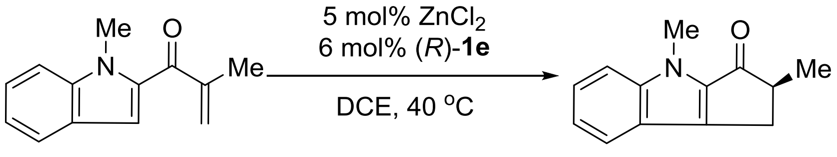

The $ZnCl_2$ (1.4 mg, 0.01 mmol, 5 mol%) and (*R*)-**1e** (8.6 mg, 0.012 mmol, 6 mol%) were introduced into an oven-dried Schlenk tube in an argon-filled glovebox. After 2 mL DCE was injected into the Schlenk tube, the mixture was stirred at 40 °C. A solution of **2a** (40 mg, 0.2 mmol) in 1 mL DCE was introduced into the mixture in one portion. The TLC showed that the reaction finished in 48 hours. Then the reaction mixture was concentrated and purified by a flash chromatography on silica gel (PE/EA = 10:1, v/v) to give **3a** as a colorless oil.

4. Analytical Data of Cyclization Products

(+)-2,4-dimethyl-1,4-dihydrocyclopenta[b]indol-3(2H)-one (3a)

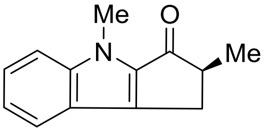

Colorless oil, TLC R_f = 0.45 (PE/EA = 10:1, v/v), 98% yield, 91:9 er. HPLC condition: Chiralcel OD-3 column (25 cm × 0.46 cm ID), hexane/2-propanol = 90:10, 1.0 mL/min, 220 nm UV detecter, t_R = 6.62 min (major) and t_R = 7.63 min (minor). $[\alpha]_D^{27}$ +3.4 (*c* 1.0, $CHCl_3$). ^1H NMR (400 MHz, $CDCl_3$) δ 7.67 (d, *J* = 8.0 Hz, 1H), 7.41 (t, *J* = 7.6 Hz, 1H), 7.35 (d, *J* = 8.3 Hz, 1H), 7.16 (t, *J* = 7.3 Hz, 1H), 3.91 (s, 3H), 3.32 (dd, *J* = 16.7, 6.3 Hz, 1H), 3.06–3.01 (m, 1H), 2.64 (d, *J* = 16.7 Hz, 1H), 1.37 (d, *J* = 7.4 Hz, 3H); ^{13}C NMR (101 MHz, $CDCl_3$) δ 198.2, 145.5, 143.2, 138.4, 127.1, 123.5, 122.1, 120.5, 111.3, 47.8, 30.4, 29.0, 17.6. IR (film): $\tilde{\nu}$ 3055, 2961, 2928, 2869, 2851, 1678, 1500, 961, 741 cm^{-1}. ESI-HRMS calcd for $[C_{13}H_{14}NO, M + H]^+$: 200.1070, Found 200.1071.

(+)-4-isopropyl-2-methyl-1,4-dihydrocyclopenta[b]indol-3(2H)-one (3b)

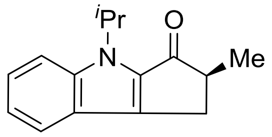

White solid, mp: 77–79 °C. TLC R_f = 0.62 (PE/EA = 10:1, v/v), 98% yield, 90:10 er. HPLC condition: Chiralcel OD-3 column (25 cm × 0.46 cm ID), hexane/2-propanol = 90:10, 1.0 mL/min, 220 nm UV detecter, t_R = 5.16 min (major) and t_R = 5.76 min (minor). $[\alpha]_D^{27}$ +10.0 (*c* 1.0, $CHCl_3$). ^{1}H NMR (400 MHz, $CDCl_3$) δ 7.67 (d, *J* = 8.0 Hz, 1H), 7.46 (d, *J* = 8.6 Hz, 1H), 7.37 (t, *J* = 7.7 Hz, 1H), 7.13 (t, *J* = 7.5 Hz, 1H), 4.89 (hept, *J* = 6.8 Hz, 1H), 3.33 (dd, *J* = 16.7, 6.5 Hz, 1H), 3.08–3.01 (m, 1H), 2.66 (dd, *J* = 16.7, 2.3 Hz, 1H), 1.61 (dd, *J* = 6.8, 1.6 Hz, 6H), 1.38 (d, *J* = 7.5 Hz, 3H); ^{13}C NMR (101 MHz, $CDCl_3$) δ 196.6, 144.6, 143.5, 137.4, 126.4, 122.8, 121.6, 119.7, 111.8, 47.3, 28.6, 22.1, 22.0, 17.3. IR (film): $\tilde{\nu}$ 2967, 2927, 2870, 1681, 1462, 1343, 968, 741 cm^{-1}. ESI-HRMS calcd for $[C_{15}H_{18}NO, M + H]^+$: 228.1383, Found 228.1382.

(+)-4-allyl-2-methyl-1,4-dihydrocyclopenta[b]indol-3(2H)-one (3c)

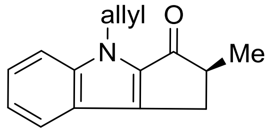

Colorless oil, TLC R_f = 0.52 (PE/EA = 10:1, v/v), 93% yield, 89:11 er. HPLC condition: Chiralcel OD-3 column (25 cm × 0.46 cm ID), hexane/2-propanol = 90:10, 1.0 mL/min, 220 nm UV detecter, t_R = 5.82 min (major) and t_R = 6.95 min (minor). $[\alpha]_D^{27}$ +2.6 (*c* 1.0, $CHCl_3$). ^{1}H NMR (400 MHz, $CDCl_3$) δ 7.68 (d, *J* = 8.0 Hz, 1H), 7.40–7.34 (m, 2H), 7.20–7.14 (m, 1H), 5.97 (ddd, *J* = 22.3, 10.5, 5.4 Hz, 1H), 5.14 (dd, *J* = 10.0, 0.8 Hz, 1H), 5.06 (dd, *J* = 17.1, 0.9 Hz, 1H), 5.01–4.90 (m, 2H), 3.32 (dd, *J* = 16.7, 6.4 Hz, 1H), 3.08–2.99 (m, 1H), 2.65 (dd, *J* = 16.8, 2.1 Hz, 1H), 1.37 (d, *J* = 7.5 Hz, 3H); ^{13}C NMR (101 MHz, $CDCl_3$) δ 197.4, 144.3, 143.2, 137.5, 133.2, 126.6, 123.2, 121.7, 120.2, 116.9, 111.6, 47.3, 46.0, 28.6, 17.1. IR (film): $\tilde{\nu}$ 3055, 2962, 2926, 2869, 2852, 1683, 1645, 1614, 1546, 947, 923, 895, 844, 742 cm^{-1}. ESI-HRMS calcd for $[C_{15}H_{16}NO, M + H]^+$: 226.1226, Found 226.1228.

(–)-4-benzyl-2-methyl-1,4-dihydrocyclopenta[b]indol-3(2H)-one (3d)

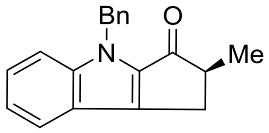

Light yellow solid, mp: 69–72 °C. TLC R_f = 0.52 (PE/EA = 10:1, v/v), 87% yield, 88:12 er. HPLC condition: Chiralpak AD-3 column (25 cm × 0.46 cm ID), hexane/2-propanol = 90:10, 1.0 mL/min, 220 nm UV detecter, t_R = 6.33 min (minor) and t_R = 7.35 min (major). $[\alpha]_D^{27}$ –1.2 (*c* 1.0, $CHCl_3$). ^1H NMR (400 MHz, $CDCl_3$) δ 7.69 (d, *J* = 8.0 Hz, 1H), 7.35–7.32 (m, 2H), 7.25–7.22 (m, 5H), 7.17–7.14 (m, 1H), 5.56 (d, *J* = 15.7 Hz, 1H), 5.51 (d, *J* = 15.7 Hz, 1H), 3.35 (dd, *J* = 16.8, 6.3 Hz, 1H), 3.12–3.03 (m, 1H), 2.68 (d, *J* = 16.8 Hz, 1H), 1.39 (d, *J* = 7.4 Hz, 3H); ^{13}C NMR (101 MHz, $CDCl_3$) δ 197.6, 144.4, 143.5, 137.7, 137.4, 128.6, 127.5, 127.3, 126.8, 123.4, 121.8, 120.3, 111.7, 47.5, 47.4, 28.7, 17.1. IR (film): ṽ 3087, 3061, 3031, 2962, 2925, 2869, 2850, 1682, 1614, 1563, 913, 742, 703 cm^{-1}. ESI-HRMS calcd for $[C_{19}H_{18}NO, M + H]^+$: 276.1383, Found 276.1384.

(+)-2-methyl-4-phenyl-1,4-dihydrocyclopenta[b]indol-3(2H)-one (3e)

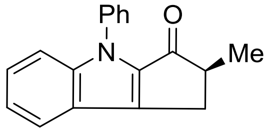

Colorless solid, mp: 94–96 °C. TLC R_f = 0.48 (PE/EA = 10:1, v/v), 90% yield, 92:8 er. HPLC condition: Chiralcel OD-3 column (25 cm × 0.46 cm ID), hexane/2-propanol = 90:10, 1.0 mL/min, 220 nm UV detecter, t_R = 5.82 min (major) and t_R = 6.90 min (minor). $[\alpha]_D^{27}$ +44.7 (*c* 1.0, $CHCl_3$). ^1H NMR (400 MHz, $CDCl_3$) δ 7.74 (d, *J* = 8.0 Hz, 1H), 7.58–7.47 (m, 5H), 7.41–7.34 (m, 2H), 7.23 (t, *J* = 7.4 Hz, 1H), 3.39 (dd, *J* = 16.9, 6.5 Hz, 1H), 3.13–3.03 (m, 1H), 2.72 (dd, *J* = 16.9, 2.0 Hz, 1H), 1.38 (d, *J* = 7.5 Hz, 3H); ^{13}C NMR (101 MHz, $CDCl_3$) δ 196.0, 145.7, 144.4, 137.3, 136.3, 129.1, 127.3, 127.1, 125.7, 123.6, 121.6, 121.1, 112.3, 47.4, 28.4, 17.3. IR (film): ṽ 3049, 2961, 2924, 2901, 2869, 2839, 1686, 1595, 1542, 1500, 892, 759, 747 cm^{-1}. ESI-HRMS calcd for $[C_{18}H_{16}NO, M + H]^+$: 226.1226, Found 262.1230.

(+)-4-(4-methoxyphenyl)-2-methyl-1,4-dihydrocyclopenta[b]indol-3(2H)-one (3f)

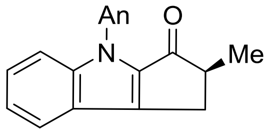

Colorless solid, mp: 97–99 °C. TLC R_f = 0.31 (PE/EA = 10:1,

v/v), 95% yield, 92:8 er. HPLC condition: Chiralcel OD-3 column (25 cm × 0.46 cm ID), hexane/2-propanol = 90:10, 1.0 mL/min, 220 nm UV detecter, t_R = 7.86 min (major) and t_R = 9.24 min (minor). $[\alpha]_D^{27}$ +35.0 (*c* 1.0, $CHCl_3$). ^{1}H NMR (400 MHz, $CDCl_3$) δ 7.77 (d, *J* = 8.0 Hz, 1H), 7.51 (d, *J* = 8.5 Hz, 1H), 7.47–7.44 (m, 2H), 7.43–7.39 (m, 1H),7.25 (t, *J* = 7.4 Hz, 1H), 7.09–7.04 (m, 2H), 3.90 (s, 3H), 3.42 (dd, *J* = 16.9, 6.5 Hz, 1H), 3.15–3.07 (m, 1H), 2.75 (dd, *J* = 16.9, 2.2 Hz, 1H), 1.42 (d, *J* = 7.5 Hz, 3H); ^{13}C NMR (101 MHz, $CDCl_3$) δ 196.1, 158.5, 145.0, 144.7, 137.5, 129.2, 127.2, 127.0, 123.3, 121.5, 120.9, 114.3, 112.2, 55.4, 47.3, 28.3, 17.2. IR (film): $\tilde{\nu}$ 2958, 2925, 2869, 2852, 1687, 1543, 1514, 1457, 1249, 743 cm^{-1}. ESI-HRMS calcd for $[C_{19}H_{18}NO_2, M + H]^+$: 292.1332, Found 292.1337.

(+)-2-methyl-4-(4-(trifluoromethyl)phenyl)-1,4-dihydrocyclopenta[b]indol-3(2H)-one (3g)

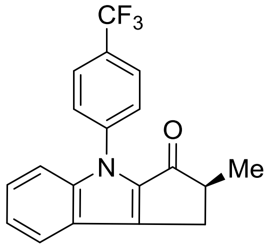

Colorless solid, mp: 136–138 °C. TLC R_f = 0.36 (PE/EA = 15:1, v/v), 76% yield, 91:9 er. HPLC condition: Chiralcel OD-3 column (25 cm × 0.46 cm ID), hexane/2-propanol = 97:3, 1.0 mL/min, 220 nm UV detecter, t_R = 6.21 min (major) and t_R = 6.79 min (minor). $[\alpha]_D^{27}$ +42.1 (*c* 1.0, $CHCl_3$). ^{1}H NMR (400 MHz, $CDCl_3$) δ 7.76 (d, *J* = 8.1 Hz, 3H), 7.66 (d, *J* = 8.3 Hz, 2H), 7.57 (d, *J* = 8.5 Hz, 1H), 7.42 (t, *J* = 7.8 Hz, 1H), 7.27 (t, *J* = 7.5 Hz, 1H), 3.40 (dd, *J* = 17.0, 6.4 Hz, 1H), 3.13–3.05 (m, 1H), 2.73 (dd, *J* = 17.0, 1.9 Hz, 1H), 1.39 (d, *J* = 7.5 Hz, 3H); ^{13}C NMR (101 MHz, $CDCl_3$) δ 196.0, 147.0, 144.0, 139.4, 137.0, 128.7 (q, *J* = 32.9 Hz), 127.9, 126.3 (q, *J* =3.7 Hz), 125.6, 124.0, 123.9 (q, *J* =273.2 Hz), 121.9, 121.7, 112.0, 47.4, 28.3, 17.2. IR (film): $\tilde{\nu}$ 3057, 2955, 2923, 2870, 2850, 1684, 1455, 1335, 1114, 849, 762, 747 cm^{-1}. ESI-HRMS calcd for $[C_{19}H_{15}F_3NO, M + H]^+$: 330.1100, Found 330.1102.

(+)-2-methyl-4-(naphthalen-2-yl)-1,4-dihydrocyclopenta[b]indol-3(2H)-one (3h)

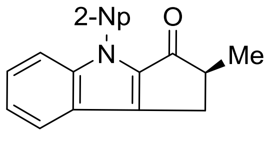

Colorless solid, mp: 119–120 °C. TLC R_f = 0.36 (PE/EA = 10:1,

v/v), 92% yield, 92:8 er. HPLC condition: Chiralpak AD-3 column (25 cm × 0.46 cm ID), hexane/2-propanol = 90:10, 1.0 mL/min, 220 nm UV detecter, t_R = 7.53 min (major) and t_R = 8.93 min (minor). $[\alpha]_D^{28}$ +49.7 (*c* 1.0, $CHCl_3$). ^1H NMR (400 MHz, $CDCl_3$) δ 7.98–7.92 (m, 2H), 7.86 (dd, *J* = 9.2, 6.6 Hz, 2H), 7.75 (d, *J* = 8.0 Hz, 1H), 7.64–7.56 (m, 2H), 7.53–7.46 (m, 2H), 7.38 (td, *J* = 7.2, 0.8 Hz, 1H), 7.23 (t, *J* = 7.6 Hz, 1H), 3.39 (dd, *J* = 16.9, 6.5 Hz, 1H), 3.08 (pd, *J* = 7.4, 2.2 Hz, 1H), 2.72 (dd, *J* = 16.9, 2.2 Hz, 1H), 1.39 (d, *J* = 7.5 Hz, 3H); ^{13}C NMR (101 MHz, $CDCl_3$) δ 196.0, 145.8, 144.5, 137.5, 133.8, 133.4, 132.1, 129.0, 127.9, 127.7, 127.4, 126.6, 126.1, 124.4, 123.6, 123.5, 121.7, 121.2, 112.3, 47.4, 28.4, 17.2. IR (film): $\tilde{\nu}$ 3056, 2958, 2925, 2868, 2851, 1684, 1512, 1476, 1451, 969, 743 cm^{-1}. ESI-HRMS calcd for $[C_{22}H_{18}NO, M + H]^+$: 312.1383, Found 312.1385.

(+)-2-ethyl-4-methyl-1,4-dihydrocyclopenta[b]indol-3(2H)-one (3i)

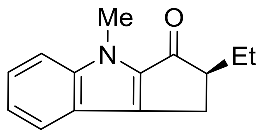

Colorless solid, mp: 83–84 °C. TLC R_f = 0.42 (PE/EA = 10:1, v/v), 93% yield, 89:11 er. HPLC condition: Chiralcel OD-3 column (25 cm × 0.46 cm ID), hexane/2-propanol = 90:10, 1.0 mL/min, 220 nm UV detecter, t_R = 5.95 min (major) and t_R = 6.70 min (minor). $[\alpha]_D^{27}$ +37.6 (*c* 1.0, $CHCl_3$). ^1H NMR (400 MHz, $CDCl_3$) δ 7.69 (d, *J* = 8.0 Hz, 1H), 7.41 (t, *J* = 8.0 Hz, 1H), 7.35 (d, *J* = 8.3 Hz, 1H), 7.17 (t, *J* = 7.4 Hz, 1H), 3.91 (s, 3H), 3.22 (dd, *J* = 16.8, 6.3 Hz, 1H), 2.97–2.89 (m, 1H), 2.73 (dd, *J* = 16.8, 1.5 Hz, 1H), 2.08–1.95 (m, 1H), 1.67–1.55 (m, 1H), 1.03 (t, *J* = 7.4 Hz, 3H); ^{13}C NMR (101 MHz, $CDCl_3$) δ 197.2, 144.9, 143.2, 138.6, 126.7, 123.0, 121.7, 120.1, 110.9, 54.1, 30.0, 25.9, 24.9, 11.4. IR (film): ṽ 3051, 2959, 2932, 2917, 2870, 2847, 1678, 1614, 1560, 1488, 902, 756, 746 cm^{-1}. ESI-HRMS calcd for $[C_{14}H_{16}NO, M + H]^+$: 214.1226, Found 214.1227.

(+)-2-isopropyl-4-methyl-1,4-dihydrocyclopenta[b]indol-3(2H)-one (3j)

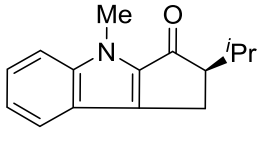

Light yellow oil. TLC R_f = 0.53 (PE/EA = 10:1, v/v), 96% yield, 95:5 er. HPLC condition: Chiralpak AD-3 column (25 cm ×

0.46 cm ID), hexane/2-propanol = 97:3, 1.0 mL/min, 220 nm UV detecter, t_R = 6.08 min (minor) and t_R = 6.55 min (major). $[\alpha]_D^{28}$ +51.1 (*c* 1.0, $CHCl_3$). ^1H NMR (400 MHz, $CDCl_3$) δ 7.70 (d, *J* = 8.0 Hz, 1H), 7.41 (td, *J* = 8.4, 1.2 Hz, 1H), 7.36 (d, *J* = 8.4 Hz, 1H), 7.17 (td, *J* = 7.6, 0.8 Hz, 1H), 3.91 (s, 3H), 3.08–2.97 (m, 2H), 2.83 (d, *J* = 15.3 Hz, 1H), 2.50–2.42 (m, 1H), 1.08 (d, *J* = 6.9 Hz, 3H), 0.83 (d, *J* = 6.8 Hz, 3H); ^{13}C NMR (101 MHz, $CDCl_3$) δ 197.0, 144.9, 143.6, 139.3, 126.6, 123.0, 121.7, 120.1, 110.9, 58.4, 30.1, 29.0, 21.5, 20.8, 16.8. IR (film): $\tilde{\nu}$ 3055, 2955, 2927, 2869, 1672, 1615, 1488, 1202, 1050, 820, 739 cm^{-1}. ESI-HRMS calcd for $[C_{15}H_{18}NO, M + H]^+$: 228.1383, Found 228.1386.

(+)-4-methyl-2-phenyl-1,4-dihydrocyclopenta[b]indol-3(2H)-one (3k)

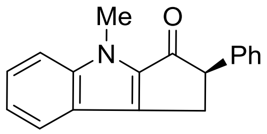

Light yellow solid, mp: 126–128 °C. TLC R_f = 0.41 (PE/EA = 6:1, v/v), 95% yield, 86:14 er. HPLC condition: Chiralpak AD-3 column (25 cm × 0.46 cm ID), hexane/2-propanol = 80:20, 1.0 mL/min, 220 nm UV detecter, t_R = 5.88 min (minor) and t_R = 7.93 min (major). $[\alpha]_D^{28}$ +177.4 (*c* 1.0, $CHCl_3$). ^1H NMR (400 MHz, $CDCl_3$) δ 7.72 (d, *J* = 8.0 Hz, 1H), 7.45 (t, *J* = 7.6 Hz, 1H), 7.39 (d, *J* = 8.3 Hz, 1H), 7.35–7.28 (m, 2H), 7.26–7.24 (m, 2H), 7.22–7.18 (m, 2H), 4.15 (d, *J* = 4.9 Hz, 1H), 3.91 (s, 3H), 3.59 (dd, *J* = 17.1, 6.8 Hz, 1H), 3.14 (d, *J* = 17.1 Hz, 1H); ^{13}C NMR (101 MHz, $CDCl_3$) δ 194.1, 145.2, 143.6, 140.2, 138.0, 128.7, 127.7, 127.0, 127.0, 122.9, 121.9, 120.3, 111.0, 58.5, 30.1, 29.7. IR (film): $\tilde{\nu}$ 3058, 3026, 2954, 2923, 2851, 1682, 1489, 1214, 744, 699 cm^{-1}. ESI-HRMS calcd for $[C_{18}H_{16}NO, M + H]^+$: 262.1226, Found 262.1228.

(+)-2-(4-methoxyphenyl)-4-methyl-1,4-dihydrocyclopenta[b]indol-3(2H)-one (3l)

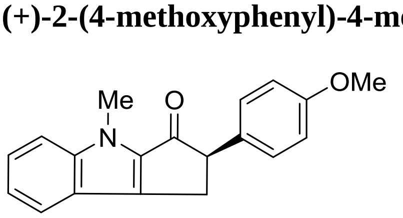

Light yellow solid, mp: 114–115 °C. TLC R_f = 0.44 (PE/EA = 6:1, v/v), 91% yield, 85:15 er. HPLC condition: Chiralcel OD-3 column (25 cm × 0.46 cm ID), hexane/2-propanol = 70:30, 1.0 mL/min, 220 nm UV detecter, t_R = 11.05 min (minor) and t_R = 14.68 min (major). $[\alpha]_D^{27}$ +210.1 (*c* 1.0, $CHCl_3$). ^1H NMR (400 MHz, $CDCl_3$) δ 7.72 (d, *J* = 8.0 Hz, 1H), 7.44 (t, *J* = 8.0 Hz, 1H), 7.38 (d, *J* = 8.4 Hz, 1H),

7.23–7.12 (m, 3H), 6.86 (d, J = 8.5 Hz, 2H), 4.10 (dd, J = 6.8, 2.0 Hz, 1H), 3.90 (s, 3H), 3.77 (s, 3H), 3.57 (dd, J = 17.1, 6.8 Hz, 1H), 3.09 (dd, J = 17.1, 1.7 Hz, 1H); ^{13}C NMR (101 MHz, $CDCl_3$) δ 194.5, 158.5, 145.2, 143.4, 137.9, 132.2, 128.7, 126.9, 122.9, 121.8, 120.3, 114.1, 111.0, 57.8, 55.2, 30.0, 29.8. IR (film): $\tilde{\nu}$ 3056, 2954, 2925, 2870, 2852, 1683, 1512, 1248, 891, 837, 746 cm^{-1}. ESI-HRMS calcd for [$C_{19}H_{18}NO_2$, M + H]$^+$: 292.1332, Found 292.1337.

(+)-2-(4-fluorophenyl)-4-methyl-1,4-dihydrocyclopenta[b]indol-3(2H)-one (3m)

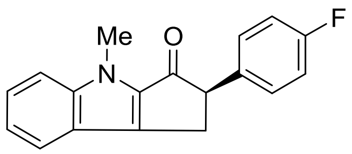

Light yellow solid, mp: 90–92 °C. TLC R_f = 0.39 (PE/EA = 10:1, v/v), 84% yield, 89:11 er. HPLC condition: Chiralcel OD-3 column (25 cm × 0.46 cm ID), hexane/2-propanol = 80:20, 1.0 mL/min, 220 nm UV detecter, t_R = 11.09 min (major) and t_R = 12.20 min (minor). $[\alpha]_D^{28}$ +198.0 (c 1.0, $CHCl_3$). ^{1}H NMR (400 MHz, $CDCl_3$) δ 7.72 (d, J = 8.1 Hz, 1H), 7.46 (t, J = 7.6 Hz, 1H), 7.40 (d, J = 8.4 Hz, 1H), 7.24–7.17 (m, 3H), 7.00 (t, J = 8.7 Hz, 2H), 4.14 (dd, J = 6.8, 2.2 Hz, 1H), 3.92 (s, 3H), 3.60 (dd, J = 17.1, 6.9 Hz, 1H), 3.10 (dd, J = 17.1, 2.2 Hz, 1H); ^{13}C NMR (101 MHz, $CDCl_3$) δ 193.9, 161.9 (d, J = 246.1 Hz), 145.3, 143.4, 137.8, 135.9 (d, J = 3.1 Hz), 129.3 (d, J = 8.0 Hz), 127.1, 122.9, 121.9, 120.4, 115.6 (d, J = 21.3 Hz), 111.1, 57.7, 30.1, 29.8. IR (film): ṽ 3066, 3043, 3010, 2949, 2920, 2900, 2858, 1696, 1549, 1511, 1220, 750, 737 cm^{-1}. ESI-HRMS calcd for [$C_{18}H_{15}FNO$, M + H]$^+$: 280.1132, Found 280.1131.

(+)-2-(4-chlorophenyl)-4-methyl-1,4-dihydrocyclopenta[b]indol-3(2H)-one (3n)

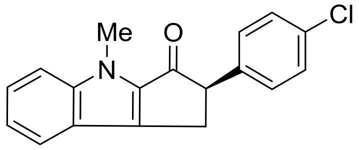

Light yellow solid, mp: 142–143 °C. TLC R_f = 0.39 (PE/EA = 10:1, v/v), 78% yield, 89:11 er. HPLC condition: Chiralcel OD-3 column (25 cm × 0.46 cm ID), hexane/2-propanol = 80:20, 1.0 mL/min, 220 nm UV detecter, t_R = 11.11 min (minor) and t_R = 12.35 min (major). $[\alpha]_D^{28}$ +217.5 (c 1.0, $CHCl_3$). ^{1}H NMR (400 MHz, $CDCl_3$) δ 7.73 (d, J = 8.0 Hz, 1H), 7.46 (t, J = 7.6 Hz, 1H), 7.40 (d, J = 8.4 Hz, 1H), 7.29 (d, J = 8.3 Hz, 2H), 7.25–7.20 (m, 1H), 7.19–7.16 (m, 2H), 4.14 (dd, J = 6.8 Hz,

1.6 Hz, 1H), 3.92 (s, 3H), 3.61 (dd, *J* = 17.1, 6.8 Hz, 1H), 3.10 (dd, *J* = 17.1 Hz, 1.6 Hz, 1H); ^{13}C NMR (101 MHz, $CDCl_3$) δ 193.6, 145.3, 143.5, 138.6, 137.7, 132.8, 129.1, 128.9, 127.2, 122.8, 121.9, 120.4, 111.1, 57.8, 30.1, 29.7. IR (film): ṽ 3051, 3025, 2965, 2923, 2849, 1678, 1615, 1491, 752 cm^{-1}. ESI-HRMS calcd for [$C_{18}H_{15}ClNO$, M + H]$^+$: 296.0837, Found 296.0831.

(+)-2-(benzo[d][1,3]dioxol-5-yl)-4-methyl-1,4-dihydrocyclopenta[b]indol-3(2H)-one (3o)

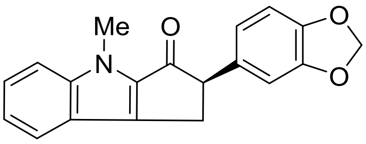

Light yellow solid, mp: 181–182 °C. TLC R_f = 0.39 (PE/EA = 10:1, v/v), 95% yield, 92:8 er. HPLC condition: Chiralcel OD-3 column (25 cm × 0.46 cm ID), hexane/2-propanol = 70:30, 1.0 mL/min, 220 nm UV detecter, t_R = 14.36 min (minor) and t_R = 18.86 min (major). $[\alpha]_D^{27}$ +241.1 (*c* 1.0, $CHCl_3$). ^{1}H NMR (400 MHz, $CDCl_3$) δ 7.72 (d, *J* = 8.0 Hz, 1H), 7.45 (t, *J* = 8.0 Hz, 1H), 7.39 (d, *J* = 8.4 Hz, 1H), 7.21 (dd, *J* = 15.2, 7.7 Hz, 1H), 6.74 (dd, *J* = 18.2, 8.0 Hz, 2H), 6.67 (s, 1H), 5.90 (d, *J* = 2.6 Hz, 2H), 4.06 (d, *J* = 5.2 Hz, 1H), 3.91 (s, 3H), 3.57 (dd, *J* = 17.1, 6.8 Hz, 1H), 3.07 (d, *J* = 17.2 Hz, 1H); ^{13}C NMR (101 MHz, $CDCl_3$) δ 194.2, 147.9, 146.5, 145.2, 143.5, 137.8, 133.9, 127.0, 122.8, 121.8, 121.1, 120.3, 111.0, 108.4, 107.8, 100.9, 58.2, 30.1, 29.9. IR (film): $\tilde{\nu}$ 2954, 2923, 2851, 1683, 1489, 1243, 1038, 745 cm^{-1}. ESI-HRMS calcd for [$C_{19}H_{16}NO_3$, M + H]$^+$: 306.1125, Found 306.1132.

(+)-2-isopropyl-4,7-dimethyl-1,4-dihydrocyclopenta[b]indol-3(2H)-one (3p)

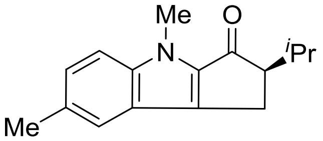

Light yellow liquid. TLC R_f = 0.44 (PE/EA = 15:1, v/v), 83% yield, 92:8 er. HPLC condition: Chiralpak AD-3 column (25 cm × 0.46 cm ID), hexane/2-propanol = 95:5, 1.0 mL/min, 220 nm UV detecter, t_R = 5.41 min (minor) and t_R = 6.85 min (major). $[\alpha]_D^{27}$ +37.0 (*c* 1.0, $CHCl_3$). ^{1}H NMR (400 MHz, $CDCl_3$) δ 7.47 (s, 1H), 7.26–7.22 (m, 2H), 3.88 (s, 3H), 3.02–2.95 (m, 2H), 2.79 (dd, *J* = 19.3, 5.2 Hz, 1H), 2.46–2.43 (m, 4H), 1.07 (d, *J* = 6.9 Hz, 3H), 0.81 (d, *J* = 6.8 Hz, 3H); ^{13}C NMR (101 MHz, $CDCl_3$) δ

196.9, 143.4, 143.1, 139.3, 129.4, 128.5, 123.1, 121.0, 110.5, 58.4, 30.0, 29.0, 21.4, 21.3, 20.8, 16.7. IR (film): ṽ 3023, 2956, 2925, 2869, 1678, 1625, 1501, 1218, 794, 761 cm^{-1}. ESI-HRMS calcd for [$C_{16}H_{20}NO$, M + H]$^+$: 242.1539, Found 242.1542.

(+)-7-fluoro-2-isopropyl-4-methyl-1,4-dihydrocyclopenta[b]indol-3(2H)-one (3q)

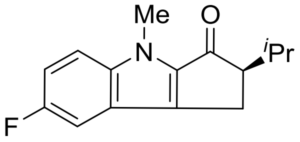

Colorless solid, mp: 110–112 °C. TLC R_f = 0.44 (PE/EA = 8:1, v/v), 94% yield, 95:5 er. HPLC condition: Chiralpak AD-3 column (25 cm × 0.46 cm ID), hexane/2-propanol = 97:3, 1.0 mL/min, 220 nm UV detecter, t_R = 6.83 min (minor) and t_R = 7.29 min (major). $[\alpha]_D^{29}$ +53.1 (*c* 1.0, $CHCl_3$). ^{1}H NMR (400 MHz, $CDCl_3$) δ 7.35–7.26 (m, 2H), 7.16 (td, *J* = 9.1, 2.5 Hz, 1H), 3.90 (s, 3H), 3.04–2.96 (m, 2H), 2.83–2.75 (m, 1H), 2.49–2.41 (m, 1H), 1.08 (d, *J* = 6.9 Hz, 3H), 0.83 (d, *J* = 6.8 Hz, 3H); ^{13}C NMR (101 MHz, $CDCl_3$) δ 197.1, 157.7 (d, *J* = 238.3 Hz), 142.6 (d, *J* = 5.5 Hz), 141.4, 140.5, 122.8 (d, *J* = 9.8 Hz), 115.4 (d, *J* = 27.1 Hz), 111.8 (d, *J* = 9.5 Hz), 106.1 (d, *J* = 23.2 Hz), 58.4, 30.2, 29.0, 21.4, 20.7, 16.8. IR (film): ṽ 2957, 2926, 2871, 1680, 1500, 1161, 943, 798, 763, 752 cm^{-1}. ESI-HRMS calcd for [$C_{15}H_{17}FNO$, M + H]$^+$: 246.1289, Found 246.1293.

(*R*)-(+)-7-chloro-2-isopropyl-4-methyl-1,4-dihydrocyclopenta[b]indol-3(2H)-one (3r)

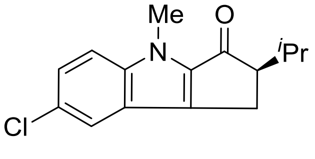

Colorless solid, mp: 110–111 °C. TLC R_f = 0.44 (PE/EA = 10:1, v/v), 79% yield, 92:8 er. HPLC condition: Chiralpak AD-3 column (25 cm × 0.46 cm ID), hexane/2-propanol = 97:3, 1.0 mL/min, 220 nm UV detecter, t_R = 7.06 min (minor) and t_R = 7.98 min (major). $[\alpha]_D^{28}$ +30.8 (*c* 1.0, $CHCl_3$). ^{1}H NMR (400 MHz, $CDCl_3$) δ 7.66 (d, *J* = 1.7 Hz, 1H), 7.33 (dd, *J* = 8.9, 1.9 Hz, 1H), 7.27 (d, *J* = 8.9 Hz, 1H), 3.89 (s, 3H), 3.02–2.95 (m, 2H), 2.82–2.75 (m, 1H), 2.50–2.39 (m, 1H), 1.07 (d, *J* = 6.9 Hz, 3H), 0.82 (d, *J* = 6.8 Hz, 3H); ^{13}C NMR (101 MHz, $CDCl_3$) δ 197.0, 143.0, 142.4, 140.2, 126.8, 125.8, 123.7, 120.9, 112.0, 58.4, 30.2, 29.0, 21.4, 20.7, 16.8. IR (film): ṽ 3059, 2956,

2926, 2870, 1679, 1490, 1467, 1204, 1072, 922, 797, 761, 695, 682 cm^{-1}. ESI-HRMS calcd for [$C_{15}H_{17}ClNO$, M + H]$^+$: 262.0993, Found 262.0998.

(+)-8-fluoro-2-isopropyl-4-methyl-1,4-dihydrocyclopenta[b]indol-3(2H)-one (3s)

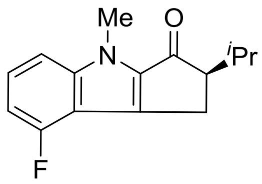

Colorless solid, mp: 38–39 °C. TLC R_f = 0.50 (PE/EA = 10:1, v/v), 71% yield, 92:8 er. HPLC condition: Chiralcel OD-3 column (25 cm × 0.46 cm ID), hexane/2-propanol = 90:10, 1.0 mL/min, 220 nm UV detecter, t_R = 5.30 min (major) and t_R = 5.84 min (minor). $[\alpha]_D^{29}$ +49.3 (*c* 1.0, $CHCl_3$). ^{1}H NMR (400 MHz, $CDCl_3$) δ 7.34–7.26 (m, 1H), 7.12 (d, *J* = 8.4 Hz, 1H), 6.81 (dd, *J* = 10.0, 7.9 Hz, 1H), 3.91 (s, 3H), 3.14 (dd, *J* = 17.0, 6.2 Hz, 1H), 3.01–2.97 (m, 1H), 2.94 (dd, *J* = 17.1, 2.2 Hz, 1H), 2.51–2.39 (m, 1H), 1.08 (d, *J* = 6.9 Hz, 3H), 0.84 (d, *J* = 6.8 Hz, 3H); ^{13}C NMR (101 MHz, $CDCl_3$) δ 196.9, 157.8 (d, *J* = 252.4 Hz), 146.7 (d, *J* = 10.5 Hz), 140.9, 139.2, 127.3 (d, *J* = 8.1 Hz), 112.9 (d, *J* = 23.0 Hz), 106.8 (d, *J* = 4.0 Hz), 104.9 (d, *J* = 18.5 Hz), 58.6, 30.4, 29.0, 22.3, 20.7, 16.8. IR (film): $\tilde{\nu}$ 2956, 2926, 2870, 1684, 1631, 1463, 1242, 989, 975, 778, 731 cm^{-1}. ESI-HRMS calcd for [$C_{15}H_{17}FNO$, M + H]$^+$: 246.1289, Found 246.1290.

5. X-Ray Diffraction Analysis of (*R*)-3r

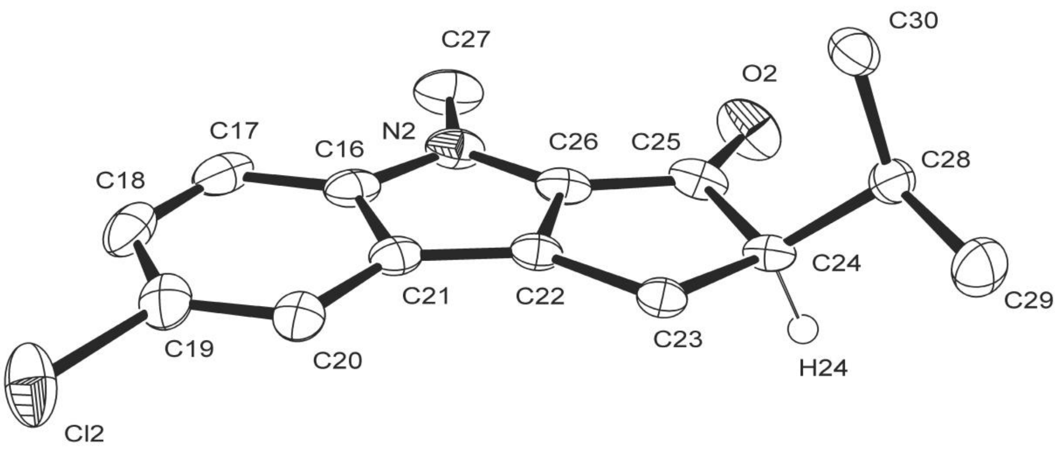

Table S1. Crystal data and structure refinement for (*R*)-3r

Empirical formula	C15 H16 Cl N O
Moiety formula	$C_{15}H_{16}ClNO$
Formula weight	261.74
Temperature	113(2) K
Wavelength	0.71073 A
Crystal system	Orthorhombic
Space group	P2(1)2(1)2(1)
Unit cell dimensions	a = 8.6226(12) A alpha = 90 deg.<br>b = 13.6329(18) A beta = 90 deg.<br>c = 22.798(3) A gamma = 90 deg.
Volume	2680.0(6) A^3
Z	8
Calculated density	1.297 Mg/m^3
Absorption coefficient	0.272 mm^{-1}
F(000)	1104
Crystal size	0.20 x 0.18 x 0.12 mm
Theta range for data collection	3.07 to 27.51°
Limiting indices	-11<=h<=10, -17<=k<=17, -29<=l<=29
Reflections collected / unique	29367 / 6127 [R(int) = 0.0274]

Completeness to theta = 25.242	99.8 %
Absorption correction	Semi-empirical from equivalents
Max. and min. transmission	0.9680 and 0.9475
Refinement method	Full-matrix least-squares on F^2
Data / restraints / parameters	6127 / 0 / 331
Goodness-of-fit on F^2	1.058
Final R indices [I>2sigma(I)]	R_1 = 0.0248, wR_2 = 0.0685
R indices (all data)	R_1 = 0.0272, wR_2 = 0.0696
Absolute structure parameter	0.01(3)
Largest diff. peak and hole	0.211 and -0.219 e.Å^{-3}

6. Transformations of Cyclization Product

A. Gram-scale experiment

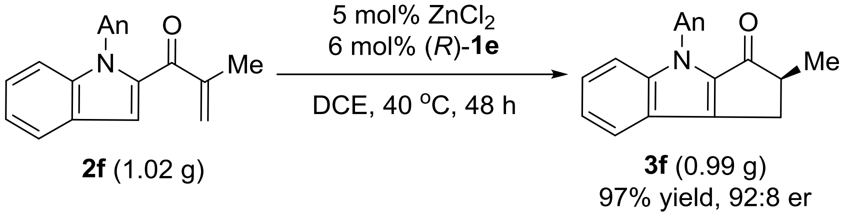

The $ZnCl_2$ (23.9 mg, 0.175 mmol, 5 mol%) and (*R*)-**1e** (151.0 mg, 0.21 mmol, 6 mol%) were introduced into an oven-dried Schlenk flask in an argon-filled glovebox. After 35 mL DCE was injected into the Schlenk tube, the mixture was stirred at 40 ^{o}C. A solution of **2f** (1.020 g, 3.5 mmol) in 17.5 mL DCE was introduced into the mixture. After 48 hours, the reaction mixture was concentrated and purified by a flash chromatography on silica gel (PE/EA = 8:1, v/v) to give **3f** as a colorless solid, 0.989 g, 97% yield, 92:8 er. After recrystallization from a mixed solution (PE/EA = 25:1, v/v) twice, crystal blocks were obtained with 60% yield, 98:2 er.

B. Synthesis of (-)-4-(4-methoxyphenyl)-2-methyl-1,2,3,4-tetrahydrocyclopenta[b] indol-3-ol (4)

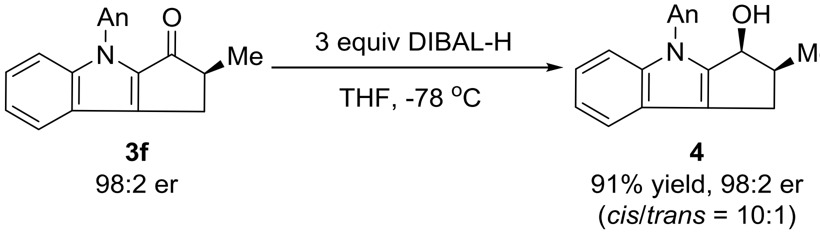

To an oven-dried Schlenk tube equipped with a magnetic stir bar was charged with substrate **3f** (20.4 mg, 0.07 mmol) and dry THF (1.5 mL). After fully cooled to –78 ^{o}C, DIBAL-H (0.21 mL, 1.0 M, 0.21 mmol) was dropped into the mixture during 30 min. The solution was stirred at –78 ^{o}C until the reaction finished (monitored by TLC, about 30 min). 50 μL water was dropped into the Schlenk tube to quench the reaction.

After filtered through a pile of Celite, the solvent was removed by a vacuum pump. ^{1}H NMR of crude product showed that the value of *cis*/*trans* was about 10:1. Pure product was obtained by flash chromatography on basic Al_2O_3 (PE/EA = 4:1 to 2:1, v/v). The major product (*cis* isomer) was white solid, mp: 97–98 °C. TLC R_f = 0.57 (PE/EA = 4:1, v/v), 18.6 mg, 91% yield, 98:2 er. HPLC condition: Chiralcel OD-3 column (25 cm × 0.46 cm ID), hexane/2-propanol = 97:3, 1.0 mL/min, 220 nm UV detecter, t_R = 14.64 min (major) and t_R = 16.07 min (minor). $[\alpha]_D^{23}$ –18.2 (*c* 1.0, $CHCl_3$). ^{1}H NMR (400 MHz, $CDCl_3$) δ 7.55–7.48 (m, 3H), 7.40 (d, *J* = 7.8 Hz, 1H), 7.19–7.11 (m, 2H), 7.03–6.98 (m, 2H), 4.90 (t, *J* = 5.8 Hz, 1H), 3.86 (s, 3H), 3.03–2.93 (m, 2H), 2.64–2.55 (m, 1H), 1.43–1.39 (m, 1H), 1.27 (d, *J* = 6.8 Hz, 3H); ^{13}C NMR (101 MHz, $CDCl_3$) δ 158.1, 146.1, 141.3, 131.4, 126.5, 124.0, 122.3, 122.0, 120.0, 119.7, 114.6, 111.3, 71.1, 55.5, 43.9, 30.9, 14.6. ESI-HRMS calcd for $[C_{19}H_{18}NO, M - OH]^+$: 276.1383, Found 276.1385.

7. NMR Spectra of Intermediates and Indole Enones

2-methyl-1-(1-methyl-1H-indol-2-yl)prop-2-en-1-ol

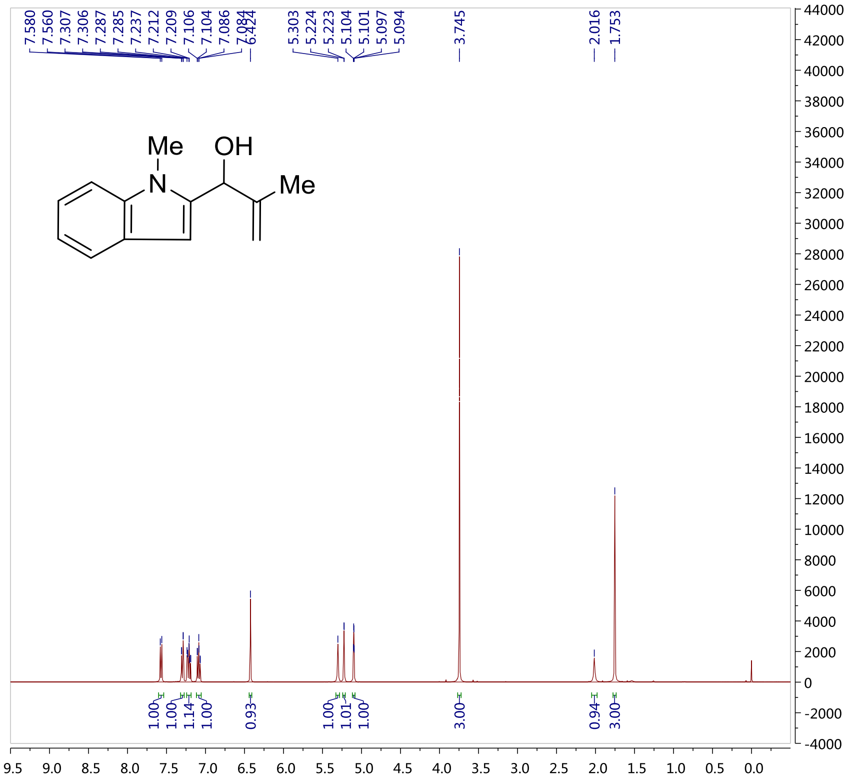

Parameters	
Parameter	Value
Title	wgp-14-178
Comment	test
Origin	Bruker BioSpin GmbH
Owner	nmr
Site	
Spectrometer	spect
Author	
Solvent	CDCl3
Temperature	301.7
Pulse Sequence	zg30
Experiment	1D
Number of Scans	8
Receiver Gain	111
Relaxation Delay	1.0000
Pulse Width	9.8000
Acquisition Time	1.9999
Acquisition Date	2015-03-14T10:31:00
Modification Date	2015-03-15T11:36:00
Spectrometer Frequency	400.13
Spectral Width	8012.8
Lowest Frequency	-1535.4
Nucleus	1H
Acquired Size	16025
Spectral Size	32768

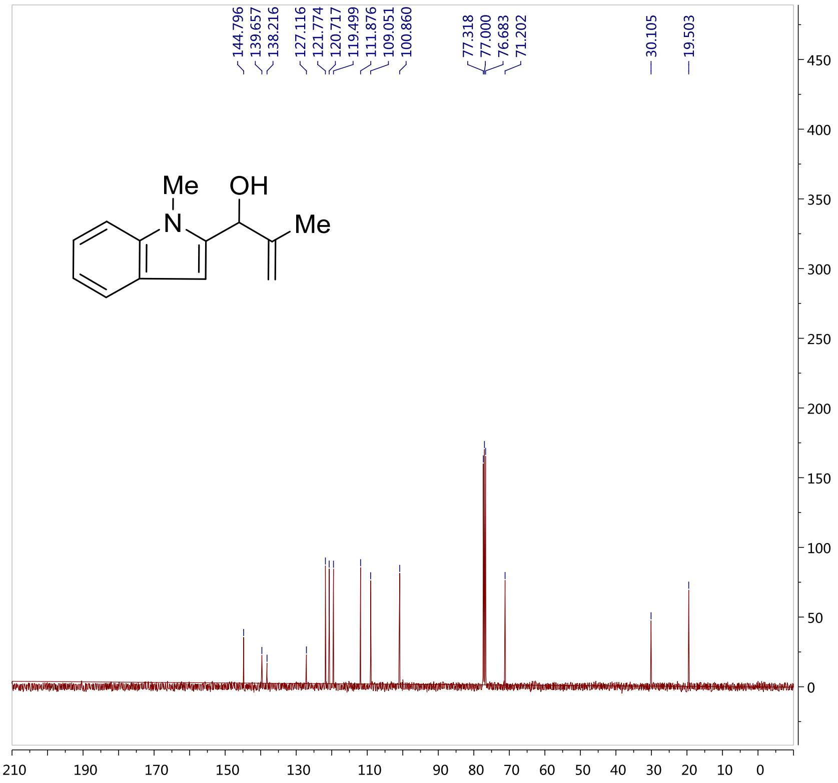

Parameters	
Parameter	Value
Title	wgp-14-178
Comment	test
Origin	Bruker BioSpin GmbH
Owner	nmr
Site	
Spectrometer	spect
Author	
Solvent	CDCl3
Temperature	302.0
Pulse Sequence	zgpg30
Experiment	1D
Number of Scans	160
Receiver Gain	46
Relaxation Delay	1.0000
Pulse Width	9.8000
Acquisition Time	0.4588
Acquisition Date	2015-03-14T10:33:00
Modification Date	2015-03-15T11:42:00
Spectrometer Frequency	100.61
Spectral Width	40760.9
Lowest Frequency	-10320.1
Nucleus	13C
Acquired Size	32768
Spectral Size	65536

1-(1-isopropyl-1H-indol-2-yl)-2-methylprop-2-en-1-ol

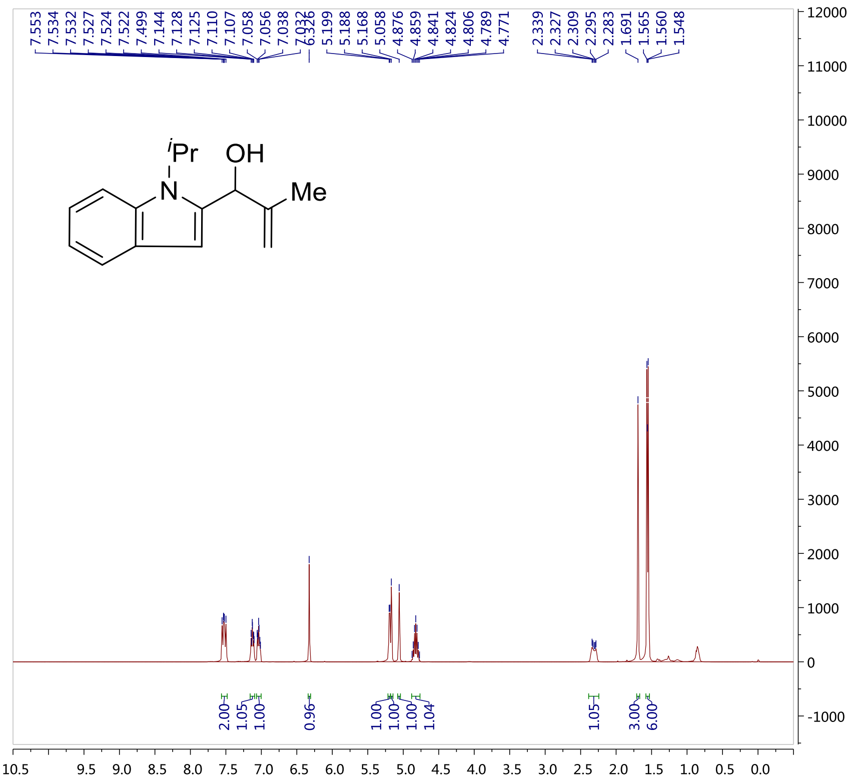

Parameters	
Parameter	Value
Title	wgp-16-86
Comment	PROTON
Origin	Bruker BioSpin GmbH
Owner	nmr
Site	
Spectrometer	spect
Author	
Solvent	CDCl3
Temperature	295.1
Pulse Sequence	zg30
Experiment	1D
Number of Scans	8
Receiver Gain	32
Relaxation Delay	1.0000
Pulse Width	15.0000
Acquisition Time	2.4999
Acquisition Date	2016-03-24T14:54:43
Modification Date	2016-03-24T14:54:00
Spectrometer Frequency	400.13
Spectral Width	8012.8
Lowest Frequency	-1535.4
Nucleus	1H
Acquired Size	20031
Spectral Size	65536

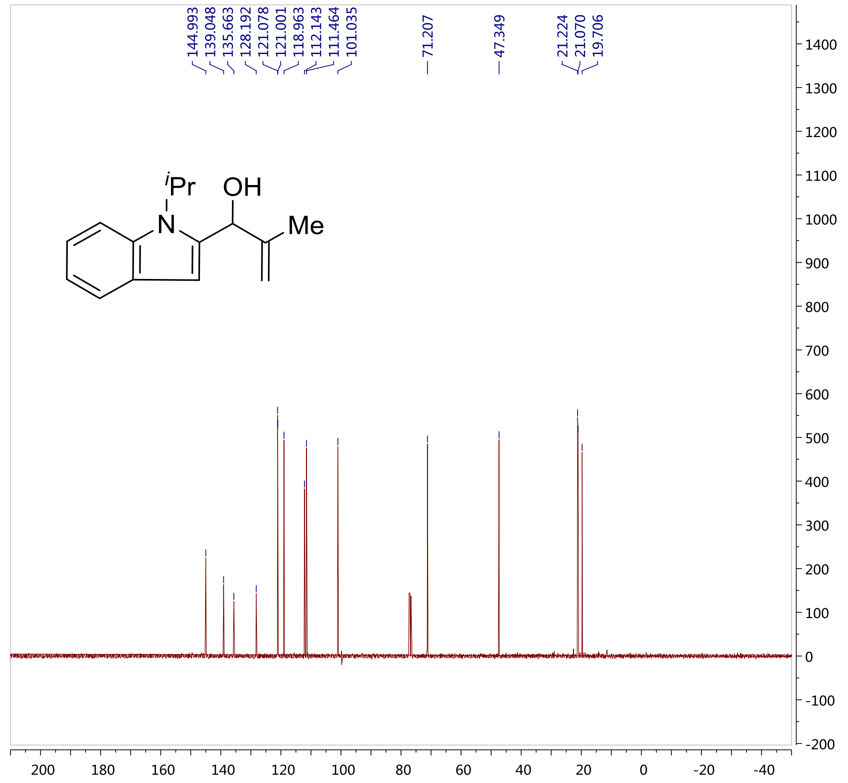

Parameters	
Parameter	Value
Title	wgp-16-86
Comment	C13CPD
Origin	Bruker BioSpin GmbH
Owner	nmr
Site	
Spectrometer	spect
Author	
Solvent	CDCl3
Temperature	295.5
Pulse Sequence	zgpg30
Experiment	1D
Number of Scans	133
Receiver Gain	47
Relaxation Delay	1.0000
Pulse Width	9.8000
Acquisition Time	0.4588
Acquisition Date	2016-03-24T14:56:52
Modification Date	2016-03-24T14:59:00
Spectrometer Frequency	100.61
Spectral Width	35714.3
Lowest Frequency	-7796.8
Nucleus	13C
Acquired Size	16384
Spectral Size	32768

1-(1-allyl-1H-indol-2-yl)-2-methylprop-2-en-1-ol

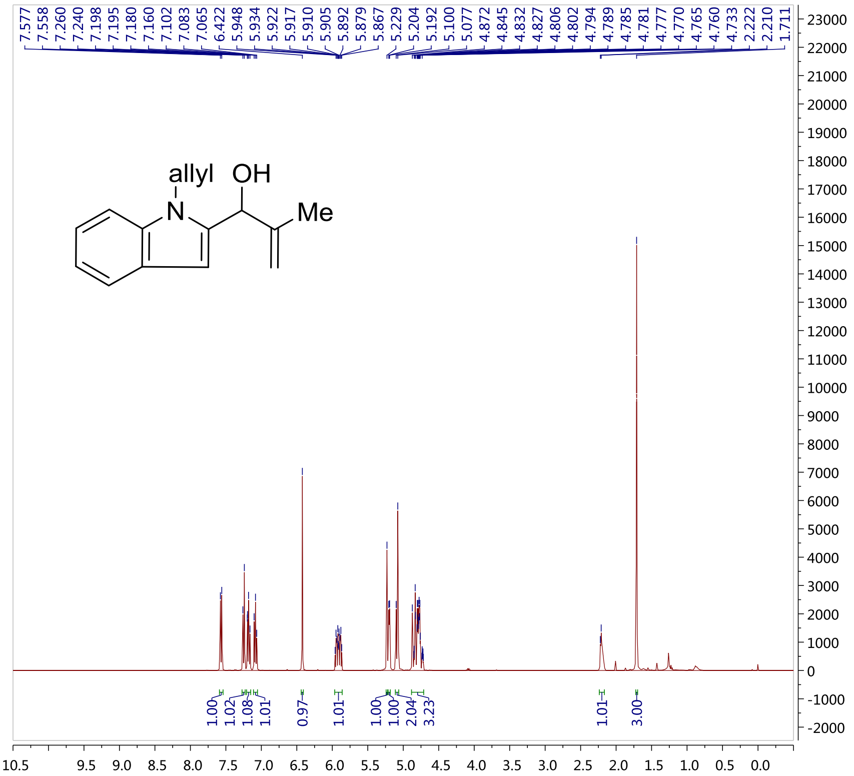

Parameters	
Parameter	Value
Title	wgp-16-85
Comment	test
Origin	Bruker BioSpin GmbH
Owner	nmr
Site	
Spectrometer	spect
Author	
Solvent	CDCl3
Temperature	293.4
Pulse Sequence	zg30
Experiment	1D
Number of Scans	8
Receiver Gain	32
Relaxation Delay	1.0000
Pulse Width	9.8000
Acquisition Time	1.9999
Acquisition Date	2016-03-23T19:50:04
Modification Date	2016-03-23T19:50:00
Spectrometer Frequency	400.13
Spectral Width	8012.8
Lowest Frequency	-1535.4
Nucleus	1H
Acquired Size	16025
Spectral Size	32768

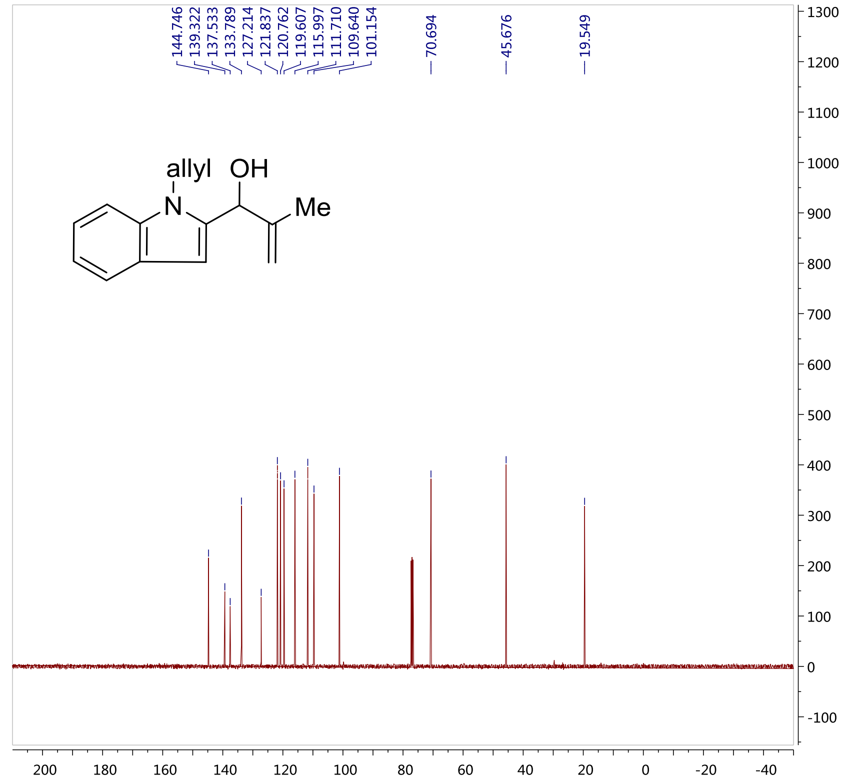

Parameters	
Parameter	Value
Title	wgp-16-85
Comment	test
Origin	Bruker BioSpin GmbH
Owner	nmr
Site	
Spectrometer	spect
Author	
Solvent	CDCl3
Temperature	293.9
Pulse Sequence	zgpg30
Experiment	1D
Number of Scans	104
Receiver Gain	87
Relaxation Delay	1.0000
Pulse Width	9.8000
Acquisition Time	0.8039
Acquisition Date	2016-03-23T19:52:16
Modification Date	2016-03-23T19:54:00
Spectrometer Frequency	100.61
Spectral Width	40760.9
Lowest Frequency	-10320.1
Nucleus	13C
Acquired Size	32768
Spectral Size	65536

1-(1-benzyl-1H-indol-2-yl)-2-methylprop-2-en-1-ol

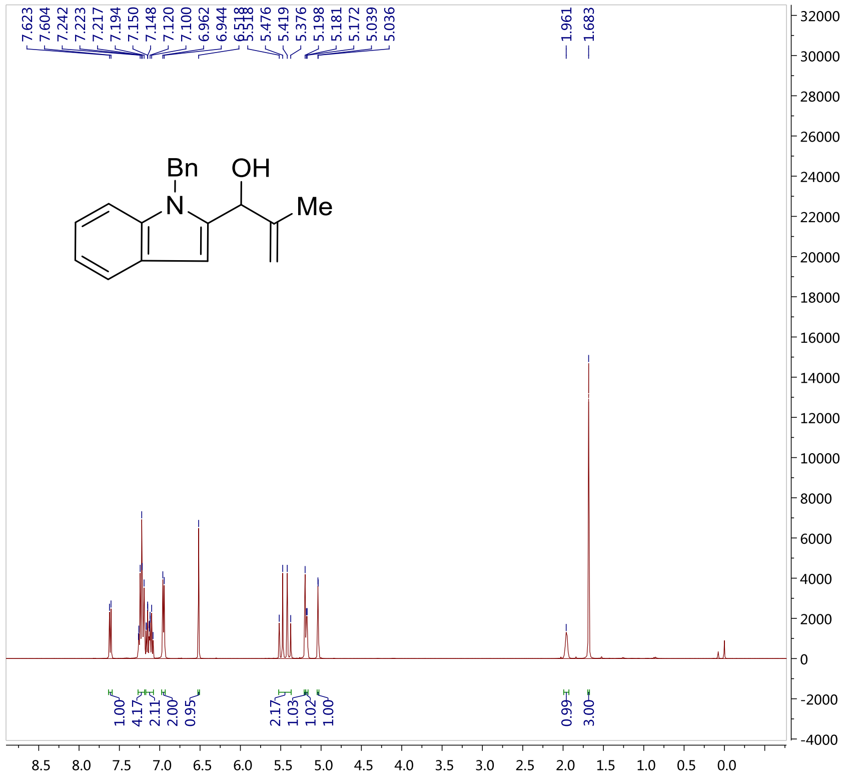

Parameters	
Parameter	Value
Title	wgp-15-181
Comment	test
Origin	Bruker BioSpin GmbH
Owner	nmr
Site	
Spectrometer	spect
Author	
Solvent	CDCl3
Temperature	293.2
Pulse Sequence	zg30
Experiment	1D
Number of Scans	8
Receiver Gain	71
Relaxation Delay	1.0000
Pulse Width	9.8000
Acquisition Time	1.9999
Acquisition Date	2015-11-16T21:02:43
Modification Date	2015-11-16T21:02:00
Spectrometer Frequency	400.13
Spectral Width	8012.8
Lowest Frequency	-1535.4
Nucleus	1H
Acquired Size	16025
Spectral Size	32768

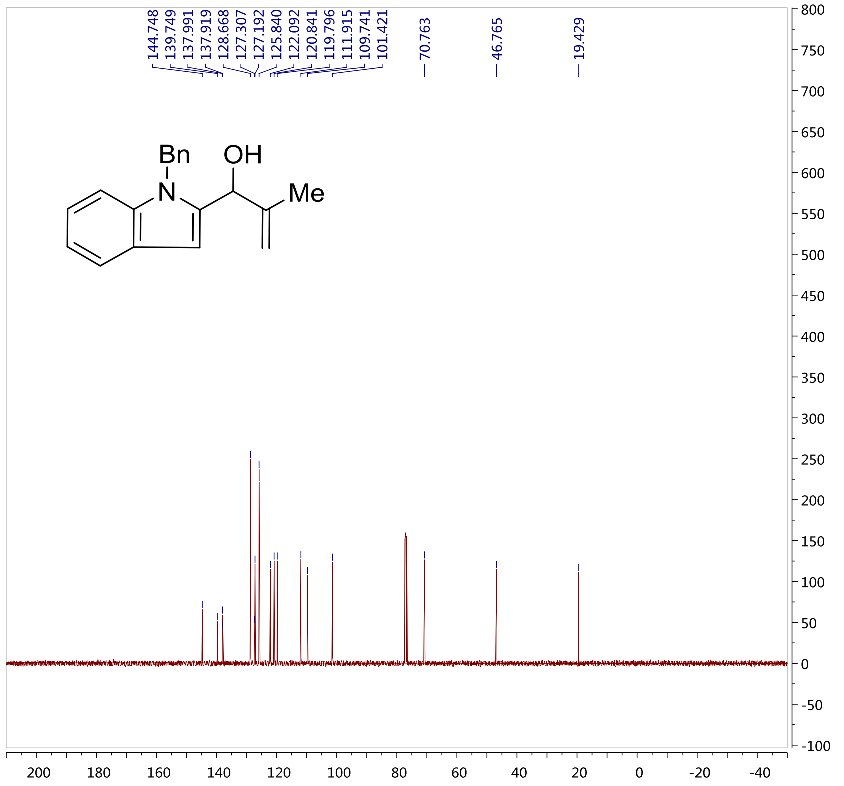

Parameters	
Parameter	Value
Title	wgp-15-181
Comment	test
Origin	Bruker BioSpin GmbH
Owner	nmr
Site	
Spectrometer	spect
Author	
Solvent	CDCl3
Temperature	293.5
Pulse Sequence	zgpg30
Experiment	1D
Number of Scans	119
Receiver Gain	55
Relaxation Delay	1.0000
Pulse Width	9.8000
Acquisition Time	0.8039
Acquisition Date	2015-11-16T21:04:48
Modification Date	2015-11-16T21:07:00
Spectrometer Frequency	100.61
Spectral Width	40760.9
Lowest Frequency	-10320.1
Nucleus	13C
Acquired Size	32768
Spectral Size	65536

2-methyl-1-(1-phenyl-1H-indol-2-yl)prop-2-en-1-ol

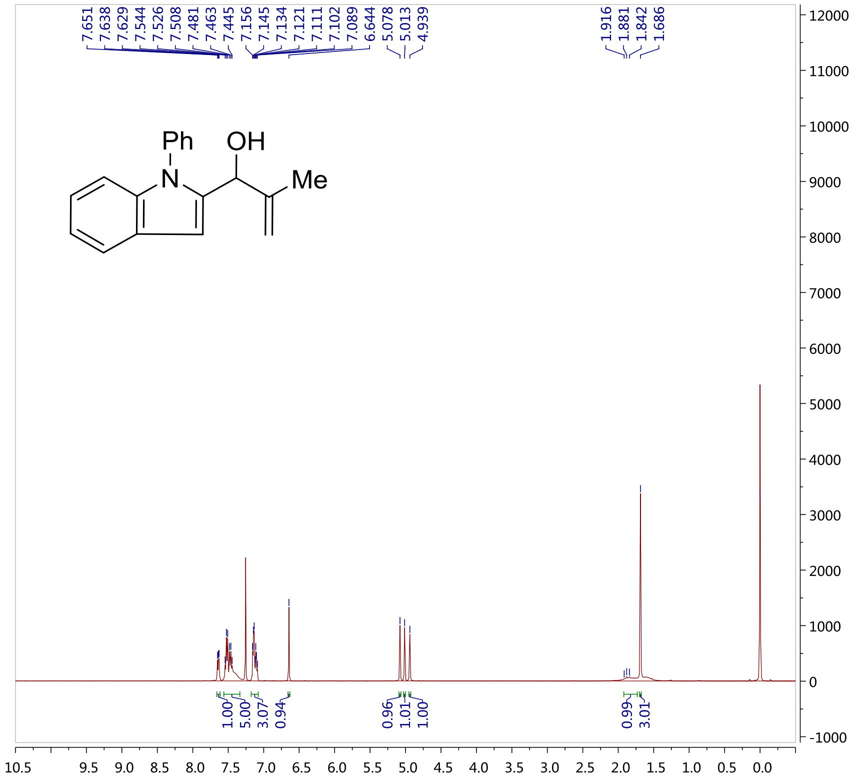

Parameters	
Parameter	Value
Title	wgp-15-115
Comment	PROTON
Origin	Bruker BioSpin GmbH
Owner	common
Site	
Spectrometer	spect
Author	
Solvent	CDCl3
Temperature	295.1
Pulse Sequence	zg30
Experiment	1D
Number of Scans	8
Receiver Gain	203
Relaxation Delay	1.0000
Pulse Width	13.7000
Acquisition Time	1.9999
Acquisition Date	2015-08-31T22:24:00
Modification Date	2015-08-31T22:24:00
Spectrometer Frequency	400.13
Spectral Width	8223.7
Lowest Frequency	-1640.9
Nucleus	1H
Acquired Size	16446
Spectral Size	65536

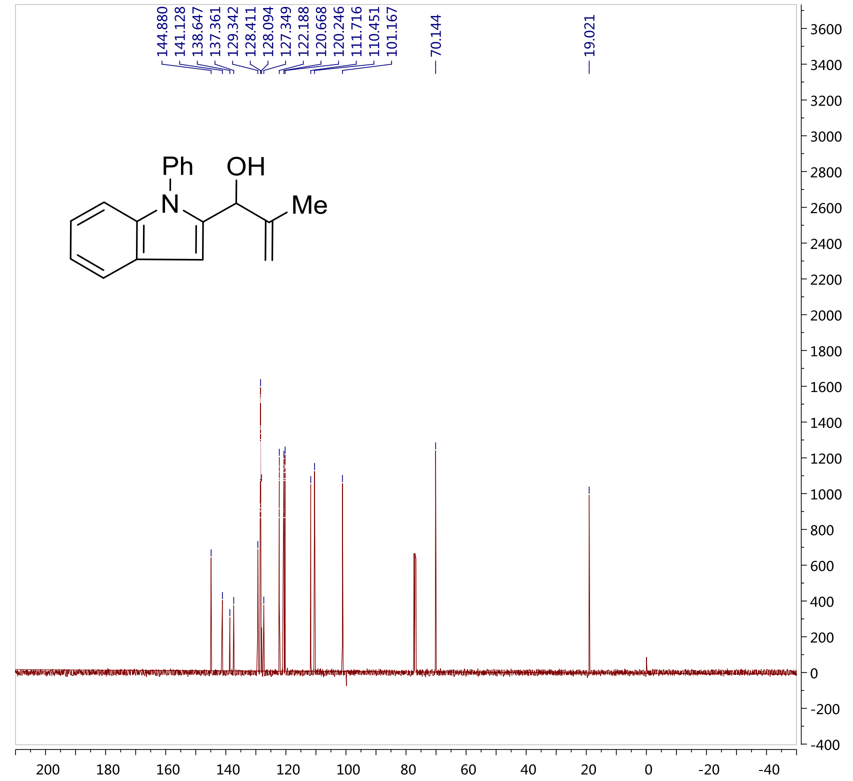

Parameters	
Parameter	Value
Title	wgp-15-115
Comment	C13CPD
Origin	Bruker BioSpin GmbH
Owner	common
Site	
Spectrometer	spect
Author	
Solvent	CDCl3
Temperature	295.3
Pulse Sequence	zgpg30
Experiment	1D
Number of Scans	133
Receiver Gain	203
Relaxation Delay	1.0000
Pulse Width	12.0000
Acquisition Time	0.4588
Acquisition Date	2015-08-31T22:29:00
Modification Date	2015-08-31T22:32:00
Spectrometer Frequency	100.61
Spectral Width	35714.3
Lowest Frequency	-7796.3
Nucleus	13C
Acquired Size	16384
Spectral Size	32768

1-(1-(4-methoxyphenyl)-1H-indol-2-yl)-2-methylprop-2-en-1-ol

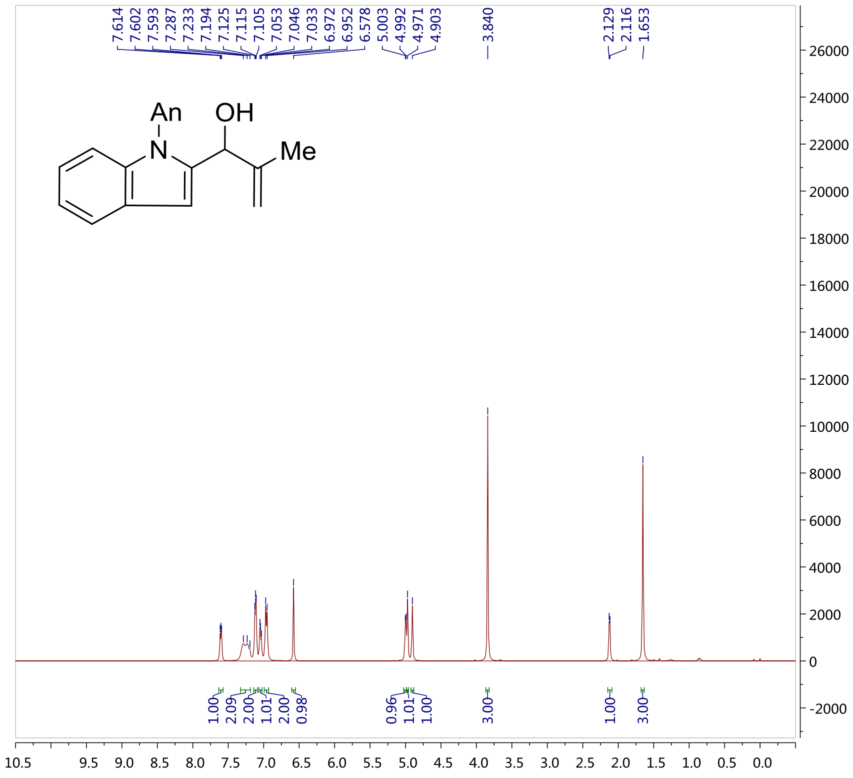

Parameters	
Parameter	Value
Title	wgp-16-73
Comment	test
Origin	Bruker BioSpin GmbH
Owner	nmr
Site	
Spectrometer	spect
Author	
Solvent	CDCl3
Temperature	292.8
Pulse Sequence	zg30
Experiment	1D
Number of Scans	8
Receiver Gain	32
Relaxation Delay	1.0000
Pulse Width	9.8000
Acquisition Time	1.9999
Acquisition Date	2016-03-14T14:49:22
Modification Date	2016-03-14T14:49:00
Spectrometer Frequency	400.13
Spectral Width	8012.8
Lowest Frequency	-1535.4
Nucleus	1H
Acquired Size	16025
Spectral Size	32768

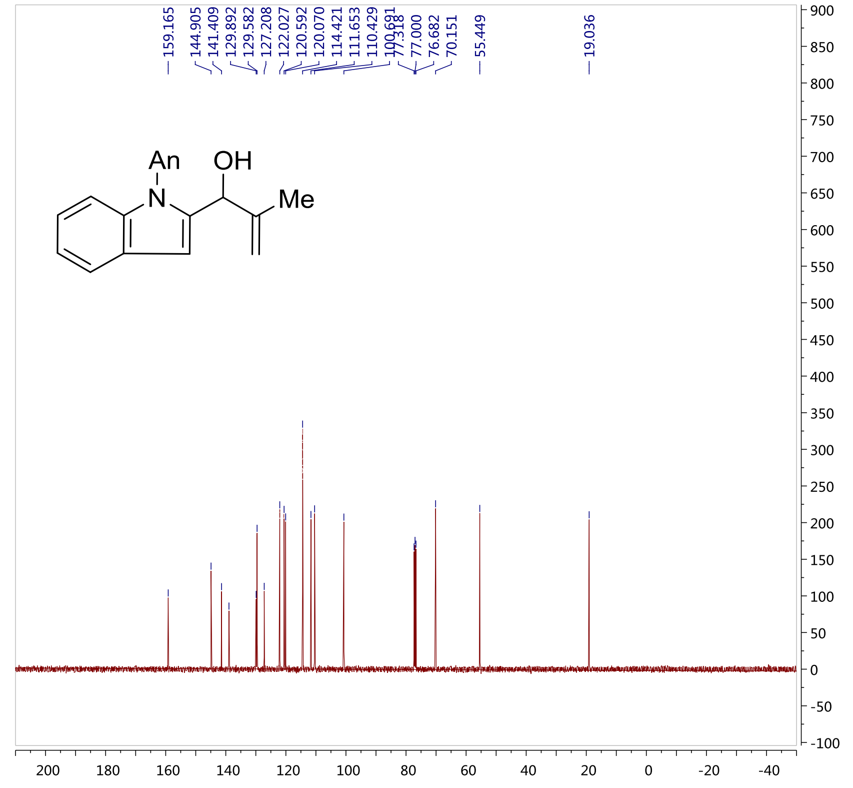

Parameters	
Parameter	Value
Title	wgp-16-73
Comment	test
Origin	Bruker BioSpin GmbH
Owner	nmr
Site	
Spectrometer	spect
Author	
Solvent	CDCl3
Temperature	293.0
Pulse Sequence	zgpg30
Experiment	1D
Number of Scans	70
Receiver Gain	97
Relaxation Delay	1.0000
Pulse Width	9.8000
Acquisition Time	0.8039
Acquisition Date	2016-03-14T14:51:00
Modification Date	2016-03-14T14:52:00
Spectrometer Frequency	100.61
Spectral Width	40760.9
Lowest Frequency	-10320.1
Nucleus	13C
Acquired Size	32768
Spectral Size	65536

2-methyl-1-(1-(4-(trifluoromethyl)phenyl)-1H-indol-2-yl)prop-2-en-1-ol

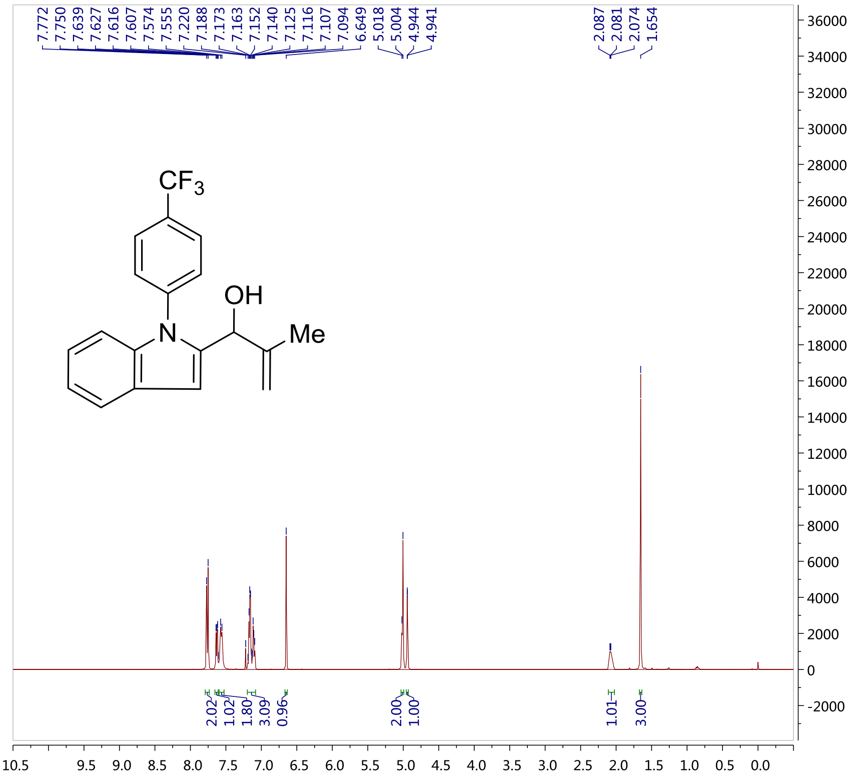

Parameters	
Parameter	Value
Title	wgp-16-74
Comment	test
Origin	Bruker BioSpin GmbH
Owner	nmr
Site	
Spectrometer	spect
Author	
Solvent	CDCl3
Temperature	293.0
Pulse Sequence	zg30
Experiment	1D
Number of Scans	8
Receiver Gain	55
Relaxation Delay	1.0000
Pulse Width	9.8000
Acquisition Time	1.9999
Acquisition Date	2016-03-14T16:35:23
Modification Date	2016-03-14T16:35:00
Spectrometer Frequency	400.13
Spectral Width	8012.8
Lowest Frequency	-1535.4
Nucleus	1H
Acquired Size	16025
Spectral Size	32768

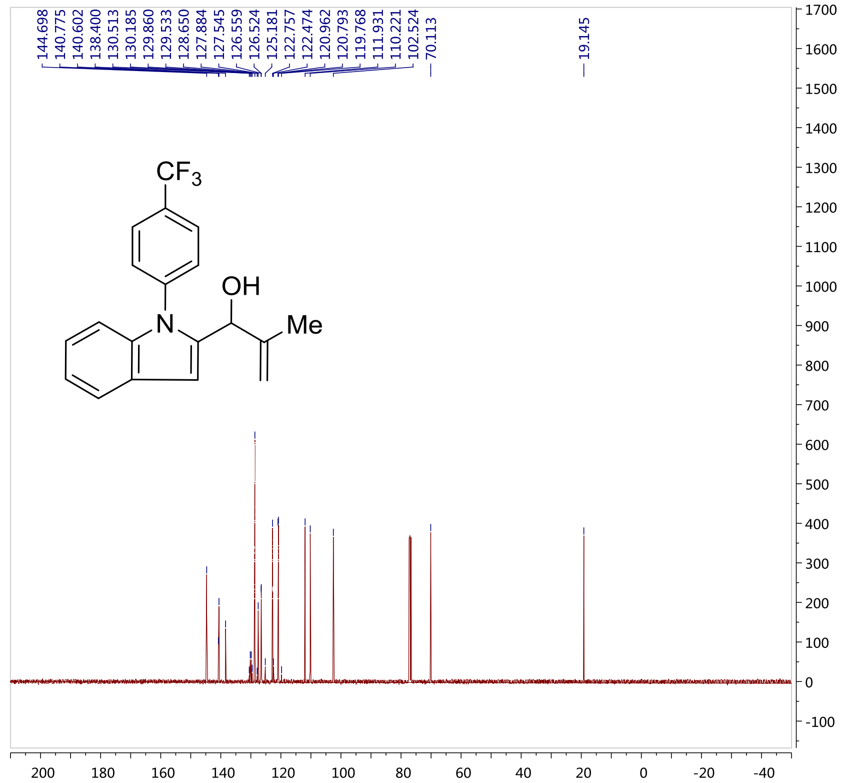

Parameters	
Parameter	Value
Title	wgp-16-74
Comment	test
Origin	Bruker BioSpin GmbH
Owner	nmr
Site	
Spectrometer	spect
Author	
Solvent	CDCl3
Temperature	293.3
Pulse Sequence	zgpg30
Experiment	1D
Number of Scans	205
Receiver Gain	78
Relaxation Delay	1.0000
Pulse Width	9.8000
Acquisition Time	0.8039
Acquisition Date	2016-03-14T16:36:59
Modification Date	2016-03-14T16:42:00
Spectrometer Frequency	100.61
Spectral Width	40760.9
Lowest Frequency	-10320.1
Nucleus	13C
Acquired Size	32768
Spectral Size	65536

2-methyl-1-(1-(naphthalen-2-yl)-1H-indol-2-yl)prop-2-en-1-ol

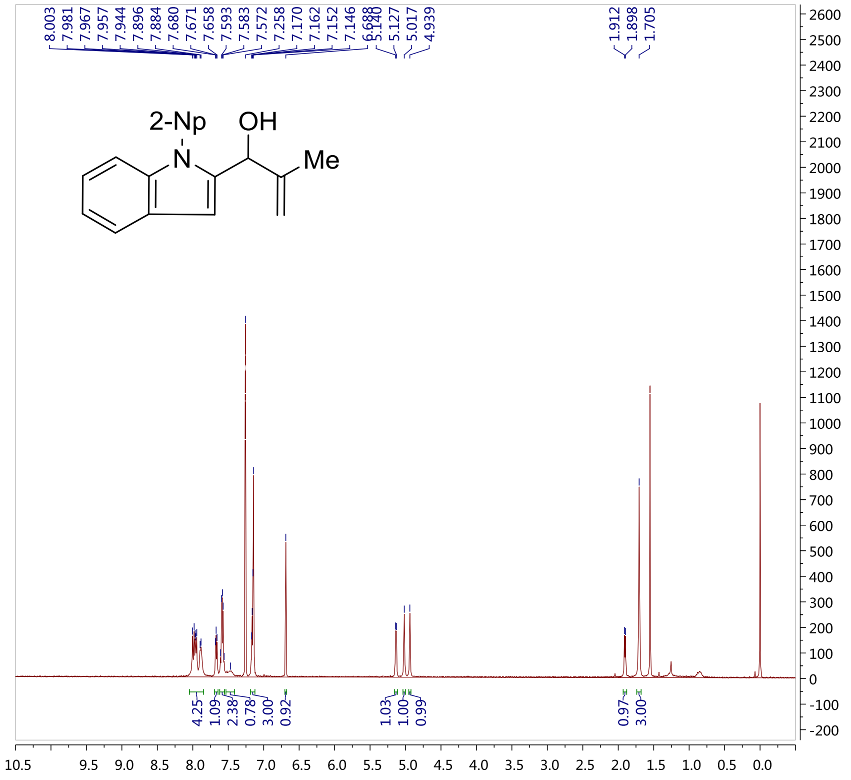

Parameters	
Parameter	Value
Title	wgp-16-99
Comment	PROTON
Origin	Bruker BioSpin GmbH
Owner	common
Site	
Spectrometer	spect
Author	
Solvent	CDCl3
Temperature	2753.5
Pulse Sequence	zg30
Experiment	1D
Number of Scans	8
Receiver Gain	144
Relaxation Delay	1.0000
Pulse Width	13.5000
Acquisition Time	1.9923
Acquisition Date	2016-04-03T19:20:00
Modification Date	2016-04-03T19:20:00
Spectrometer Frequency	400.23
Spectral Width	8223.7
Lowest Frequency	-1640.3
Nucleus	1H
Acquired Size	16384
Spectral Size	32768

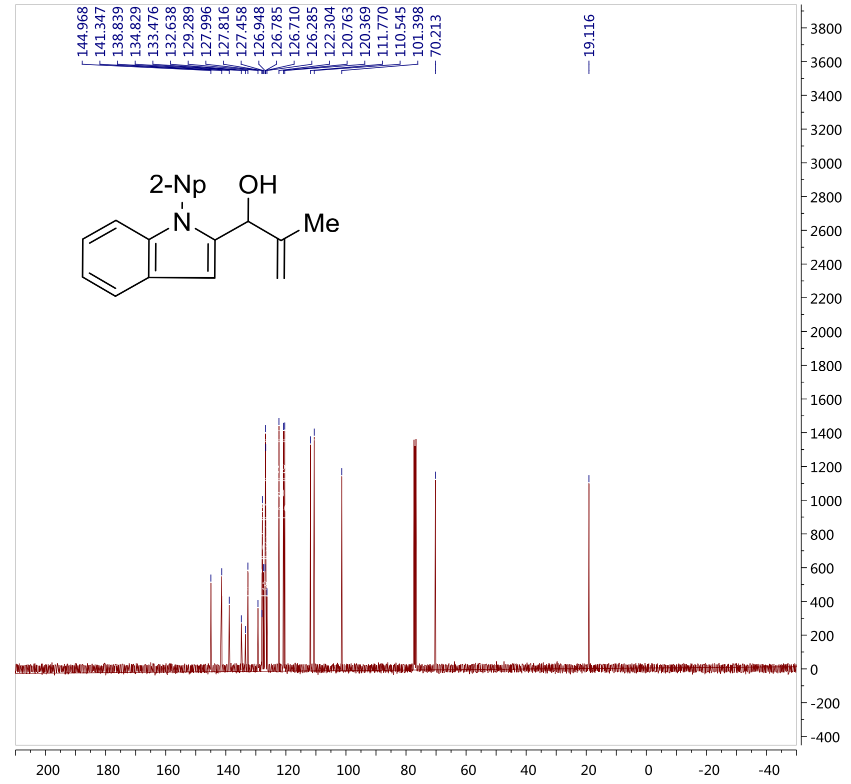

Parameters	
Parameter	Value
Title	wgp-16-99
Comment	13CPD
Origin	Bruker BioSpin GmbH
Owner	nmr
Site	
Spectrometer	spect
Author	
Solvent	CDCl3
Temperature	293.5
Pulse Sequence	zgpg30
Experiment	1D
Number of Scans	306
Receiver Gain	205
Relaxation Delay	1.0000
Pulse Width	9.3000
Acquisition Time	0.8039
Acquisition Date	2016-04-03T18:34:29
Modification Date	2016-04-03T18:43:00
Spectrometer Frequency	100.61
Spectral Width	40760.9
Lowest Frequency	-15349.8
Nucleus	13C
Acquired Size	32768
Spectral Size	65536

1-(1-methyl-1H-indol-2-yl)-2-methylenebutan-1-ol

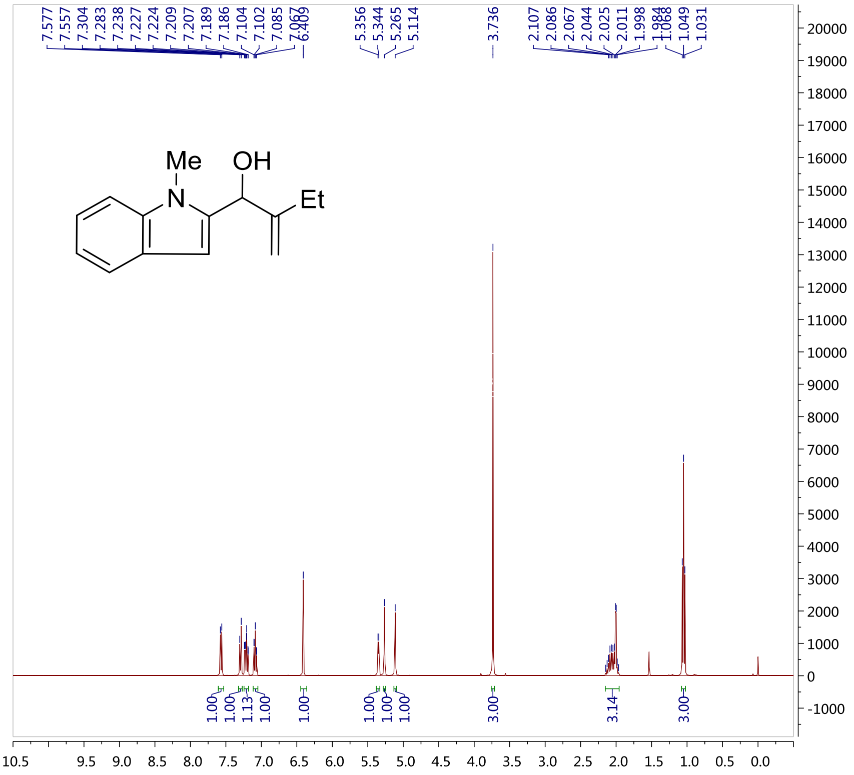

Parameters	
Parameter	Value
Title	wgp-14-185
Comment	PROTON
Origin	Bruker BioSpin GmbH
Owner	nmr
Site	
Spectrometer	spect
Author	
Solvent	CDCl3
Temperature	302.4
Pulse Sequence	zg30
Experiment	1D
Number of Scans	8
Receiver Gain	161
Relaxation Delay	1.0000
Pulse Width	13.7000
Acquisition Time	1.9999
Acquisition Date	2015-03-18T21:01:00
Modification Date	2015-03-18T21:01:00
Spectrometer Frequency	400.13
Spectral Width	8223.7
Lowest Frequency	-1640.9
Nucleus	1H
Acquired Size	16446
Spectral Size	65536

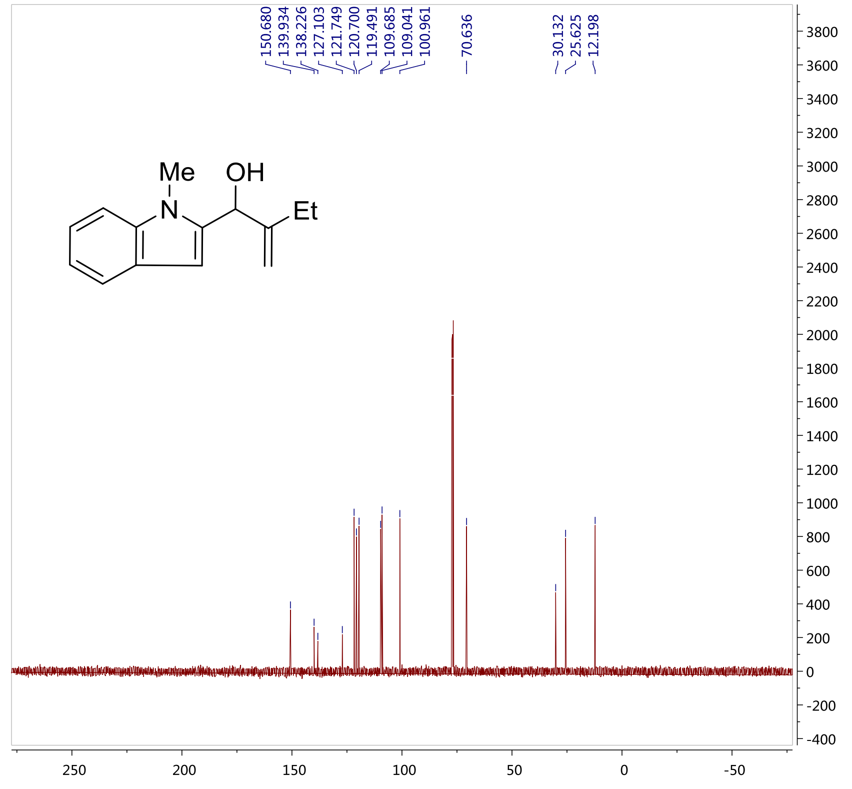

Parameters	
Parameter	Value
Title	wgp-14-185
Comment	C13CPD
Origin	Bruker BioSpin GmbH
Owner	nmr
Site	
Spectrometer	spect
Author	
Solvent	CDCl3
Temperature	302.8
Pulse Sequence	zgpg30
Experiment	1D
Number of Scans	323
Receiver Gain	203
Relaxation Delay	1.0000
Pulse Width	12.0000
Acquisition Time	0.4588
Acquisition Date	2015-03-18T21:03:00
Modification Date	2015-03-18T21:10:00
Spectrometer Frequency	100.61
Spectral Width	35714.3
Lowest Frequency	-7796.3
Nucleus	13C
Acquired Size	16384
Spectral Size	32768

3-methyl-1-(1-methyl-1H-indol-2-yl)-2-methylenebutan-1-ol

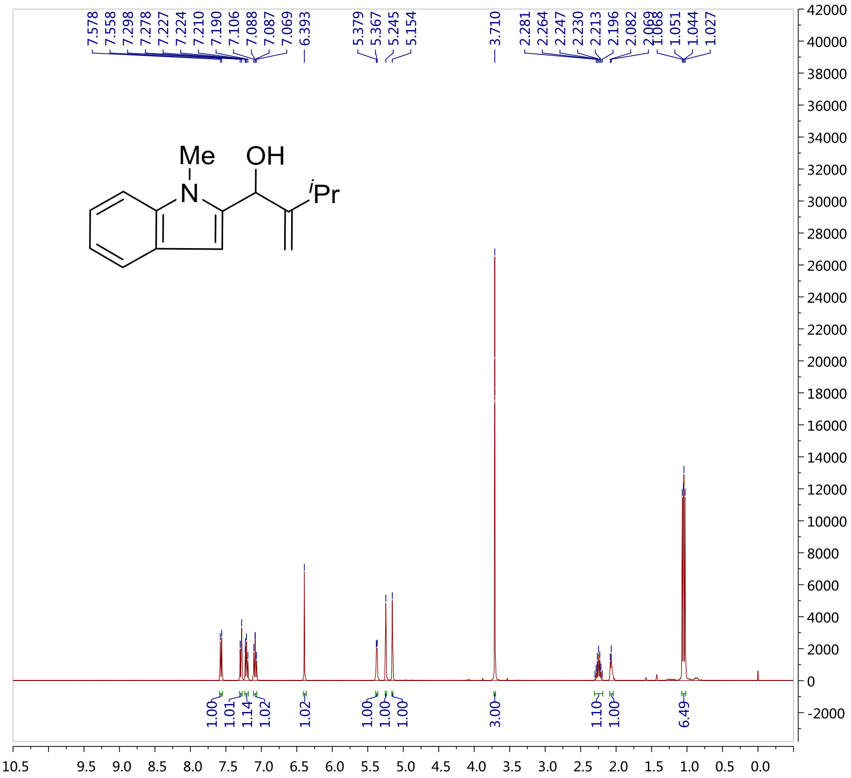

Parameters	
Parameter	Value
Title	wgp-15-186
Comment	test
Origin	Bruker BioSpin GmbH
Owner	nmr
Site	
Spectrometer	spect
Author	
Solvent	CDCl3
Temperature	292.7
Pulse Sequence	zg30
Experiment	1D
Number of Scans	8
Receiver Gain	55
Relaxation Delay	1.0000
Pulse Width	9.8000
Acquisition Time	1.9999
Acquisition Date	2015-11-19T18:12:32
Modification Date	2015-11-19T18:12:00
Spectrometer Frequency	400.13
Spectral Width	8012.8
Lowest Frequency	-1535.4
Nucleus	1H
Acquired Size	16025
Spectral Size	32768

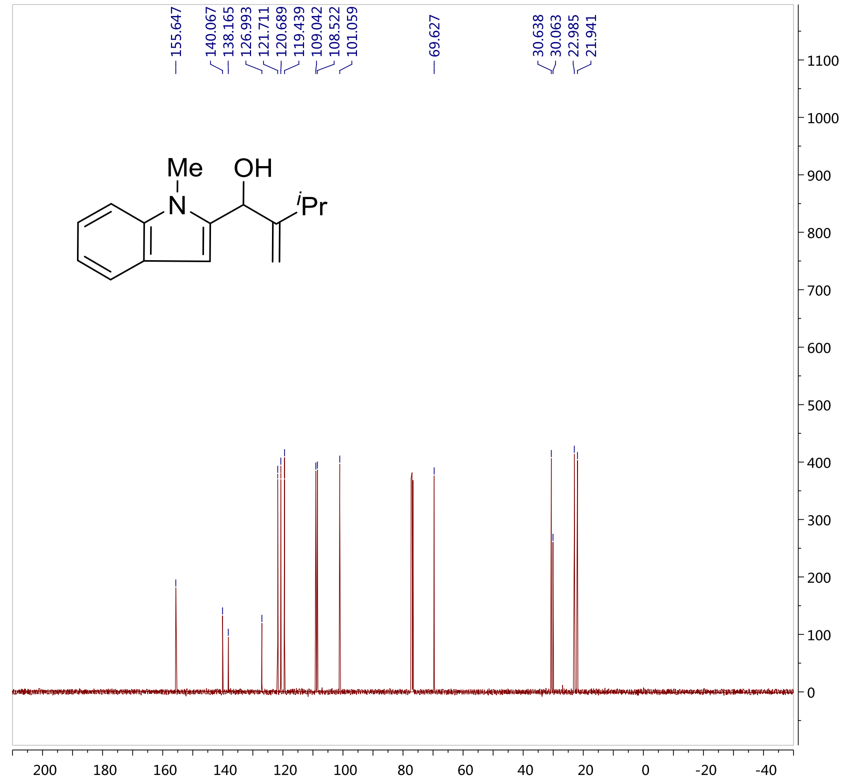

Parameters	
Parameter	Value
Title	wgp-15-186
Comment	test
Origin	Bruker BioSpin GmbH
Owner	nmr
Site	
Spectrometer	spect
Author	
Solvent	CDCl3
Temperature	293.3
Pulse Sequence	zgpg30
Experiment	1D
Number of Scans	319
Receiver Gain	50
Relaxation Delay	1.0000
Pulse Width	9.8000
Acquisition Time	0.8039
Acquisition Date	2015-11-19T18:14:55
Modification Date	2015-11-19T18:23:00
Spectrometer Frequency	100.61
Spectral Width	40760.9
Lowest Frequency	-10320.1
Nucleus	13C
Acquired Size	32768
Spectral Size	65536

1-(1-methyl-1H-indol-2-yl)-2-phenylprop-2-en-1-ol

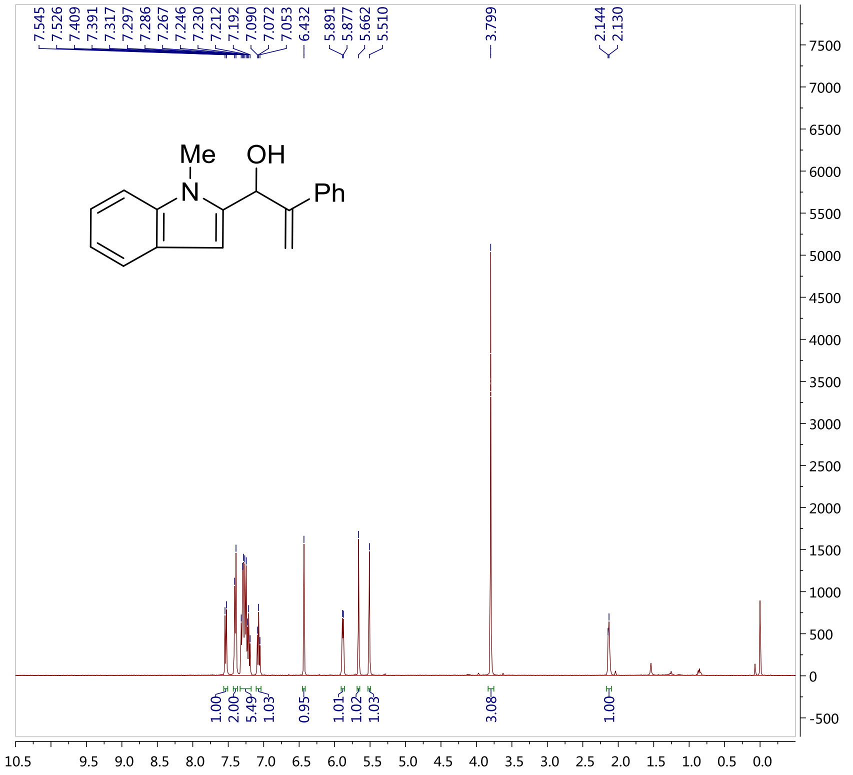

Parameters	
Parameter	Value
Title	wgp-15-182
Comment	PROTON
Origin	Bruker BioSpin GmbH
Owner	common
Site	
Spectrometer	spect
Author	
Solvent	CDCl3
Temperature	294.3
Pulse Sequence	zg30
Experiment	1D
Number of Scans	8
Receiver Gain	203
Relaxation Delay	1.0000
Pulse Width	13.7000
Acquisition Time	1.9999
Acquisition Date	2015-11-17T11:39:00
Modification Date	2015-11-17T11:39:00
Spectrometer Frequency	400.13
Spectral Width	8223.7
Lowest Frequency	-1640.9
Nucleus	1H
Acquired Size	16446
Spectral Size	65536

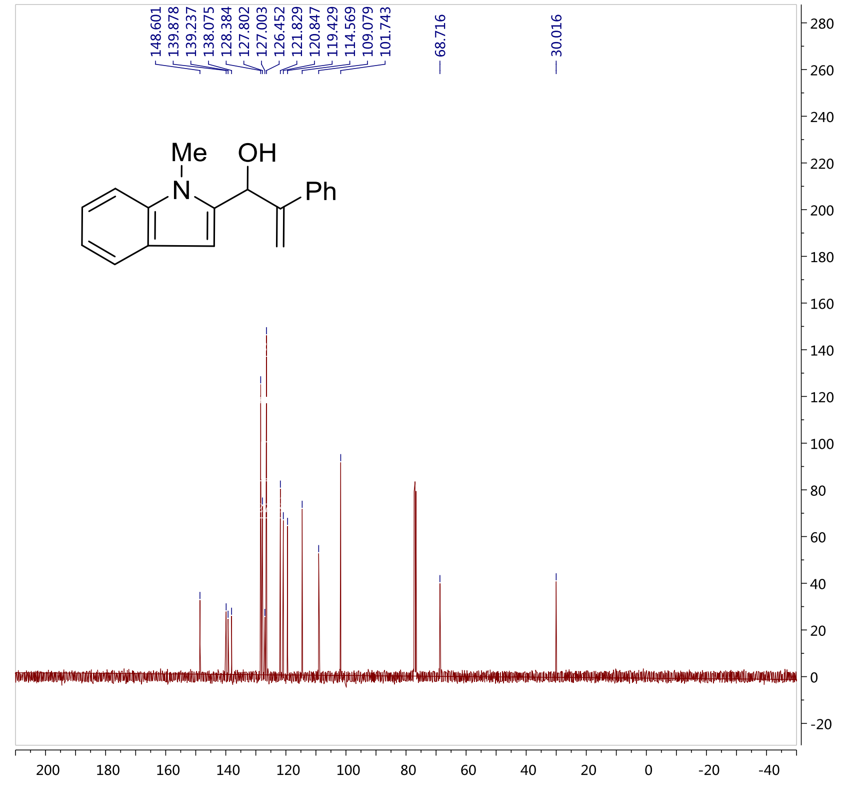

Parameters	
Parameter	Value
Title	wgp-15-182
Comment	test
Origin	Bruker BioSpin GmbH
Owner	nmr
Site	
Spectrometer	spect
Author	
Solvent	CDCl3
Temperature	293.6
Pulse Sequence	zgpg30
Experiment	1D
Number of Scans	111
Receiver Gain	46
Relaxation Delay	1.0000
Pulse Width	9.8000
Acquisition Time	0.8039
Acquisition Date	2015-11-17T11:04:30
Modification Date	2015-11-17T11:07:00
Spectrometer Frequency	100.61
Spectral Width	40760.9
Lowest Frequency	-10320.1
Nucleus	13C
Acquired Size	32768
Spectral Size	65536

2-(4-methoxyphenyl)-1-(1-methyl-1H-indol-2-yl)prop-2-en-1-ol

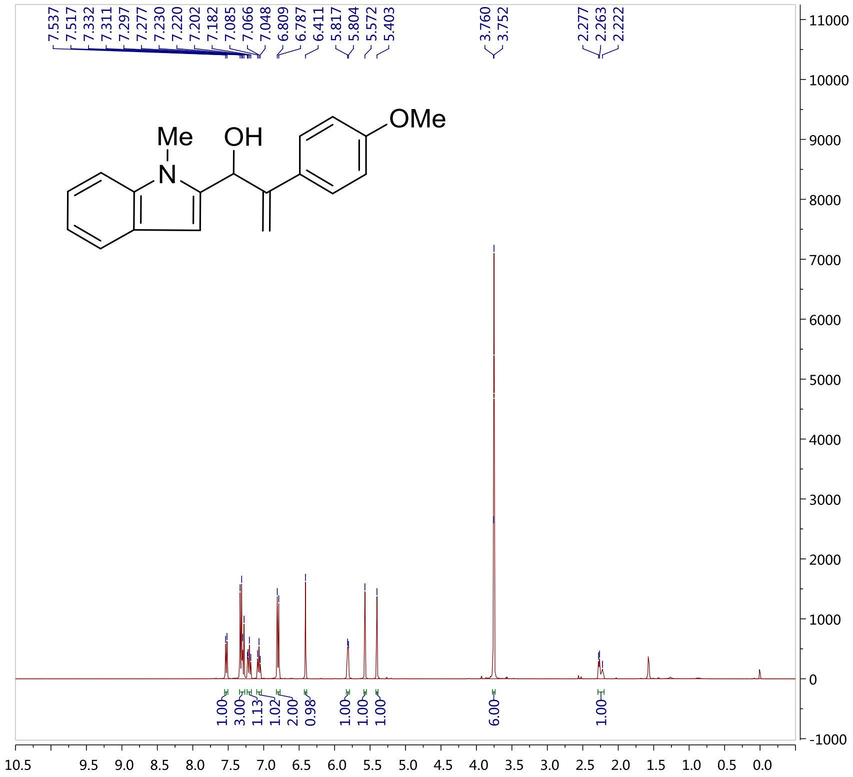

	Parameter	Value
1	Data File Name	E:/ 0 NMR & MS/ 吲哚底物/ wgp-17-12/ 1/ fid
2	Title	wgp-17-12
3	Comment	PROTON
4	Origin	Bruker BioSpin GmbH
5	Owner	nmr
6	Site	
7	Spectrometer	spect
8	Author	
9	Solvent	CDCl3
10	Temperature	298.8
11	Pulse Sequence	zg30
12	Experiment	1D
13	Number of Scans	8
14	Receiver Gain	52
15	Relaxation Delay	1.0000
16	Pulse Width	9.1200
17	Acquisition Time	4.0894
18	Acquisition Date	2016-08-10T08:49:33
19	Modification Date	2016-08-10T08:49:00
20	Spectrometer Frequency	400.13
21	Spectral Width	8012.8
22	Lowest Frequency	-1535.4
23	Nucleus	1H
24	Acquired Size	32768
25	Spectral Size	65536

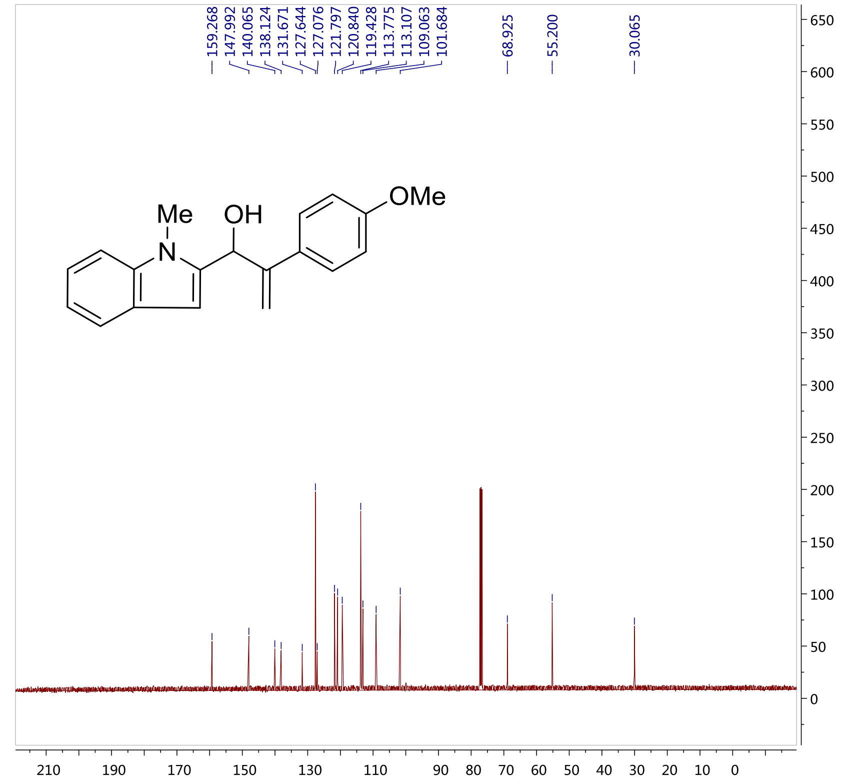

	Parameter	Value
1	Data File Name	E:/ 0 NMR & MS/ 吲哚底物/ wgp-17-12/ 2/ fid
2	Title	wgp-17-12
3	Comment	C13CPD
4	Origin	Bruker BioSpin GmbH
5	Owner	nmr
6	Site	
7	Spectrometer	spect
8	Author	
9	Solvent	CDCl3
10	Temperature	299.2
11	Pulse Sequence	zgpg30
12	Experiment	1D
13	Number of Scans	166
14	Receiver Gain	33
15	Relaxation Delay	2.0000
16	Pulse Width	9.4000
17	Acquisition Time	1.3631
18	Acquisition Date	2016-08-10T08:51:05
19	Modification Date	2016-08-10T08:59:00
20	Spectrometer Frequency	100.61
21	Spectral Width	24038.5
22	Lowest Frequency	-1958.9
23	Nucleus	13C
24	Acquired Size	32768
25	Spectral Size	65536

2-(4-fluorophenyl)-1-(1-methyl-1H-indol-2-yl)prop-2-en-1-ol

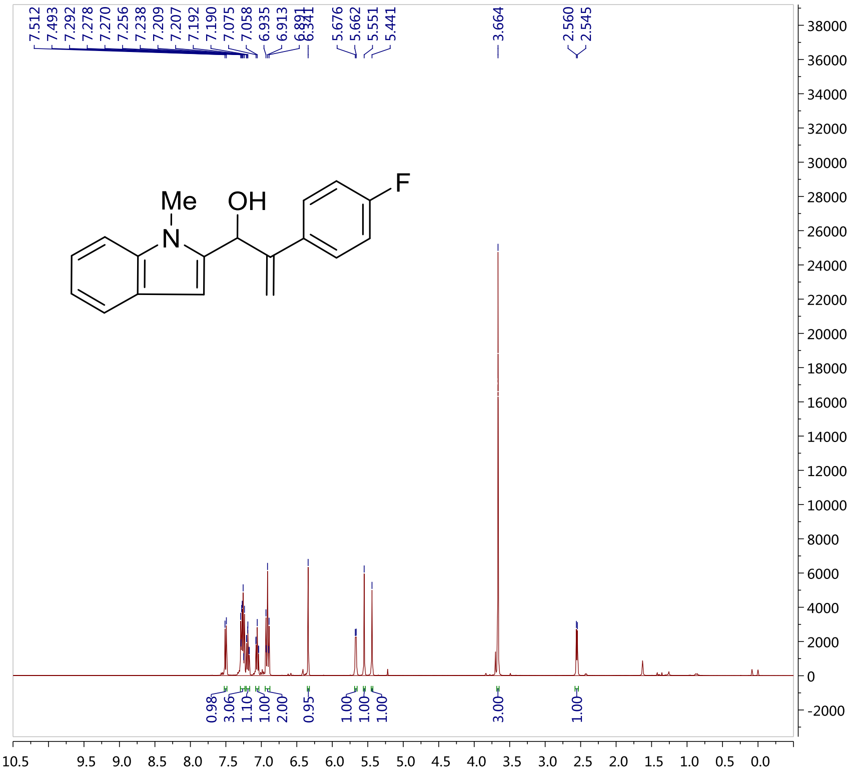

Parameters	
Parameter	Value
Title	wgp-16-190
Comment	PROTON
Origin	Bruker BioSpin GmbH
Owner	common
Site	
Spectrometer	spect
Author	
Solvent	CDCl3
Temperature	-1616.9
Pulse Sequence	zg30
Experiment	1D
Number of Scans	8
Receiver Gain	72
Relaxation Delay	1.0000
Pulse Width	13.5000
Acquisition Time	1.9923
Acquisition Date	2016-07-16T20:31:00
Modification Date	2016-07-16T20:31:00
Spectrometer Frequency	400.23
Spectral Width	8223.7
Lowest Frequency	-1640.3
Nucleus	1H
Acquired Size	16384
Spectral Size	32768

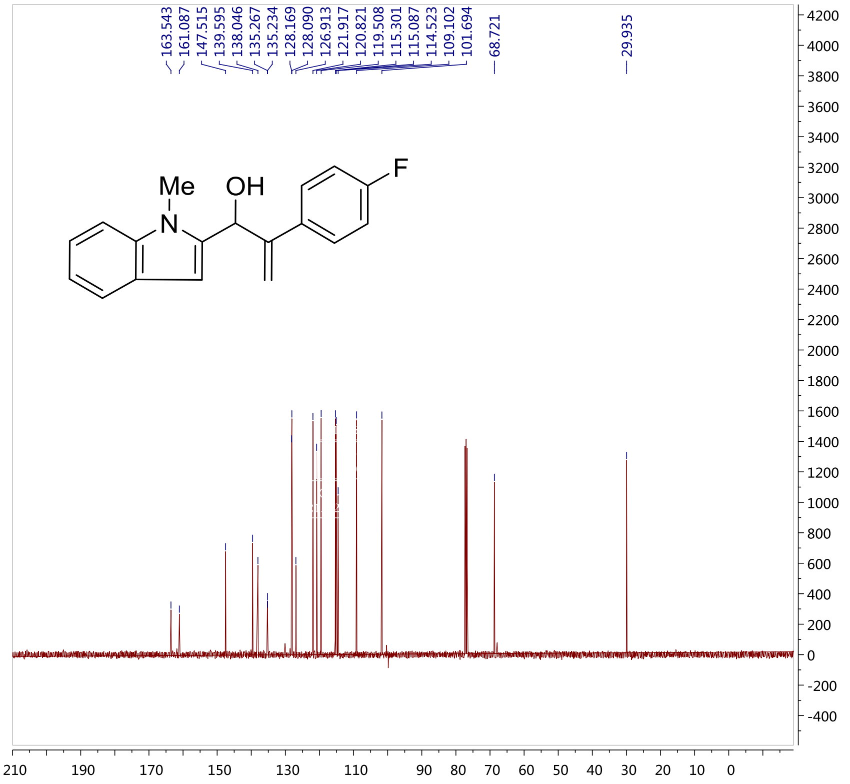

Parameters	
Parameter	Value
Title	wgp-16-190
Comment	C13CPD
Origin	Bruker BioSpin GmbH
Owner	common
Site	
Spectrometer	spect
Author	
Solvent	CDCl3
Temperature	-1598.7
Pulse Sequence	zgpg30
Experiment	1D
Number of Scans	253
Receiver Gain	203
Relaxation Delay	1.0000
Pulse Width	12.0000
Acquisition Time	0.5506
Acquisition Date	2016-07-16T20:36:00
Modification Date	2016-07-16T20:39:00
Spectrometer Frequency	100.64
Spectral Width	29761.9
Lowest Frequency	-4817.6
Nucleus	13C
Acquired Size	16384
Spectral Size	32768

2-(4-chlorophenyl)-1-(1-methyl-1H-indol-2-yl)prop-2-en-1-ol

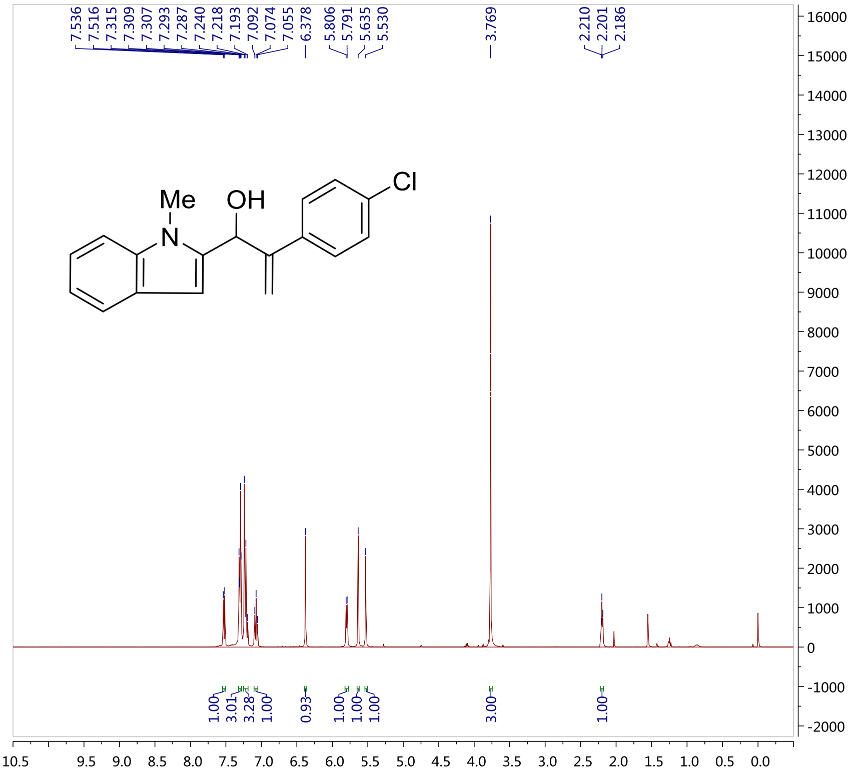

Parameters	
Parameter	Value
Title	wgp-16-188
Comment	PROTON
Origin	Bruker BioSpin GmbH
Owner	common
Site	
Spectrometer	spect
Author	
Solvent	CDCl3
Temperature	299.5
Pulse Sequence	zg30
Experiment	1D
Number of Scans	8
Receiver Gain	203
Relaxation Delay	1.0000
Pulse Width	13.7000
Acquisition Time	1.9999
Acquisition Date	2016-07-14T17:23:00
Modification Date	2016-07-14T17:23:00
Spectrometer Frequency	400.13
Spectral Width	8223.7
Lowest Frequency	-1640.9
Nucleus	1H
Acquired Size	16446
Spectral Size	65536

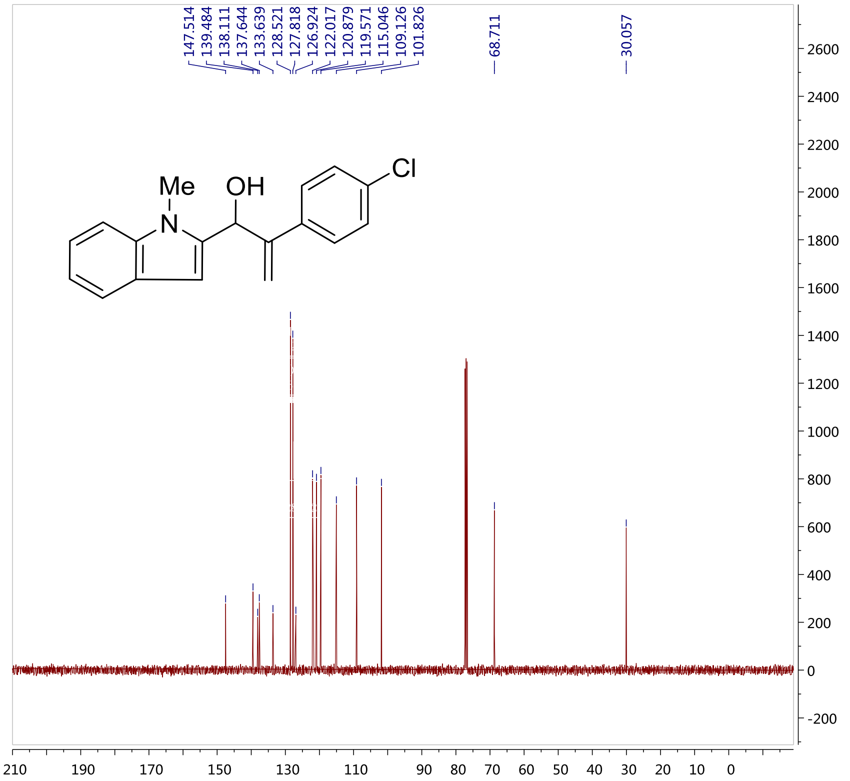

Parameters	
Parameter	Value
Title	wgp-16-188
Comment	C13CPD
Origin	Bruker BioSpin GmbH
Owner	common
Site	
Spectrometer	spect
Author	
Solvent	CDCl3
Temperature	292.7
Pulse Sequence	zgpg30
Experiment	1D
Number of Scans	261
Receiver Gain	203
Relaxation Delay	1.0000
Pulse Width	12.5000
Acquisition Time	0.5506
Acquisition Date	2016-07-14T17:50:00
Modification Date	2016-07-14T17:56:00
Spectrometer Frequency	100.61
Spectral Width	29761.9
Lowest Frequency	-4820.2
Nucleus	13C
Acquired Size	16384
Spectral Size	32768

2-(benzo[d][1,3]dioxol-5-yl)-1-(1-methyl-1H-indol-2-yl)prop-2-en-1-ol

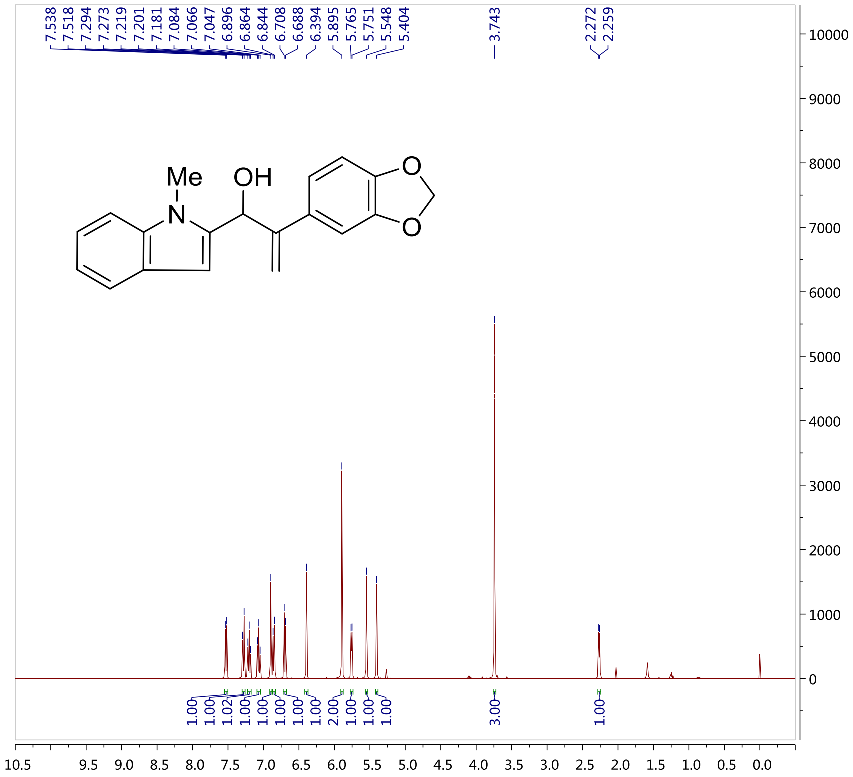

	Parameter	Value
1	Data File Name	E:/ 0 NMR & MS/ 吲哚底物/ cmq-1-46/ 1/ fid
2	Title	cmq-1-46
3	Comment	PROTON
4	Origin	Bruker BioSpin GmbH
5	Owner	nmr
6	Site	
7	Spectrometer	spect
8	Author	
9	Solvent	CDCl3
10	Temperature	291.3
11	Pulse Sequence	zg30
12	Experiment	1D
13	Number of Scans	8
14	Receiver Gain	57
15	Relaxation Delay	1.0000
16	Pulse Width	9.1200
17	Acquisition Time	4.0894
18	Acquisition Date	2016-08-13T15:08:01
19	Modification Date	2016-08-13T15:08:00
20	Spectrometer Frequency	400.13
21	Spectral Width	8012.8
22	Lowest Frequency	-1535.4
23	Nucleus	1H
24	Acquired Size	32768
25	Spectral Size	65536

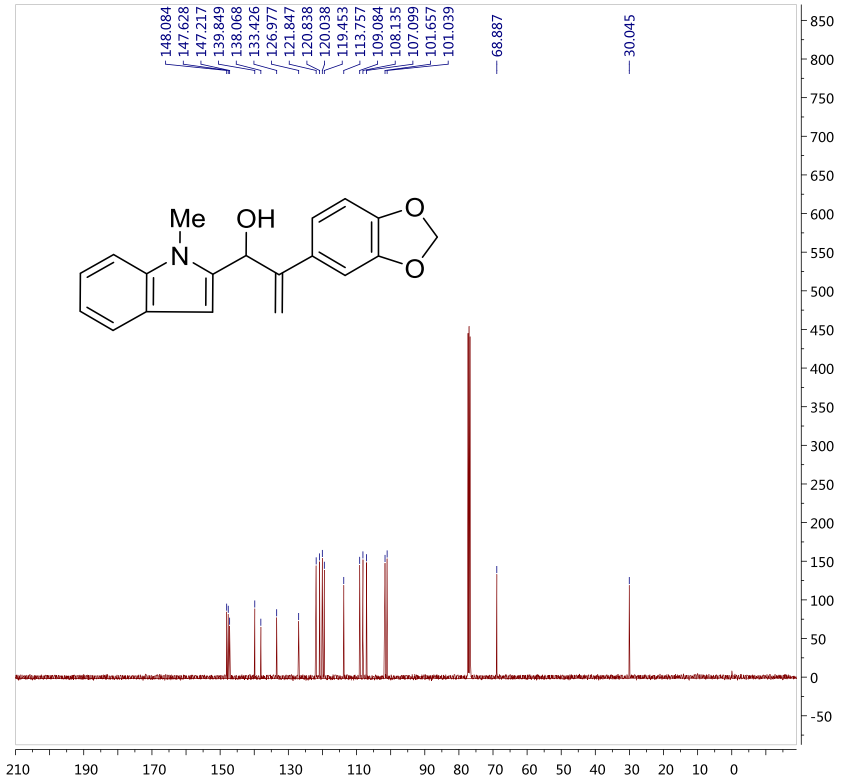

	Parameter	Value
1	Data File Name	E:/ 0 NMR & MS/ 吲哚底物/ cmq-1-46/ 2/ fid
2	Title	cmq-1-46
3	Comment	C13CPD
4	Origin	Bruker BioSpin GmbH
5	Owner	nmr
6	Site	
7	Spectrometer	spect
8	Author	
9	Solvent	CDCl3
10	Temperature	292.5
11	Pulse Sequence	zgpg30
12	Experiment	1D
13	Number of Scans	323
14	Receiver Gain	29
15	Relaxation Delay	2.0000
16	Pulse Width	9.4000
17	Acquisition Time	1.3631
18	Acquisition Date	2016-08-13T15:23:45
19	Modification Date	2016-08-13T15:27:00
20	Spectrometer Frequency	100.61
21	Spectral Width	24038.5
22	Lowest Frequency	-1958.9
23	Nucleus	13C
24	Acquired Size	32768
25	Spectral Size	65536

1-(1,5-dimethyl-1H-indol-2-yl)-3-methyl-2-methylenebutan-1-ol

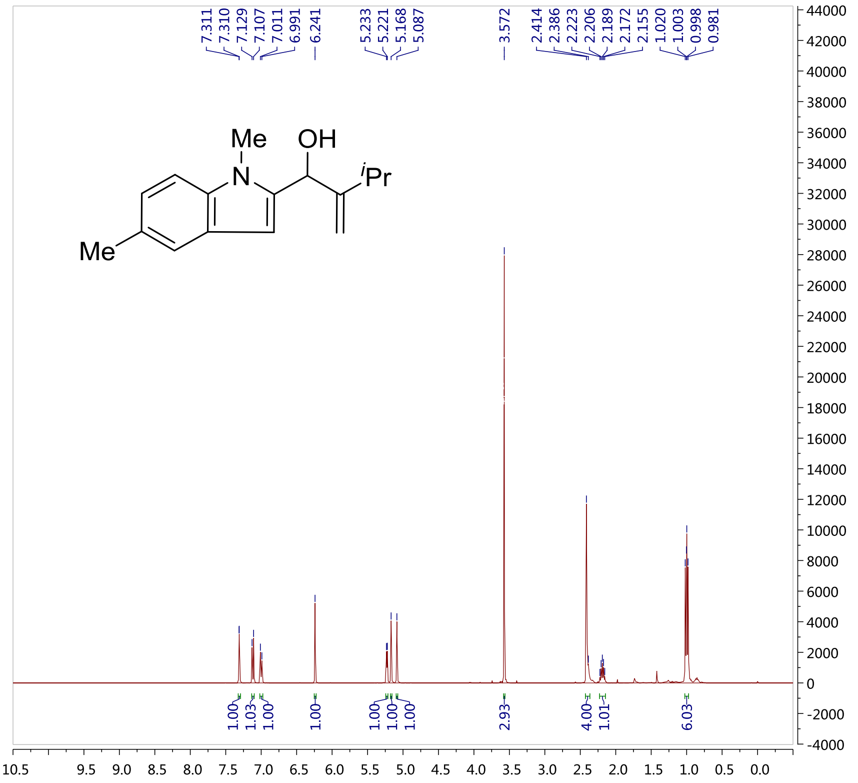

	Parameter	Value
1	Data File Name	E:/ 20160730/ 0 NMR & MS/ 吲哚底物/ wgp-17-7/ 1/ fid
2	Title	wgp-17-7
3	Comment	test
4	Origin	Bruker BioSpin GmbH
5	Owner	nmr
6	Site	
7	Spectrometer	spect
8	Author	
9	Solvent	CDCl3
10	Temperature	294.0
11	Pulse Sequence	zg30
12	Experiment	1D
13	Number of Scans	8
14	Receiver Gain	17
15	Relaxation Delay	1.0000
16	Pulse Width	9.8000
17	Acquisition Time	1.9999
18	Acquisition Date	2016-08-05T14:23:02
19	Modification Date	2016-08-05T14:23:00
20	Spectrometer Frequency	400.13
21	Spectral Width	8012.8
22	Lowest Frequency	-1535.4
23	Nucleus	1H
24	Acquired Size	16025
25	Spectral Size	32768

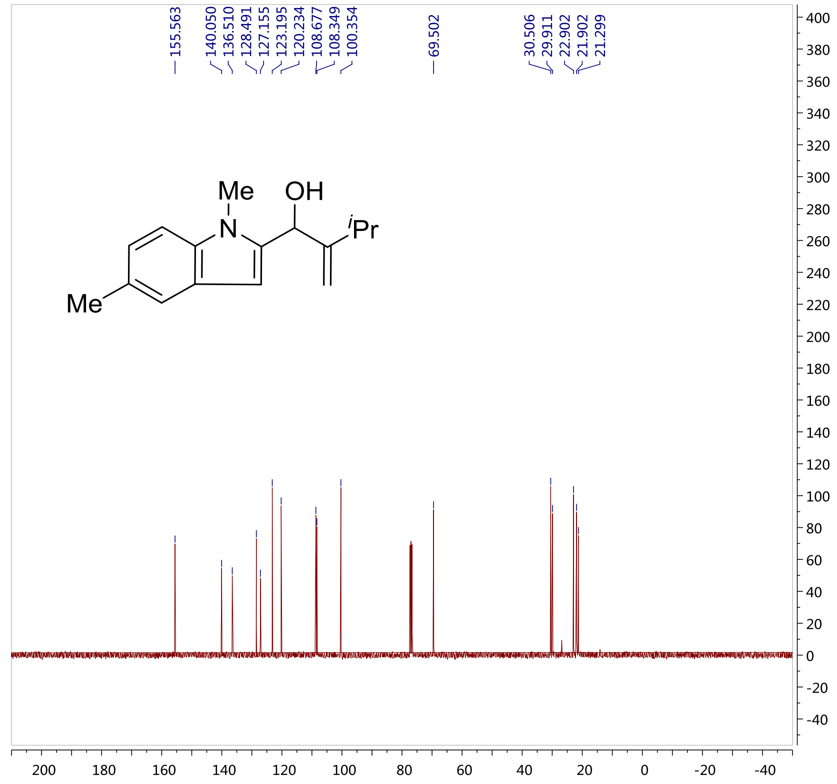

	Parameter	Value
1	Data File Name	E:/ 20160730/ 0 NMR & MS/ 吲哚底物/ wgp-17-7/ 2/ fid
2	Title	wgp-17-7
3	Comment	test
4	Origin	Bruker BioSpin GmbH
5	Owner	nmr
6	Site	
7	Spectrometer	spect
8	Author	
9	Solvent	CDCl3
10	Temperature	294.4
11	Pulse Sequence	zgpg30
12	Experiment	1D
13	Number of Scans	91
14	Receiver Gain	50
15	Relaxation Delay	1.0000
16	Pulse Width	9.8000
17	Acquisition Time	0.8039
18	Acquisition Date	2016-08-05T14:24:43
19	Modification Date	2016-08-05T14:26:00
20	Spectrometer Frequency	100.61
21	Spectral Width	40760.9
22	Lowest Frequency	-10320.1
23	Nucleus	13C
24	Acquired Size	32768
25	Spectral Size	65536

1-(5-fluoro-1-methyl-1H-indol-2-yl)-3-methylbutan-1-one

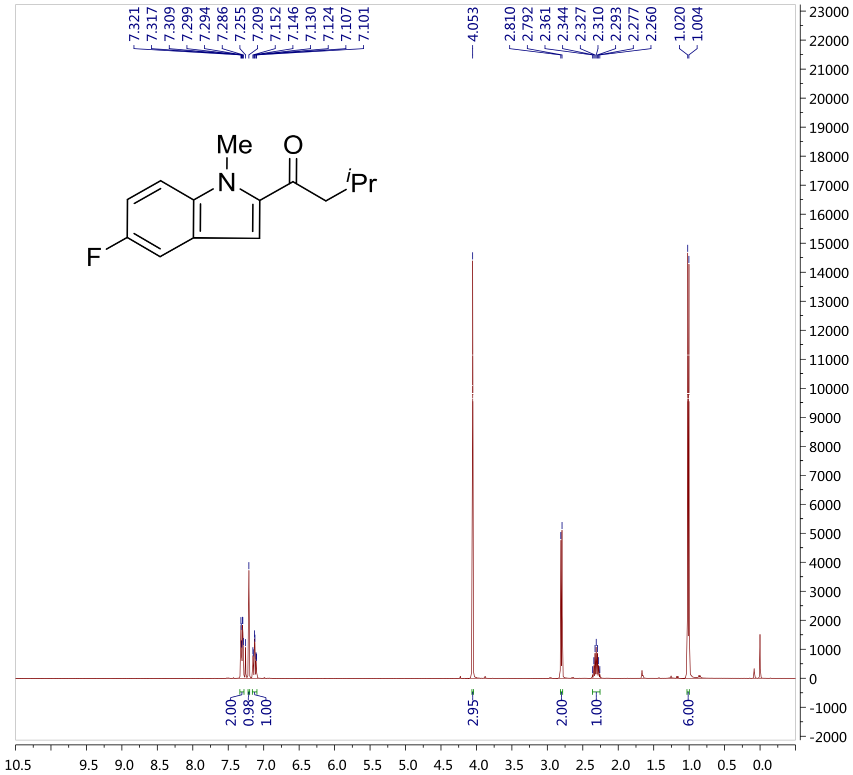

	Parameter	Value
1	Data File Name	E:/ 0 NMR & MS/ 吲哚底物/ wgp-17-29/ 1/ fid
2	Title	wgp-17-29
3	Comment	PROTON
4	Origin	Bruker BioSpin GmbH
5	Owner	common
6	Site	
7	Spectrometer	spect
8	Author	
9	Solvent	CDCl3
10	Temperature	1687.1
11	Pulse Sequence	zg30
12	Experiment	1D
13	Number of Scans	8
14	Receiver Gain	81
15	Relaxation Delay	1.0000
16	Pulse Width	13.7000
17	Acquisition Time	1.9999
18	Acquisition Date	2016-08-26T20:44:00
19	Modification Date	2016-08-26T20:44:00
20	Spectrometer Frequency	400.13
21	Spectral Width	8223.7
22	Lowest Frequency	-1640.9
23	Nucleus	1H
24	Acquired Size	16446
25	Spectral Size	65536

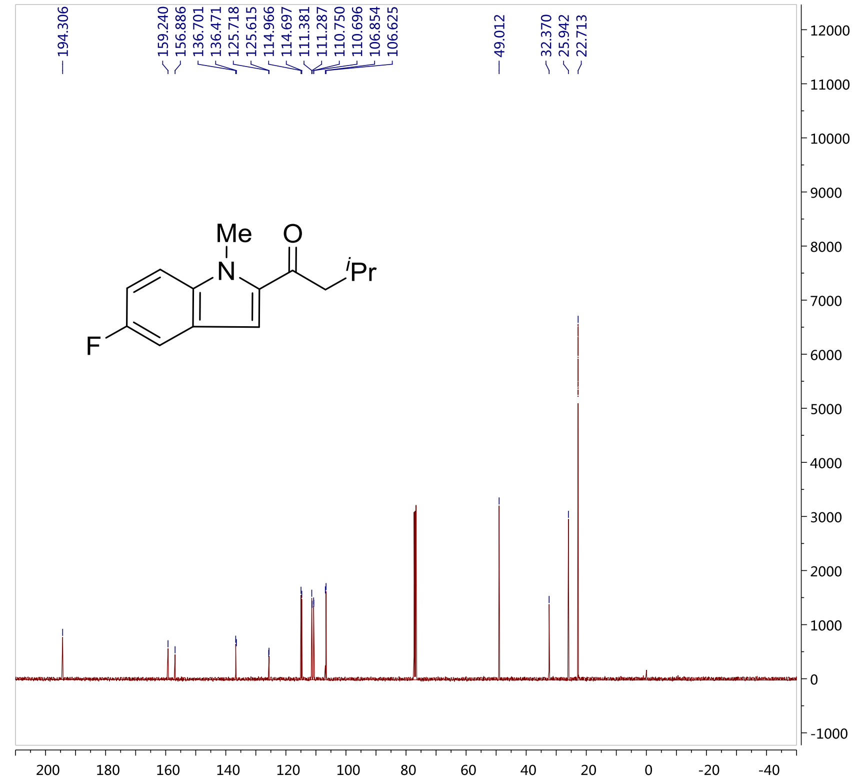

	Parameter	Value
1	Data File Name	E:/ 0 NMR & MS/ 吲哚底物/ wgp-17-29/ 2/ fid
2	Title	wgp-17-29
3	Comment	C13CPD
4	Origin	Bruker BioSpin GmbH
5	Owner	common
6	Site	
7	Spectrometer	spect
8	Author	
9	Solvent	CDCl3
10	Temperature	1689.3
11	Pulse Sequence	zgpg30
12	Experiment	1D
13	Number of Scans	514
14	Receiver Gain	203
15	Relaxation Delay	1.0000
16	Pulse Width	12.0000
17	Acquisition Time	0.4588
18	Acquisition Date	2016-08-26T20:46:00
19	Modification Date	2016-08-26T20:58:00
20	Spectrometer Frequency	100.61
21	Spectral Width	35714.3
22	Lowest Frequency	-7795.9
23	Nucleus	13C
24	Acquired Size	16384
25	Spectral Size	32768

1-(5-chloro-1-methyl-1H-indol-2-yl)-3-methylbutan-1-one

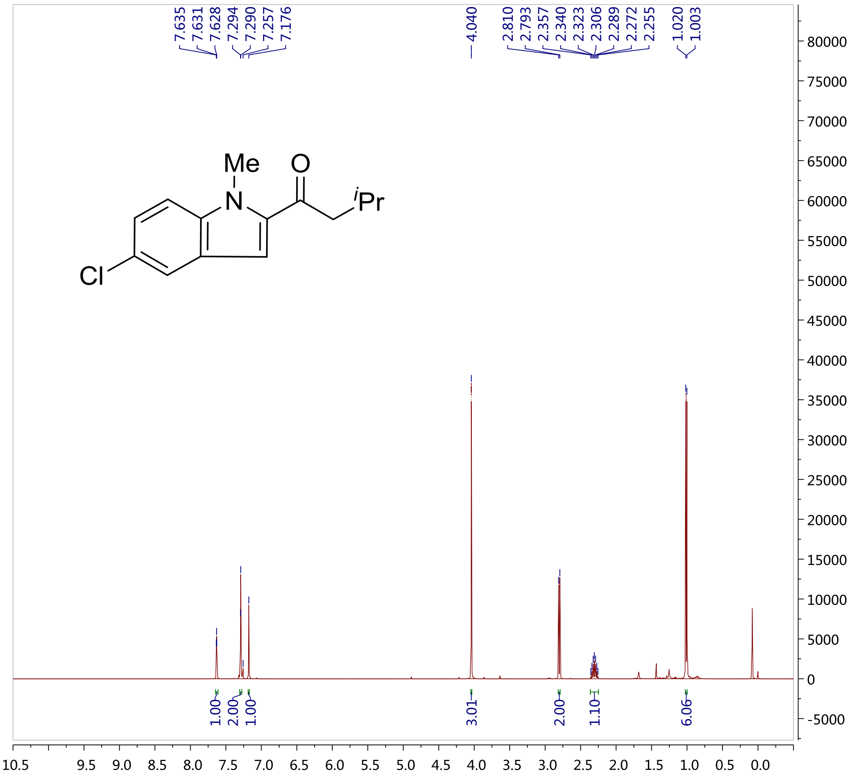

Parameter		Value
1	Data File Name	E:/ 0 NMR & MS/ 1 吲哚底物/ wgp-17-47-1/ 1/ fid
2	Title	wgp-17-47-1
3	Comment	test
4	Origin	Bruker BioSpin GmbH
5	Owner	nmr
6	Site	
7	Spectrometer	spect
8	Author	
9	Solvent	CDCl3
10	Temperature	294.0
11	Pulse Sequence	zg30
12	Experiment	1D
13	Number of Scans	8
14	Receiver Gain	55
15	Relaxation Delay	1.0000
16	Pulse Width	9.8000
17	Acquisition Time	1.9999
18	Acquisition Date	2016-09-15T09:47:28
19	Modification Date	2016-09-15T09:47:00
20	Spectrometer Frequency	400.13
21	Spectral Width	8012.8
22	Lowest Frequency	-1535.4
23	Nucleus	1H
24	Acquired Size	16025
25	Spectral Size	32768

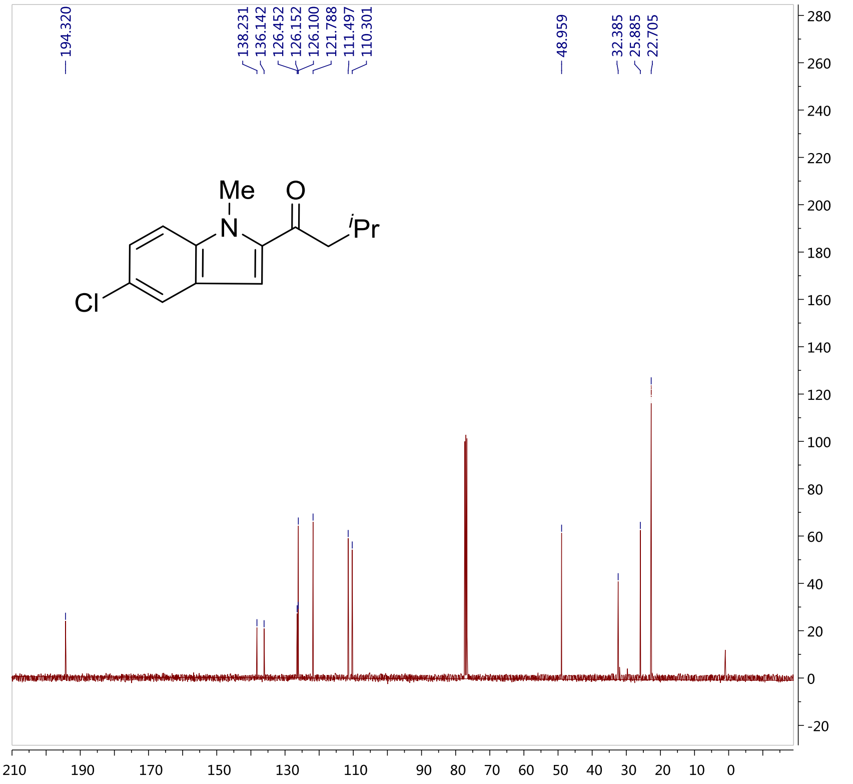

Parameter		Value
1	Data File Name	E:/ 0 NMR & MS/ 1 吲哚底物/ wgp-17-47-1/ 2/ fid
2	Title	wgp-17-47-1
3	Comment	C13CPD
4	Origin	Bruker BioSpin GmbH
5	Owner	nmr
6	Site	
7	Spectrometer	spect
8	Author	
9	Solvent	CDCl3
10	Temperature	293.2
11	Pulse Sequence	zgpg30
12	Experiment	1D
13	Number of Scans	76
14	Receiver Gain	29
15	Relaxation Delay	2.0000
16	Pulse Width	9.4000
17	Acquisition Time	1.3631
18	Acquisition Date	2016-09-15T10:21:06
19	Modification Date	2016-09-15T10:23:00
20	Spectrometer Frequency	100.61
21	Spectral Width	24038.5
22	Lowest Frequency	-2065.9
23	Nucleus	13C
24	Acquired Size	32768
25	Spectral Size	65536

1-(4-fluoro-1-methyl-1H-indol-2-yl)-3-methylbutan-1-one

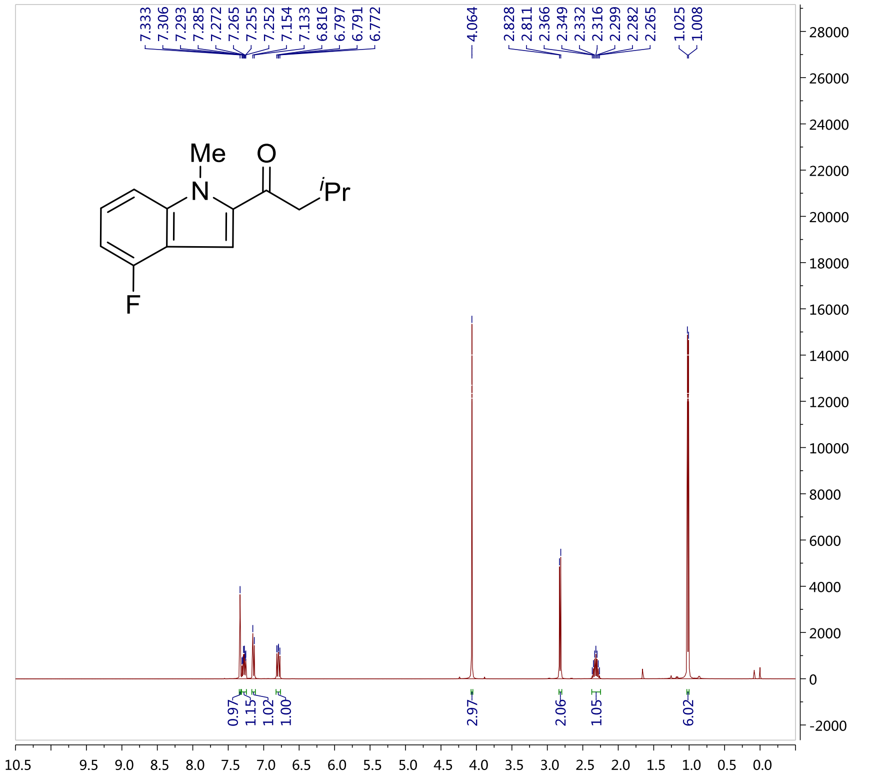

	Parameter	Value
1	Data File Name	E:/ 0 Data/ 1 吲哚底物/ wgp-17-58/ 1/ fid
2	Title	wgp-17-58
3	Comment	PROTON
4	Origin	Bruker BioSpin GmbH
5	Owner	nmr
6	Site	
7	Spectrometer	spect
8	Author	
9	Solvent	CDCl3
10	Temperature	295.8
11	Pulse Sequence	zg30
12	Experiment	1D
13	Number of Scans	8
14	Receiver Gain	71
15	Relaxation Delay	1.0000
16	Pulse Width	15.0000
17	Acquisition Time	1.9999
18	Acquisition Date	2016-09-26T16:53:00
19	Modification Date	2016-09-26T21:10:00
20	Spectrometer Frequency	400.13
21	Spectral Width	8012.8
22	Lowest Frequency	-1584.2
23	Nucleus	1H
24	Acquired Size	20031
25	Spectral Size	65536

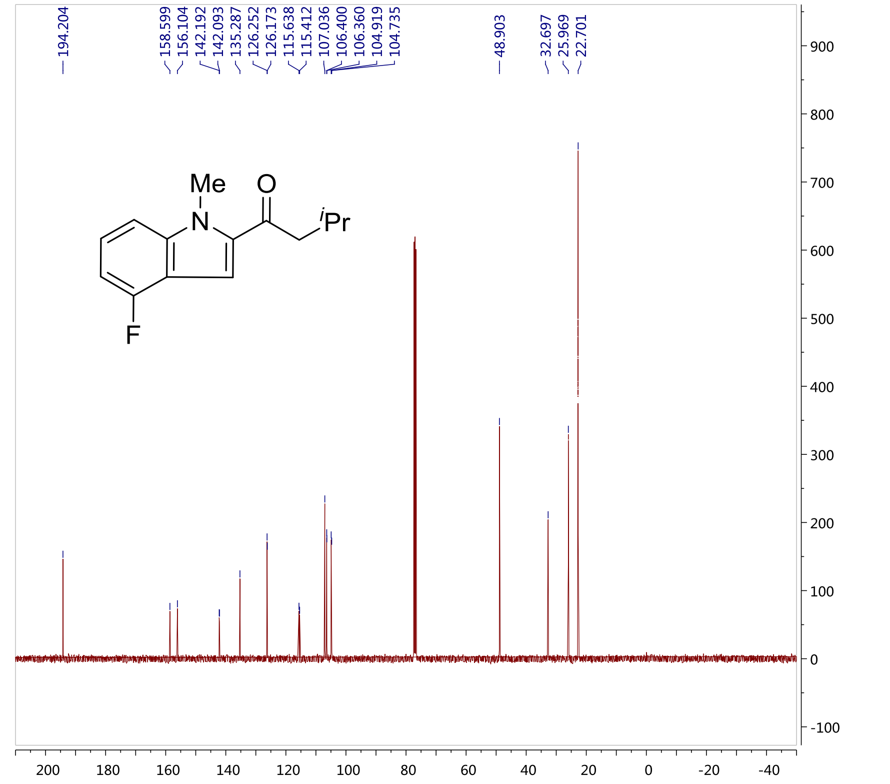

	Parameter	Value
1	Data File Name	E:/ 0 Data/ 1 吲哚底物/ wgp-17-58/ 2/ fid
2	Title	wgp-17-58
3	Comment	test
4	Origin	Bruker BioSpin GmbH
5	Owner	nmr
6	Site	
7	Spectrometer	spect
8	Author	
9	Solvent	CDCl3
10	Temperature	294.1
11	Pulse Sequence	zgpg30
12	Experiment	1D
13	Number of Scans	761
14	Receiver Gain	50
15	Relaxation Delay	1.0000
16	Pulse Width	9.8000
17	Acquisition Time	0.8039
18	Acquisition Date	2016-09-26T19:35:55
19	Modification Date	2016-09-26T19:58:00
20	Spectrometer Frequency	100.61
21	Spectral Width	40760.9
22	Lowest Frequency	-10320.1
23	Nucleus	13C
24	Acquired Size	32768
25	Spectral Size	65536

2-methyl-1-(1-methyl-1H-indol-2-yl)prop-2-en-1-one (2a)

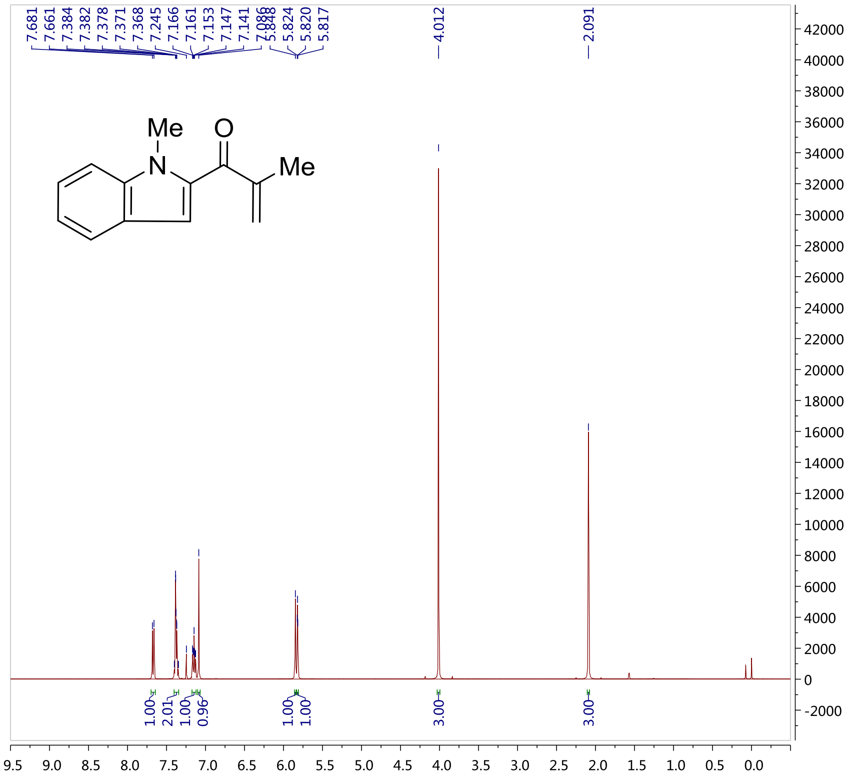

Parameters	
Parameter	Value
Title	wgp-14-180
Comment	test
Origin	Bruker BioSpin GmbH
Owner	nmr
Site	
Spectrometer	spect
Author	
Solvent	CDCl3
Temperature	302.2
Pulse Sequence	zg30
Experiment	1D
Number of Scans	8
Receiver Gain	111
Relaxation Delay	1.0000
Pulse Width	9.8000
Acquisition Time	1.9999
Acquisition Date	2015-03-16T20:44:05
Modification Date	2015-03-16T20:44:00
Spectrometer Frequency	400.13
Spectral Width	8012.8
Lowest Frequency	-1535.4
Nucleus	1H
Acquired Size	16025
Spectral Size	32768

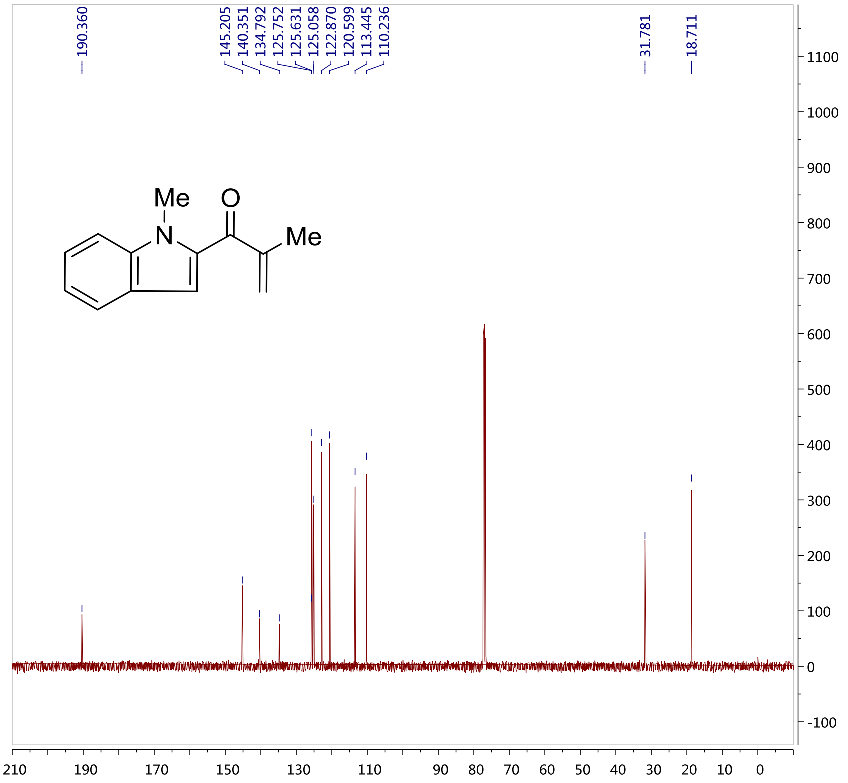

Parameters	
Parameter	Value
Title	wgp-14-180
Comment	test
Origin	Bruker BioSpin GmbH
Owner	nmr
Site	
Spectrometer	spect
Author	
Solvent	CDCl3
Temperature	302.6
Pulse Sequence	zgpg30
Experiment	1D
Number of Scans	266
Receiver Gain	111
Relaxation Delay	1.0000
Pulse Width	9.8000
Acquisition Time	0.8039
Acquisition Date	2015-03-16T20:45:50
Modification Date	2015-03-16T20:53:00
Spectrometer Frequency	100.61
Spectral Width	40760.9
Lowest Frequency	-10320.1
Nucleus	13C
Acquired Size	32768
Spectral Size	65536

1-(1-isopropyl-1H-indol-2-yl)-2-methylprop-2-en-1-one (2b)

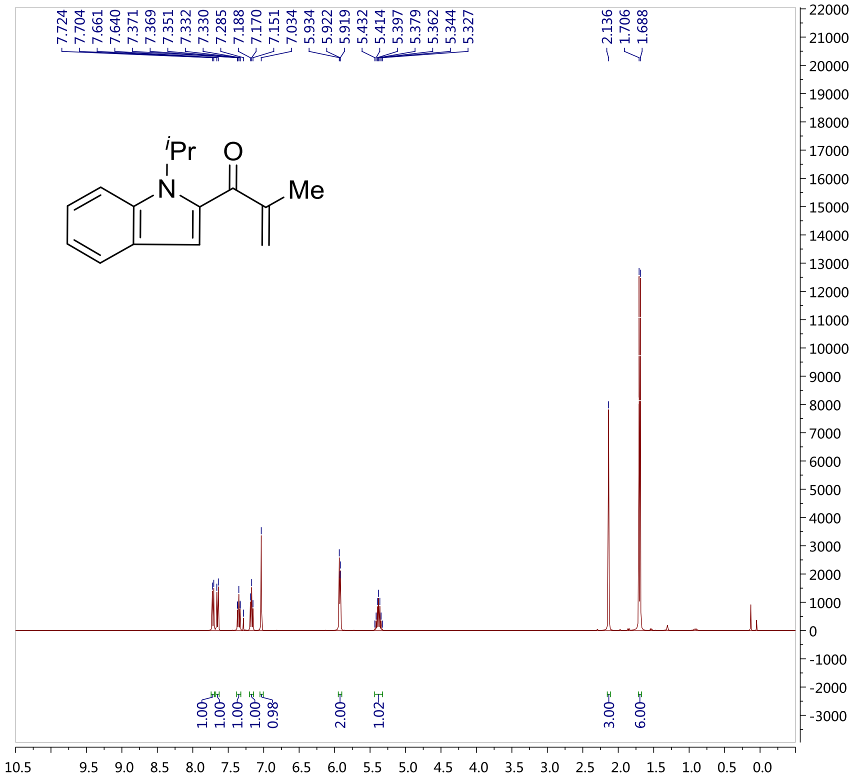

Parameters	
Parameter	Value
Title	wgp-16-88
Comment	PROTON
Origin	Bruker BioSpin GmbH
Owner	nmr
Site	
Spectrometer	spect
Author	
Solvent	CDCl3
Temperature	291.6
Pulse Sequence	zg30
Experiment	1D
Number of Scans	8
Receiver Gain	47
Relaxation Delay	1.0000
Pulse Width	9.1200
Acquisition Time	4.0894
Acquisition Date	2016-03-25T20:14:51
Modification Date	2016-03-25T20:14:00
Spectrometer Frequency	400.13
Spectral Width	8012.8
Lowest Frequency	-1535.4
Nucleus	1H
Acquired Size	32768
Spectral Size	65536

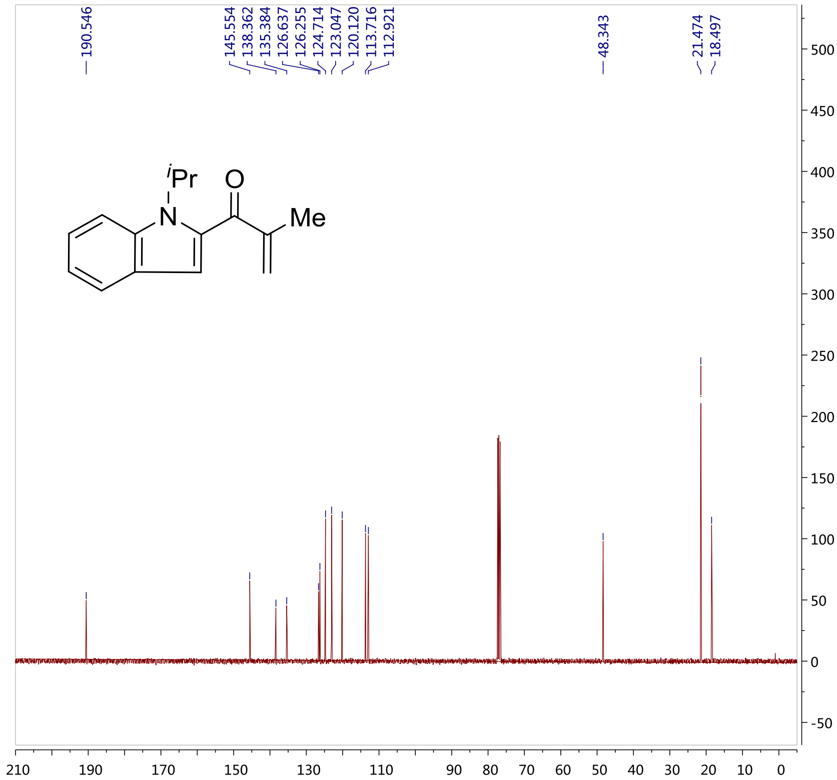

Parameters	
Parameter	Value
Title	wgp-16-88
Comment	C13CPD
Origin	Bruker BioSpin GmbH
Owner	nmr
Site	
Spectrometer	spect
Author	
Solvent	CDCl3
Temperature	292.4
Pulse Sequence	zgpg30
Experiment	1D
Number of Scans	133
Receiver Gain	33
Relaxation Delay	2.0000
Pulse Width	9.4000
Acquisition Time	1.3631
Acquisition Date	2016-03-25T20:18:12
Modification Date	2016-03-25T20:23:00
Spectrometer Frequency	100.61
Spectral Width	24038.5
Lowest Frequency	-1958.9
Nucleus	13C
Acquired Size	32768
Spectral Size	65536

1-(1-allyl-1H-indol-2-yl)-2-methylprop-2-en-1-one (2c)

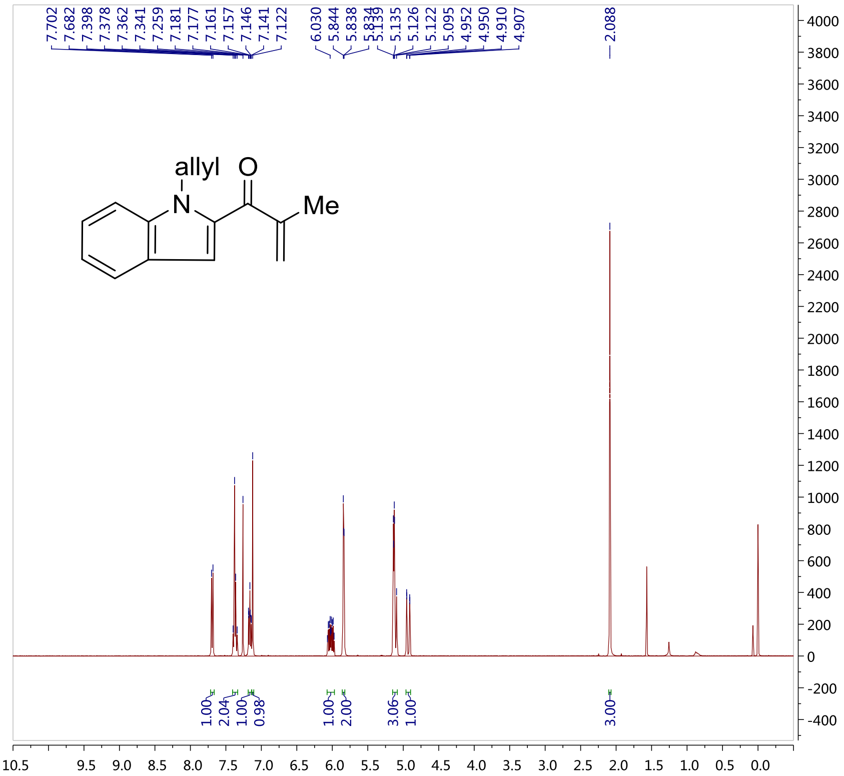

Parameters	
Parameter	Value
Title	wgp-16-87
Comment	PROTON
Origin	Bruker BioSpin GmbH
Owner	nmr
Site	
Spectrometer	spect
Author	
Solvent	CDCl3
Temperature	291.7
Pulse Sequence	zg30
Experiment	1D
Number of Scans	8
Receiver Gain	97
Relaxation Delay	1.0000
Pulse Width	9.1200
Acquisition Time	4.0894
Acquisition Date	2016-03-25T20:10:34
Modification Date	2016-03-25T20:10:00
Spectrometer Frequency	400.13
Spectral Width	8012.8
Lowest Frequency	-1535.4
Nucleus	1H
Acquired Size	32768
Spectral Size	65536

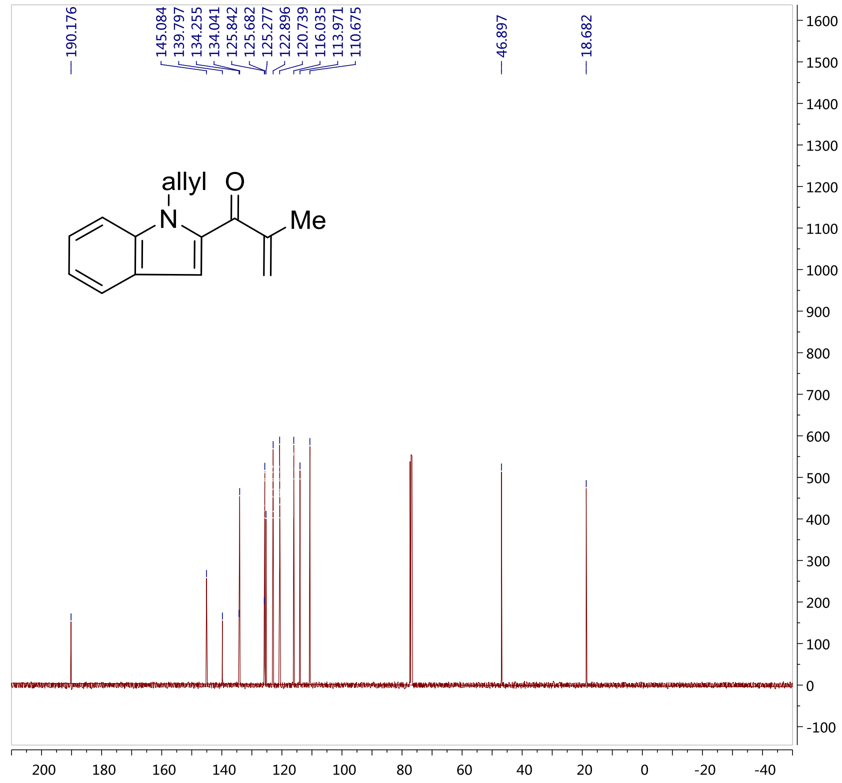

Parameters	
Parameter	Value
Title	wgp-16-87
Comment	test
Origin	Bruker BioSpin GmbH
Owner	nmr
Site	
Spectrometer	spect
Author	
Solvent	CDCl3
Temperature	293.9
Pulse Sequence	zgpg30
Experiment	1D
Number of Scans	203
Receiver Gain	97
Relaxation Delay	1.0000
Pulse Width	9.8000
Acquisition Time	0.8039
Acquisition Date	2016-03-25T16:10:31
Modification Date	2016-03-25T16:15:00
Spectrometer Frequency	100.61
Spectral Width	40760.9
Lowest Frequency	-10320.1
Nucleus	13C
Acquired Size	32768
Spectral Size	65536

1-(1-benzyl-1H-indol-2-yl)-2-methylprop-2-en-1-one (2d)

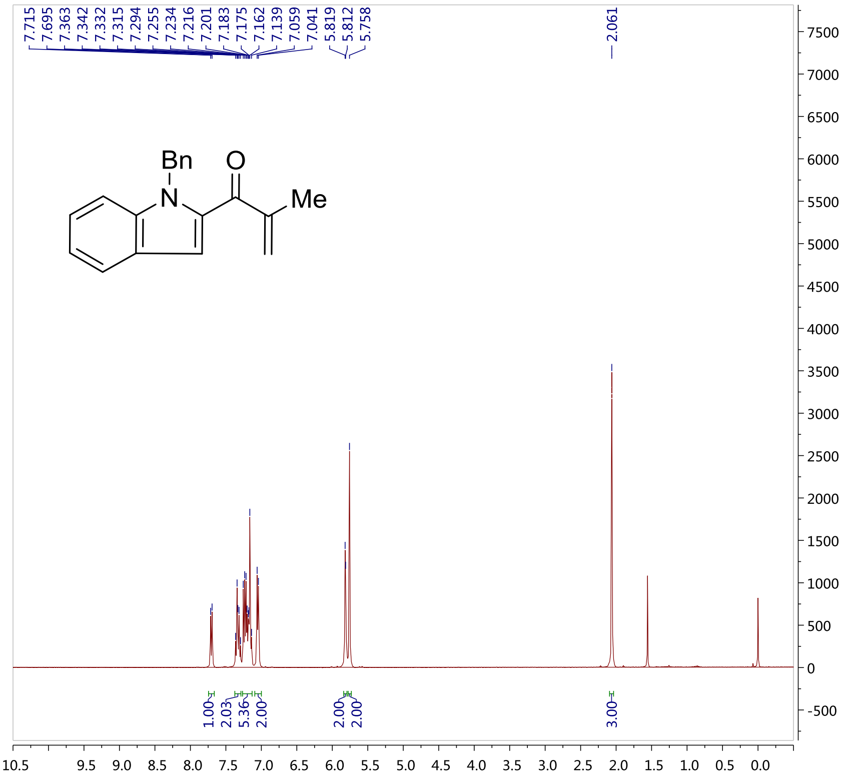

Parameters	
Parameter	Value
Title	wgp-14-18(150402)
Comment	PROTON
Origin	Bruker BioSpin GmbH
Owner	nmr
Site	
Spectrometer	spect
Author	
Solvent	CDCl3
Temperature	294.4
Pulse Sequence	zg30
Experiment	1D
Number of Scans	8
Receiver Gain	203
Relaxation Delay	1.0000
Pulse Width	13.7000
Acquisition Time	1.9999
Acquisition Date	2015-04-02T20:52:00
Modification Date	2015-04-02T20:52:00
Spectrometer Frequency	400.13
Spectral Width	8223.7
Lowest Frequency	-1640.9
Nucleus	1H
Acquired Size	16446
Spectral Size	65536

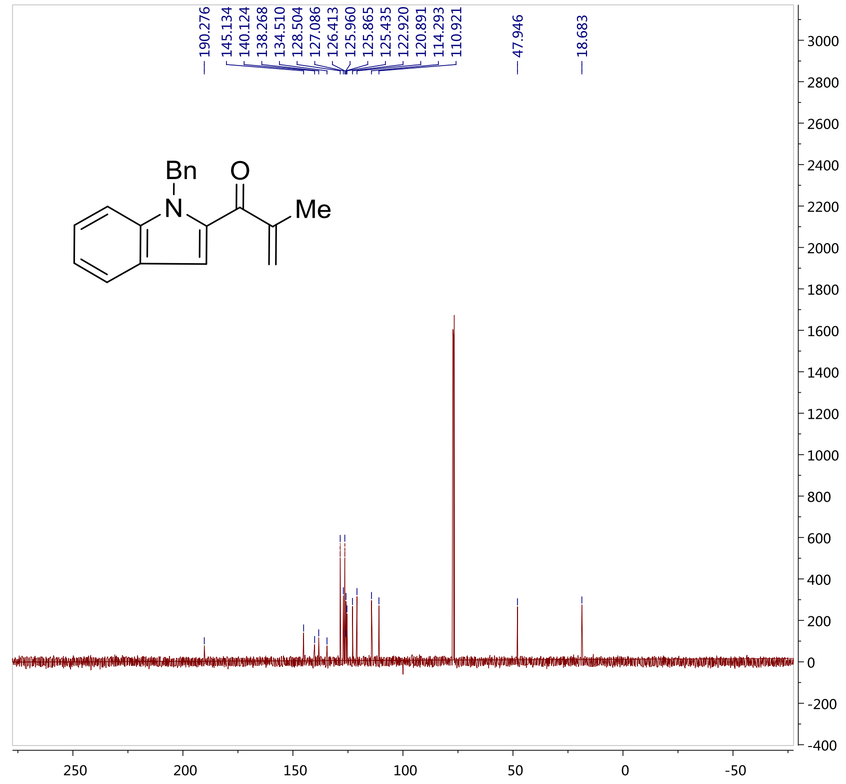

Parameters	
Parameter	Value
Title	wgp-14-18(150402)
Comment	C13CPD
Origin	Bruker BioSpin GmbH
Owner	nmr
Site	
Spectrometer	spect
Author	
Solvent	CDCl3
Temperature	295.0
Pulse Sequence	zgpg30
Experiment	1D
Number of Scans	264
Receiver Gain	203
Relaxation Delay	1.0000
Pulse Width	12.0000
Acquisition Time	0.4588
Acquisition Date	2015-04-02T20:54:00
Modification Date	2015-04-02T21:00:00
Spectrometer Frequency	100.61
Spectral Width	35714.3
Lowest Frequency	-7796.3
Nucleus	13C
Acquired Size	16384
Spectral Size	32768

2-methyl-1-(1-phenyl-1H-indol-2-yl)prop-2-en-1-one (2e)

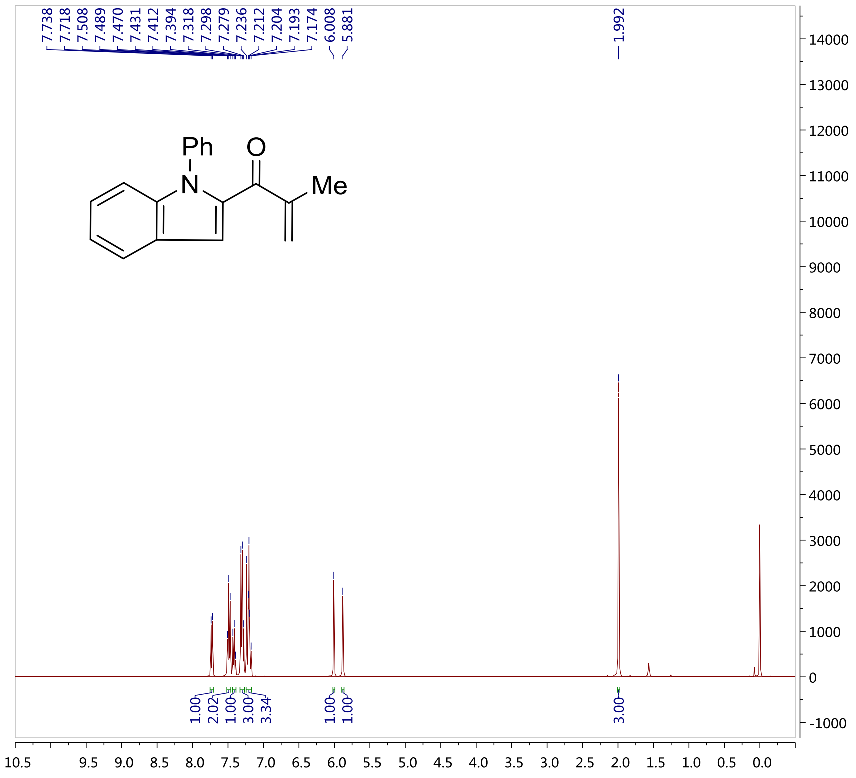

Parameters	
Parameter	Value
Title	wgp-15-119
Comment	PROTON
Origin	Bruker BioSpin GmbH
Owner	common
Site	
Spectrometer	spect
Author	
Solvent	CDCl3
Temperature	295.5
Pulse Sequence	zg30
Experiment	1D
Number of Scans	8
Receiver Gain	114
Relaxation Delay	1.0000
Pulse Width	13.7000
Acquisition Time	1.9999
Acquisition Date	2015-09-02T15:04:00
Modification Date	2015-09-02T15:04:00
Spectrometer Frequency	400.13
Spectral Width	8223.7
Lowest Frequency	-1640.9
Nucleus	1H
Acquired Size	16446
Spectral Size	65536

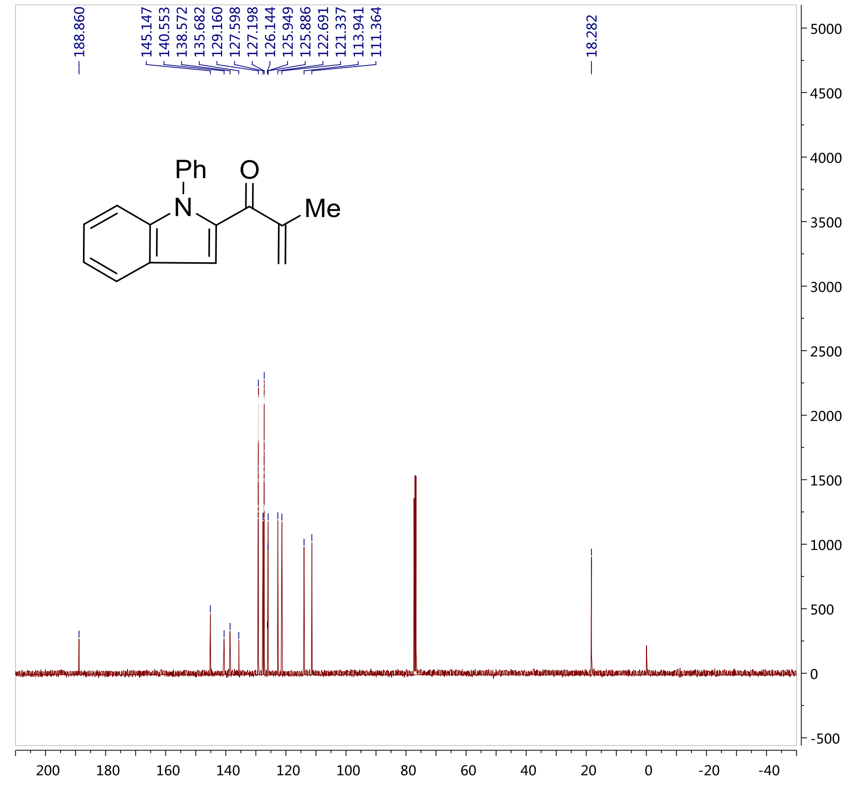

Parameters	
Parameter	Value
Title	wgp-15-119
Comment	C13CPD
Origin	Bruker BioSpin GmbH
Owner	common
Site	
Spectrometer	spect
Author	
Solvent	CDCl3
Temperature	296.0
Pulse Sequence	zgpg30
Experiment	1D
Number of Scans	263
Receiver Gain	203
Relaxation Delay	1.0000
Pulse Width	12.0000
Acquisition Time	0.4588
Acquisition Date	2015-09-02T15:06:00
Modification Date	2015-09-02T15:12:00
Spectrometer Frequency	100.61
Spectral Width	35714.3
Lowest Frequency	-7801.3
Nucleus	13C
Acquired Size	16384
Spectral Size	32768

1-(1-(4-methoxyphenyl)-1H-indol-2-yl)-2-methylprop-2-en-1-one (2f)

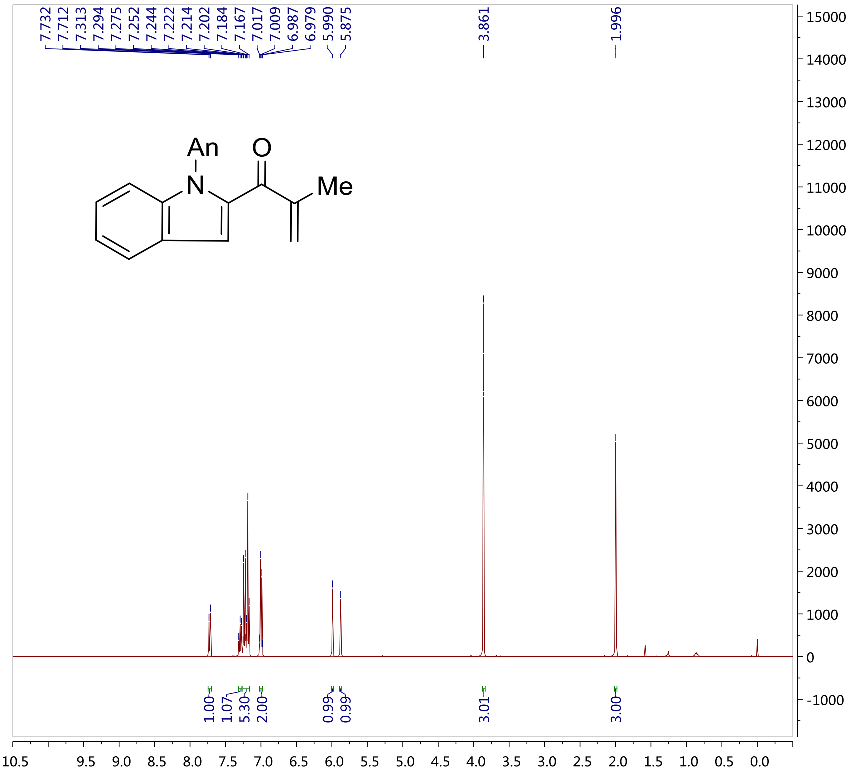

Parameters	
Parameter	Value
Title	wgp-16-77
Comment	PROTON
Origin	Bruker BioSpin GmbH
Owner	nmr
Site	
Spectrometer	spect
Author	
Solvent	CDCl3
Temperature	291.5
Pulse Sequence	zg30
Experiment	1D
Number of Scans	8
Receiver Gain	57
Relaxation Delay	1.0000
Pulse Width	9.1200
Acquisition Time	1.9999
Acquisition Date	2016-03-17T09:09:00
Modification Date	2016-03-17T10:32:00
Spectrometer Frequency	400.13
Spectral Width	8012.8
Lowest Frequency	-1535.4
Nucleus	1H
Acquired Size	32768
Spectral Size	65536

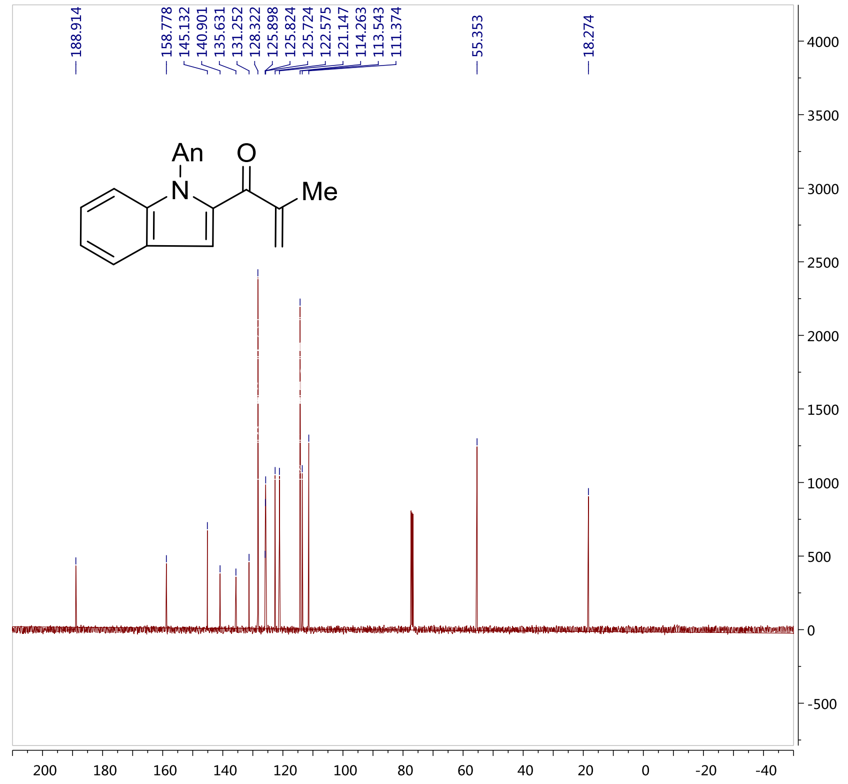

Parameters	
Parameter	Value
Title	wgp-16-77
Comment	C13CPD
Origin	Bruker BioSpin GmbH
Owner	common
Site	
Spectrometer	spect
Author	
Solvent	CDCl3
Temperature	294.7
Pulse Sequence	zgpg30
Experiment	1D
Number of Scans	261
Receiver Gain	203
Relaxation Delay	1.0000
Pulse Width	12.0000
Acquisition Time	0.4588
Acquisition Date	2016-03-17T09:11:00
Modification Date	2016-03-17T09:17:00
Spectrometer Frequency	100.61
Spectral Width	35714.3
Lowest Frequency	-7795.9
Nucleus	13C
Acquired Size	16384
Spectral Size	32768

2-methyl-1-(1-(4-(trifluoromethyl)phenyl)-1H-indol-2-yl)prop-2-en-1-one (2g)

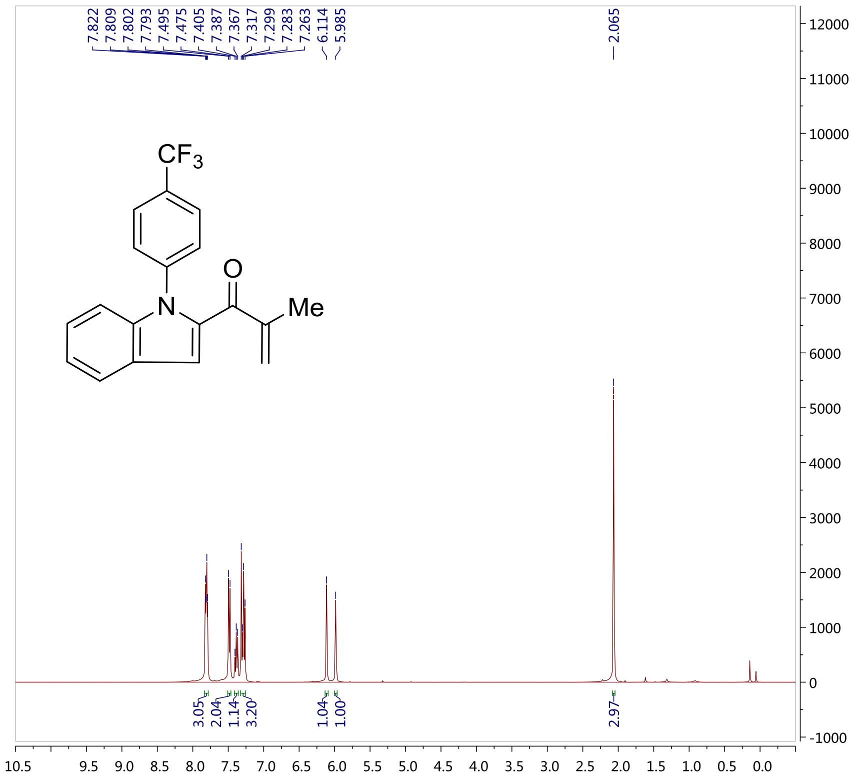

Parameters	
Parameter	Value
Title	wgp-16-76
Comment	PROTON
Origin	Bruker BioSpin GmbH
Owner	nmr
Site	
Spectrometer	spect
Author	
Solvent	CDCl3
Temperature	291.8
Pulse Sequence	zg30
Experiment	1D
Number of Scans	8
Receiver Gain	47
Relaxation Delay	1.0000
Pulse Width	9.1200
Acquisition Time	4.0894
Acquisition Date	2016-03-16T14:31:15
Modification Date	2016-03-16T14:31:00
Spectrometer Frequency	400.13
Spectral Width	8012.8
Lowest Frequency	-1535.4
Nucleus	1H
Acquired Size	32768
Spectral Size	65536

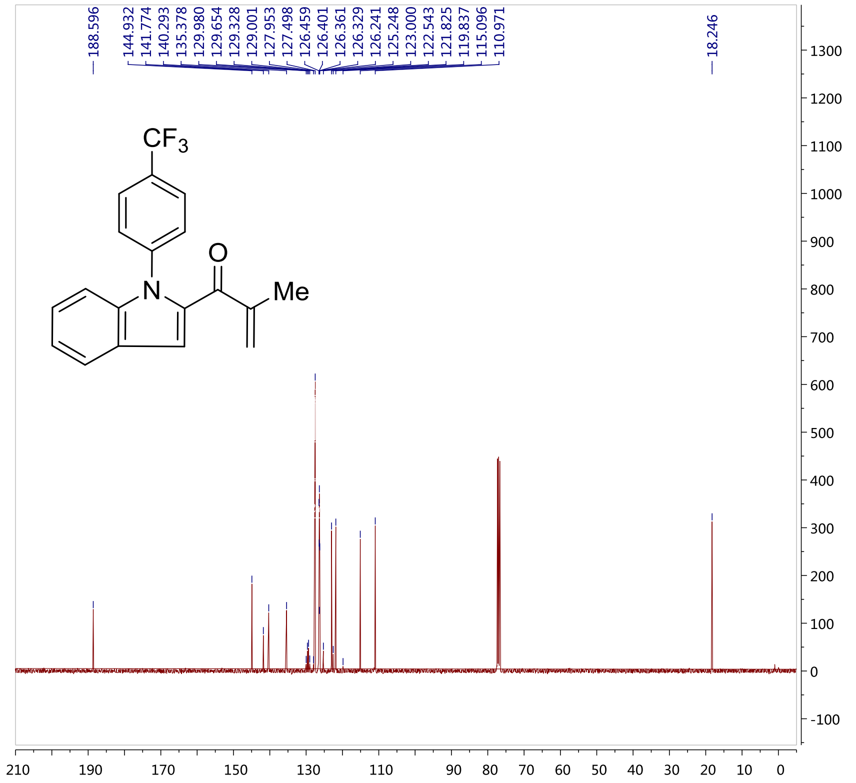

Parameters	
Parameter	Value
Title	wgp-16-76
Comment	C13CPD
Origin	Bruker BioSpin GmbH
Owner	nmr
Site	
Spectrometer	spect
Author	
Solvent	CDCl3
Temperature	292.6
Pulse Sequence	zgpg30
Experiment	1D
Number of Scans	346
Receiver Gain	41
Relaxation Delay	2.0000
Pulse Width	9.4000
Acquisition Time	1.3631
Acquisition Date	2016-03-16T14:34:25
Modification Date	2016-03-16T14:52:00
Spectrometer Frequency	100.61
Spectral Width	24038.5
Lowest Frequency	-1958.9
Nucleus	13C
Acquired Size	32768
Spectral Size	65536

2-methyl-1-(1-(naphthalen-2-yl)-1H-indol-2-yl)prop-2-en-1-one (2h)

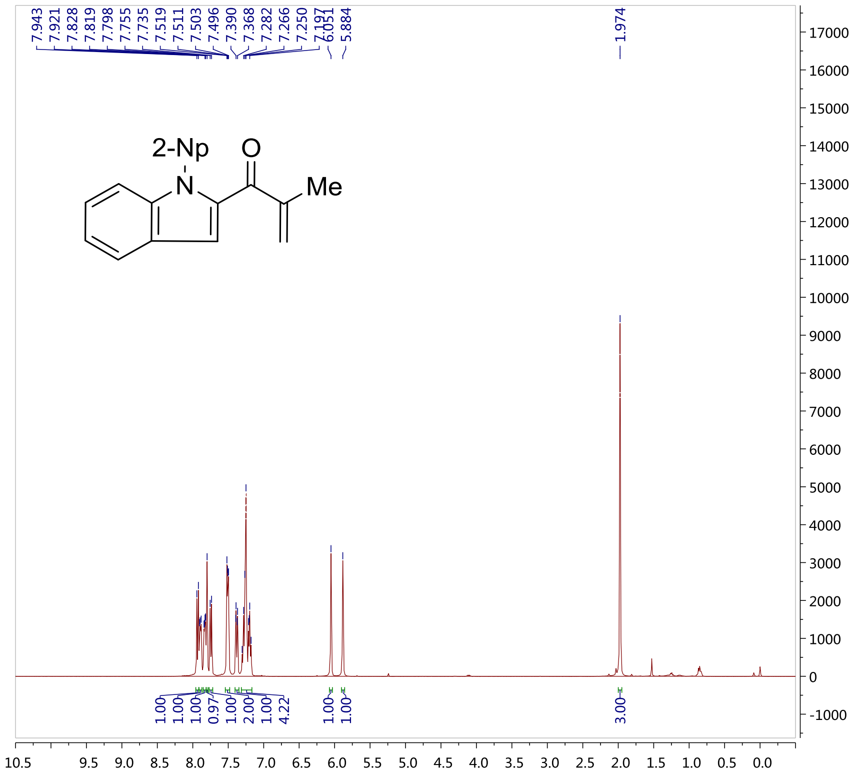

Parameters	
Parameter	Value
Title	wgp-16-101
Comment	test
Origin	Bruker BioSpin GmbH
Owner	nmr
Site	
Spectrometer	spect
Author	
Solvent	CDCl3
Temperature	293.5
Pulse Sequence	zg30
Experiment	1D
Number of Scans	8
Receiver Gain	55
Relaxation Delay	1.0000
Pulse Width	9.8000
Acquisition Time	1.9999
Acquisition Date	2016-04-06T08:53:30
Modification Date	2016-04-06T08:53:00
Spectrometer Frequency	400.13
Spectral Width	8012.8
Lowest Frequency	-1535.4
Nucleus	1H
Acquired Size	16025
Spectral Size	32768

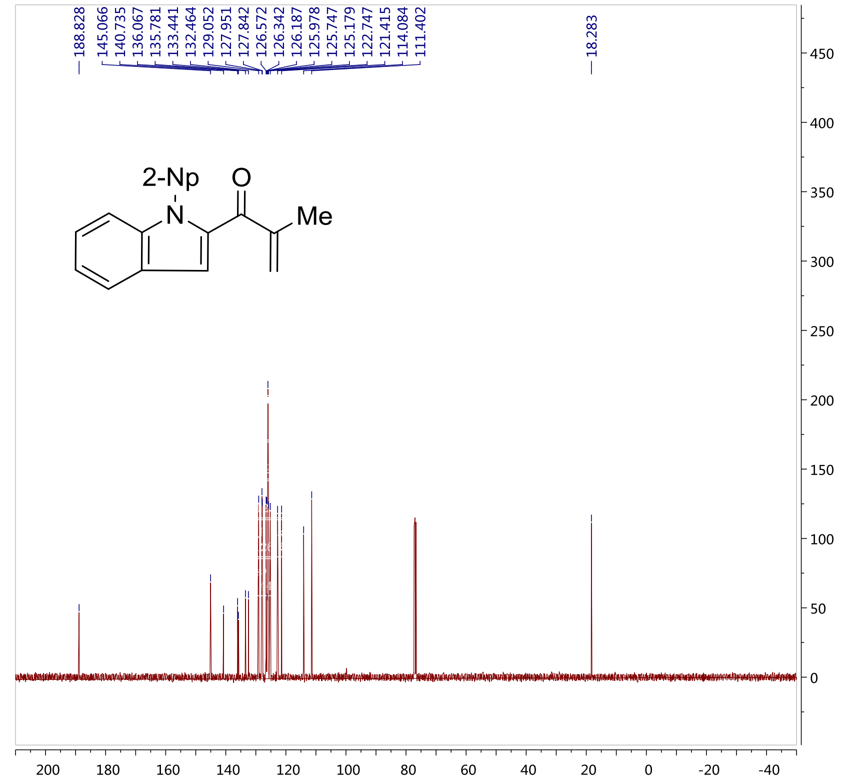

Parameters	
Parameter	Value
Title	wgp-16-101
Comment	test
Origin	Bruker BioSpin GmbH
Owner	nmr
Site	
Spectrometer	spect
Author	
Solvent	CDCl3
Temperature	293.8
Pulse Sequence	zgpg30
Experiment	1D
Number of Scans	134
Receiver Gain	46
Relaxation Delay	1.0000
Pulse Width	9.8000
Acquisition Time	0.8039
Acquisition Date	2016-04-06T08:55:11
Modification Date	2016-04-06T08:58:00
Spectrometer Frequency	100.61
Spectral Width	40760.9
Lowest Frequency	-10320.1
Nucleus	13C
Acquired Size	32768
Spectral Size	65536

1-(1-methyl-1H-indol-2-yl)-2-methylenebutan-1-one (2i)

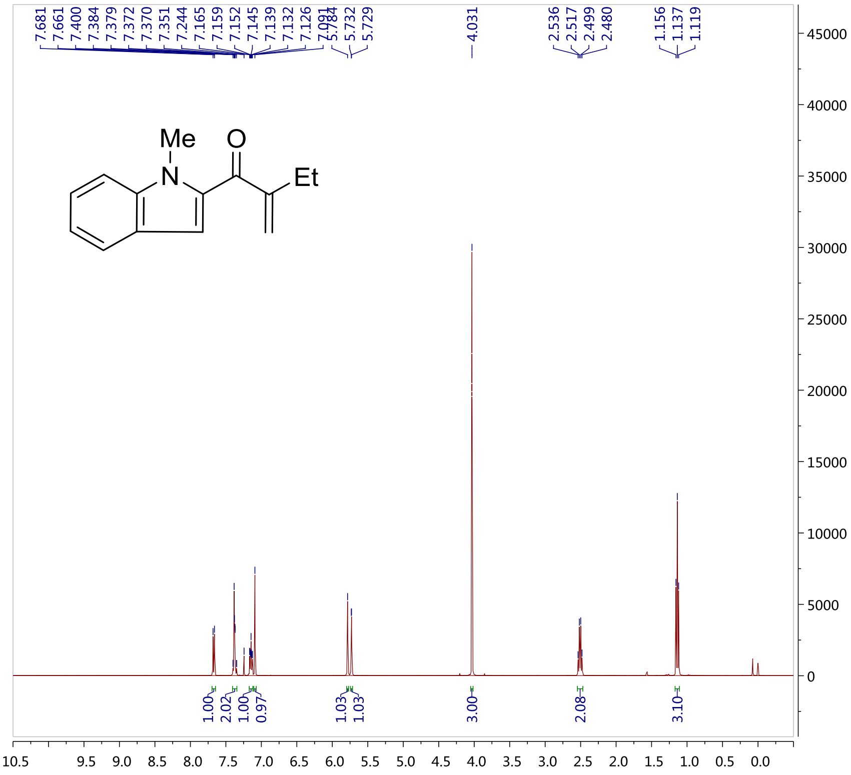

Parameters	
Parameter	Value
Title	wgp-14-186
Comment	test
Origin	Bruker BioSpin GmbH
Owner	nmr
Site	
Spectrometer	spect
Author	
Solvent	CDCl3
Temperature	303.6
Pulse Sequence	zg30
Experiment	1D
Number of Scans	8
Receiver Gain	97
Relaxation Delay	1.0000
Pulse Width	9.8000
Acquisition Time	1.9999
Acquisition Date	2015-03-21T19:19:28
Modification Date	2015-03-21T19:19:00
Spectrometer Frequency	400.13
Spectral Width	8012.8
Lowest Frequency	-1535.4
Nucleus	1H
Acquired Size	16025
Spectral Size	32768

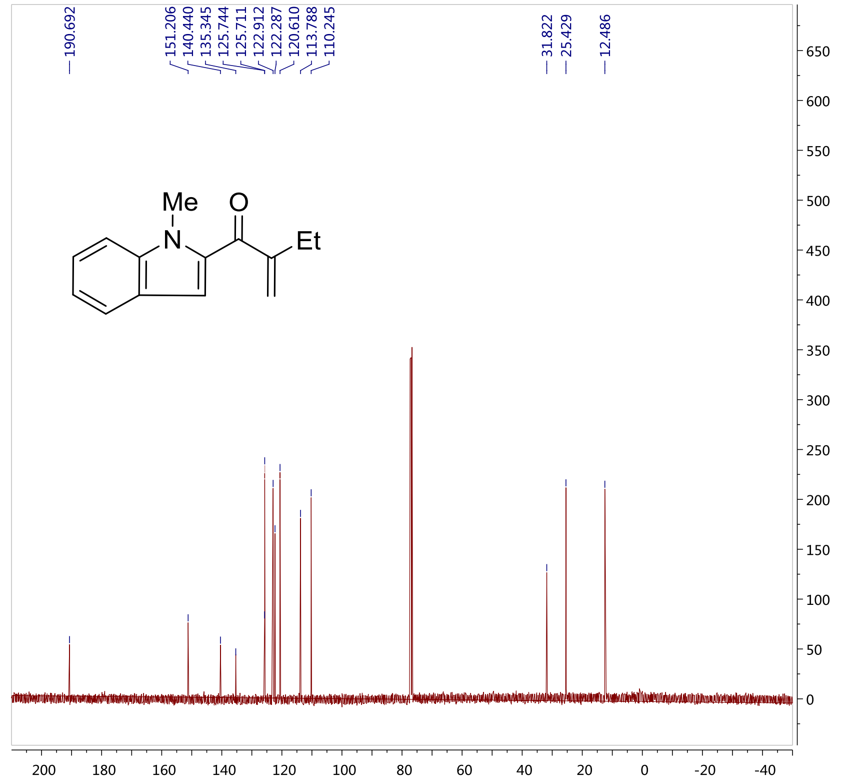

Parameters	
Parameter	Value
Title	wgp-14-186
Comment	test
Origin	Bruker BioSpin GmbH
Owner	nmr
Site	
Spectrometer	spect
Author	
Solvent	CDCl3
Temperature	304.0
Pulse Sequence	zgpg30
Experiment	1D
Number of Scans	208
Receiver Gain	78
Relaxation Delay	1.0000
Pulse Width	9.8000
Acquisition Time	0.8039
Acquisition Date	2015-03-21T19:21:28
Modification Date	2015-03-21T19:27:00
Spectrometer Frequency	100.61
Spectral Width	40760.9
Lowest Frequency	-10320.1
Nucleus	13C
Acquired Size	32768
Spectral Size	65536

3-methyl-1-(1-methyl-1H-indol-2-yl)-2-methylenebutan-1-one (2j)

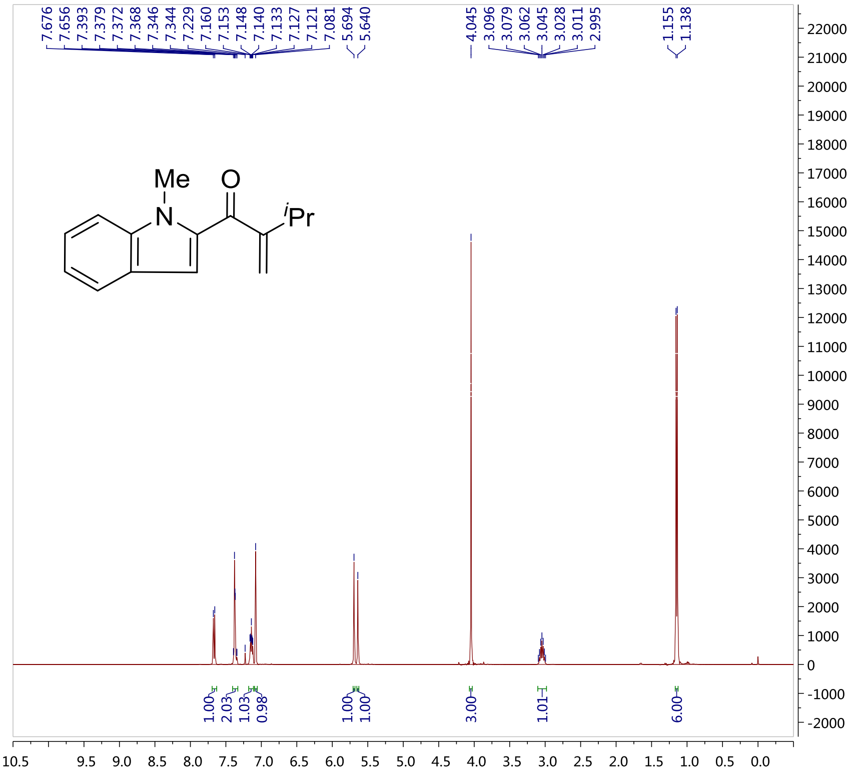

Parameters	
Parameter	Value
Title	wgp-15-188
Comment	PROTON
Origin	Bruker BioSpin GmbH
Owner	common
Site	
Spectrometer	spect
Author	
Solvent	CDCl3
Temperature	297.0
Pulse Sequence	zg30
Experiment	1D
Number of Scans	8
Receiver Gain	64
Relaxation Delay	1.0000
Pulse Width	13.7000
Acquisition Time	1.9999
Acquisition Date	2015-11-23T16:19:00
Modification Date	2015-11-23T16:19:00
Spectrometer Frequency	400.13
Spectral Width	8223.7
Lowest Frequency	-1640.9
Nucleus	1H
Acquired Size	16446
Spectral Size	65536

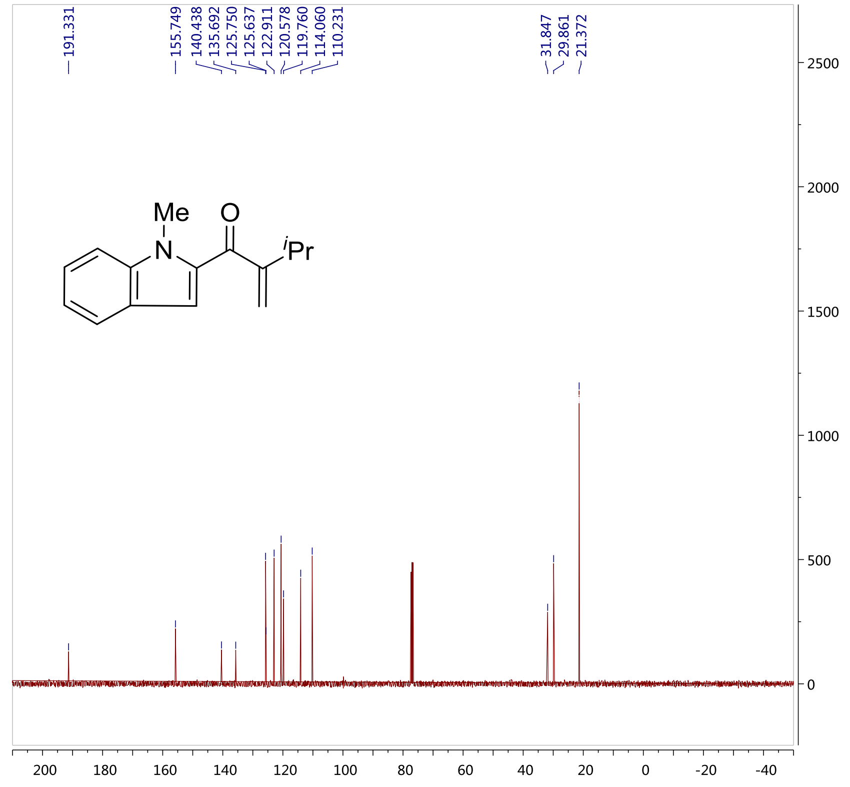

Parameters	
Parameter	Value
Title	wgp-15-188
Comment	C13CPD
Origin	Bruker BioSpin GmbH
Owner	common
Site	
Spectrometer	spect
Author	
Solvent	CDCl3
Temperature	297.5
Pulse Sequence	zgpg30
Experiment	1D
Number of Scans	65
Receiver Gain	203
Relaxation Delay	1.0000
Pulse Width	12.0000
Acquisition Time	0.4588
Acquisition Date	2015-11-23T16:21:00
Modification Date	2015-11-23T16:22:00
Spectrometer Frequency	100.61
Spectral Width	35714.3
Lowest Frequency	-7795.9
Nucleus	13C
Acquired Size	16384
Spectral Size	32768

1-(1-methyl-1H-indol-2-yl)-2-phenylprop-2-en-1-one (2k)

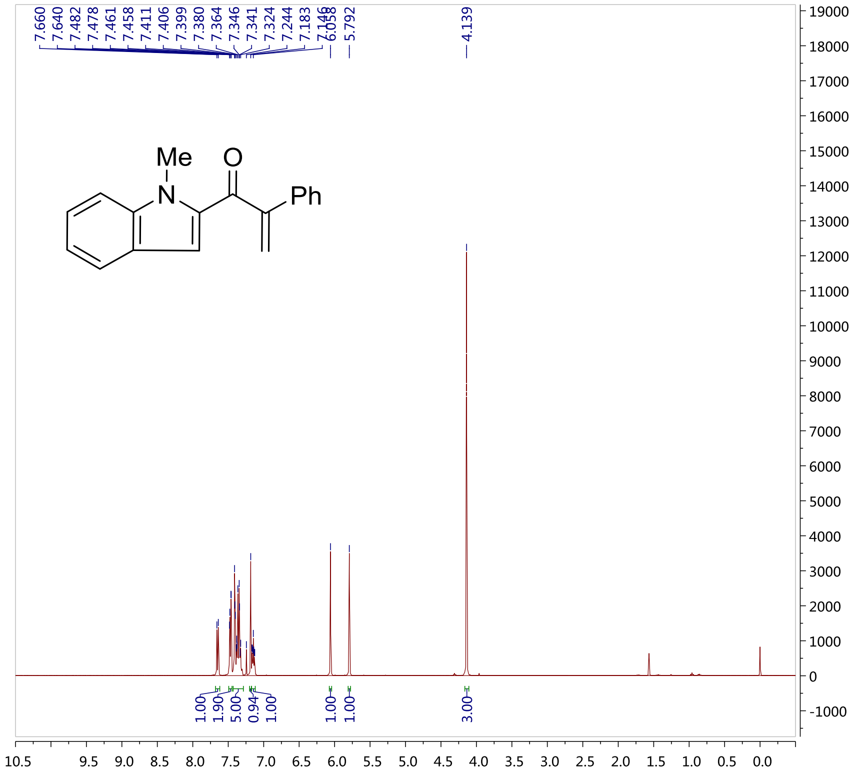

Parameters	
Parameter	Value
Title	wgp-14-9(150401)
Comment	PROTON
Origin	Bruker BioSpin GmbH
Owner	nmr
Site	
Spectrometer	spect
Author	
Solvent	CDCl3
Temperature	294.7
Pulse Sequence	zg30
Experiment	1D
Number of Scans	8
Receiver Gain	144
Relaxation Delay	1.0000
Pulse Width	13.7000
Acquisition Time	1.9999
Acquisition Date	2015-04-01T22:04:00
Modification Date	2015-04-01T22:04:00
Spectrometer Frequency	400.13
Spectral Width	8223.7
Lowest Frequency	-1640.9
Nucleus	1H
Acquired Size	16446
Spectral Size	65536

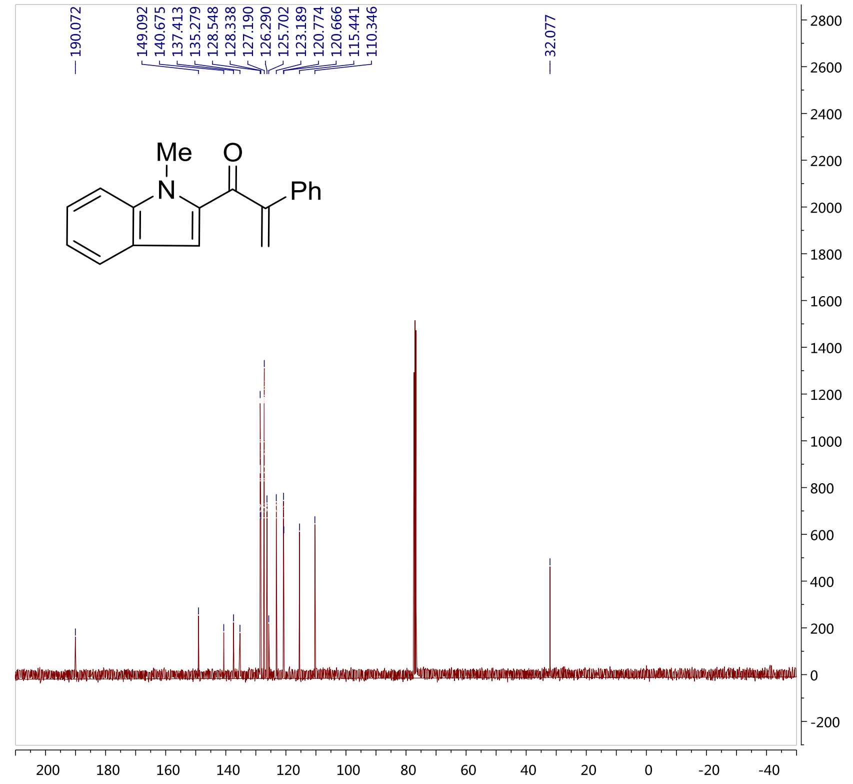

Parameters	
Parameter	Value
Title	wgp-14-9(150401)
Comment	C13CPD
Origin	Bruker BioSpin GmbH
Owner	nmr
Site	
Spectrometer	spect
Author	
Solvent	CDCl3
Temperature	295.4
Pulse Sequence	zgpg30
Experiment	1D
Number of Scans	256
Receiver Gain	203
Relaxation Delay	1.0000
Pulse Width	12.0000
Acquisition Time	0.4588
Acquisition Date	2015-04-01T22:06:00
Modification Date	2015-04-01T22:12:00
Spectrometer Frequency	100.61
Spectral Width	35714.3
Lowest Frequency	-7796.3
Nucleus	13C
Acquired Size	16384
Spectral Size	32768

2-(4-methoxyphenyl)-1-(1-methyl-1H-indol-2-yl)prop-2-en-1-one (2l)

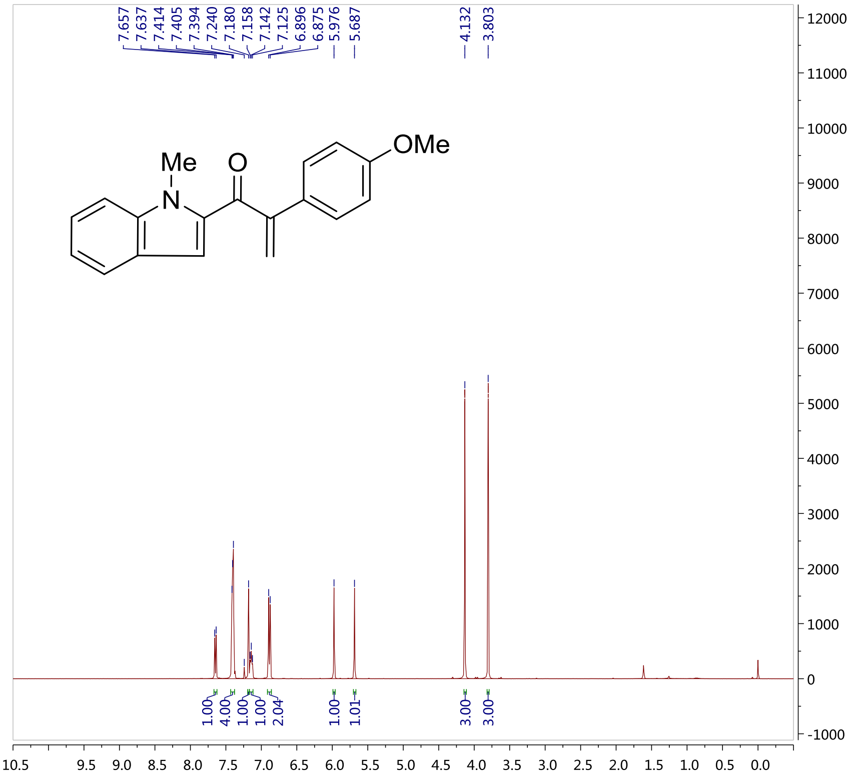

Parameter	Value
1 Data File Name	E:/ 0 NMR & MS/ 吲哚底物/ wgp-17-14/ 1/ fid
2 Title	wgp-17-14
3 Comment	PROTON
4 Origin	Bruker BioSpin GmbH
5 Owner	nmr
6 Site	
7 Spectrometer	spect
8 Author	
9 Solvent	CDCl3
10 Temperature	291.8
11 Pulse Sequence	zg30
12 Experiment	1D
13 Number of Scans	8
14 Receiver Gain	52
15 Relaxation Delay	1.0000
16 Pulse Width	9.1200
17 Acquisition Time	4.0894
18 Acquisition Date	2016-08-12T14:30:33
19 Modification Date	2016-08-12T14:30:00
20 Spectrometer Frequency	400.13
21 Spectral Width	8012.8
22 Lowest Frequency	-1535.4
23 Nucleus	1H
24 Acquired Size	32768
25 Spectral Size	65536

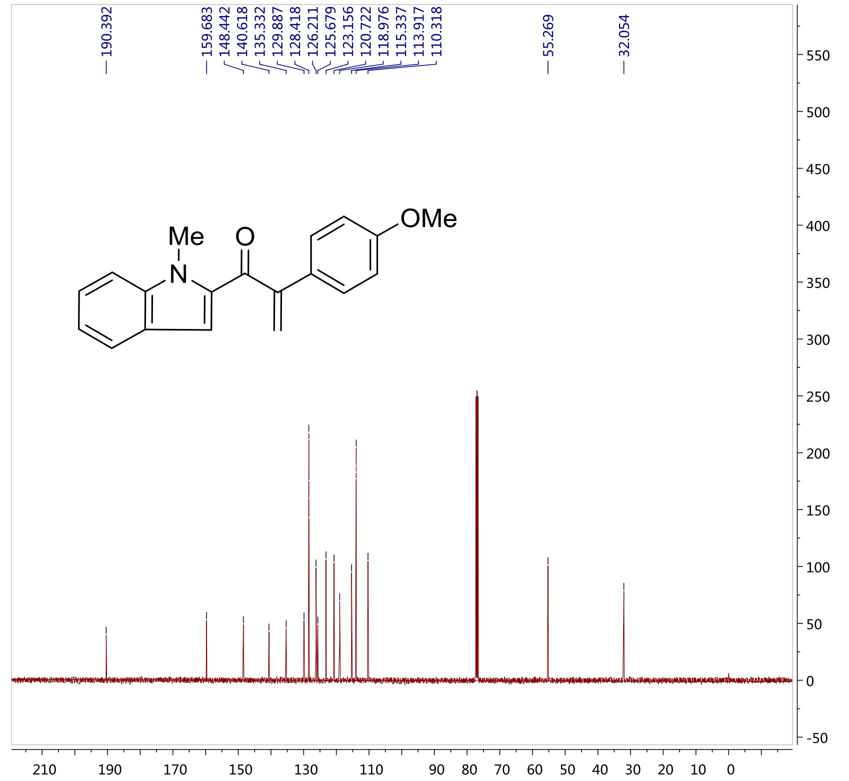

Parameter	Value
1 Data File Name	E:/ 0 NMR & MS/ 吲哚底物/ wgp-17-14/ 2/ fid
2 Title	wgp-17-14
3 Comment	C13CPD
4 Origin	Bruker BioSpin GmbH
5 Owner	nmr
6 Site	
7 Spectrometer	spect
8 Author	
9 Solvent	CDCl3
10 Temperature	292.6
11 Pulse Sequence	zgpg30
12 Experiment	1D
13 Number of Scans	216
14 Receiver Gain	29
15 Relaxation Delay	2.0000
16 Pulse Width	9.4000
17 Acquisition Time	1.3631
18 Acquisition Date	2016-08-12T14:33:40
19 Modification Date	2016-08-12T14:43:00
20 Spectrometer Frequency	100.61
21 Spectral Width	24038.5
22 Lowest Frequency	-1958.9
23 Nucleus	13C
24 Acquired Size	32768
25 Spectral Size	65536

2-(4-fluorophenyl)-1-(1-methyl-1H-indol-2-yl)prop-2-en-1-one (2m)

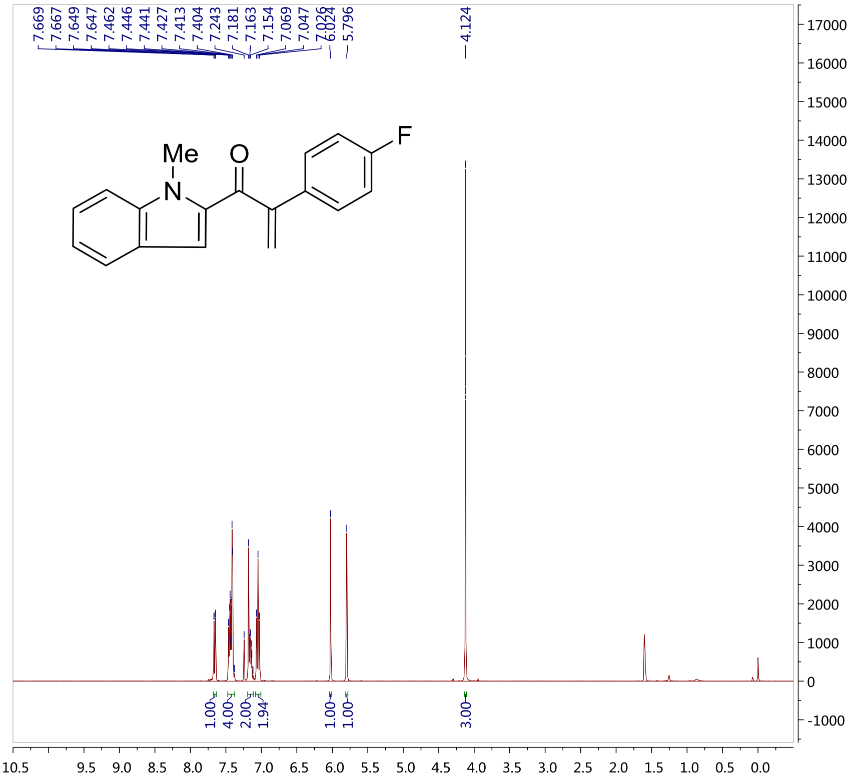

Parameters	
Parameter	Value
Title	wgp-16-192
Comment	PROTON
Origin	Bruker BioSpin GmbH
Owner	common
Site	
Spectrometer	spect
Author	
Solvent	CDCl3
Temperature	-1492.6
Pulse Sequence	zg30
Experiment	1D
Number of Scans	8
Receiver Gain	114
Relaxation Delay	1.0000
Pulse Width	13.5000
Acquisition Time	1.9923
Acquisition Date	2016-07-19T21:22:00
Modification Date	2016-07-19T21:22:00
Spectrometer Frequency	400.23
Spectral Width	8223.7
Lowest Frequency	-1640.3
Nucleus	1H
Acquired Size	16384
Spectral Size	32768

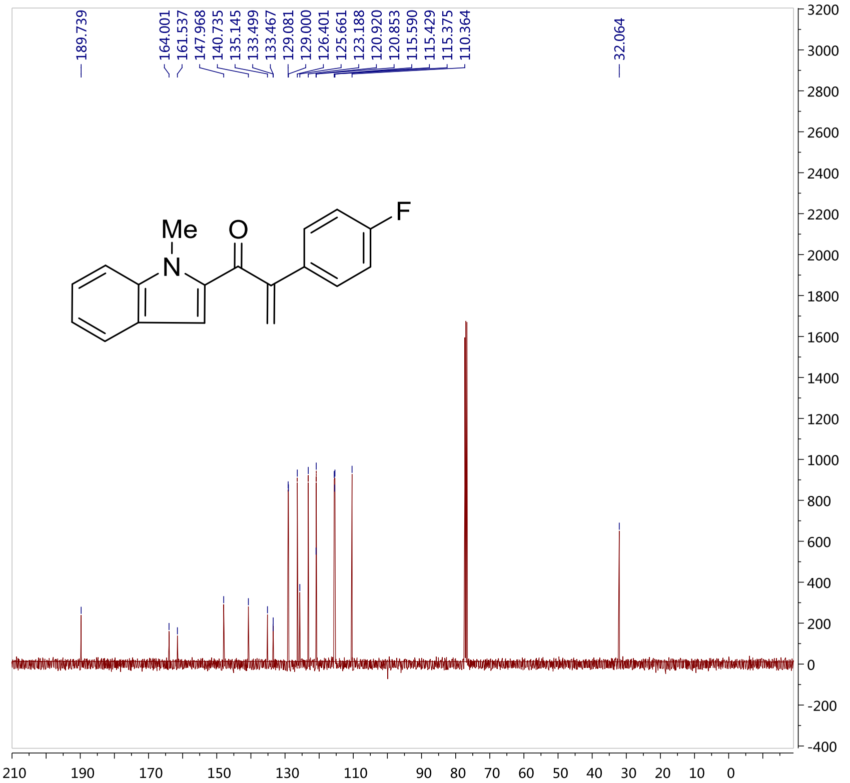

Parameters	
Parameter	Value
Title	wgp-16-192
Comment	C13CPD
Origin	Bruker BioSpin GmbH
Owner	common
Site	
Spectrometer	spect
Author	
Solvent	CDCl3
Temperature	-1513.0
Pulse Sequence	zgpg30
Experiment	1D
Number of Scans	360
Receiver Gain	203
Relaxation Delay	1.0000
Pulse Width	12.0000
Acquisition Time	0.5506
Acquisition Date	2016-07-19T21:24:00
Modification Date	2016-07-19T21:32:00
Spectrometer Frequency	100.64
Spectral Width	29761.9
Lowest Frequency	-4817.6
Nucleus	13C
Acquired Size	16384
Spectral Size	32768

2-(4-chlorophenyl)-1-(1-methyl-1H-indol-2-yl)prop-2-en-1-one (2n)

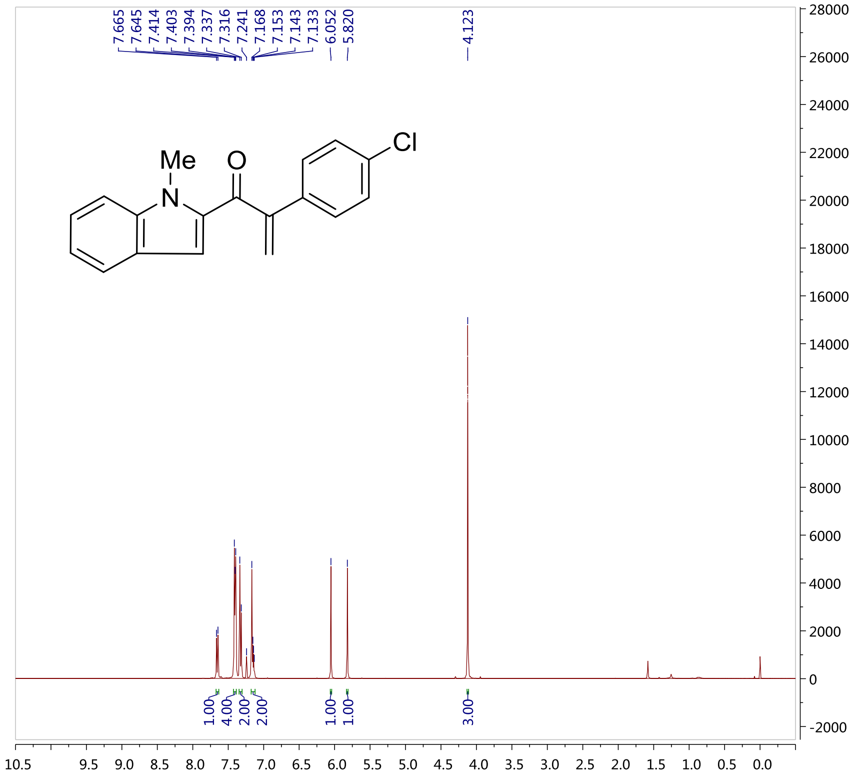

Parameters	
Parameter	Value
Title	wgp-16-189
Comment	PROTON
Origin	Bruker BioSpin GmbH
Owner	common
Site	
Spectrometer	spect
Author	
Solvent	CDCl3
Temperature	-1699.2
Pulse Sequence	zg30
Experiment	1D
Number of Scans	8
Receiver Gain	114
Relaxation Delay	1.0000
Pulse Width	13.5000
Acquisition Time	1.9923
Acquisition Date	2016-07-16T21:21:00
Modification Date	2016-07-16T21:21:00
Spectrometer Frequency	400.23
Spectral Width	8223.7
Lowest Frequency	-1640.3
Nucleus	1H
Acquired Size	16384
Spectral Size	32768

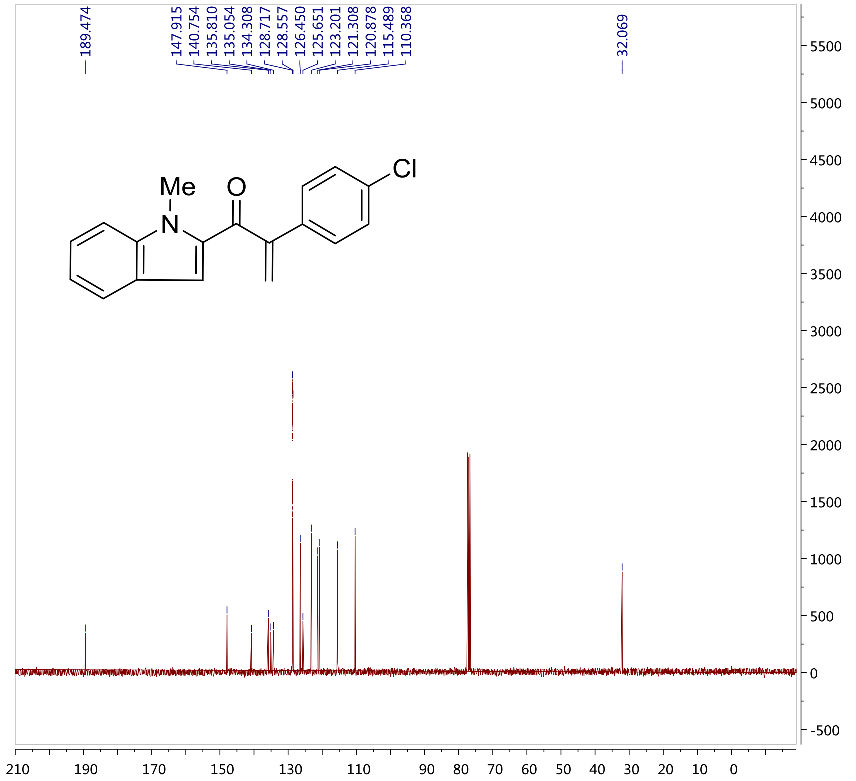

Parameters	
Parameter	Value
Title	wgp-16-189
Comment	C13CPD
Origin	Bruker BioSpin GmbH
Owner	common
Site	
Spectrometer	spect
Author	
Solvent	CDCl3
Temperature	-1705.9
Pulse Sequence	zgpg30
Experiment	1D
Number of Scans	455
Receiver Gain	203
Relaxation Delay	1.0000
Pulse Width	12.0000
Acquisition Time	0.5506
Acquisition Date	2016-07-16T21:25:00
Modification Date	2016-07-16T21:34:00
Spectrometer Frequency	100.64
Spectral Width	29761.9
Lowest Frequency	-4817.6
Nucleus	13C
Acquired Size	16384
Spectral Size	32768

2-(benzo[d][1,3]dioxol-5-yl)-1-(1-methyl-1H-indol-2-yl)prop-2-en-1-one (2o)

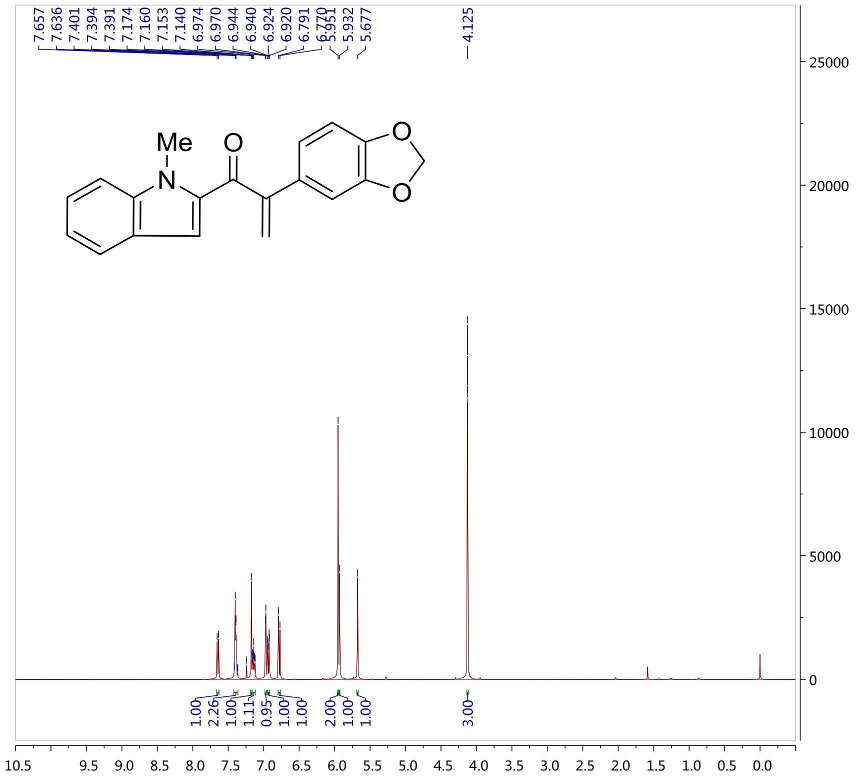

Parameter		Value
1	Data File Name	E:/ 0 NMR & MS/ 吲哚底物/ wgp-17-19/ 1/ fid
2	Title	wgp-17-19
3	Comment	PROTON
4	Origin	Bruker BioSpin GmbH
5	Owner	common
6	Site	
7	Spectrometer	spect
8	Author	
9	Solvent	CDCl3
10	Temperature	-150.1
11	Pulse Sequence	zg30
12	Experiment	1D
13	Number of Scans	8
14	Receiver Gain	128
15	Relaxation Delay	1.0000
16	Pulse Width	13.7000
17	Acquisition Time	1.9999
18	Acquisition Date	2016-08-16T16:58:00
19	Modification Date	2016-08-16T16:58:00
20	Spectrometer Frequency	400.13
21	Spectral Width	8223.7
22	Lowest Frequency	-1640.9
23	Nucleus	1H
24	Acquired Size	16446
25	Spectral Size	65536

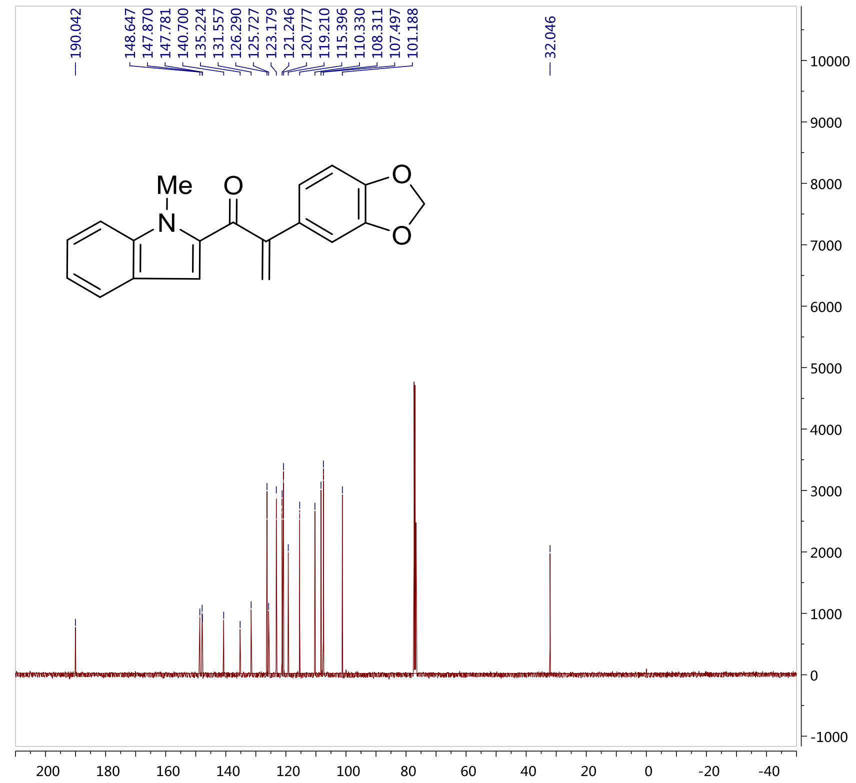

Parameter		Value
1	Data File Name	E:/ 0 NMR & MS/ 吲哚底物/ wgp-17-19/ 2/ fid
2	Title	wgp-17-19
3	Comment	C13CPD
4	Origin	Bruker BioSpin GmbH
5	Owner	common
6	Site	
7	Spectrometer	spect
8	Author	
9	Solvent	CDCl3
10	Temperature	-1604.5
11	Pulse Sequence	zgpg30
12	Experiment	1D
13	Number of Scans	873
14	Receiver Gain	203
15	Relaxation Delay	1.0000
16	Pulse Width	12.0000
17	Acquisition Time	0.4588
18	Acquisition Date	2016-08-16T17:00:00
19	Modification Date	2016-08-16T17:21:00
20	Spectrometer Frequency	100.61
21	Spectral Width	35714.3
22	Lowest Frequency	-7795.9
23	Nucleus	13C
24	Acquired Size	16384
25	Spectral Size	32768

1-(1,5-dimethyl-1H-indol-2-yl)-3-methyl-2-methylenebutan-1-one (2p)

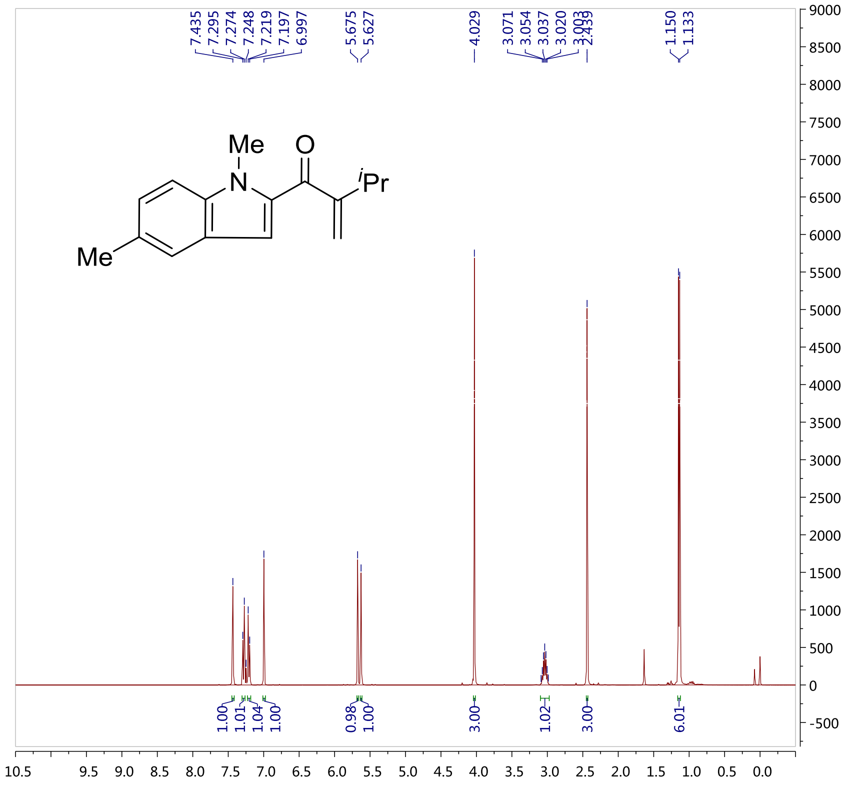

Parameter	Value
1 Data File Name	E:/ 0 NMR & MS/ 吲哚底物/ wgp-17-9/ 1/ fid
2 Title	wgp-17-9
3 Comment	PROTON
4 Origin	Bruker BioSpin GmbH
5 Owner	nmr
6 Site	
7 Spectrometer	spect
8 Author	
9 Solvent	CDCl3
10 Temperature	291.7
11 Pulse Sequence	zg30
12 Experiment	1D
13 Number of Scans	8
14 Receiver Gain	52
15 Relaxation Delay	1.0000
16 Pulse Width	9.1200
17 Acquisition Time	4.0894
18 Acquisition Date	2016-08-11T10:59:04
19 Modification Date	2016-08-11T10:59:00
20 Spectrometer Frequency	400.13
21 Spectral Width	8012.8
22 Lowest Frequency	-1535.4
23 Nucleus	1H
24 Acquired Size	32768
25 Spectral Size	65536

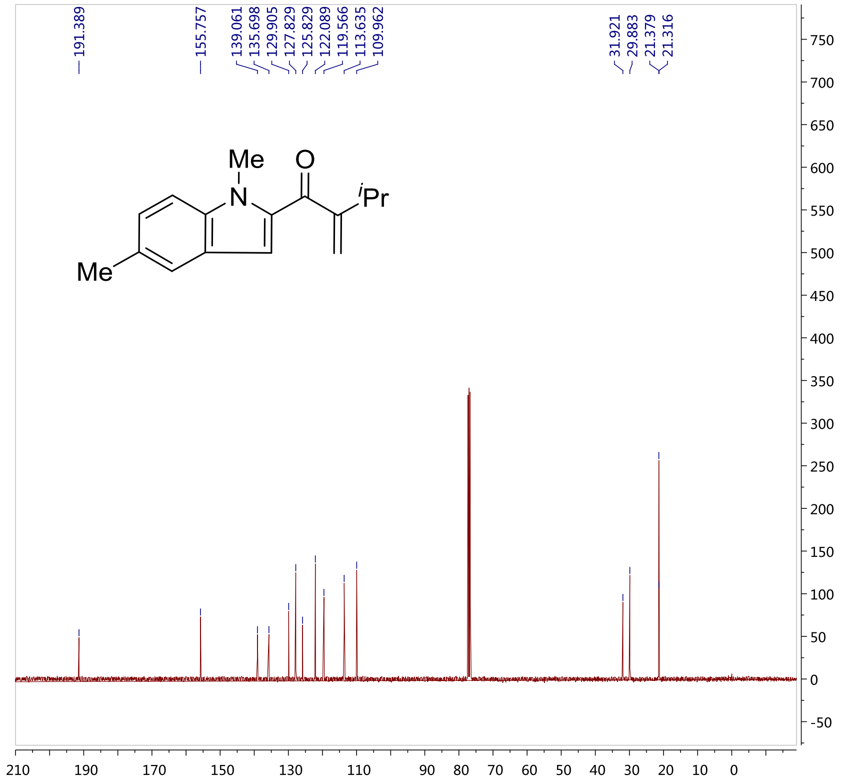

Parameter	Value
1 Data File Name	E:/ 0 NMR & MS/ 吲哚底物/ wgp-17-9/ 2/ fid
2 Title	wgp-17-9
3 Comment	C13CPD
4 Origin	Bruker BioSpin GmbH
5 Owner	nmr
6 Site	
7 Spectrometer	spect
8 Author	
9 Solvent	CDCl3
10 Temperature	292.2
11 Pulse Sequence	zgpg30
12 Experiment	1D
13 Number of Scans	257
14 Receiver Gain	29
15 Relaxation Delay	2.0000
16 Pulse Width	9.4000
17 Acquisition Time	1.3631
18 Acquisition Date	2016-08-11T11:00:31
19 Modification Date	2016-08-11T11:14:00
20 Spectrometer Frequency	100.61
21 Spectral Width	24038.5
22 Lowest Frequency	-1958.9
23 Nucleus	13C
24 Acquired Size	32768
25 Spectral Size	65536

1-(5-fluoro-1-methyl-1H-indol-2-yl)-3-methyl-2-methylenebutan-1-one (2q)

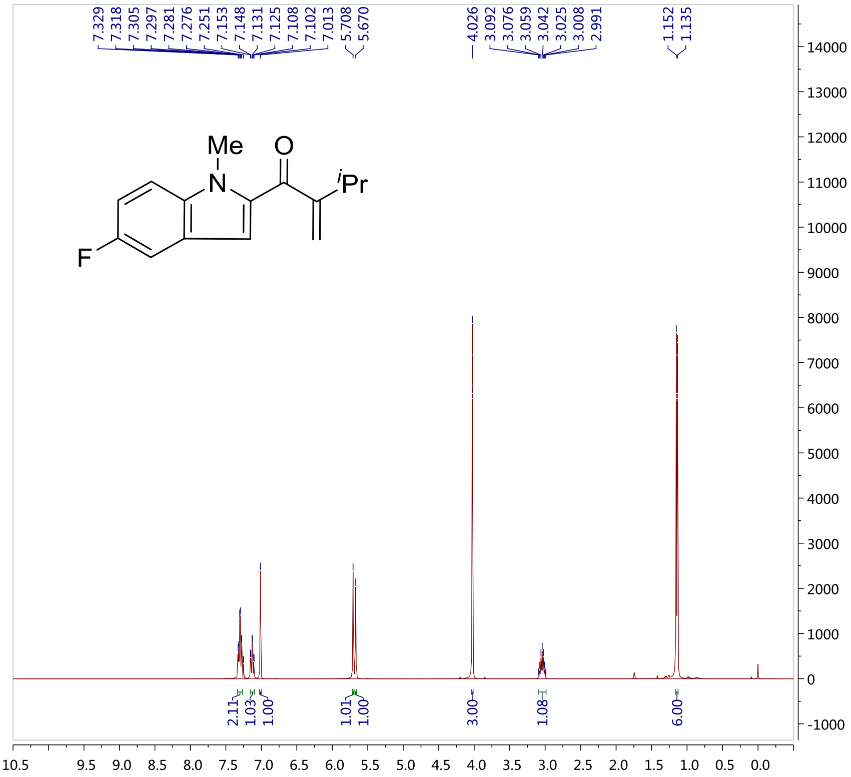

	Parameter	Value
1	Data File Name	E:/ 0 NMR & MS/ 吲哚底物/ wgp-17-31/ 1/ fid
2	Title	wgp-17-31
3	Comment	PROTON
4	Origin	Bruker BioSpin GmbH
5	Owner	nmr
6	Site	
7	Spectrometer	spect
8	Author	
9	Solvent	CDCl3
10	Temperature	0.0
11	Pulse Sequence	zg30
12	Experiment	1D
13	Number of Scans	8
14	Receiver Gain	27
15	Relaxation Delay	1.0000
16	Pulse Width	9.1200
17	Acquisition Time	4.0894
18	Acquisition Date	2016-08-29T14:21:53
19	Modification Date	2016-08-29T14:21:00
20	Spectrometer Frequency	400.13
21	Spectral Width	8012.8
22	Lowest Frequency	-1535.4
23	Nucleus	1H
24	Acquired Size	32768
25	Spectral Size	65536

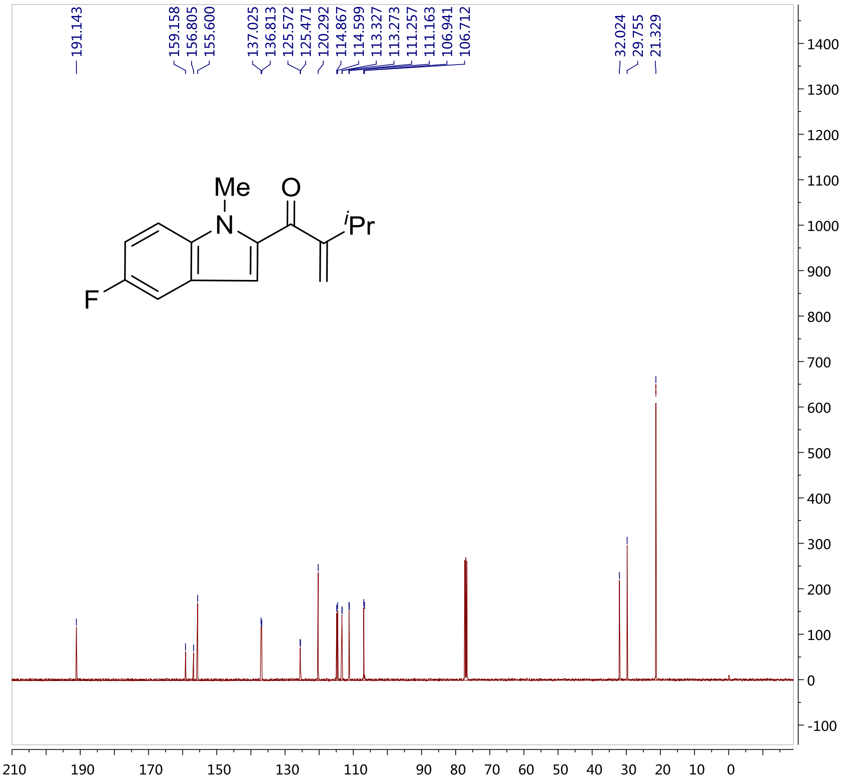

	Parameter	Value
1	Data File Name	E:/ 0 NMR & MS/ 吲哚底物/ wgp-17-31/ 2/ fid
2	Title	wgp-17-31
3	Comment	C13CPD
4	Origin	Bruker BioSpin GmbH
5	Owner	nmr
6	Site	
7	Spectrometer	spect
8	Author	
9	Solvent	CDCl3
10	Temperature	0.0
11	Pulse Sequence	zgpg30
12	Experiment	1D
13	Number of Scans	238
14	Receiver Gain	29
15	Relaxation Delay	2.0000
16	Pulse Width	9.4000
17	Acquisition Time	1.3631
18	Acquisition Date	2016-08-29T14:24:30
19	Modification Date	2016-08-29T14:36:00
20	Spectrometer Frequency	100.61
21	Spectral Width	24038.5
22	Lowest Frequency	-1958.9
23	Nucleus	13C
24	Acquired Size	32768
25	Spectral Size	65536

1-(5-chloro-1-methyl-1H-indol-2-yl)-3-methyl-2-methylenebutan-1-one (2r)

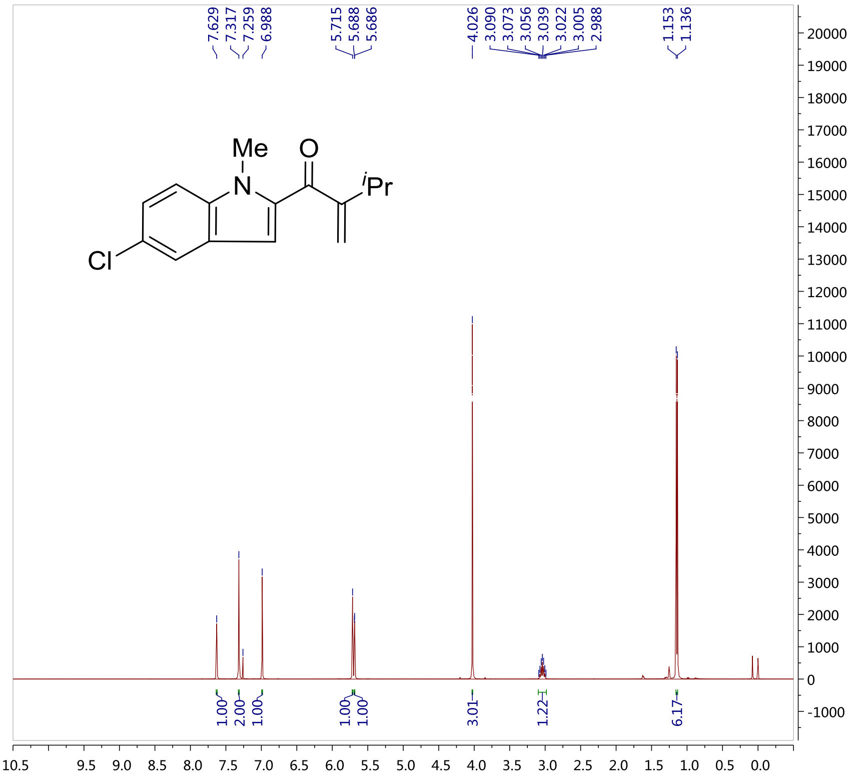

Parameter	Value
1 Data File Name	E:/ 0 Data/ 1 吲哚底物/ wgp-17-50/ 1/ fid
2 Title	wgp-17-50
3 Comment	PROTON
4 Origin	Bruker BioSpin GmbH
5 Owner	nmr
6 Site	
7 Spectrometer	spect
8 Author	
9 Solvent	CDCl3
10 Temperature	291.6
11 Pulse Sequence	zg30
12 Experiment	1D
13 Number of Scans	8
14 Receiver Gain	57
15 Relaxation Delay	1.0000
16 Pulse Width	9.1200
17 Acquisition Time	4.0894
18 Acquisition Date	2016-09-22T09:27:28
19 Modification Date	2016-09-22T09:27:00
20 Spectrometer Frequency	400.13
21 Spectral Width	8012.8
22 Lowest Frequency	-1577.2
23 Nucleus	1H
24 Acquired Size	32768
25 Spectral Size	65536

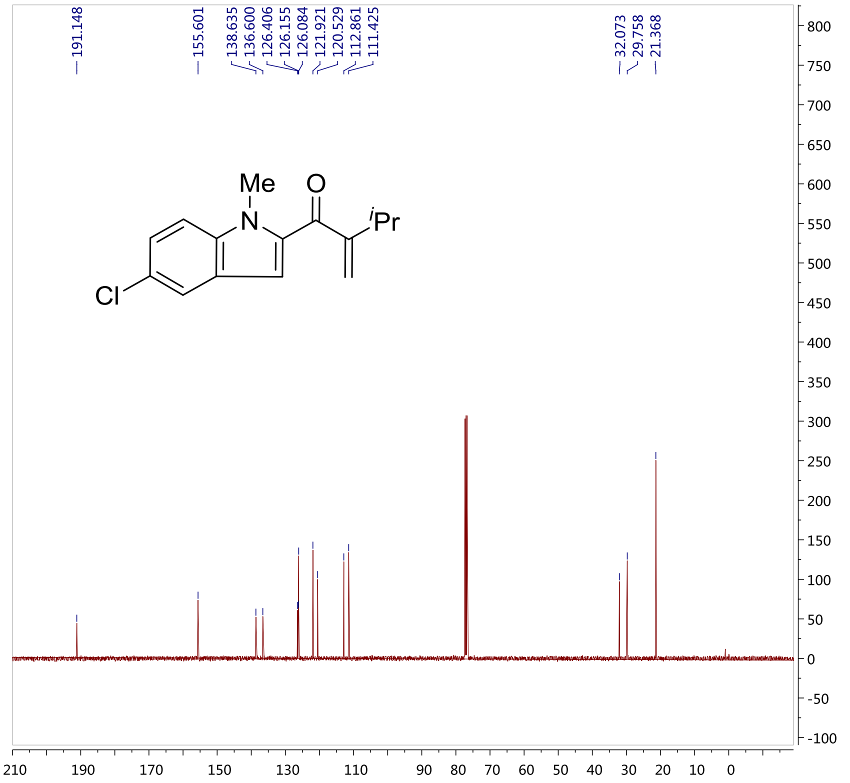

Parameter	Value
1 Data File Name	E:/ 0 Data/ 1 吲哚底物/ wgp-17-50/ 2/ fid
2 Title	wgp-17-50
3 Comment	C13CPD
4 Origin	Bruker BioSpin GmbH
5 Owner	nmr
6 Site	
7 Spectrometer	spect
8 Author	
9 Solvent	CDCl3
10 Temperature	292.2
11 Pulse Sequence	zgpg30
12 Experiment	1D
13 Number of Scans	302
14 Receiver Gain	29
15 Relaxation Delay	2.0000
16 Pulse Width	9.4000
17 Acquisition Time	1.3631
18 Acquisition Date	2016-09-22T09:30:49
19 Modification Date	2016-09-22T09:47:00
20 Spectrometer Frequency	100.61
21 Spectral Width	24038.5
22 Lowest Frequency	-1958.9
23 Nucleus	13C
24 Acquired Size	32768
25 Spectral Size	65536

1-(4-fluoro-1-methyl-1H-indol-2-yl)-3-methyl-2-methylenebutan-1-one (2s)

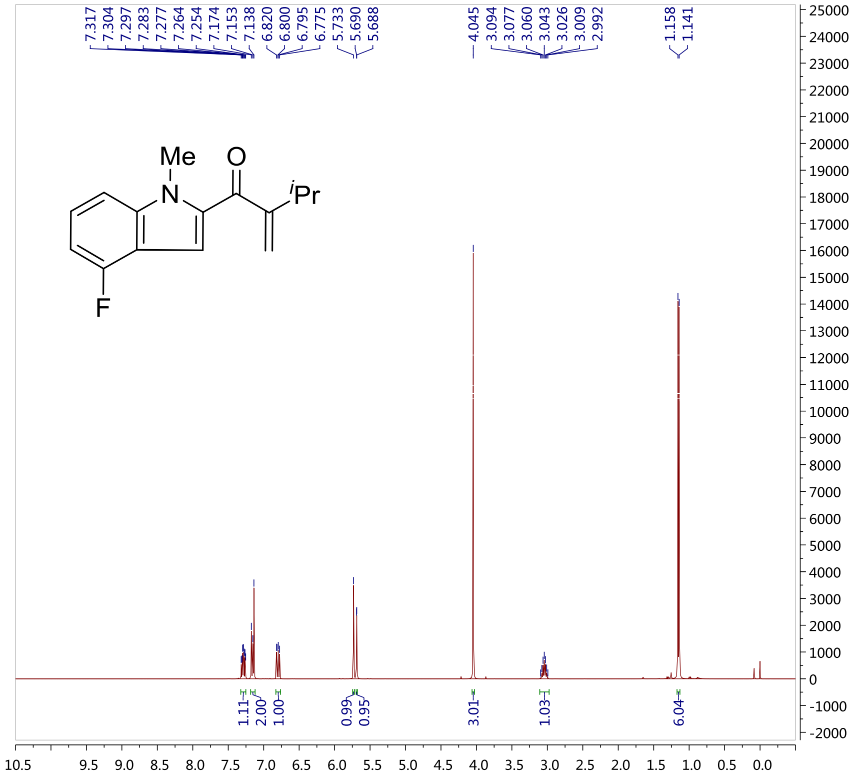

Parameter	Value
1 Data File Name	E:/ 0 Data/ 1 吲哚底物/ wgp-17-59/ 1/ fid
2 Title	wgp-17-59
3 Comment	PROTON
4 Origin	Bruker BioSpin GmbH
5 Owner	nmr
6 Site	
7 Spectrometer	spect
8 Author	
9 Solvent	CDCl3
10 Temperature	291.6
11 Pulse Sequence	zg30
12 Experiment	1D
13 Number of Scans	8
14 Receiver Gain	41
15 Relaxation Delay	1.0000
16 Pulse Width	9.1200
17 Acquisition Time	4.0894
18 Acquisition Date	2016-09-30T10:26:08
19 Modification Date	2016-09-30T10:26:00
20 Spectrometer Frequency	400.13
21 Spectral Width	8012.8
22 Lowest Frequency	-1547.5
23 Nucleus	1H
24 Acquired Size	32768
25 Spectral Size	65536

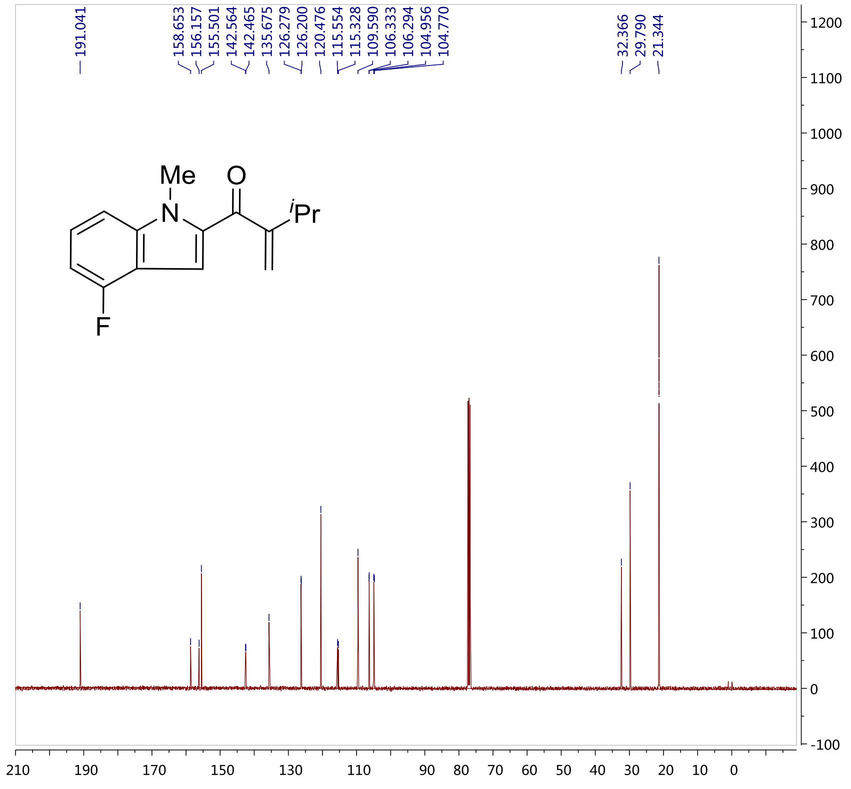

Parameter	Value
1 Data File Name	E:/ 0 Data/ 1 吲哚底物/ wgp-17-59/ 2/ fid
2 Title	wgp-17-59
3 Comment	C13CPD
4 Origin	Bruker BioSpin GmbH
5 Owner	nmr
6 Site	
7 Spectrometer	spect
8 Author	
9 Solvent	CDCl3
10 Temperature	292.7
11 Pulse Sequence	zgpg30
12 Experiment	1D
13 Number of Scans	438
14 Receiver Gain	29
15 Relaxation Delay	2.0000
16 Pulse Width	9.4000
17 Acquisition Time	1.3631
18 Acquisition Date	2016-09-30T10:46:14
19 Modification Date	2016-09-30T10:52:00
20 Spectrometer Frequency	100.61
21 Spectral Width	24038.5
22 Lowest Frequency	-1958.9
23 Nucleus	13C
24 Acquired Size	32768
25 Spectral Size	65536

8. NMR Spectra of Cyclization Products and Transformation Product

(+)-2,4-dimethyl-1,4-dihydrocyclopenta[b]indol-3(2H)-one (3a)

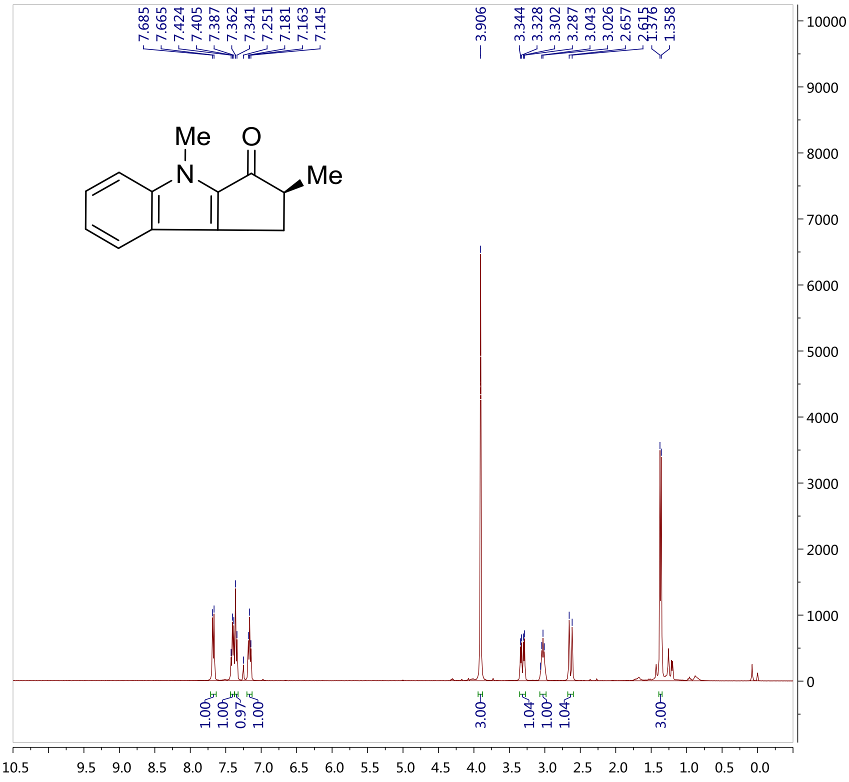

Parameters	
Parameter	Value
Title	wgp-14-189-A
Comment	PROTON
Origin	Bruker BioSpin GmbH
Owner	nmr
Site	
Spectrometer	spect
Author	
Solvent	CDCl3
Temperature	302.7
Pulse Sequence	zg30
Experiment	1D
Number of Scans	8
Receiver Gain	144
Relaxation Delay	1.0000
Pulse Width	13.7000
Acquisition Time	1.9999
Acquisition Date	2015-03-23T16:06:00
Modification Date	2015-03-23T16:06:00
Spectrometer Frequency	400.13
Spectral Width	8223.7
Lowest Frequency	-1640.9
Nucleus	1H
Acquired Size	16446
Spectral Size	65536

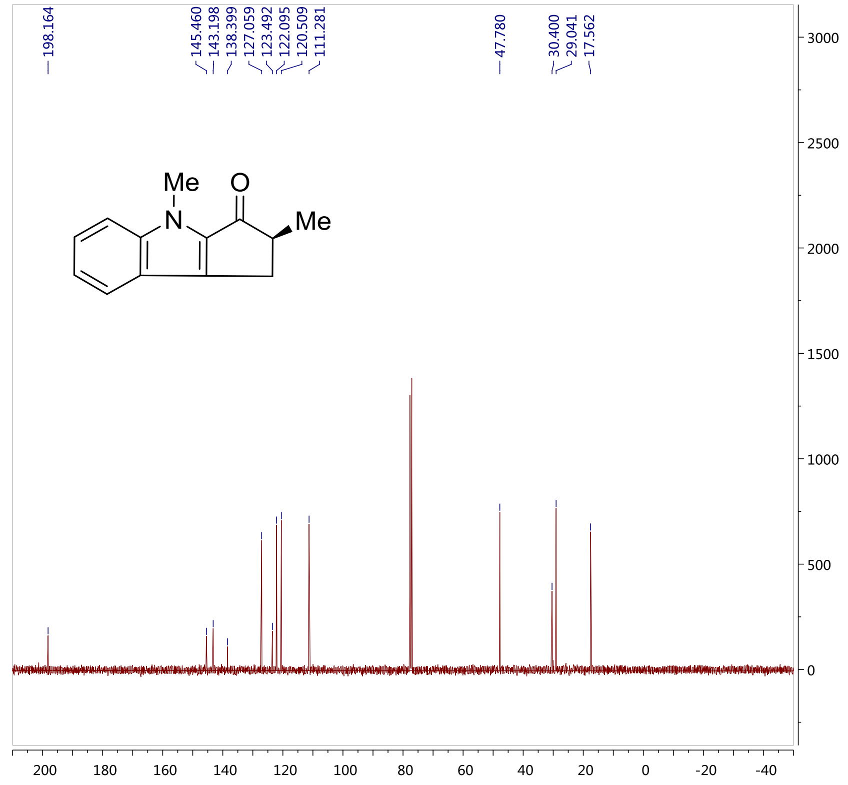

Parameters	
Parameter	Value
Title	wgp-14-189-A
Comment	C13CPD
Origin	Bruker BioSpin GmbH
Owner	nmr
Site	
Spectrometer	spect
Author	
Solvent	CDCl3
Temperature	303.5
Pulse Sequence	zgpg30
Experiment	1D
Number of Scans	205
Receiver Gain	203
Relaxation Delay	1.0000
Pulse Width	12.0000
Acquisition Time	0.4588
Acquisition Date	2015-03-23T16:09:00
Modification Date	2015-03-23T16:12:00
Spectrometer Frequency	100.61
Spectral Width	35714.3
Lowest Frequency	-7796.3
Nucleus	13C
Acquired Size	16384
Spectral Size	32768

(+)-4-isopropyl-2-methyl-1,4-dihydrocyclopenta[b]indol-3(2H)-one (3b)

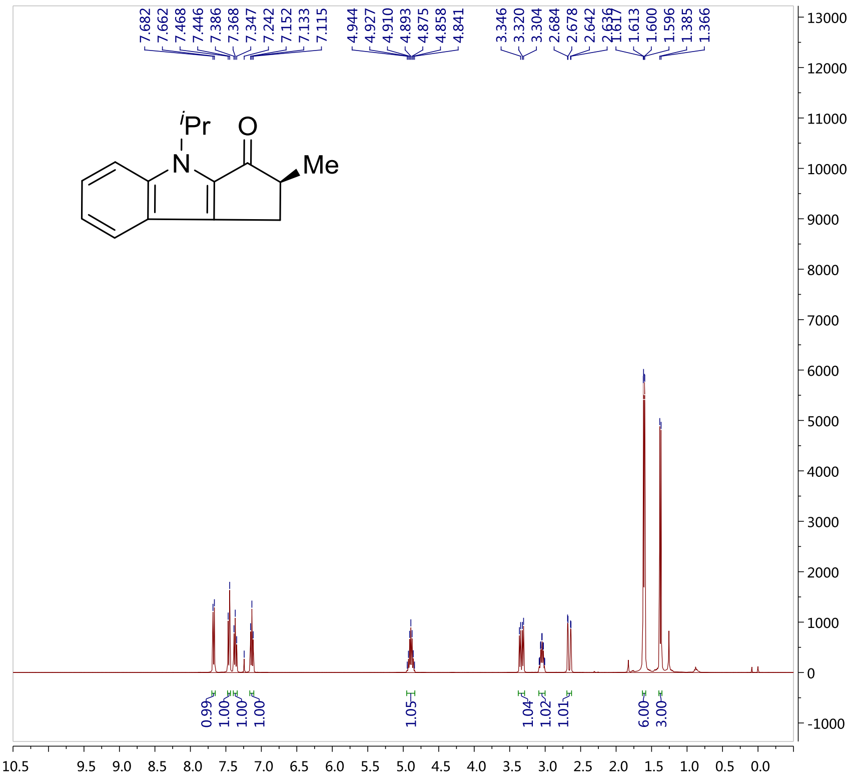

Parameters	
Parameter	Value
Title	wgp-16-90-A
Comment	PROTON
Origin	Bruker BioSpin GmbH
Owner	nmr
Site	
Spectrometer	spect
Author	
Solvent	CDCl3
Temperature	292.5
Pulse Sequence	zg30
Experiment	1D
Number of Scans	8
Receiver Gain	36
Relaxation Delay	1.0000
Pulse Width	9.1200
Acquisition Time	4.0894
Acquisition Date	2016-03-31T19:39:19
Modification Date	2016-03-31T19:39:00
Spectrometer Frequency	400.13
Spectral Width	8012.8
Lowest Frequency	-1535.4
Nucleus	1H
Acquired Size	32768
Spectral Size	65536

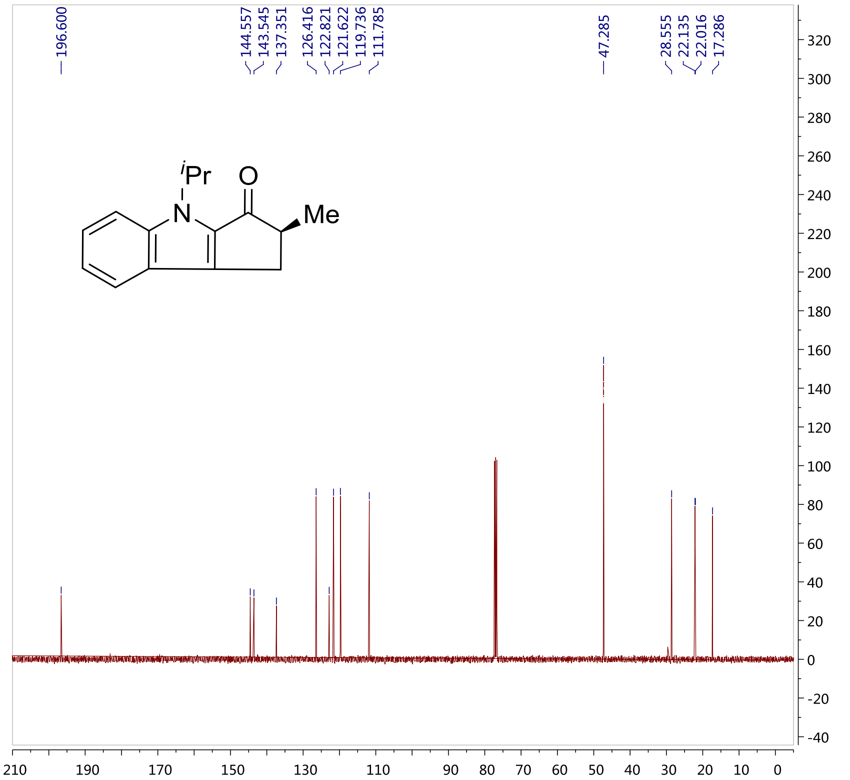

Parameters	
Parameter	Value
Title	wgp-16-90-A
Comment	C13CPD
Origin	Bruker BioSpin GmbH
Owner	nmr
Site	
Spectrometer	spect
Author	
Solvent	CDCl3
Temperature	292.9
Pulse Sequence	zgpg30
Experiment	1D
Number of Scans	74
Receiver Gain	36
Relaxation Delay	2.0000
Pulse Width	9.4000
Acquisition Time	1.3631
Acquisition Date	2016-03-31T19:40:49
Modification Date	2016-03-31T19:44:00
Spectrometer Frequency	100.61
Spectral Width	24038.5
Lowest Frequency	-1958.9
Nucleus	13C
Acquired Size	32768
Spectral Size	65536

(+)-4-allyl-2-methyl-1,4-dihydrocyclopenta[b]indol-3(2H)-one (3c)

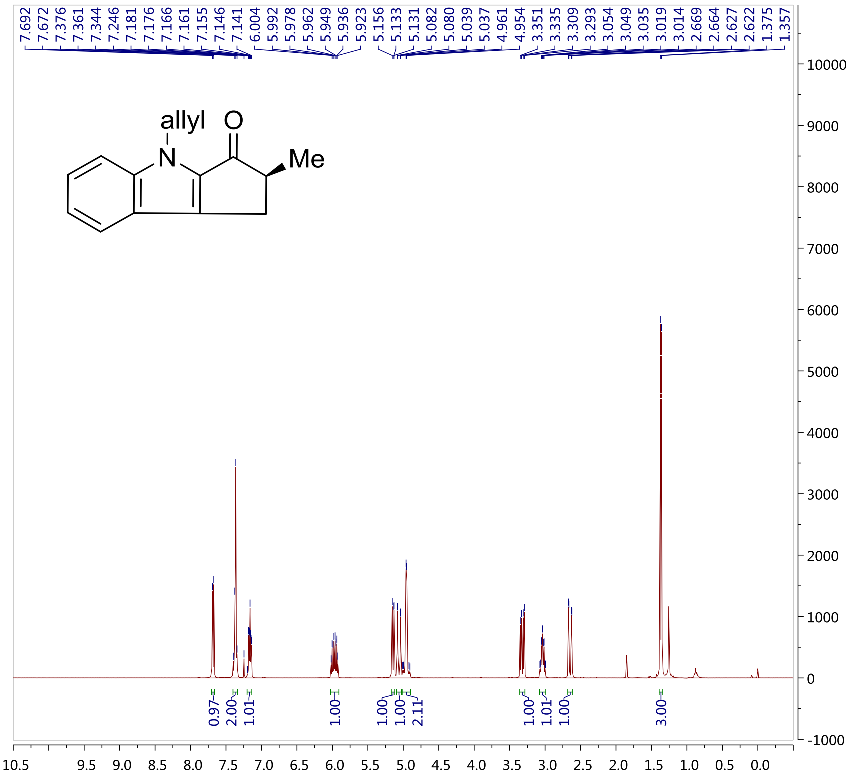

Parameters	
Parameter	Value
Title	wgp-16-89-A
Comment	PROTON
Origin	Bruker BioSpin GmbH
Owner	nmr
Site	
Spectrometer	spect
Author	
Solvent	CDCl3
Temperature	292.6
Pulse Sequence	zg30
Experiment	1D
Number of Scans	8
Receiver Gain	41
Relaxation Delay	1.0000
Pulse Width	9.1200
Acquisition Time	4.0894
Acquisition Date	2016-03-31T19:30:19
Modification Date	2016-03-31T19:30:00
Spectrometer Frequency	400.13
Spectral Width	8012.8
Lowest Frequency	-1535.4
Nucleus	1H
Acquired Size	32768
Spectral Size	65536

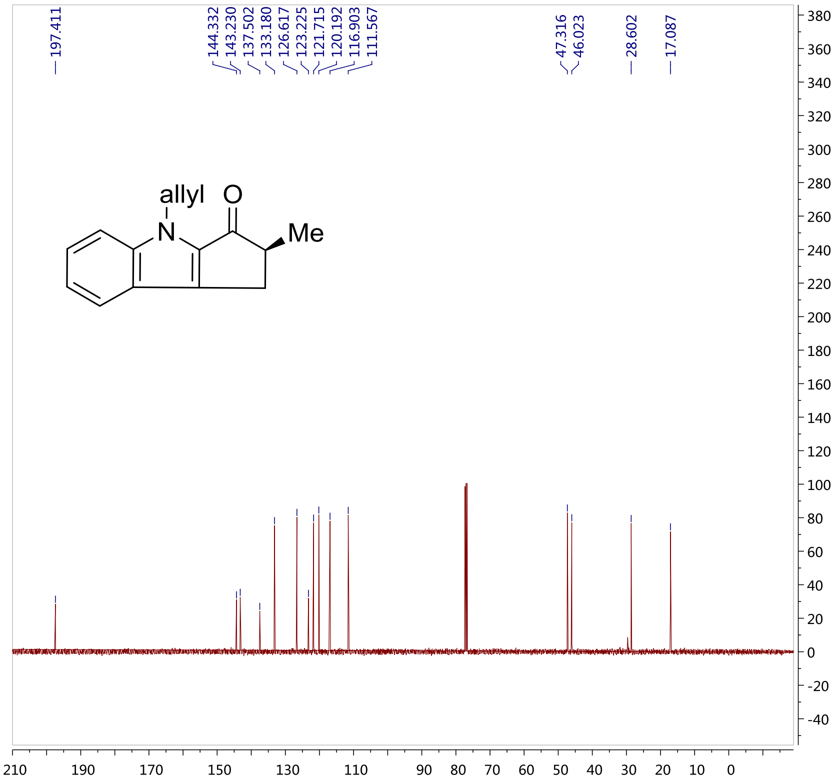

Parameters	
Parameter	Value
Title	wgp-16-89-A
Comment	C13CPD
Origin	Bruker BioSpin GmbH
Owner	nmr
Site	
Spectrometer	spect
Author	
Solvent	CDCl3
Temperature	293.1
Pulse Sequence	zgpg30
Experiment	1D
Number of Scans	67
Receiver Gain	36
Relaxation Delay	2.0000
Pulse Width	9.4000
Acquisition Time	1.3631
Acquisition Date	2016-03-31T19:32:22
Modification Date	2016-03-31T19:35:00
Spectrometer Frequency	100.61
Spectral Width	24038.5
Lowest Frequency	-1958.9
Nucleus	13C
Acquired Size	32768
Spectral Size	65536

(-)-4-benzyl-2-methyl-1,4-dihydrocyclopenta[b]indol-3(2H)-one (3d)

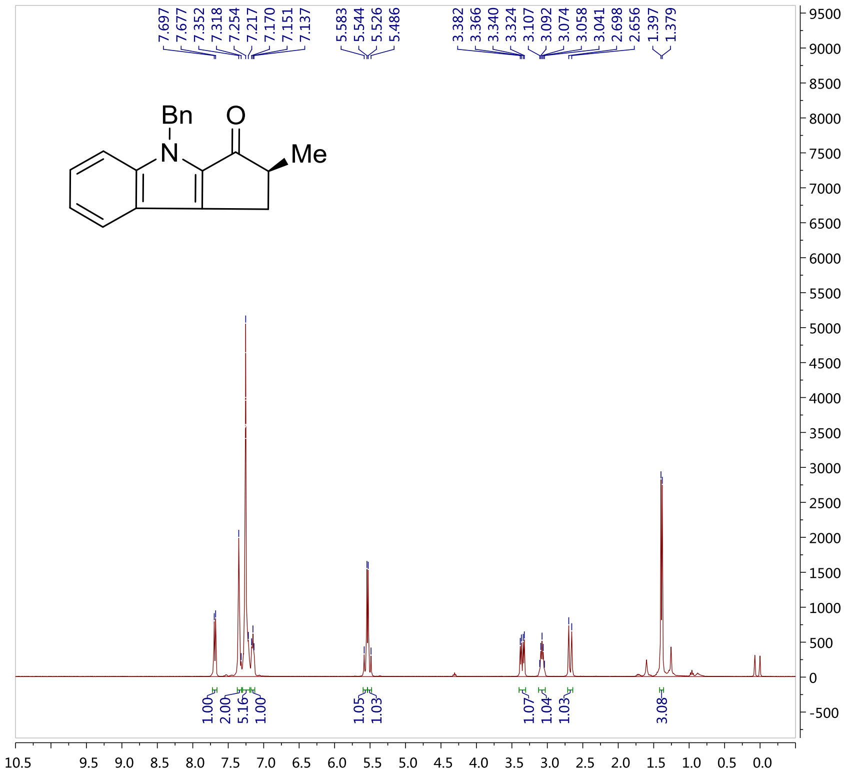

Parameters	
Parameter	Value
Title	wgp-15-4-B
Comment	PROTON
Origin	Bruker BioSpin GmbH
Owner	nmr
Site	
Spectrometer	spect
Author	
Solvent	CDCl3
Temperature	295.5
Pulse Sequence	zg30
Experiment	1D
Number of Scans	8
Receiver Gain	203
Relaxation Delay	1.0000
Pulse Width	13.7000
Acquisition Time	1.9999
Acquisition Date	2015-04-22T16:32:00
Modification Date	2015-04-22T16:32:00
Spectrometer Frequency	400.13
Spectral Width	8223.7
Lowest Frequency	-1640.9
Nucleus	1H
Acquired Size	16446
Spectral Size	65536

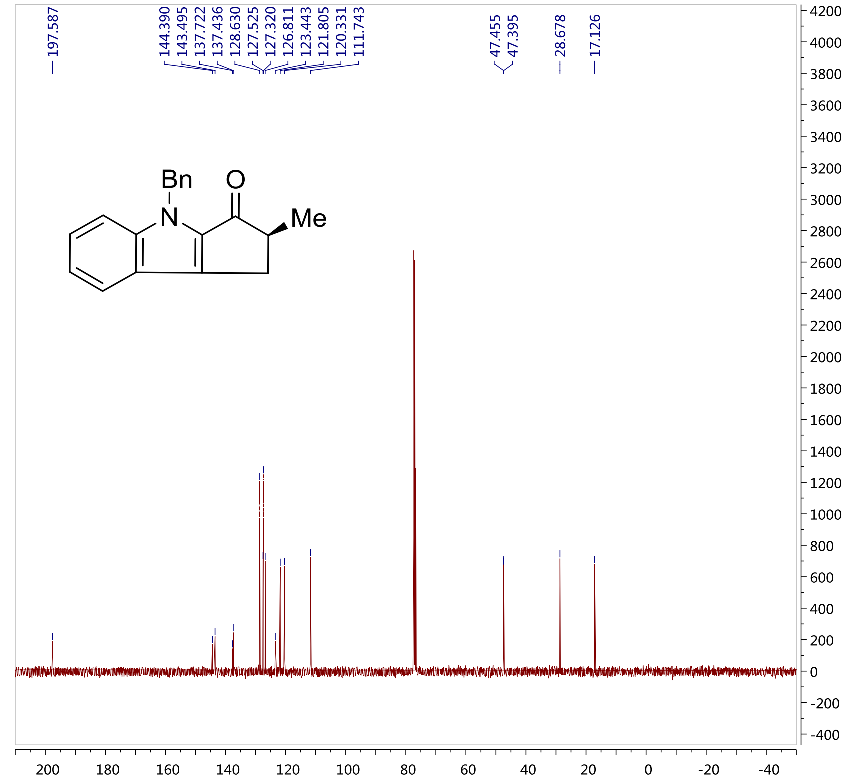

Parameters	
Parameter	Value
Title	wgp-15-4-B
Comment	C13CPD
Origin	Bruker BioSpin GmbH
Owner	nmr
Site	
Spectrometer	spect
Author	
Solvent	CDCl3
Temperature	296.2
Pulse Sequence	zgpg30
Experiment	1D
Number of Scans	395
Receiver Gain	203
Relaxation Delay	1.0000
Pulse Width	12.0000
Acquisition Time	0.4588
Acquisition Date	2015-04-22T16:34:00
Modification Date	2015-04-22T16:43:00
Spectrometer Frequency	100.61
Spectral Width	35714.3
Lowest Frequency	-7796.3
Nucleus	13C
Acquired Size	16384
Spectral Size	32768

(+)-2-methyl-4-phenyl-1,4-dihydrocyclopenta[b]indol-3(2H)-one (3e)

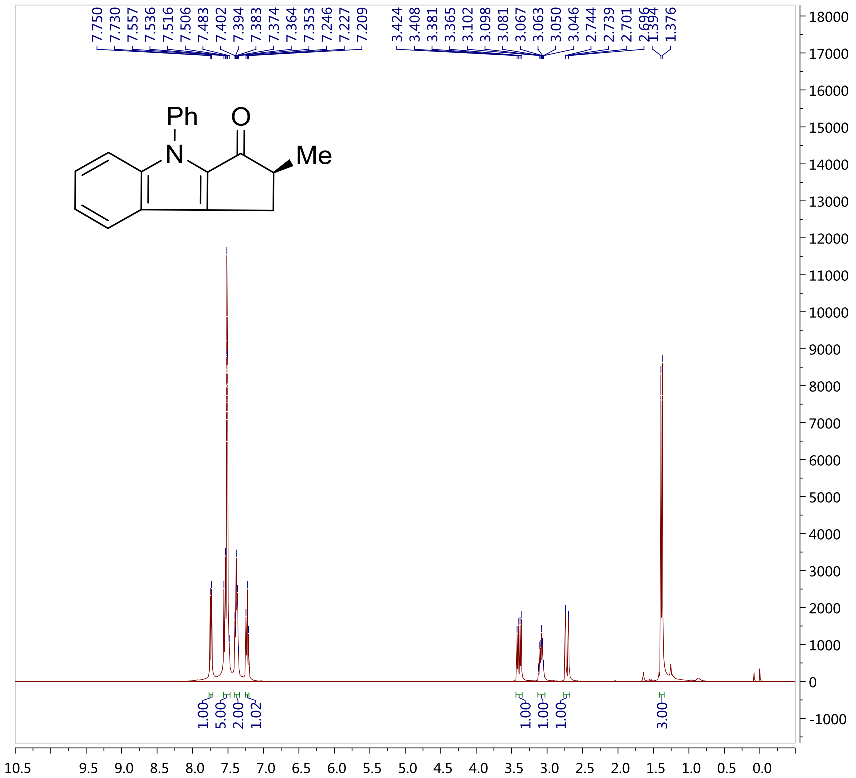

Parameters	
Parameter	Value
Title	wgp-16-63
Comment	
Origin	Bruker BioSpin GmbH
Owner	nmr
Site	
Spectrometer	spect
Author	
Solvent	CDCl3
Temperature	293.8
Pulse Sequence	zg30
Experiment	1D
Number of Scans	8
Receiver Gain	66
Relaxation Delay	1.0000
Pulse Width	9.8000
Acquisition Time	1.9999
Acquisition Date	2016-03-02T15:36:16
Modification Date	2016-03-02T15:36:00
Spectrometer Frequency	400.13
Spectral Width	8012.8
Lowest Frequency	-2125.8
Nucleus	1H
Acquired Size	16025
Spectral Size	32768

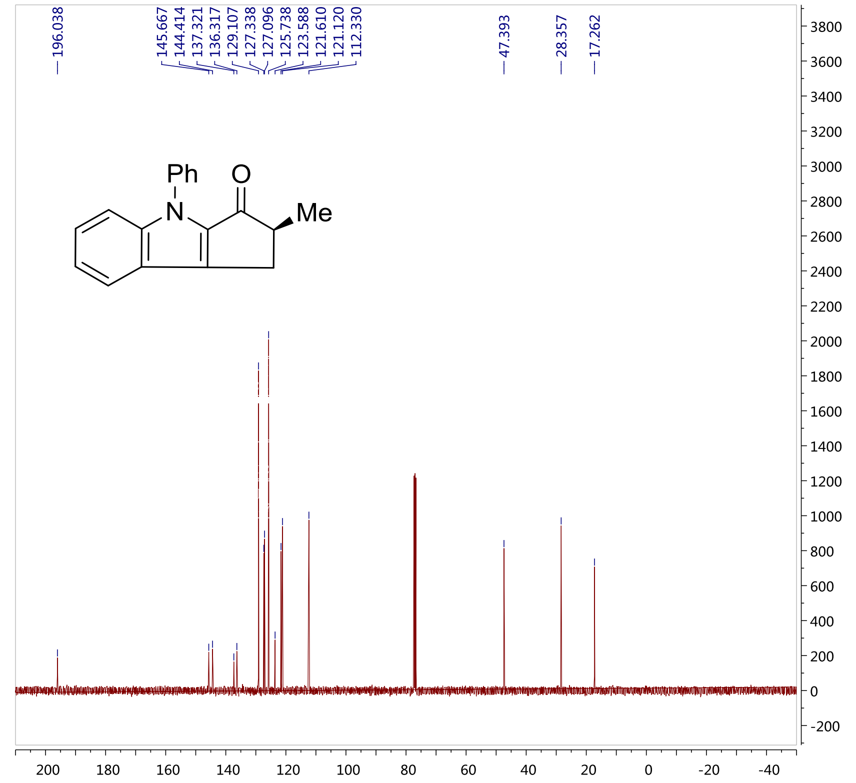

Parameters	
Parameter	Value
Title	wgp-16-63
Comment	13CPD
Origin	Bruker BioSpin GmbH
Owner	nmr
Site	
Spectrometer	spect
Author	
Solvent	CDCl3
Temperature	293.8
Pulse Sequence	zgpg30
Experiment	1D
Number of Scans	234
Receiver Gain	205
Relaxation Delay	1.0000
Pulse Width	9.3000
Acquisition Time	0.8039
Acquisition Date	2016-03-02T15:38:13
Modification Date	2016-03-02T15:45:00
Spectrometer Frequency	100.61
Spectral Width	40760.9
Lowest Frequency	-15349.8
Nucleus	13C
Acquired Size	32768
Spectral Size	65536

(+)-4-(4-methoxyphenyl)-2-methyl-1,4-dihydrocyclopenta[b]indol-3(2H)-one (3f)

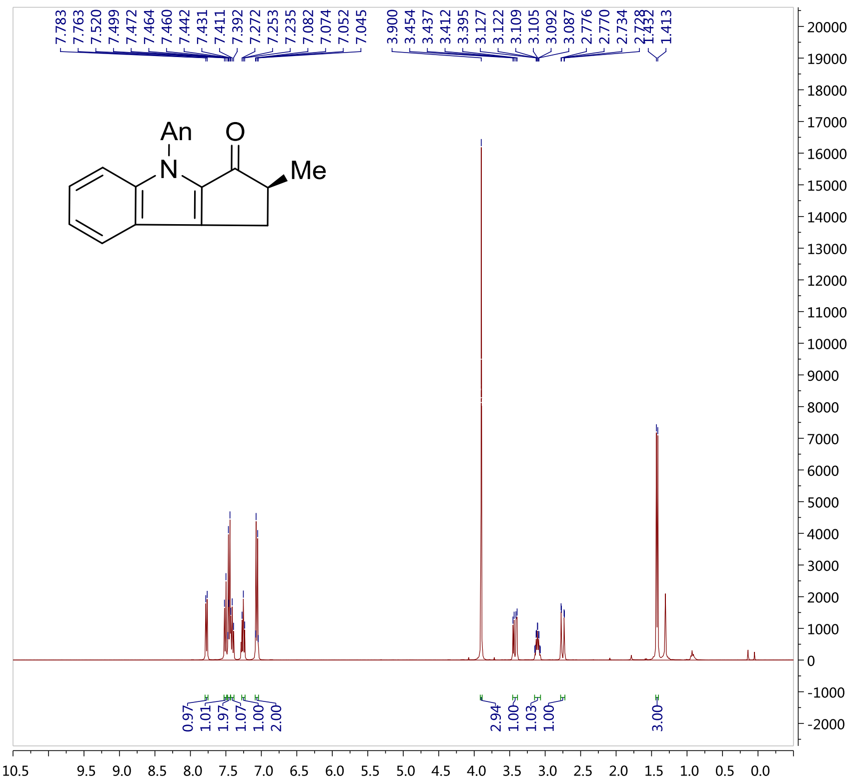

Parameters	
Parameter	Value
Title	wgp-16-79-A
Comment	test
Origin	Bruker BioSpin GmbH
Owner	nmr
Site	
Spectrometer	spect
Author	
Solvent	CDCl3
Temperature	293.3
Pulse Sequence	zg30
Experiment	1D
Number of Scans	8
Receiver Gain	32
Relaxation Delay	1.0000
Pulse Width	9.8000
Acquisition Time	1.9999
Acquisition Date	2016-03-25T20:09:55
Modification Date	2016-03-25T20:09:00
Spectrometer Frequency	400.13
Spectral Width	8012.8
Lowest Frequency	-1535.4
Nucleus	1H
Acquired Size	16025
Spectral Size	32768

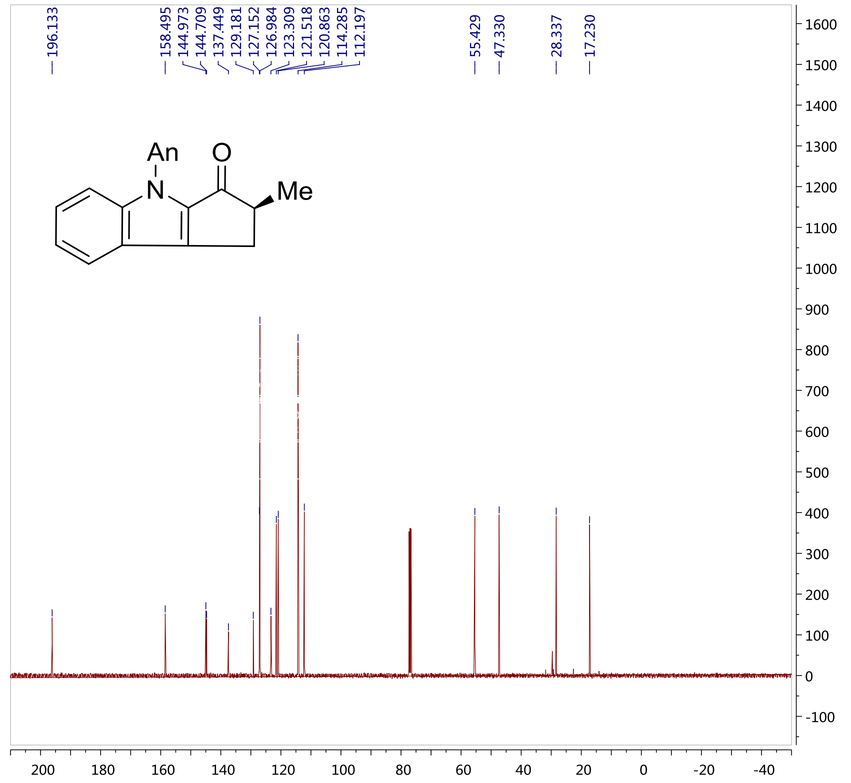

Parameters	
Parameter	Value
Title	wgp-16-79-A
Comment	test
Origin	Bruker BioSpin GmbH
Owner	nmr
Site	
Spectrometer	spect
Author	
Solvent	CDCl3
Temperature	293.6
Pulse Sequence	zgpg30
Experiment	1D
Number of Scans	133
Receiver Gain	97
Relaxation Delay	1.0000
Pulse Width	9.8000
Acquisition Time	0.8039
Acquisition Date	2016-03-25T20:11:34
Modification Date	2016-03-25T20:15:00
Spectrometer Frequency	100.61
Spectral Width	40760.9
Lowest Frequency	-10320.1
Nucleus	13C
Acquired Size	32768
Spectral Size	65536

(+)-2-methyl-4-(4-(trifluoromethyl)phenyl)-1,4-dihydrocyclopenta[b]indol-3(2H)-one (3g)

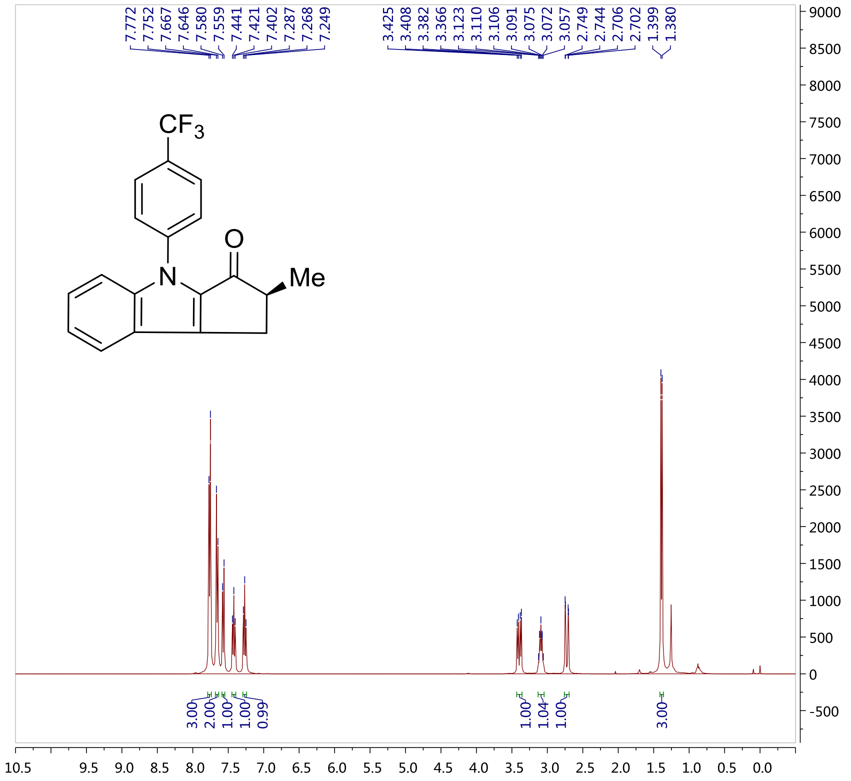

Parameters	
Parameter	Value
Title	wgp-16-78-A
Comment	PROTON
Origin	Bruker BioSpin GmbH
Owner	nmr
Site	
Spectrometer	spect
Author	
Solvent	CDCl3
Temperature	291.9
Pulse Sequence	zg30
Experiment	1D
Number of Scans	8
Receiver Gain	47
Relaxation Delay	1.0000
Pulse Width	9.1200
Acquisition Time	4.0894
Acquisition Date	2016-03-23T14:34:49
Modification Date	2016-03-23T14:34:00
Spectrometer Frequency	400.13
Spectral Width	8012.8
Lowest Frequency	-1535.4
Nucleus	1H
Acquired Size	32768
Spectral Size	65536

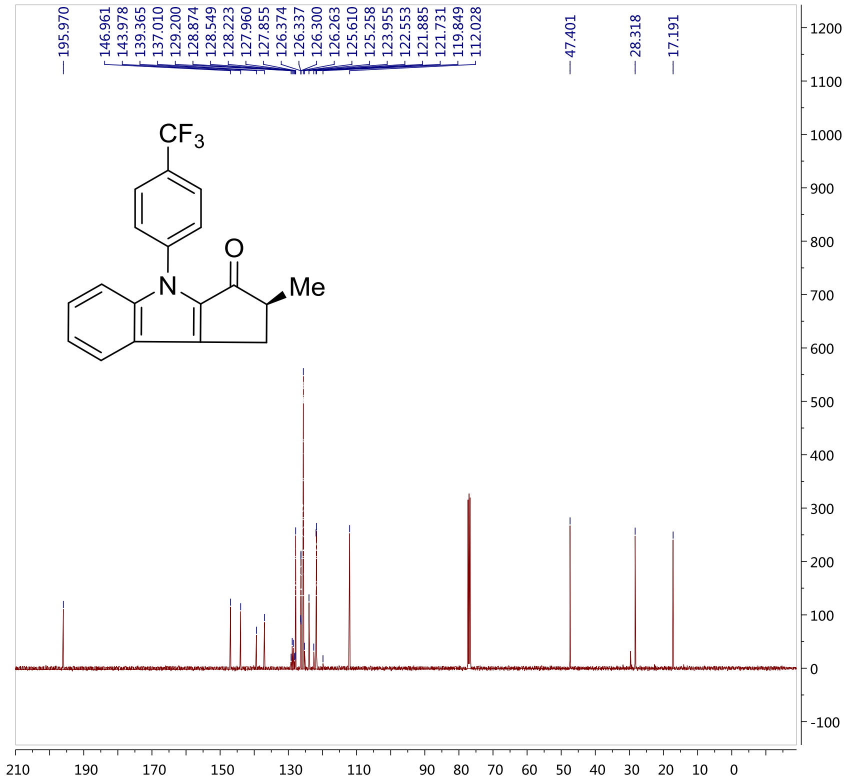

Parameters	
Parameter	Value
Title	wgp-16-78-A
Comment	C13CPD
Origin	Bruker BioSpin GmbH
Owner	nmr
Site	
Spectrometer	spect
Author	
Solvent	CDCl3
Temperature	292.5
Pulse Sequence	zgpg30
Experiment	1D
Number of Scans	195
Receiver Gain	47
Relaxation Delay	2.0000
Pulse Width	9.4000
Acquisition Time	1.3631
Acquisition Date	2016-03-23T14:36:25
Modification Date	2016-03-23T14:46:00
Spectrometer Frequency	100.61
Spectral Width	24038.5
Lowest Frequency	-1958.9
Nucleus	13C
Acquired Size	32768
Spectral Size	65536

(+)-2-methyl-4-(naphthalen-2-yl)-1,4-dihydrocyclopenta[b]indol-3(2H)-one (3h)

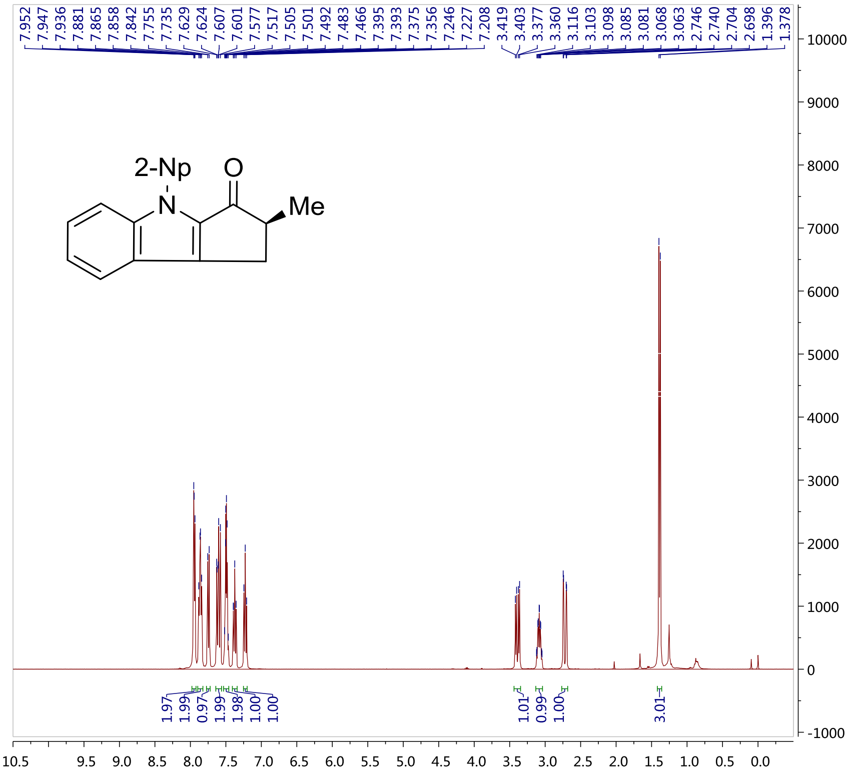

Parameters	
Parameter	Value
Title	wgp-16-104-A
Comment	test
Origin	Bruker BioSpin GmbH
Owner	nmr
Site	
Spectrometer	spect
Author	
Solvent	CDCl3
Temperature	293.1
Pulse Sequence	zg30
Experiment	1D
Number of Scans	8
Receiver Gain	32
Relaxation Delay	1.0000
Pulse Width	9.8000
Acquisition Time	1.9999
Acquisition Date	2016-04-10T13:20:30
Modification Date	2016-04-10T13:20:00
Spectrometer Frequency	400.13
Spectral Width	8012.8
Lowest Frequency	-1535.4
Nucleus	1H
Acquired Size	16025
Spectral Size	32768

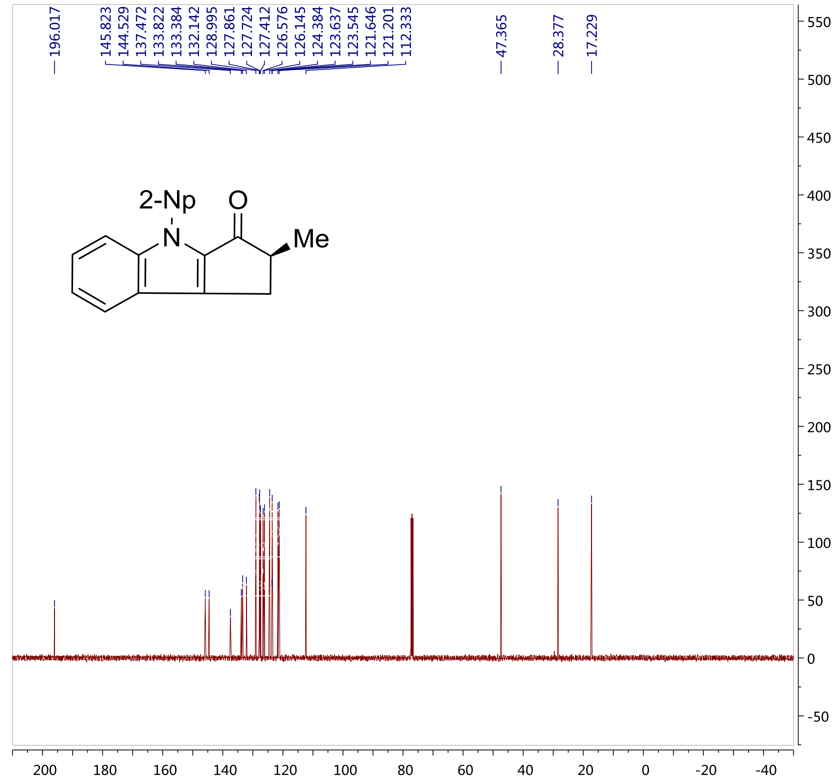

Parameters	
Parameter	Value
Title	wgp-16-104-A
Comment	test
Origin	Bruker BioSpin GmbH
Owner	nmr
Site	
Spectrometer	spect
Author	
Solvent	CDCl3
Temperature	293.5
Pulse Sequence	zgpg30
Experiment	1D
Number of Scans	79
Receiver Gain	55
Relaxation Delay	1.0000
Pulse Width	9.8000
Acquisition Time	0.8039
Acquisition Date	2016-04-10T13:21:40
Modification Date	2016-04-10T13:23:00
Spectrometer Frequency	100.61
Spectral Width	40760.9
Lowest Frequency	-10320.1
Nucleus	13C
Acquired Size	32768
Spectral Size	65536

(+)-2-ethyl-4-methyl-1,4-dihydrocyclopenta[b]indol-3(2H)-one (3i)

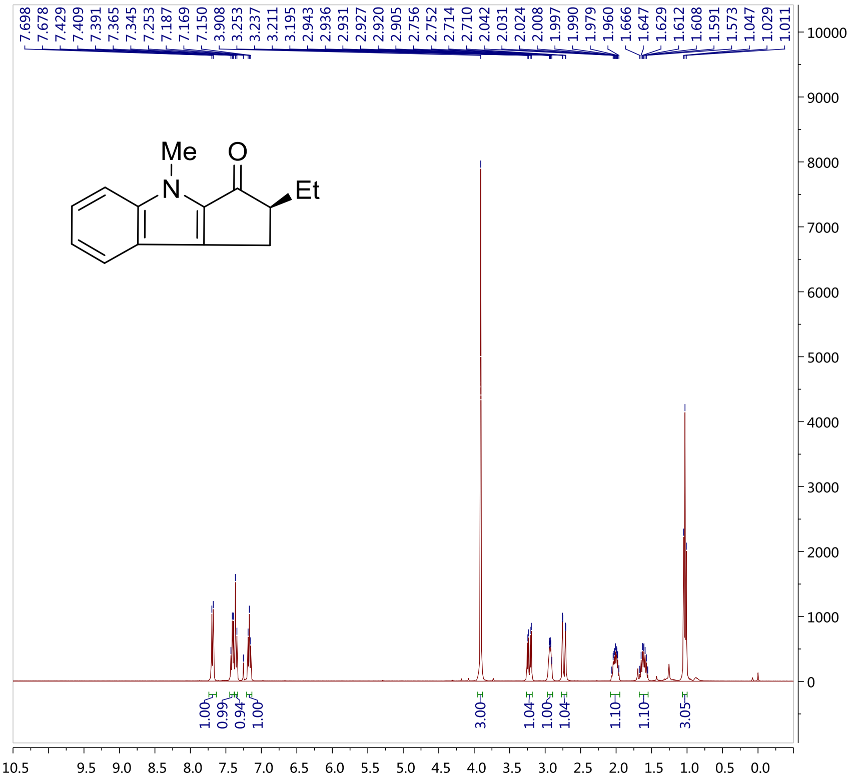

Parameters	
Parameter	Value
Title	wgp-14-191
Comment	PROTON
Origin	Bruker BioSpin GmbH
Owner	nmr
Site	
Spectrometer	spect
Author	
Solvent	CDCl3
Temperature	295.6
Pulse Sequence	zg30
Experiment	1D
Number of Scans	8
Receiver Gain	101
Relaxation Delay	1.0000
Pulse Width	13.7000
Acquisition Time	1.9999
Acquisition Date	2015-03-24T21:32:00
Modification Date	2015-03-24T21:32:00
Spectrometer Frequency	400.13
Spectral Width	8223.7
Lowest Frequency	-1640.9
Nucleus	1H
Acquired Size	16446
Spectral Size	65536

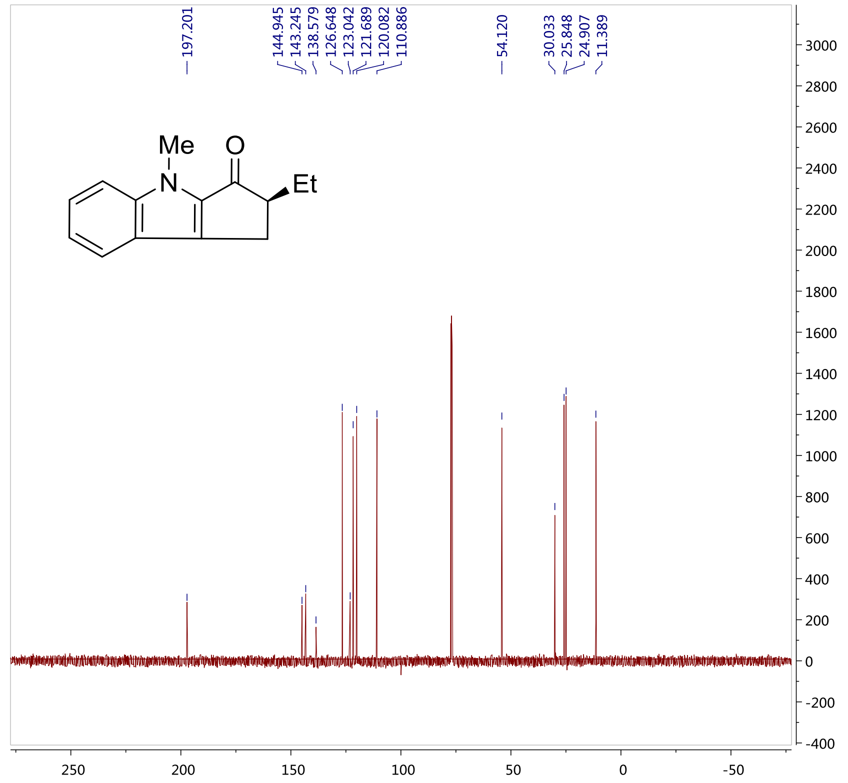

Parameters	
Parameter	Value
Title	wgp-14-191
Comment	C13CPD
Origin	Bruker BioSpin GmbH
Owner	nmr
Site	
Spectrometer	spect
Author	
Solvent	CDCl3
Temperature	296.1
Pulse Sequence	zgpg30
Experiment	1D
Number of Scans	274
Receiver Gain	203
Relaxation Delay	1.0000
Pulse Width	12.0000
Acquisition Time	0.4588
Acquisition Date	2015-03-24T21:34:00
Modification Date	2015-03-24T21:40:00
Spectrometer Frequency	100.61
Spectral Width	35714.3
Lowest Frequency	-7796.3
Nucleus	13C
Acquired Size	16384
Spectral Size	32768

(+)-2-isopropyl-4-methyl-1,4-dihydrocyclopenta[b]indol-3(2H)-one (3j)

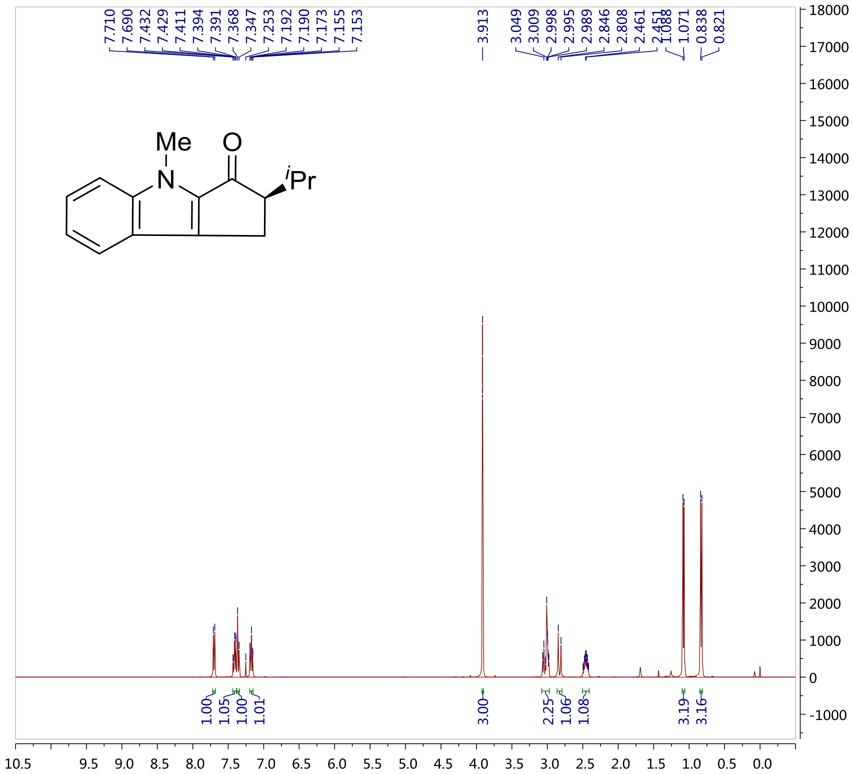

Parameters	
Parameter	Value
Title	wgp-15-192-B
Comment	PROTON
Origin	Bruker BioSpin GmbH
Owner	nmr
Site	
Spectrometer	spect
Author	
Solvent	CDCl3
Temperature	295.9
Pulse Sequence	zg30
Experiment	1D
Number of Scans	8
Receiver Gain	87
Relaxation Delay	1.0000
Pulse Width	15.0000
Acquisition Time	2.4999
Acquisition Date	2015-11-28T18:36:33
Modification Date	2015-11-28T18:36:00
Spectrometer Frequency	400.13
Spectral Width	8012.8
Lowest Frequency	-1535.4
Nucleus	1H
Acquired Size	20031
Spectral Size	65536

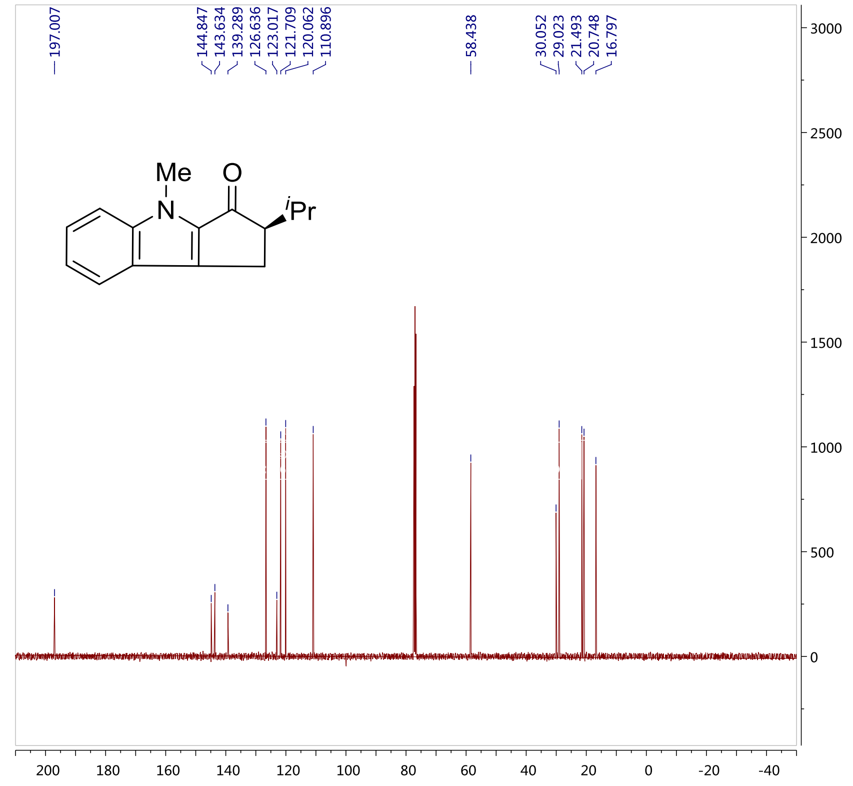

Parameters	
Parameter	Value
Title	wgp-15-192-B
Comment	C13CPD
Origin	Bruker BioSpin GmbH
Owner	nmr
Site	
Spectrometer	spect
Author	
Solvent	CDCl3
Temperature	296.3
Pulse Sequence	zgpg30
Experiment	1D
Number of Scans	516
Receiver Gain	87
Relaxation Delay	1.0000
Pulse Width	9.8000
Acquisition Time	0.4588
Acquisition Date	2015-11-28T18:38:22
Modification Date	2015-11-28T18:50:00
Spectrometer Frequency	100.61
Spectral Width	35714.3
Lowest Frequency	-7796.8
Nucleus	13C
Acquired Size	16384
Spectral Size	32768

(+)-4-methyl-2-phenyl-1,4-dihydrocyclopenta[b]indol-3(2H)-one (3k)

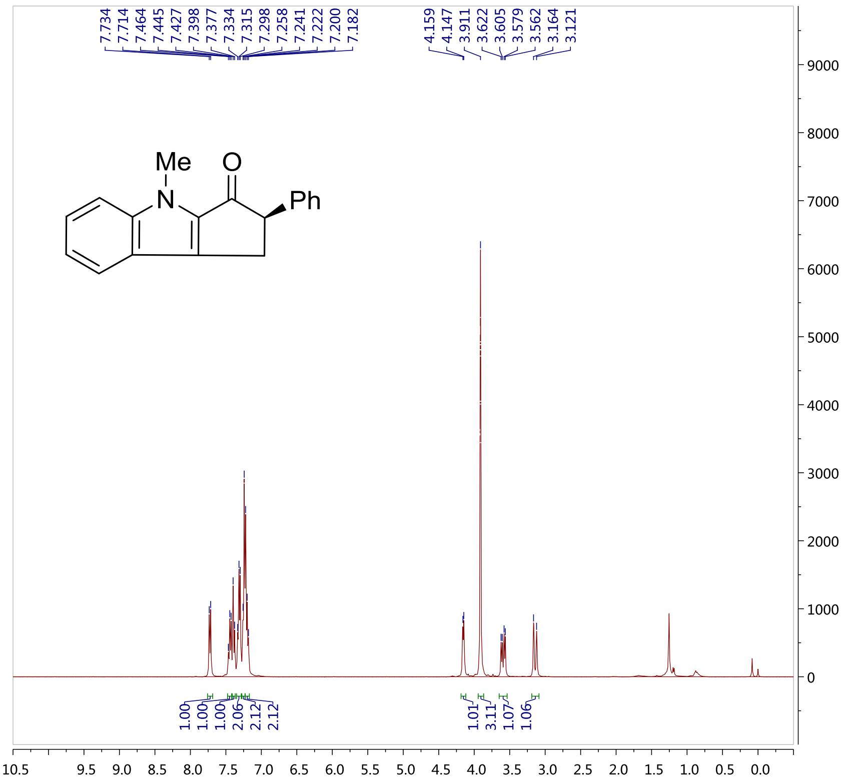

Parameters	
Parameter	Value
Title	wgp-14-12-A
Comment	PROTON
Origin	Bruker BioSpin GmbH
Owner	common
Site	
Spectrometer	spect
Author	
Solvent	CDCl3
Temperature	295.4
Pulse Sequence	zg30
Experiment	1D
Number of Scans	8
Receiver Gain	101
Relaxation Delay	1.0000
Pulse Width	13.7000
Acquisition Time	1.9999
Acquisition Date	2016-03-18T14:38:00
Modification Date	2016-03-18T14:38:00
Spectrometer Frequency	400.13
Spectral Width	8223.7
Lowest Frequency	-1640.9
Nucleus	1H
Acquired Size	16446
Spectral Size	65536

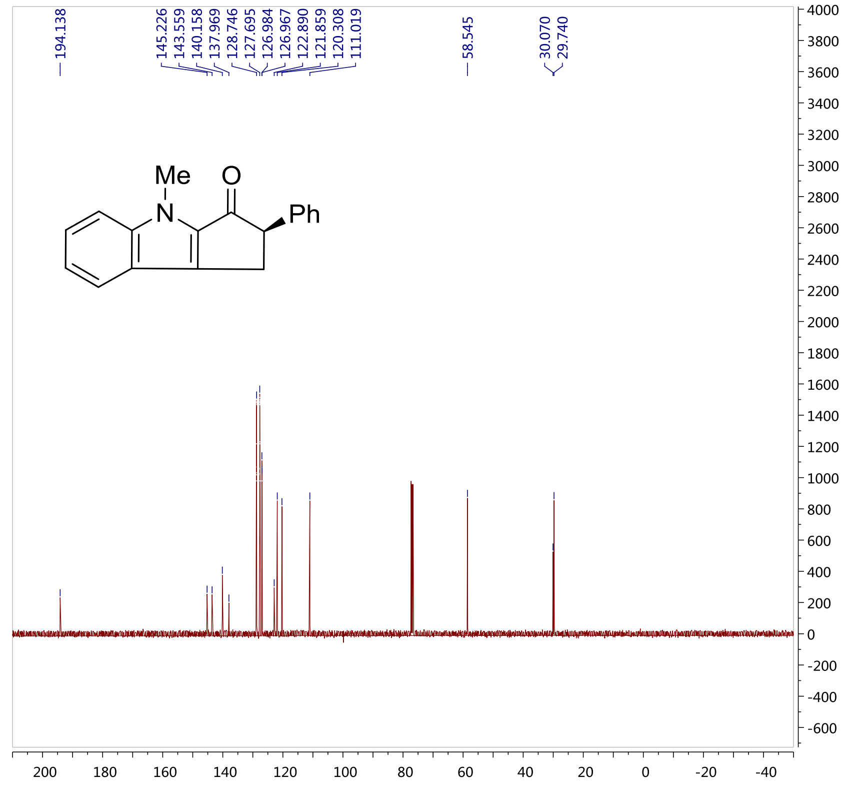

Parameters	
Parameter	Value
Title	wgp-14-12-A
Comment	C13CPD
Origin	Bruker BioSpin GmbH
Owner	common
Site	
Spectrometer	spect
Author	
Solvent	CDCl3
Temperature	295.5
Pulse Sequence	zgpg30
Experiment	1D
Number of Scans	205
Receiver Gain	203
Relaxation Delay	1.0000
Pulse Width	12.0000
Acquisition Time	0.4588
Acquisition Date	2016-03-18T14:40:00
Modification Date	2016-03-18T14:44:00
Spectrometer Frequency	100.61
Spectral Width	35714.3
Lowest Frequency	-7795.9
Nucleus	13C
Acquired Size	16384
Spectral Size	32768

(+)-2-(4-methoxyphenyl)-4-methyl-1,4-dihydrocyclopenta[b]indol-3(2H)-one (3l)

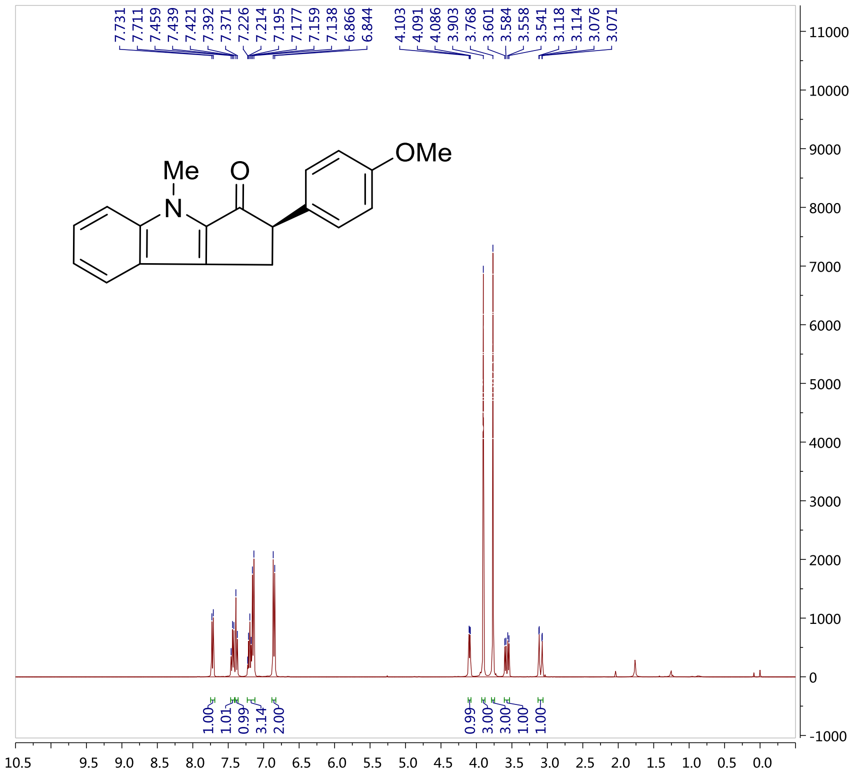

Parameter	Value
1 Data File Name	E:/ 0 NMR & MS/ 吲哚底物/ wgp-17-20-A/ 1/ fid
2 Title	wgp-17-20-A
3 Comment	PROTON
4 Origin	Bruker BioSpin GmbH
5 Owner	nmr
6 Site	
7 Spectrometer	spect
8 Author	
9 Solvent	CDCl3
10 Temperature	292.0
11 Pulse Sequence	zg30
12 Experiment	1D
13 Number of Scans	8
14 Receiver Gain	36
15 Relaxation Delay	1.0000
16 Pulse Width	9.1200
17 Acquisition Time	4.0894
18 Acquisition Date	2016-08-22T10:55:11
19 Modification Date	2016-08-22T10:55:00
20 Spectrometer Frequency	400.13
21 Spectral Width	8012.8
22 Lowest Frequency	-1535.4
23 Nucleus	1H
24 Acquired Size	32768
25 Spectral Size	65536

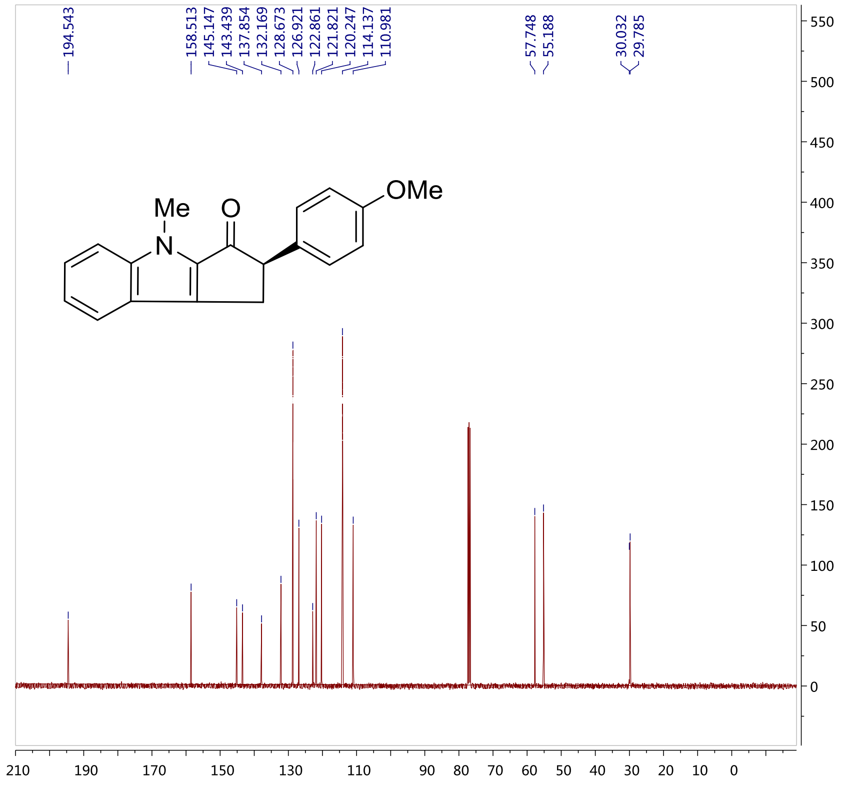

Parameter	Value
1 Data File Name	E:/ 0 NMR & MS/ 吲哚底物/ wgp-17-20-A/ 2/ fid
2 Title	wgp-17-20-A
3 Comment	C13CPD
4 Origin	Bruker BioSpin GmbH
5 Owner	nmr
6 Site	
7 Spectrometer	spect
8 Author	
9 Solvent	CDCl3
10 Temperature	292.5
11 Pulse Sequence	zgpg30
12 Experiment	1D
13 Number of Scans	142
14 Receiver Gain	33
15 Relaxation Delay	2.0000
16 Pulse Width	9.4000
17 Acquisition Time	1.3631
18 Acquisition Date	2016-08-22T10:56:40
19 Modification Date	2016-08-22T11:04:00
20 Spectrometer Frequency	100.61
21 Spectral Width	24038.5
22 Lowest Frequency	-1958.9
23 Nucleus	13C
24 Acquired Size	32768
25 Spectral Size	65536

(+)-2-(4-fluorophenyl)-4-methyl-1,4-dihydrocyclopenta[b]indol-3(2H)-one (3m)

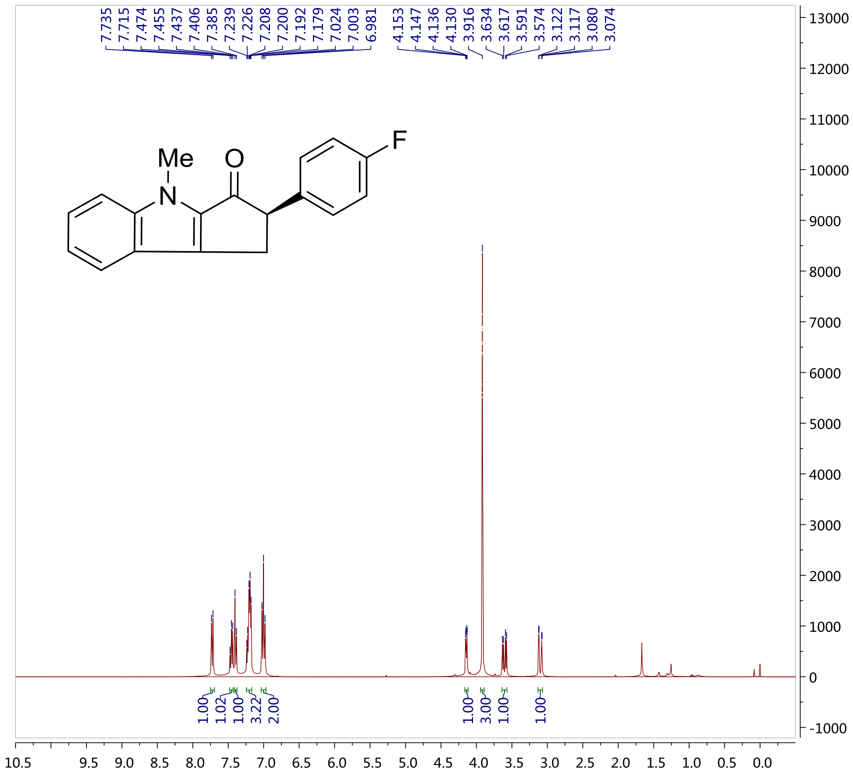

Parameter	Value
1 Data File Name	E:/ 20160730/ 0 NMR & MS/ 吲哚底物/ cmq-1-36-B/ 1/ fid
2 Title	cmq-1-36-B
3 Comment	PROTON
4 Origin	Bruker BioSpin GmbH
5 Owner	common
6 Site	
7 Spectrometer	spect
8 Author	
9 Solvent	CDCl3
10 Temperature	298.7
11 Pulse Sequence	zg30
12 Experiment	1D
13 Number of Scans	8
14 Receiver Gain	101
15 Relaxation Delay	1.0000
16 Pulse Width	13.7000
17 Acquisition Time	1.9999
18 Acquisition Date	2016-08-03T21:02:00
19 Modification Date	2016-08-03T21:03:00
20 Spectrometer Frequency	400.13
21 Spectral Width	8223.7
22 Lowest Frequency	-1640.9
23 Nucleus	1H
24 Acquired Size	16446
25 Spectral Size	65536

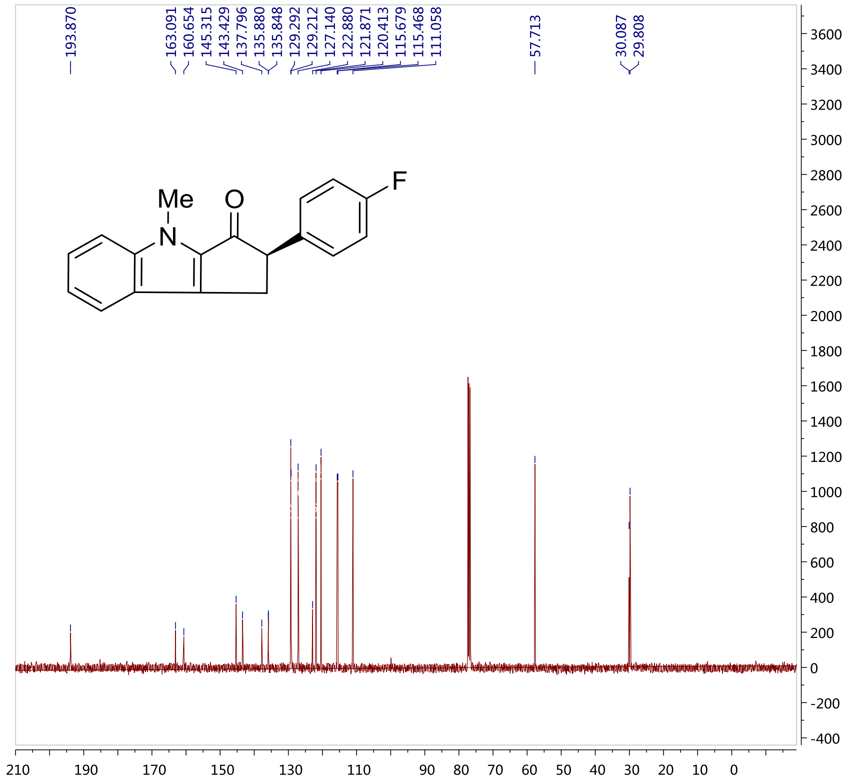

Parameter	Value
1 Data File Name	E:/ 20160730/ 0 NMR & MS/ 吲哚底物/ cmq-1-36-B/ 2/ fid
2 Title	cmq-1-36-B
3 Comment	C13CPD
4 Origin	Bruker BioSpin GmbH
5 Owner	common
6 Site	
7 Spectrometer	spect
8 Author	
9 Solvent	CDCl3
10 Temperature	299.0
11 Pulse Sequence	zgpg30
12 Experiment	1D
13 Number of Scans	312
14 Receiver Gain	203
15 Relaxation Delay	1.0000
16 Pulse Width	12.0000
17 Acquisition Time	0.4588
18 Acquisition Date	2016-08-03T21:05:00
19 Modification Date	2016-08-03T21:12:00
20 Spectrometer Frequency	100.61
21 Spectral Width	35714.3
22 Lowest Frequency	-7795.9
23 Nucleus	13C
24 Acquired Size	16384
25 Spectral Size	32768

(+)-2-(4-chlorophenyl)-4-methyl-1,4-dihydrocyclopenta[b]indol-3(2H)-one (3n)

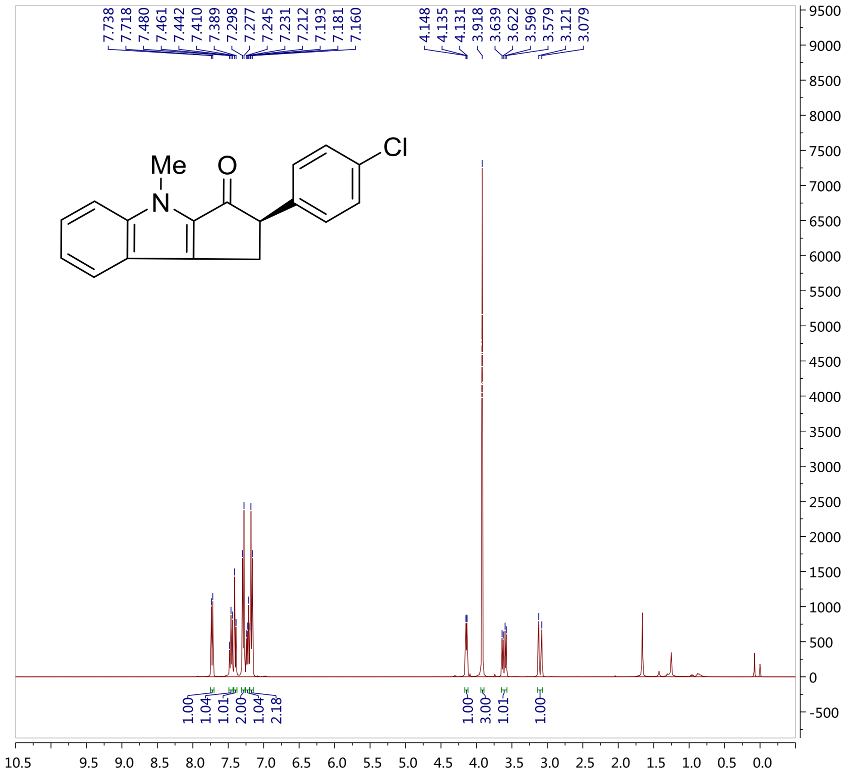

Parameter		Value
1	Data File Name	E:/ 20160730/ 0 NMR & MS/ 吲哚底物/ cmq-1-36-A/ 1/ fid
2	Title	cmq-1-36-A
3	Comment	PROTON
4	Origin	Bruker BioSpin GmbH
5	Owner	nmr
6	Site	
7	Spectrometer	spect
8	Author	
9	Solvent	CDCl3
10	Temperature	292.3
11	Pulse Sequence	zg30
12	Experiment	1D
13	Number of Scans	8
14	Receiver Gain	71
15	Relaxation Delay	1.0000
16	Pulse Width	9.1200
17	Acquisition Time	1.9923
18	Acquisition Date	2016-08-04T09:03:00
19	Modification Date	2016-08-04T10:10:00
20	Spectrometer Frequency	400.13
21	Spectral Width	8012.8
22	Lowest Frequency	-1535.4
23	Nucleus	1H
24	Acquired Size	32768
25	Spectral Size	65536

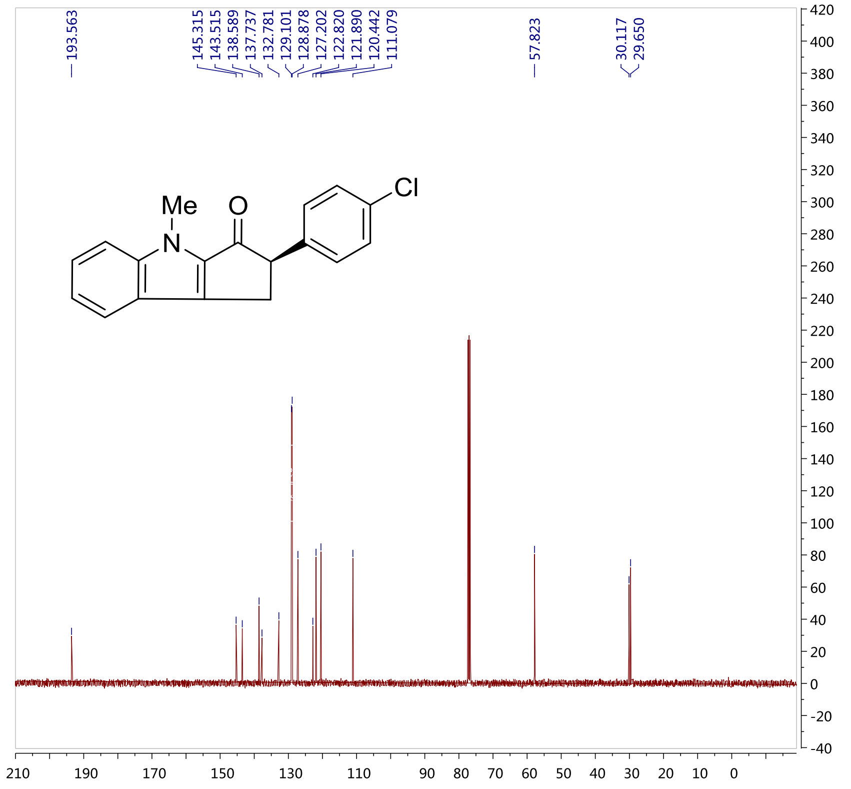

Parameter		Value
1	Data File Name	E:/ 20160730/ 0 NMR & MS/ 吲哚底物/ cmq-1-36-A/ 2/ fid
2	Title	cmq-1-36-A
3	Comment	C13CPD
4	Origin	Bruker BioSpin GmbH
5	Owner	nmr
6	Site	
7	Spectrometer	spect
8	Author	
9	Solvent	CDCl3
10	Temperature	292.7
11	Pulse Sequence	zgpg30
12	Experiment	1D
13	Number of Scans	187
14	Receiver Gain	27
15	Relaxation Delay	2.0000
16	Pulse Width	9.4000
17	Acquisition Time	0.5506
18	Acquisition Date	2016-08-04T09:17:00
19	Modification Date	2016-08-04T10:22:00
20	Spectrometer Frequency	100.61
21	Spectral Width	24038.5
22	Lowest Frequency	-1958.9
23	Nucleus	13C
24	Acquired Size	32768
25	Spectral Size	65536

(+)-2-(benzo[d][1,3]dioxol-5-yl)-4-methyl-1,4-dihydrocyclopenta[b]indol-3(2H)-one (3o)

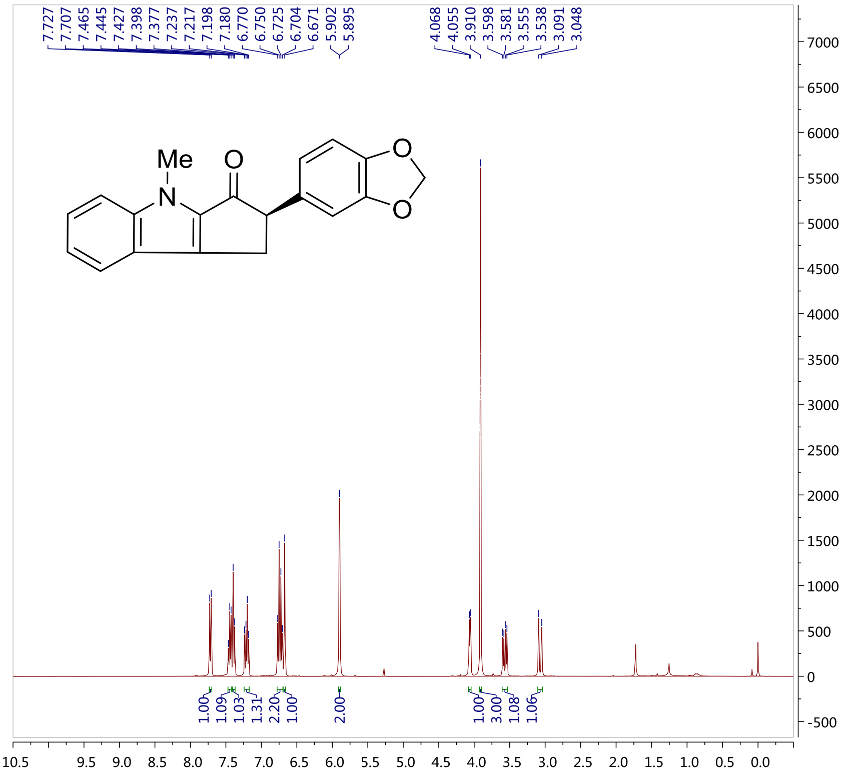

Parameter	Value
1 Data File Name	E:/ 0 NMR & MS/ 吲哚底物/ wgp-17-24-A/ 1/ fid
2 Title	wgp-17-24-A
3 Comment	PROTON
4 Origin	Bruker BioSpin GmbH
5 Owner	nmr
6 Site	
7 Spectrometer	spect
8 Author	
9 Solvent	CDCl3
10 Temperature	292.3
11 Pulse Sequence	zg30
12 Experiment	1D
13 Number of Scans	8
14 Receiver Gain	41
15 Relaxation Delay	1.0000
16 Pulse Width	9.1200
17 Acquisition Time	4.0894
18 Acquisition Date	2016-08-25T08:43:48
19 Modification Date	2016-08-25T08:43:00
20 Spectrometer Frequency	400.13
21 Spectral Width	8012.8
22 Lowest Frequency	-1535.4
23 Nucleus	1H
24 Acquired Size	32768
25 Spectral Size	65536

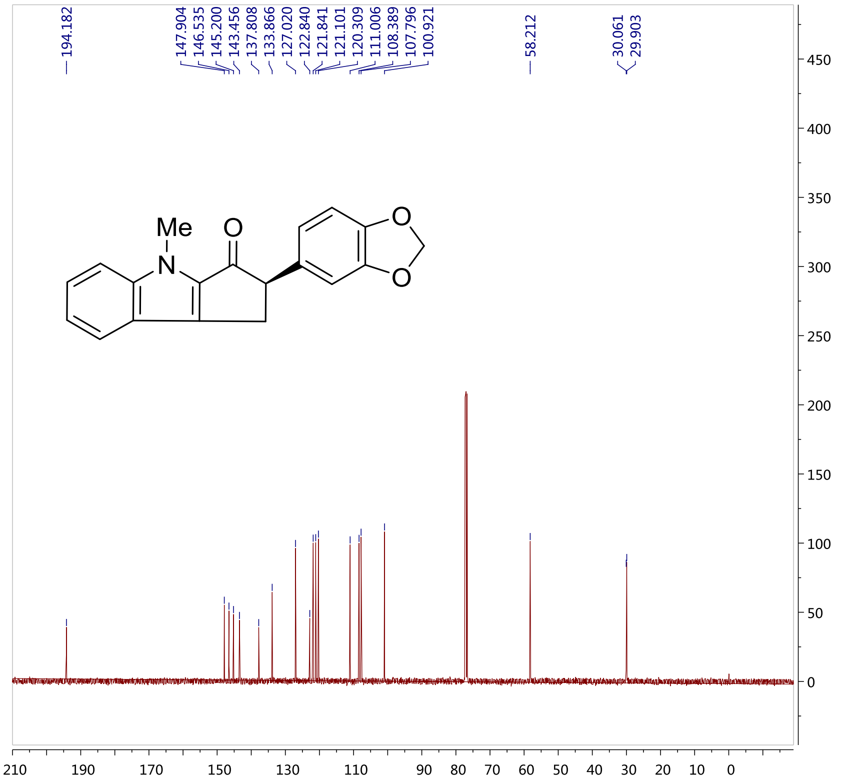

Parameter	Value
1 Data File Name	E:/ 0 NMR & MS/ 吲哚底物/ wgp-17-24-A/ 2/ fid
2 Title	wgp-17-24-A
3 Comment	C13CPD
4 Origin	Bruker BioSpin GmbH
5 Owner	nmr
6 Site	
7 Spectrometer	spect
8 Author	
9 Solvent	CDCl3
10 Temperature	292.8
11 Pulse Sequence	zgpg30
12 Experiment	1D
13 Number of Scans	131
14 Receiver Gain	33
15 Relaxation Delay	2.0000
16 Pulse Width	9.4000
17 Acquisition Time	1.3631
18 Acquisition Date	2016-08-25T08:45:40
19 Modification Date	2016-08-25T08:52:00
20 Spectrometer Frequency	100.61
21 Spectral Width	24038.5
22 Lowest Frequency	-1958.9
23 Nucleus	13C
24 Acquired Size	32768
25 Spectral Size	65536

(+)-2-isopropyl-4,7-dimethyl-1,4-dihydrocyclopenta[b]indol-3(2H)-one (3p)

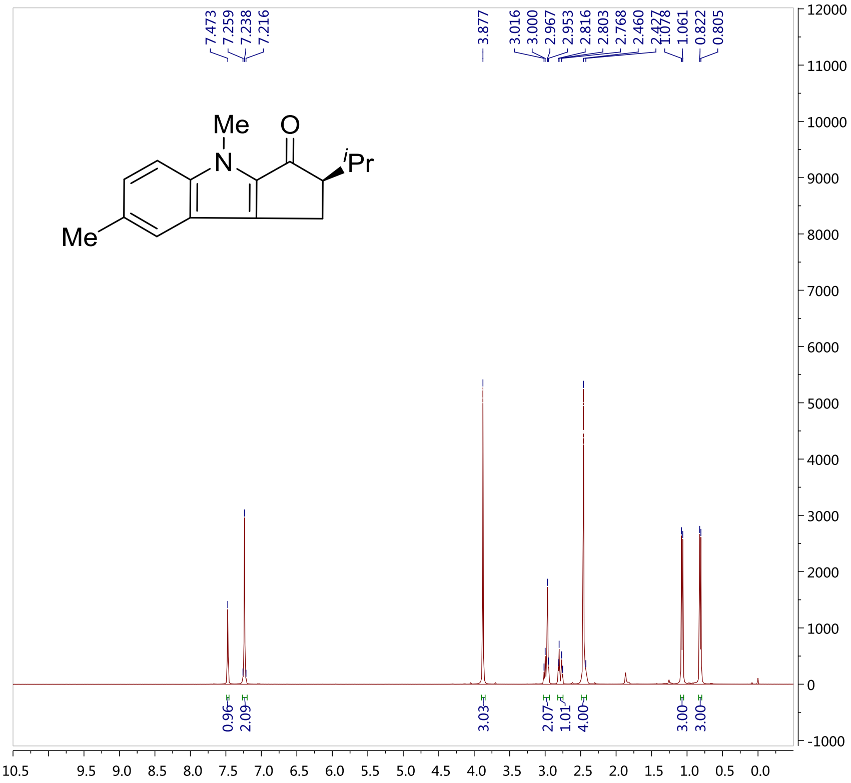

Parameter	Value
1 Data File Name	E:/ 0 NMR & MS/ 吲哚底物/ wgp-15-17-A/ 1/ fid
2 Title	wgp-15-17-A
3 Comment	PROTON
4 Origin	Bruker BioSpin GmbH
5 Owner	nmr
6 Site	
7 Spectrometer	spect
8 Author	
9 Solvent	CDCl3
10 Temperature	291.3
11 Pulse Sequence	zg30
12 Experiment	1D
13 Number of Scans	8
14 Receiver Gain	33
15 Relaxation Delay	1.0000
16 Pulse Width	9.1200
17 Acquisition Time	4.0894
18 Acquisition Date	2016-08-18T16:08:29
19 Modification Date	2016-08-18T16:08:00
20 Spectrometer Frequency	400.13
21 Spectral Width	8012.8
22 Lowest Frequency	-1535.4
23 Nucleus	1H
24 Acquired Size	32768
25 Spectral Size	65536

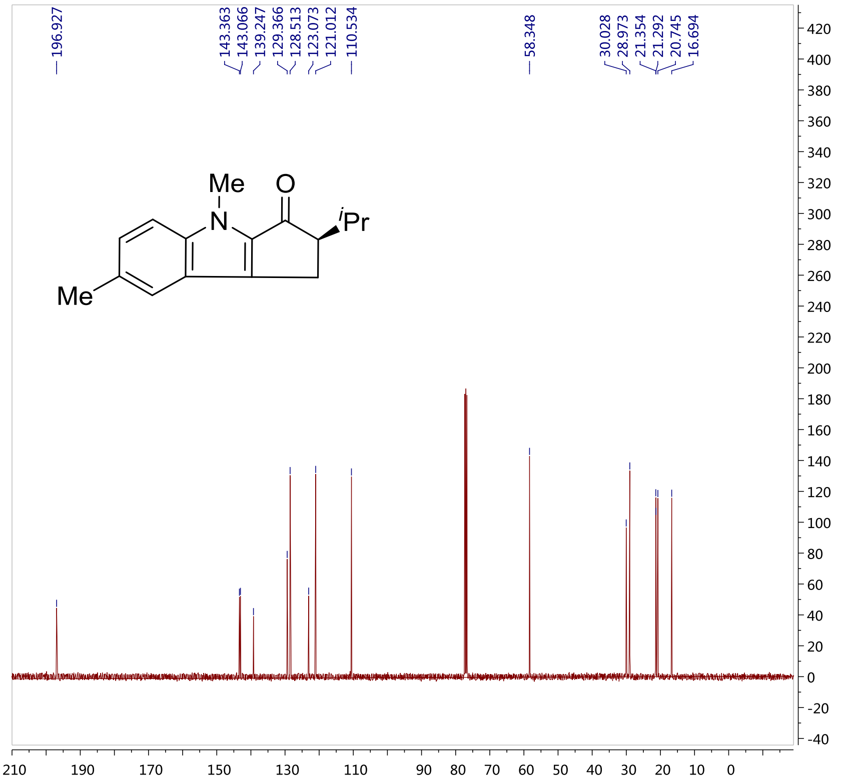

Parameter	Value
1 Data File Name	E:/ 0 NMR & MS/ 吲哚底物/ wgp-15-17-A/ 2/ fid
2 Title	wgp-15-17-A
3 Comment	C13CPD
4 Origin	Bruker BioSpin GmbH
5 Owner	nmr
6 Site	
7 Spectrometer	spect
8 Author	
9 Solvent	CDCl3
10 Temperature	291.8
11 Pulse Sequence	zgpg30
12 Experiment	1D
13 Number of Scans	159
14 Receiver Gain	29
15 Relaxation Delay	2.0000
16 Pulse Width	9.4000
17 Acquisition Time	1.3631
18 Acquisition Date	2016-08-18T16:10:24
19 Modification Date	2016-08-18T16:18:00
20 Spectrometer Frequency	100.61
21 Spectral Width	24038.5
22 Lowest Frequency	-1958.9
23 Nucleus	13C
24 Acquired Size	32768
25 Spectral Size	65536

(+)-7-fluoro-2-isopropyl-4-methyl-1,4-dihydrocyclopenta[b]indol-3(2H)-one (3q)

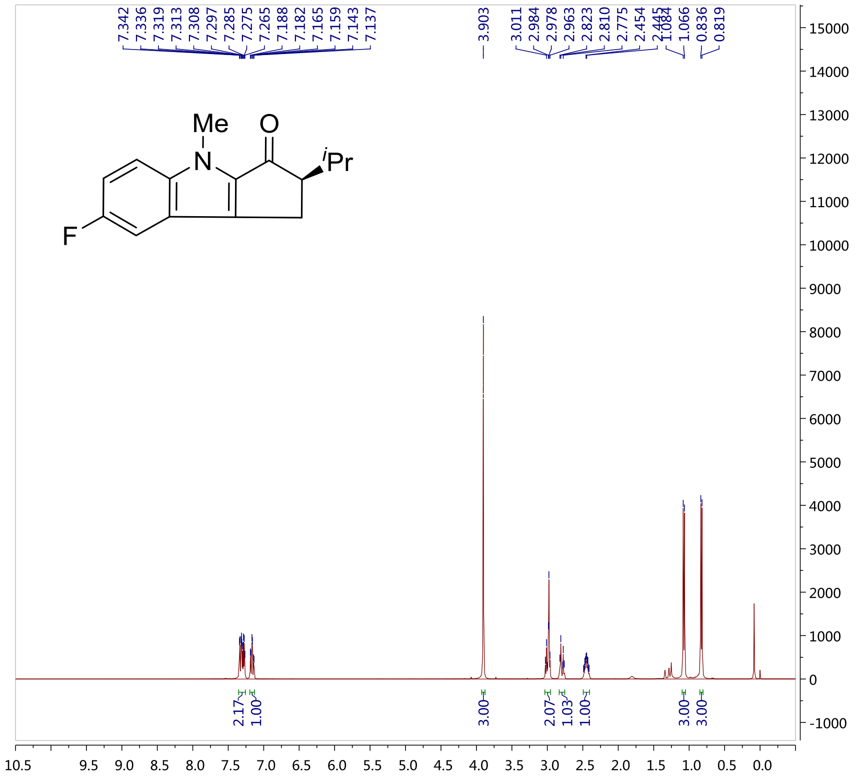

Parameter	Value
1 Data File Name	E:/ 0 Data/ 1 吲哚底物/ wgp-17-35-B/ 1/ fid
2 Title	wgp-17-35-B
3 Comment	PROTON
4 Origin	Bruker BioSpin GmbH
5 Owner	nmr
6 Site	
7 Spectrometer	spect
8 Author	
9 Solvent	CDCl3
10 Temperature	292.2
11 Pulse Sequence	zg30
12 Experiment	1D
13 Number of Scans	8
14 Receiver Gain	41
15 Relaxation Delay	1.0000
16 Pulse Width	9.1200
17 Acquisition Time	4.0894
18 Acquisition Date	2016-10-06T13:14:53
19 Modification Date	2016-10-06T13:14:00
20 Spectrometer Frequency	400.13
21 Spectral Width	8012.8
22 Lowest Frequency	-1576.6
23 Nucleus	1H
24 Acquired Size	32768
25 Spectral Size	65536

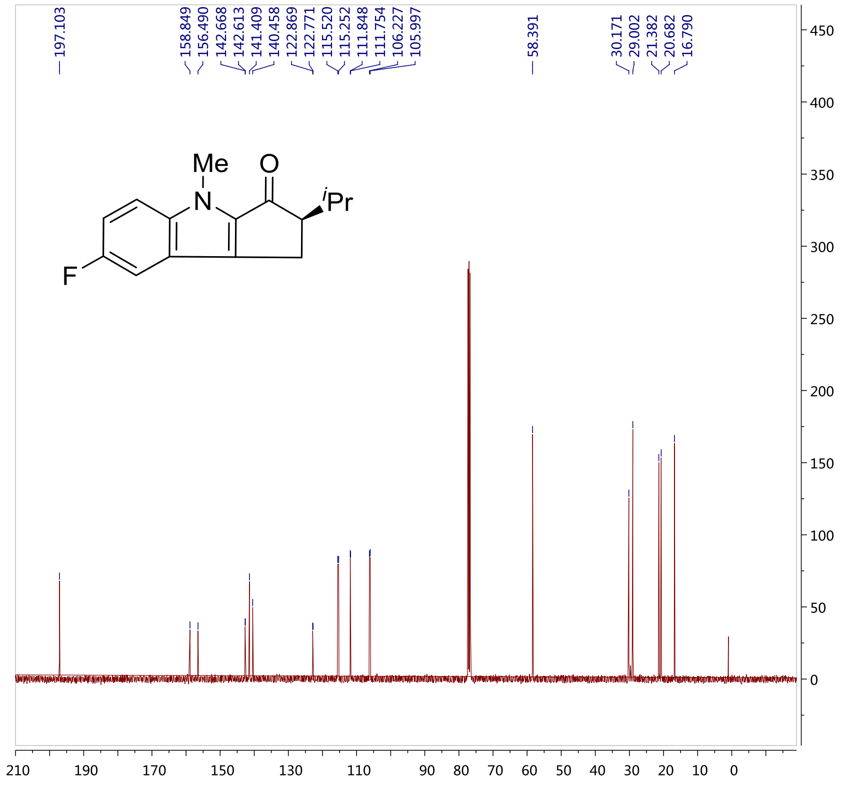

Parameter	Value
1 Data File Name	E:/ 0 Data/ 1 吲哚底物/ wgp-17-35-B/ 2/ fid
2 Title	wgp-17-35-B
3 Comment	C13CPD
4 Origin	Bruker BioSpin GmbH
5 Owner	nmr
6 Site	
7 Spectrometer	spect
8 Author	
9 Solvent	CDCl3
10 Temperature	292.8
11 Pulse Sequence	zgpg30
12 Experiment	1D
13 Number of Scans	259
14 Receiver Gain	27
15 Relaxation Delay	2.0000
16 Pulse Width	9.4000
17 Acquisition Time	1.3631
18 Acquisition Date	2016-10-06T13:17:05
19 Modification Date	2016-10-06T13:30:00
20 Spectrometer Frequency	100.61
21 Spectral Width	24038.5
22 Lowest Frequency	-2064.8
23 Nucleus	13C
24 Acquired Size	32768
25 Spectral Size	65536

(*R*)-(+)-7-chloro-2-isopropyl-4-methyl-1,4-dihydrocyclopenta[b]indol-3(2H)-one (3r)

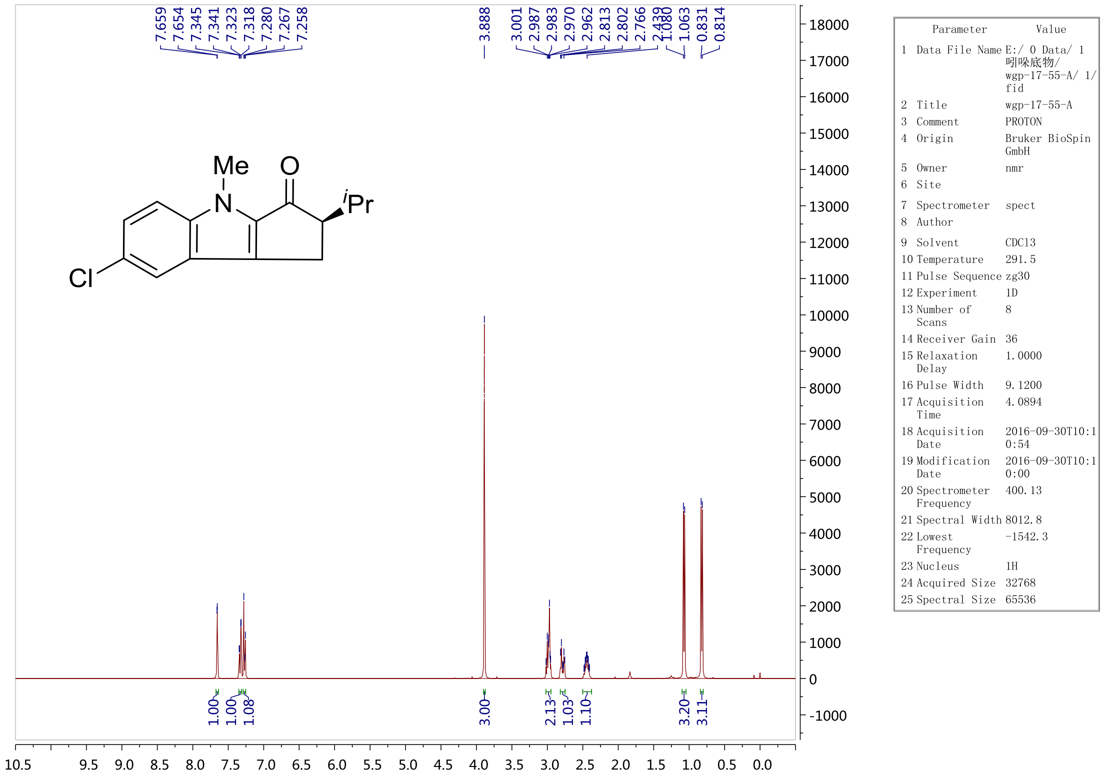

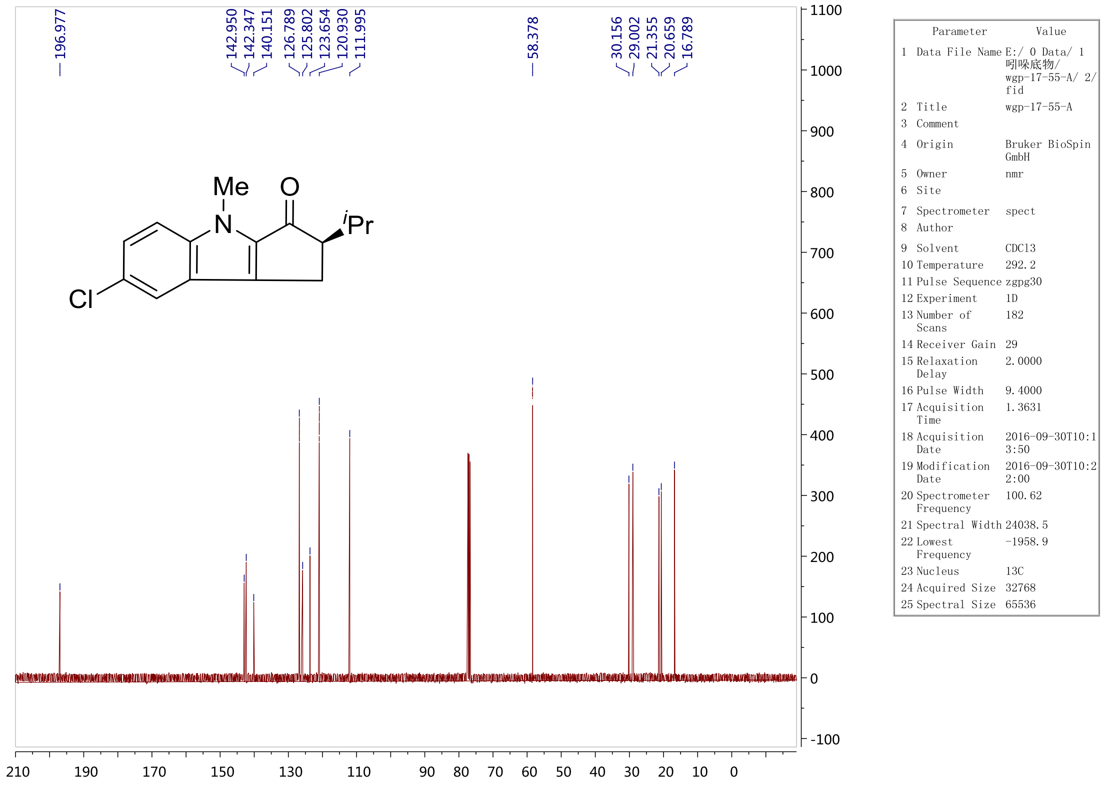

(+)-8-fluoro-2-isopropyl-4-methyl-1,4-dihydrocyclopenta[b]indol-3(2H)-one (3s)

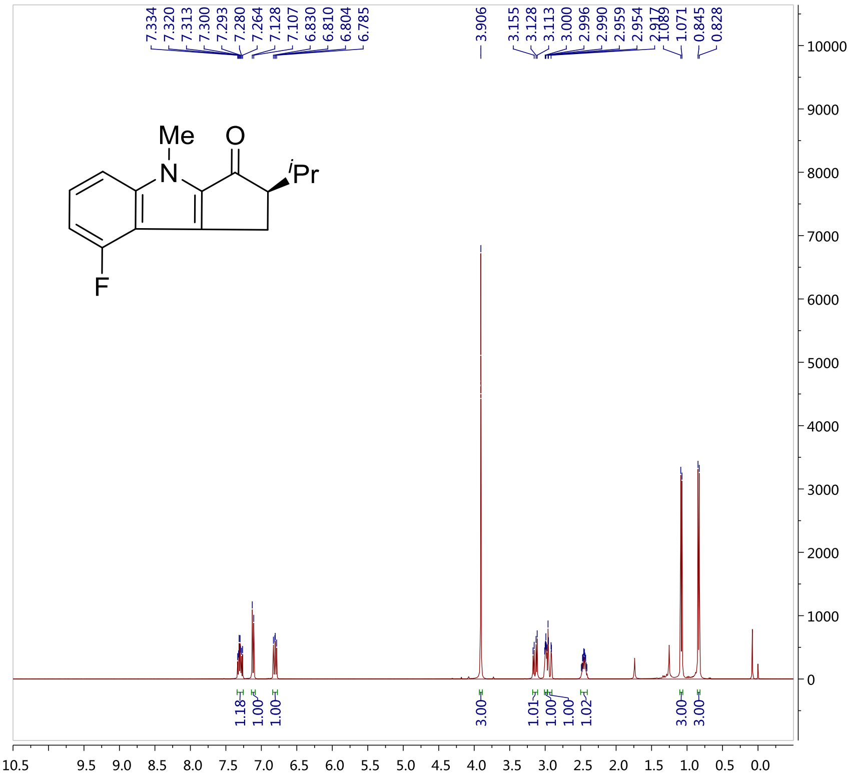

Parameter	Value
1 Data File Name	E:/ 0 Data/ 1 吲哚底物/ wgp-17-61-B/ 1/ fid
2 Title	wgp-17-61-B
3 Comment	PROTON
4 Origin	Bruker BioSpin GmbH
5 Owner	nmr
6 Site	
7 Spectrometer	spect
8 Author	
9 Solvent	CDCl3
10 Temperature	292.1
11 Pulse Sequence	zg30
12 Experiment	1D
13 Number of Scans	8
14 Receiver Gain	41
15 Relaxation Delay	1.0000
16 Pulse Width	9.1200
17 Acquisition Time	4.0894
18 Acquisition Date	2016-10-06T16:30:00
19 Modification Date	2016-10-06T16:30:00
20 Spectrometer Frequency	400.13
21 Spectral Width	8012.8
22 Lowest Frequency	-1575.6
23 Nucleus	1H
24 Acquired Size	32768
25 Spectral Size	65536

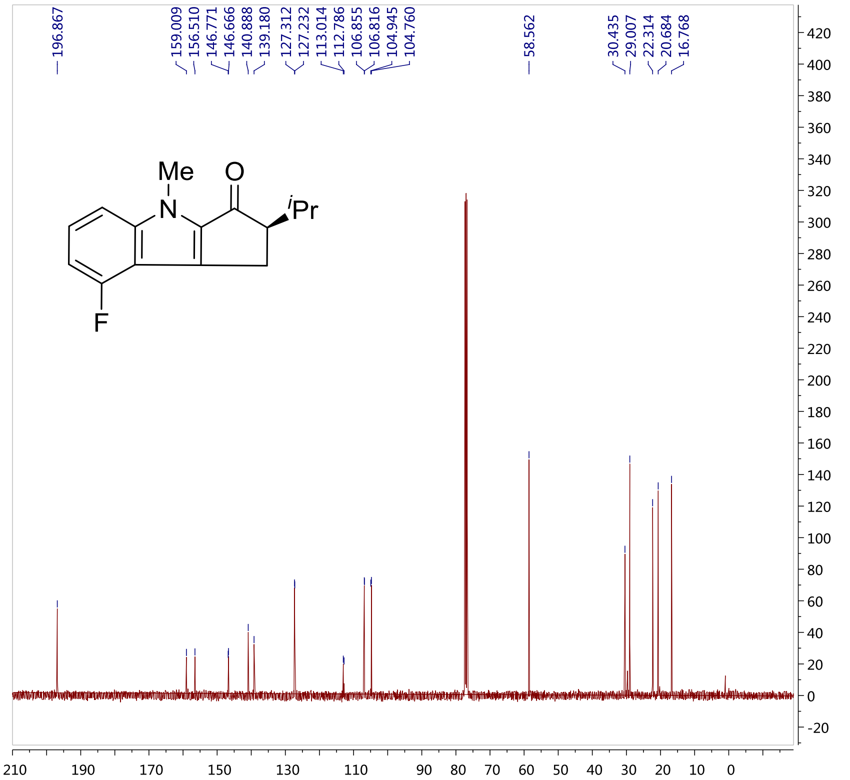

Parameter	Value
1 Data File Name	E:/ 0 Data/ 1 吲哚底物/ wgp-17-61-B/ 2/ fid
2 Title	wgp-17-61-B
3 Comment	C13CPD
4 Origin	Bruker BioSpin GmbH
5 Owner	nmr
6 Site	
7 Spectrometer	spect
8 Author	
9 Solvent	CDCl3
10 Temperature	292.9
11 Pulse Sequence	zgpg30
12 Experiment	1D
13 Number of Scans	226
14 Receiver Gain	33
15 Relaxation Delay	2.0000
16 Pulse Width	9.4000
17 Acquisition Time	1.3631
18 Acquisition Date	2016-10-06T16:34:01
19 Modification Date	2016-10-06T16:44:00
20 Spectrometer Frequency	100.61
21 Spectral Width	24038.5
22 Lowest Frequency	-1958.9
23 Nucleus	13C
24 Acquired Size	32768
25 Spectral Size	65536

(-)-4-(4-methoxyphenyl)-2-methyl-1,2,3,4-tetrahydrocyclopenta[b] indol-3-ol (4)

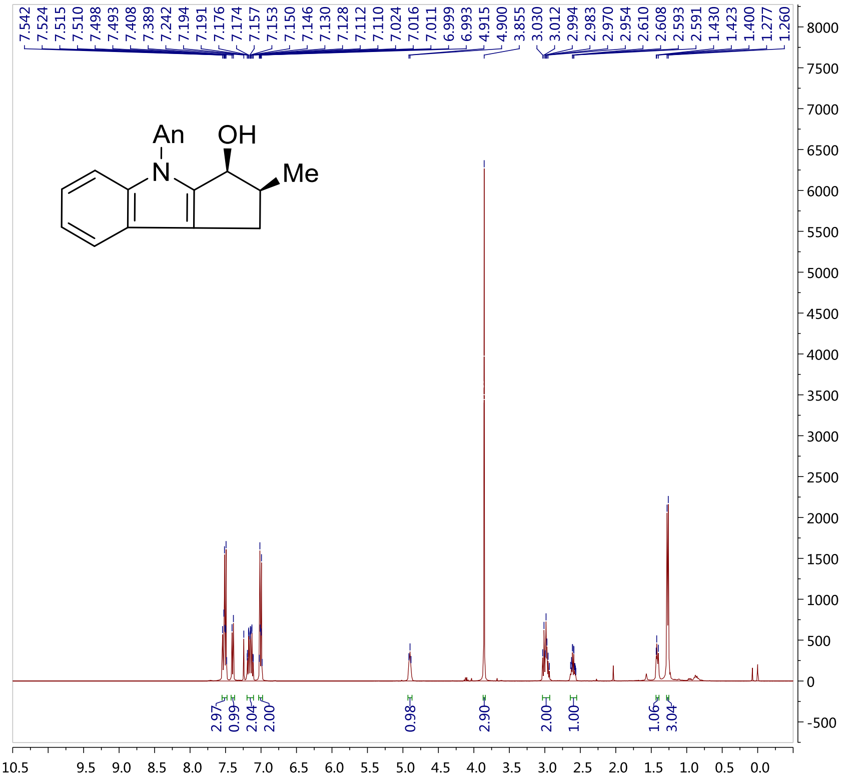

Parameter		Value
1	Data File Name	E:/ 0 Data/ 核磁原始数据/ 吲哚 转化/ wgp-17-95-1/ 1/ fid
2	Title	wgp-17-95-1
3	Comment	PROTON
4	Origin	Bruker BioSpin GmbH
5	Owner	nmr
6	Site	
7	Spectrometer	spect
8	Author	
9	Solvent	CDCl3
10	Temperature	0.0
11	Pulse Sequence	zg30
12	Experiment	1D
13	Number of Scans	8
14	Receiver Gain	57
15	Relaxation Delay	1.0000
16	Pulse Width	9.1200
17	Acquisition Time	4.0894
18	Acquisition Date	2016-12-27T13:39:01
19	Modification Date	2016-12-27T13:39:00
20	Spectrometer Frequency	400.13
21	Spectral Width	8012.8
22	Lowest Frequency	-1552.3
23	Nucleus	1H
24	Acquired Size	32768
25	Spectral Size	65536

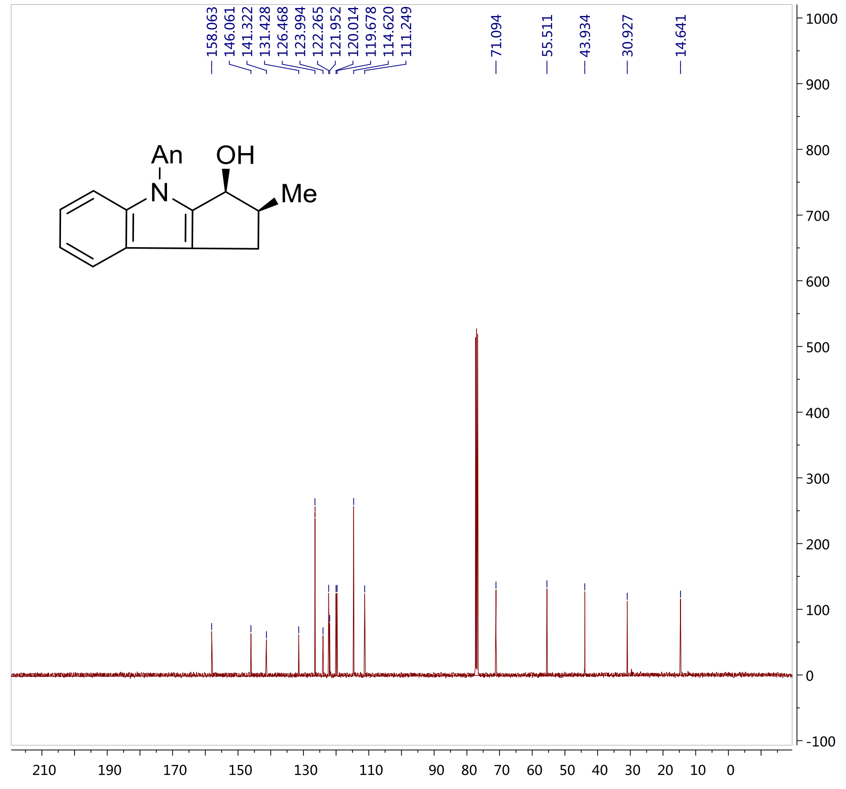

Parameter		Value
1	Data File Name	E:/ 0 Data/ 核磁原始数据/ 吲哚 转化/ wgp-17-95-1/ 2/ fid
2	Title	wgp-17-95-1
3	Comment	C13CPD
4	Origin	Bruker BioSpin GmbH
5	Owner	nmr
6	Site	
7	Spectrometer	spect
8	Author	
9	Solvent	CDCl3
10	Temperature	0.0
11	Pulse Sequence	zgpg30
12	Experiment	1D
13	Number of Scans	456
14	Receiver Gain	27
15	Relaxation Delay	2.0000
16	Pulse Width	9.4000
17	Acquisition Time	1.3631
18	Acquisition Date	2016-12-27T13:41:49
19	Modification Date	2016-12-27T14:05:00
20	Spectrometer Frequency	100.61
21	Spectral Width	24038.5
22	Lowest Frequency	-1958.9
23	Nucleus	13C
24	Acquired Size	32768
25	Spectral Size	65536

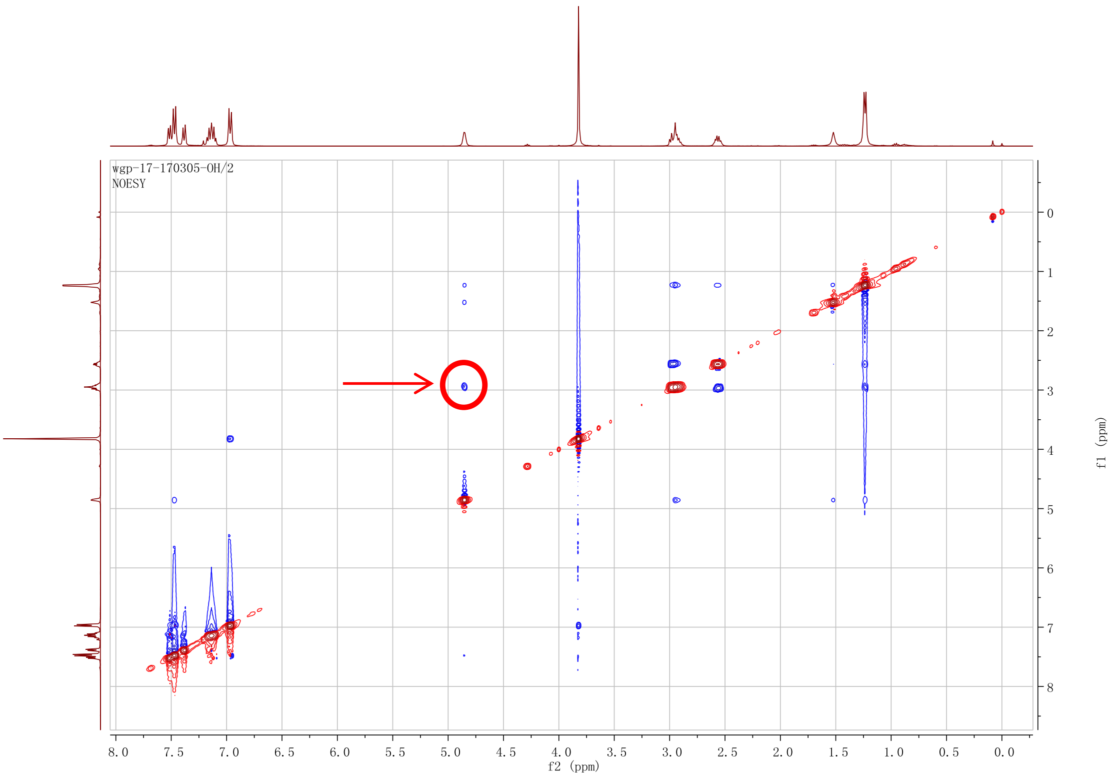

wgp-17-170305-0H/2
NOESY
f2 (ppm)
f1 (ppm)

9. HPLC Charts of Cyclization Products and Transformation Product

(+)-2,4-dimethyl-1,4-dihydrocyclopenta[b]indol-3(2H)-one (3a)

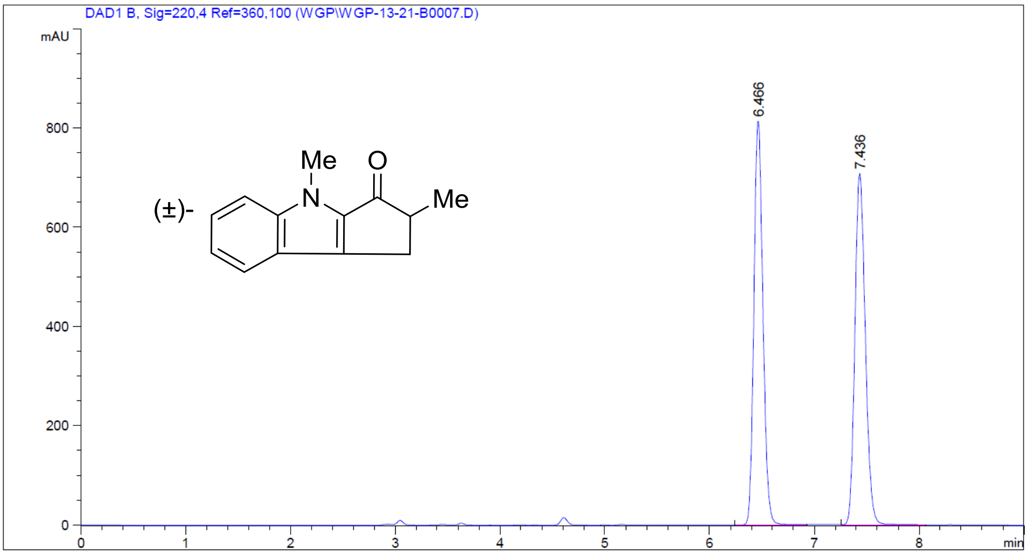

Peak #	RetTime [min]	Type	Width [min]	Area [mAU*s]	Height [mAU]	Area %
1	6.466	BB	0.0936	4867.17529	815.72778	49.8979
2	7.436	BB	0.1065	4887.09375	708.57227	50.1021

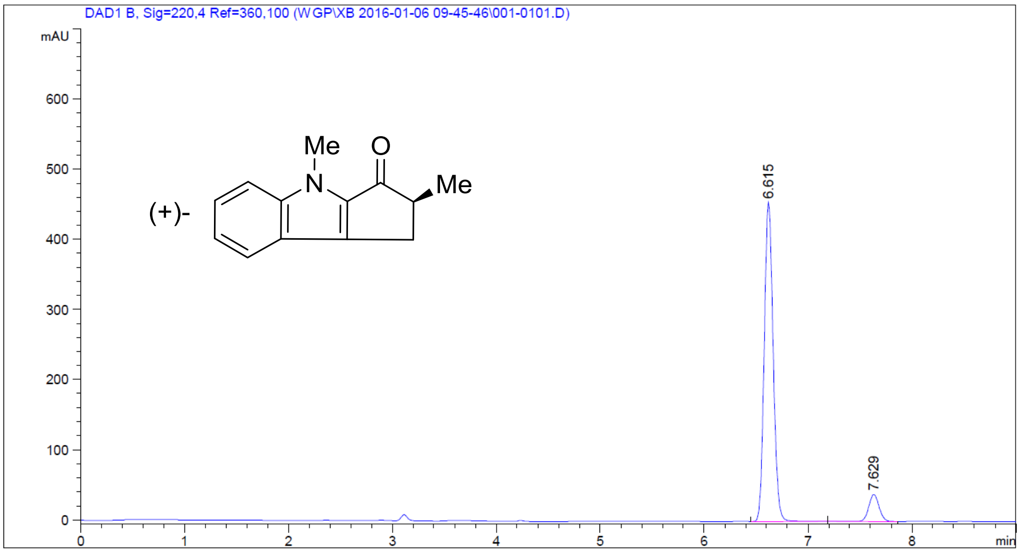

Peak #	RetTime [min]	Type	Width [min]	Area [mAU*s]	Height [mAU]	Area %
1	6.615	BB	0.0915	2709.63696	455.02417	90.9136
2	7.629	BB	0.1077	270.81601	38.67042	9.0864

(+)-4-isopropyl-2-methyl-1,4-dihydrocyclopenta[b]indol-3(2H)-one (3b)

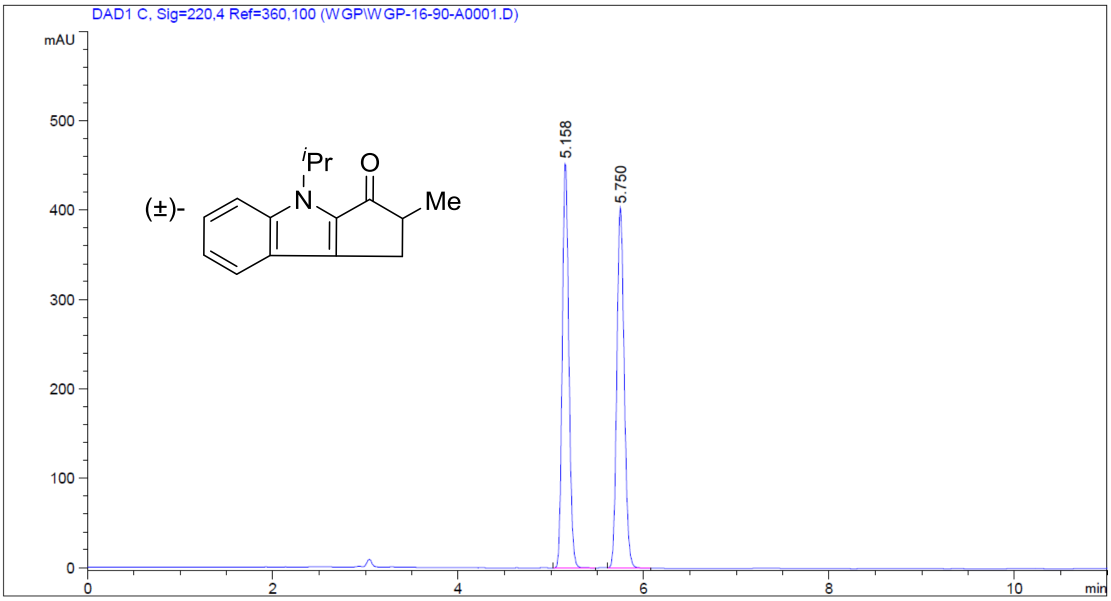

Peak #	RetTime [min]	Type	Width [min]	Area [mAU*s]	Height [mAU]	Area %
1	5.158	VV	0.0773	2231.38525	454.04486	49.9498
2	5.750	VB	0.0866	2235.86816	403.93549	50.0502

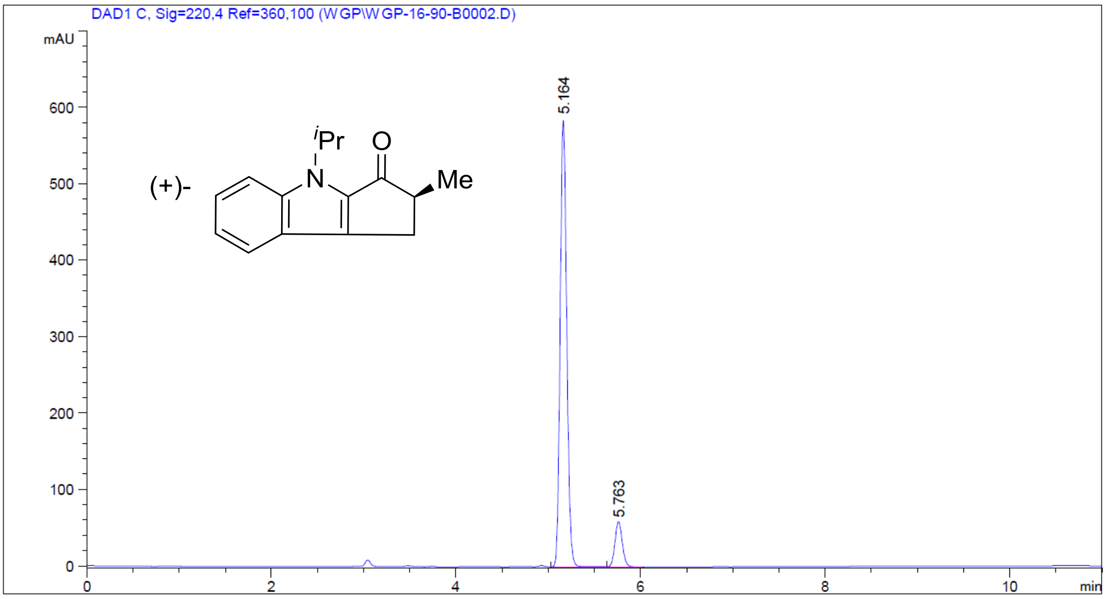

Peak #	RetTime [min]	Type	Width [min]	Area [mAU*s]	Height [mAU]	Area %
1	5.164	BV	0.0775	2891.86133	586.35291	89.8241
2	5.763	VB	0.0864	327.60858	59.36699	10.1759

(+)-4-allyl-2-methyl-1,4-dihydrocyclopenta[b]indol-3(2H)-one (3c)

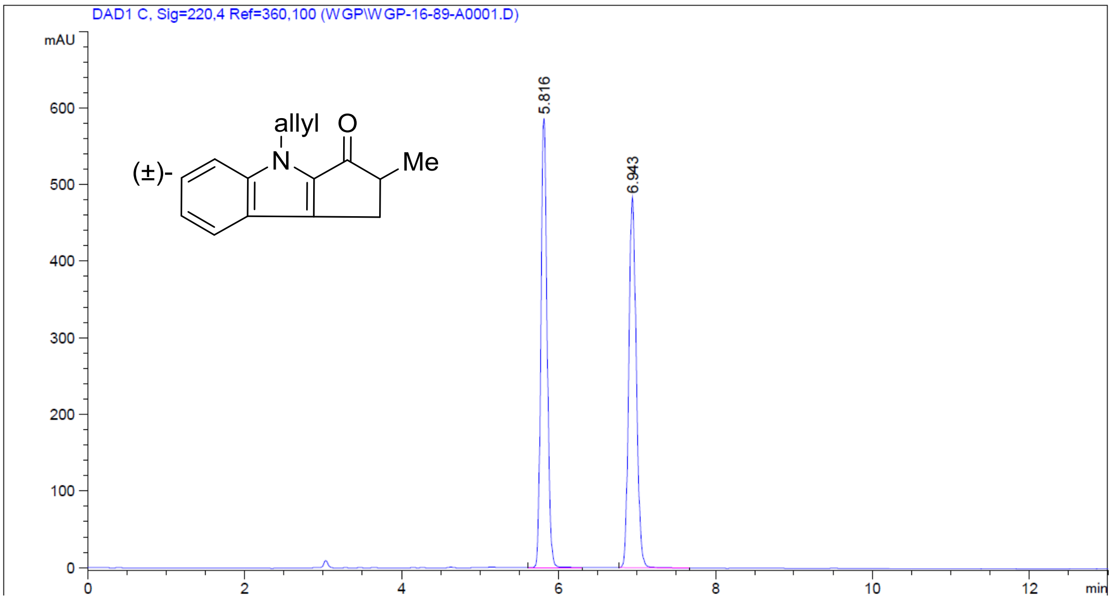

Peak #	RetTime [min]	Type	Width [min]	Area [mAU*s]	Height [mAU]	Area %
1	5.816	BB	0.0859	3213.46118	586.38086	49.9229
2	6.943	BB	0.1039	3223.38062	482.74057	50.0771

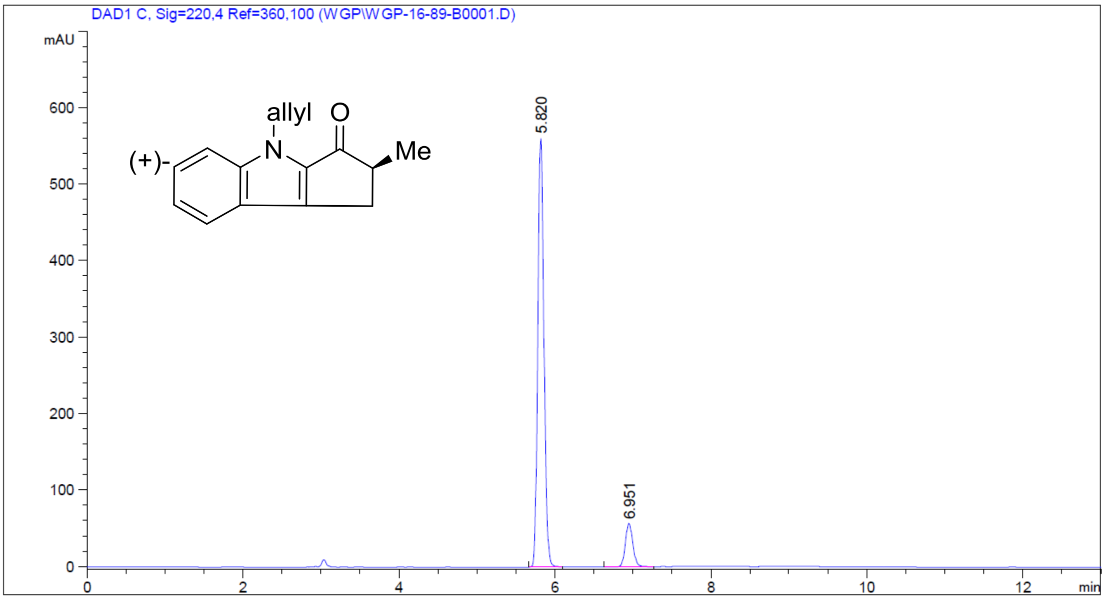

Peak #	RetTime [min]	Type	Width [min]	Area [mAU*s]	Height [mAU]	Area %
1	5.820	BB	0.0857	3063.91016	560.94293	89.0622
2	6.951	BB	0.1035	376.28085	56.63813	10.9378

(-)-4-benzyl-2-methyl-1,4-dihydrocyclopenta[b]indol-3(2H)-one (3d)

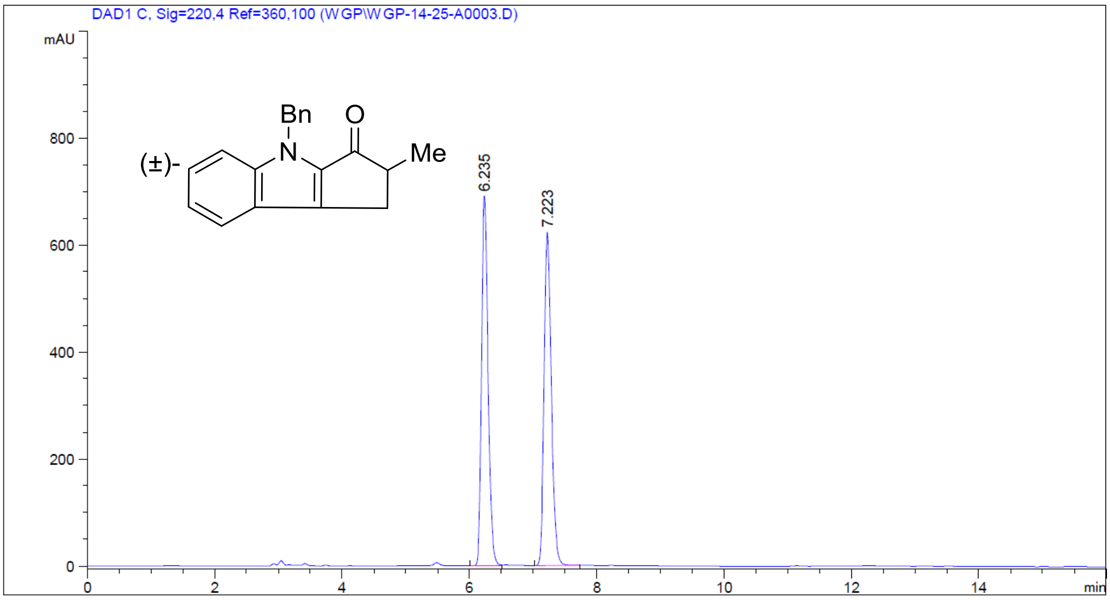

Peak #	RetTime [min]	Type	Width [min]	Area [mAU*s]	Height [mAU]	Area %
1	6.235	BV	0.1111	4934.35742	692.75580	49.9591
2	7.223	BB	0.1203	4942.43164	624.85785	50.0409

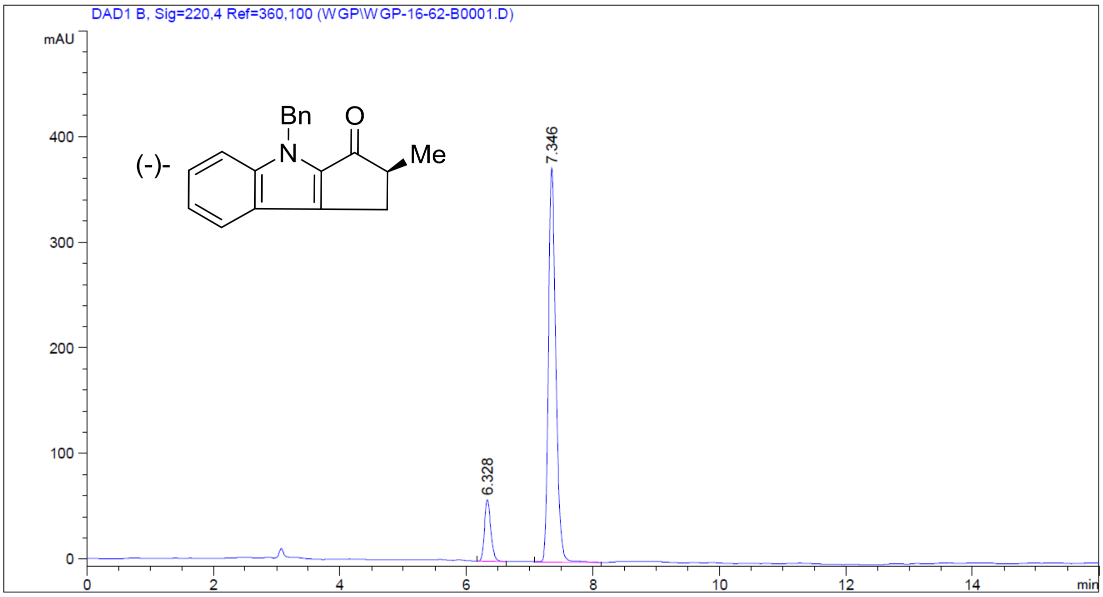

Peak #	RetTime [min]	Type	Width [min]	Area [mAU*s]	Height [mAU]	Area %
1	6.328	BB	0.1041	401.74609	58.48005	11.7360
2	7.346	BB	0.1245	3021.44604	373.21472	88.2640

(+)-2-methyl-4-phenyl-1,4-dihydrocyclopenta[b]indol-3(2H)-one (3e)

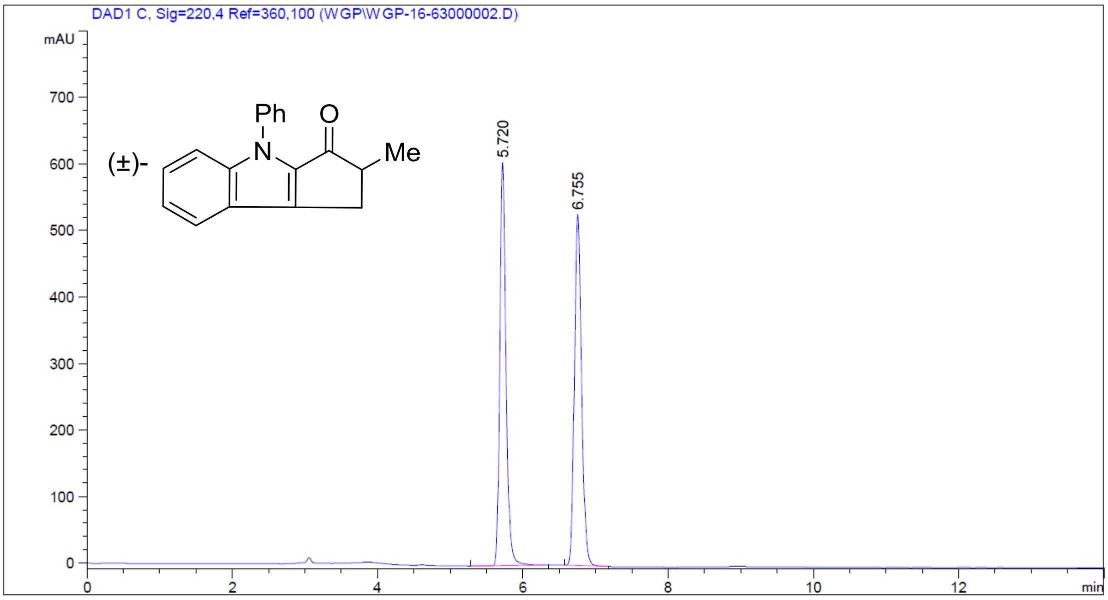

Peak #	RetTime [min]	Type	Width [min]	Area [mAU*s]	Height [mAU]	Area %
1	5.720	BB	0.0925	3667.38599	606.57990	50.0780
2	6.755	BB	0.1088	3655.96875	528.13989	49.9220

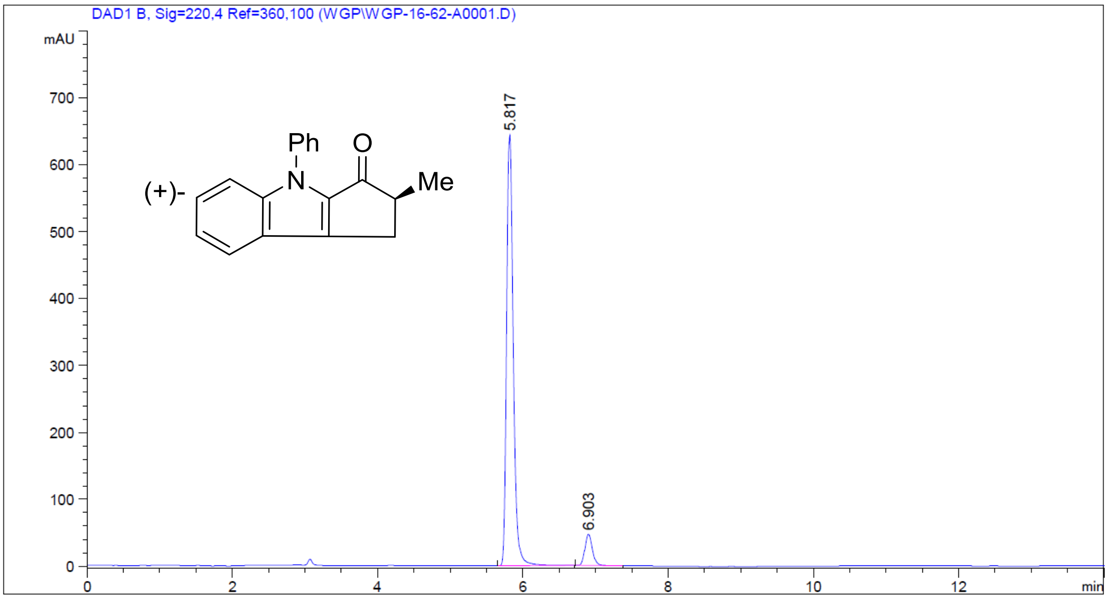

Peak #	RetTime [min]	Type	Width [min]	Area [mAU*s]	Height [mAU]	Area %
1	5.817	BB	0.0973	4046.63794	644.17590	92.2467
2	6.903	BB	0.1123	340.11893	47.06593	7.7533

(+)-4-(3-methoxyphenyl)-2-methyl-1,4-dihydrocyclopenta[b]indol-3(2H)-one (3f)

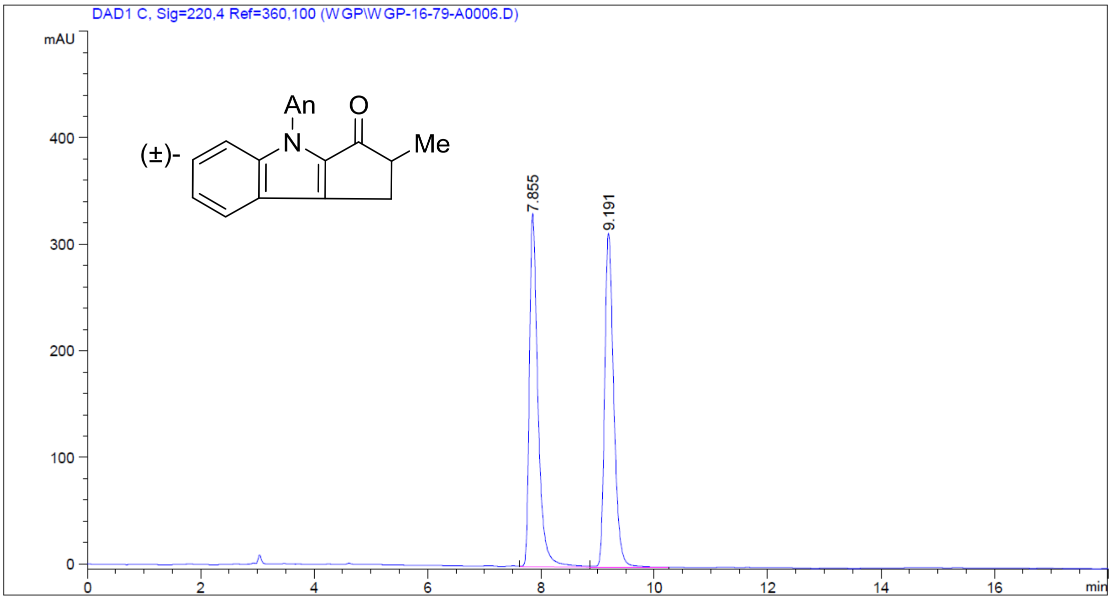

Peak #	RetTime [min]	Type	Width [min]	Area [mAU*s]	Height [mAU]	Area %
1	7.855	VV	0.1634	3478.91333	328.91351	49.9364
2	9.191	VB	0.1744	3487.77856	312.07806	50.0636

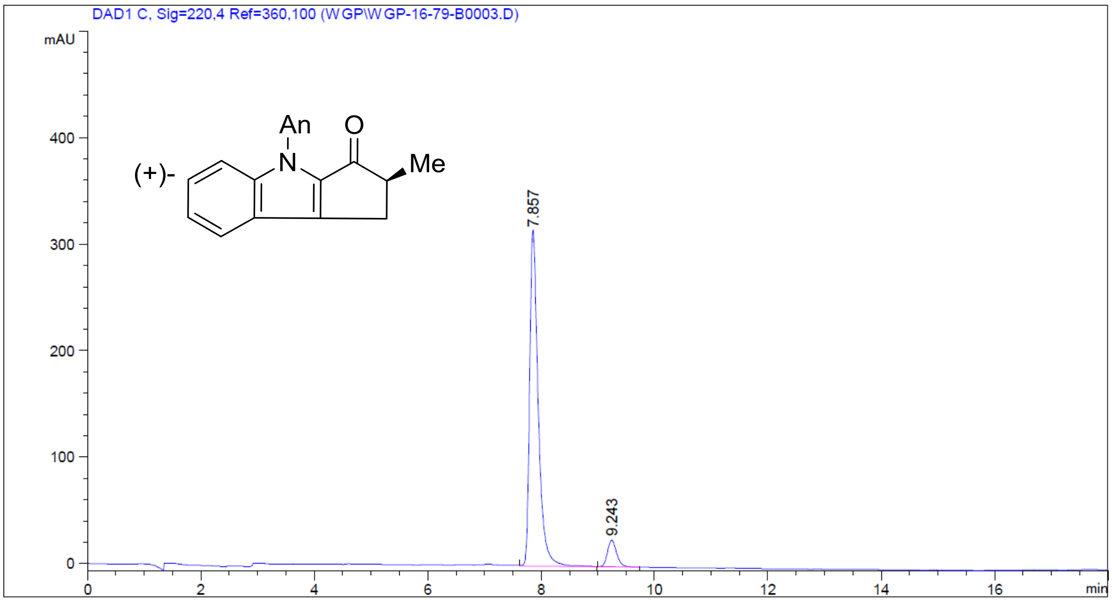

Peak #	RetTime [min]	Type	Width [min]	Area [mAU*s]	Height [mAU]	Area %
1	7.857	VB	0.1590	3310.79541	314.10974	92.1710
2	9.243	BB	0.1752	281.21967	25.01088	7.8290

(+)-2-methyl-4-(3-(trifluoromethyl)phenyl)-1,4-dihydrocyclopenta[b]indol-3(2H)-one (3g)

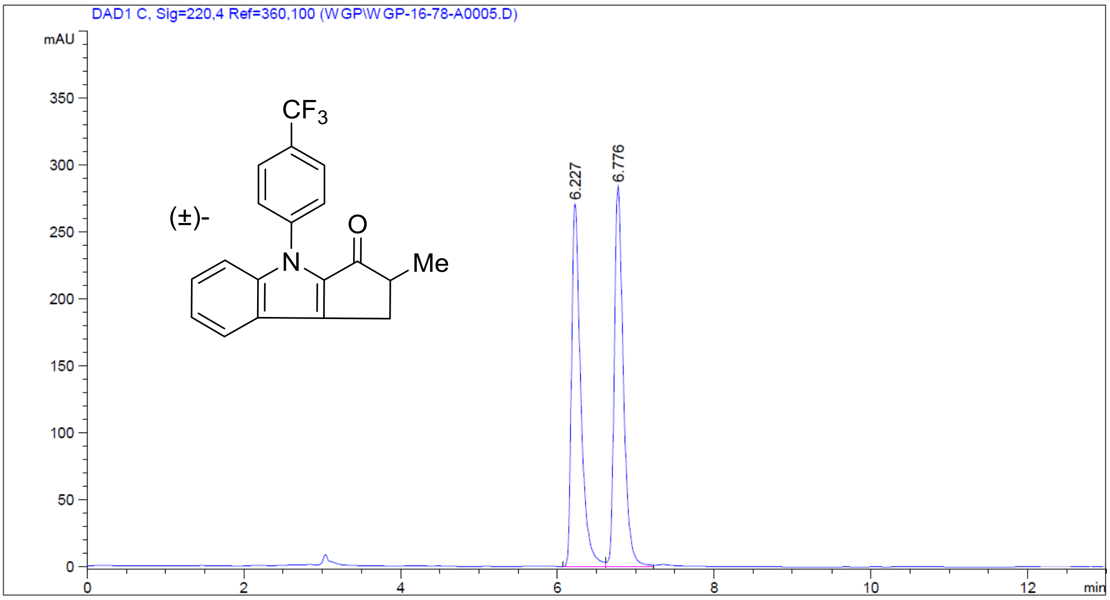

Peak #	RetTime [min]	Type	Width [min]	Area [mAU*s]	Height [mAU]	Area %
1	6.227	BV	0.1238	2222.37671	270.74310	49.1373
2	6.776	VV	0.1244	2300.40942	284.19302	50.8627

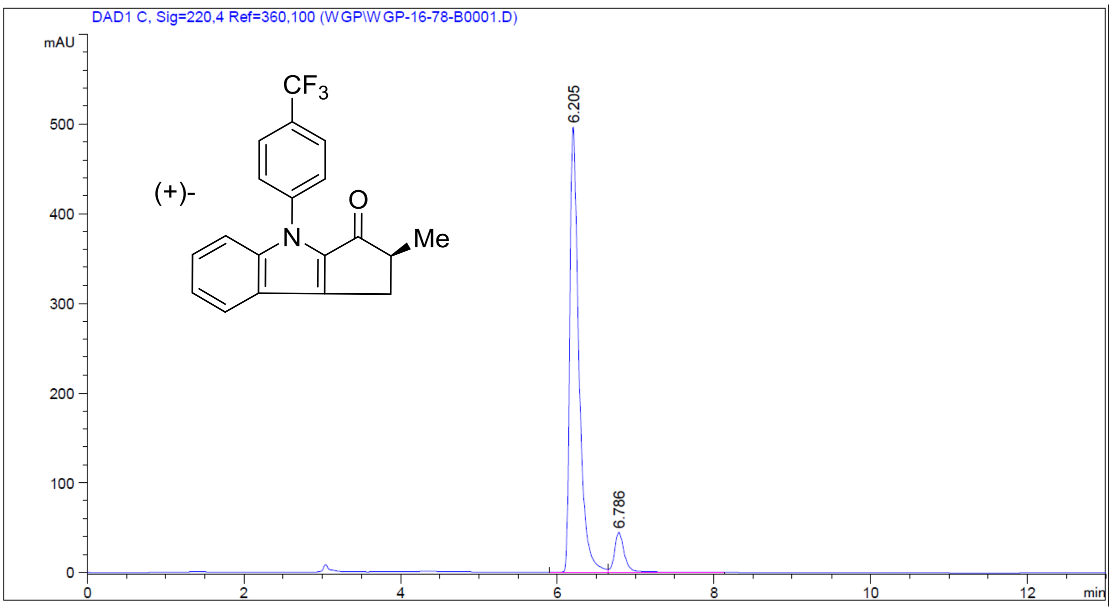

Peak #	RetTime [min]	Type	Width [min]	Area [mAU*s]	Height [mAU]	Area %
1	6.205	BV	0.1227	4026.77759	496.19296	90.9884
2	6.786	VB	0.1345	398.81454	44.53178	9.0116

(+)-2-methyl-4-(naphthalen-2-yl)-1,4-dihydrocyclopenta[b]indol-3(2H)-one (3h)

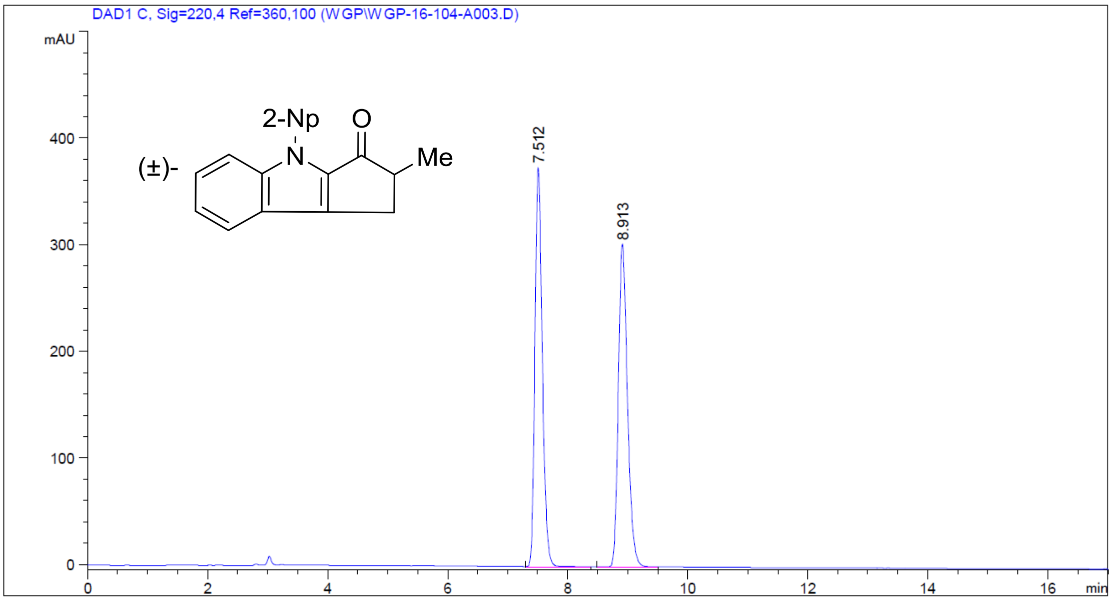

Peak #	RetTime [min]	Type	Width [min]	Area [mAU*s]	Height [mAU]	Area %
1	7.512	BB	0.1318	3197.82837	374.00400	49.9857
2	8.913	BB	0.1634	3199.65796	302.59549	50.0143

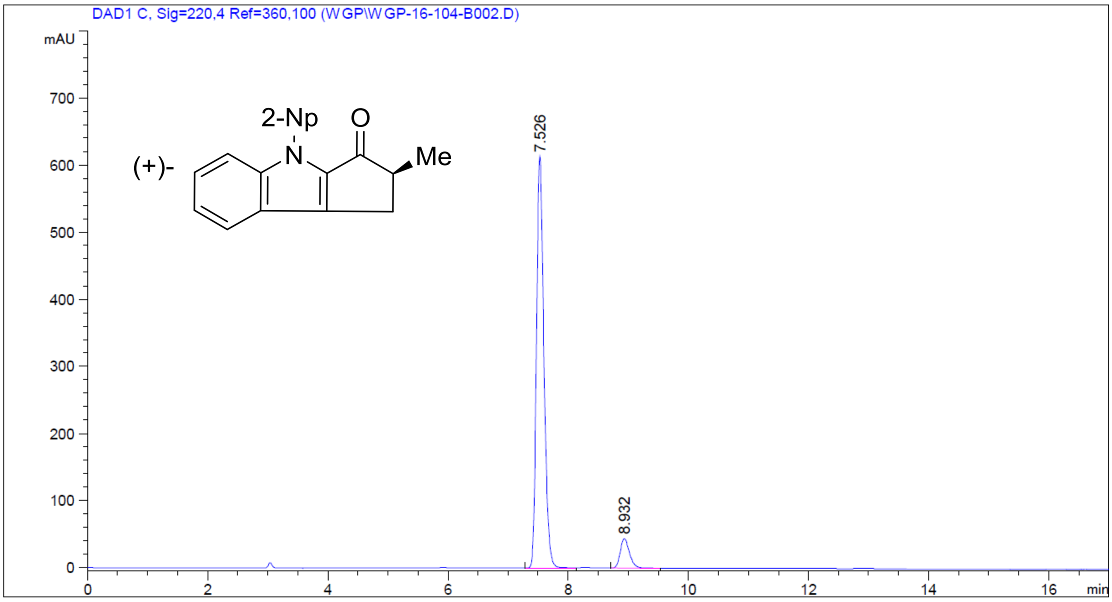

Peak #	RetTime [min]	Type	Width [min]	Area [mAU*s]	Height [mAU]	Area %
1	7.526	BB	0.1299	5245.70215	612.84015	91.7620
2	8.932	BB	0.1621	470.93466	44.27336	8.2380

(+)-2-ethyl-4-methyl-1,4-dihydrocyclopenta[b]indol-3(2H)-one (3i)

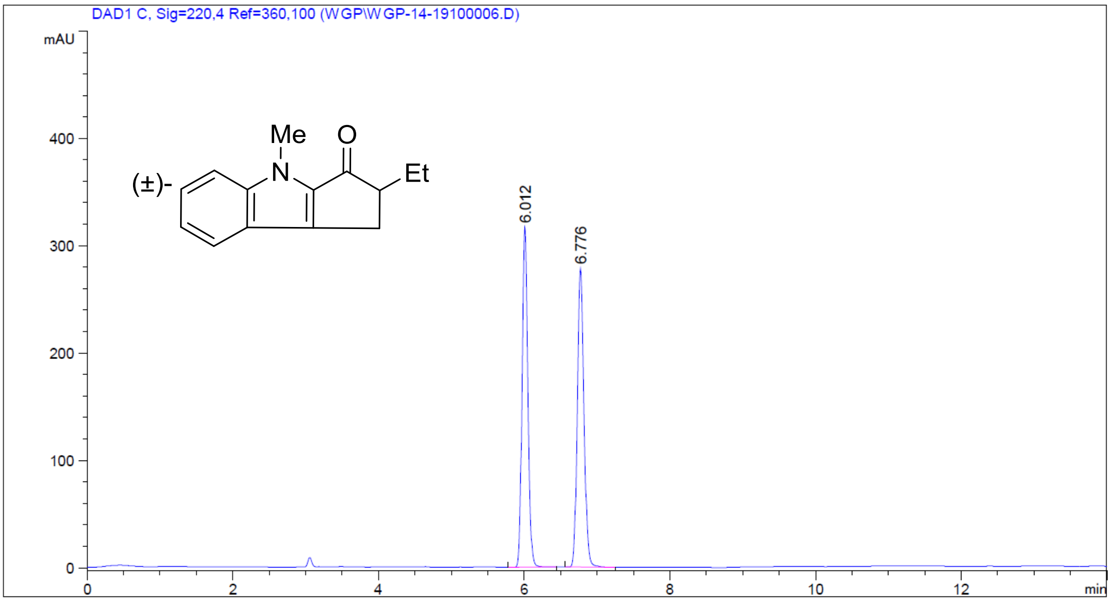

Peak #	RetTime [min]	Type	Width [min]	Area [mAU*s]	Height [mAU]	Area %
1	6.012	BB	0.0871	1771.34790	317.23035	49.8658
2	6.776	BB	0.1006	1780.88123	278.41373	50.1342

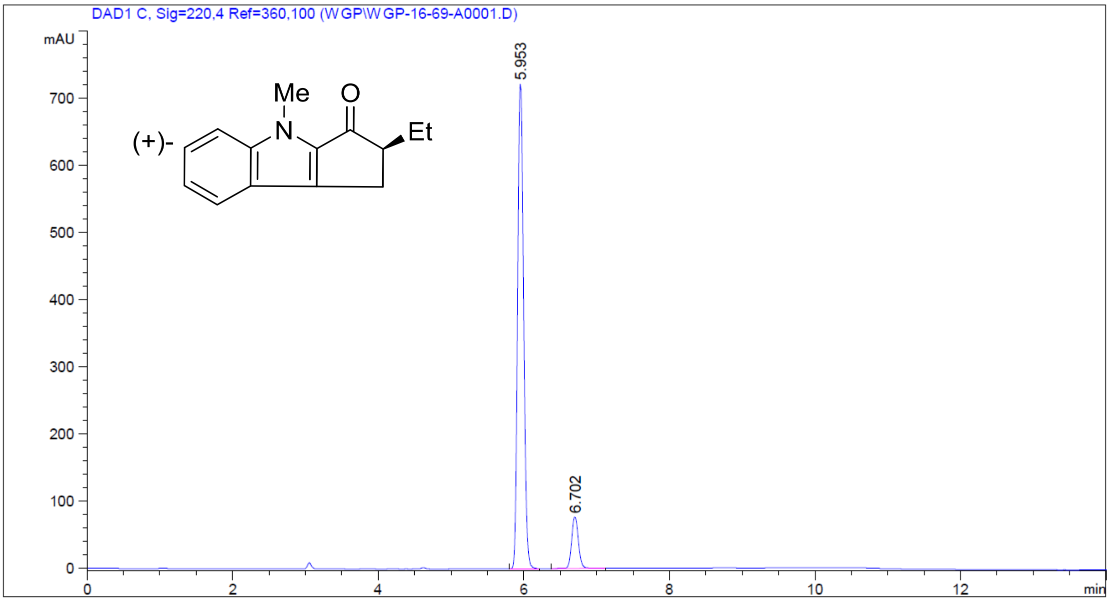

Peak #	RetTime [min]	Type	Width [min]	Area [mAU*s]	Height [mAU]	Area %
1	5.953	BB	0.0867	4006.32813	721.99646	89.1700
2	6.702	BB	0.0983	486.58258	76.43518	10.8300

(+)-2-isopropyl-4-methyl-1,4-dihydrocyclopenta[b]indol-3(2H)-one (3j)

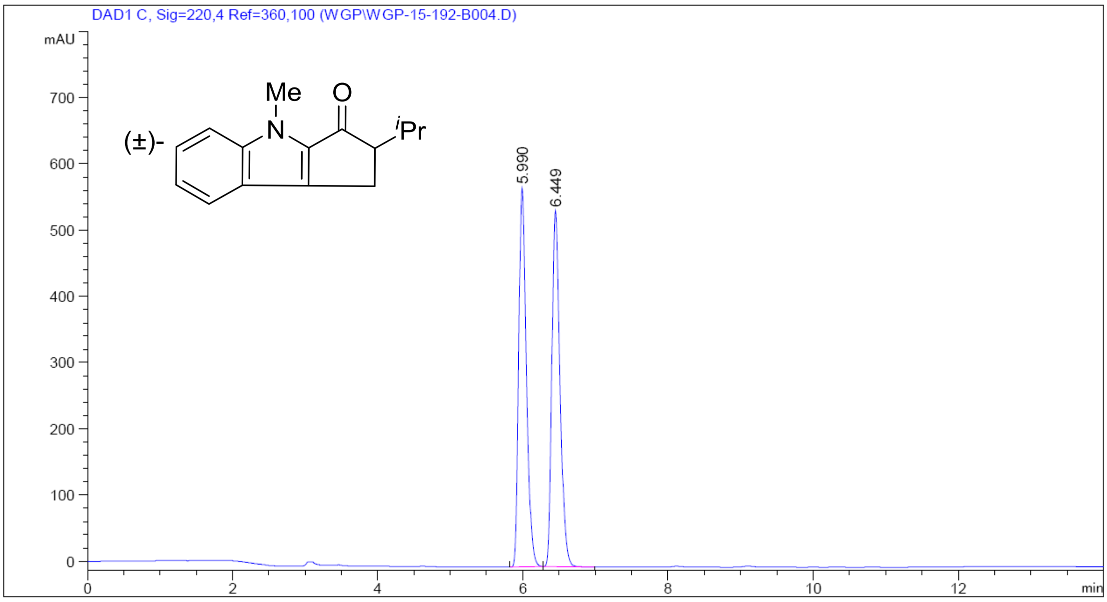

Peak #	RetTime [min]	Type	Width [min]	Area [mAU*s]	Height [mAU]	Area %
1	5.990	BB	0.1115	4185.81201	571.25684	49.9622
2	6.449	BB	0.1210	4192.15234	537.23822	50.0378

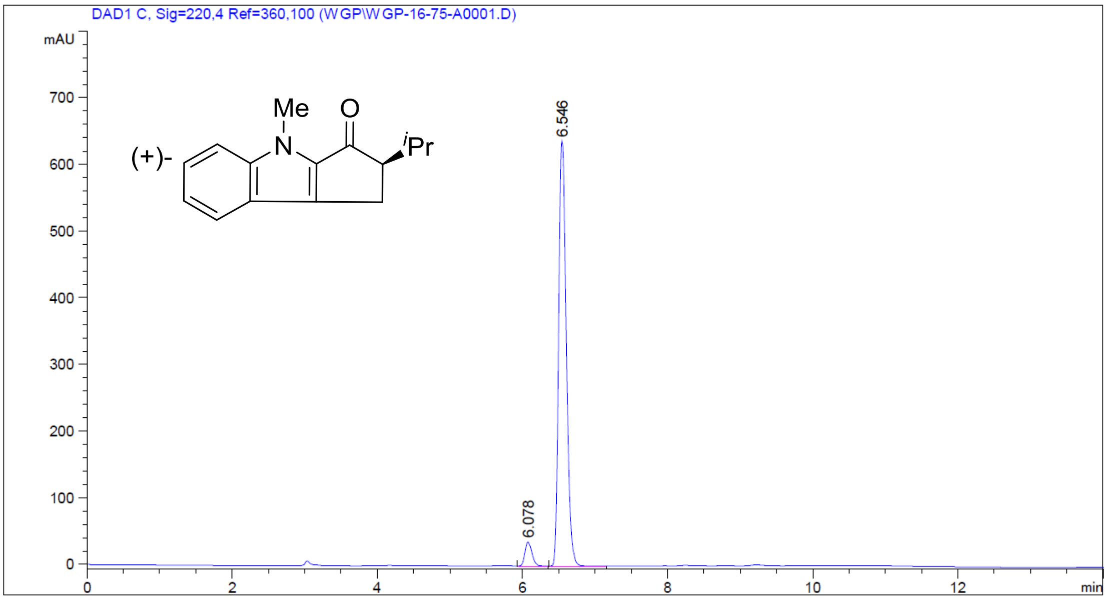

Peak #	RetTime [min]	Type	Width [min]	Area [mAU*s]	Height [mAU]	Area %
1	6.078	VB	0.1061	242.51311	36.23941	5.0169
2	6.546	BB	0.1121	4591.42285	636.79755	94.9831

(+)-4-methyl-2-phenyl-1,4-dihydrocyclopenta[b]indol-3(2H)-one (3k)

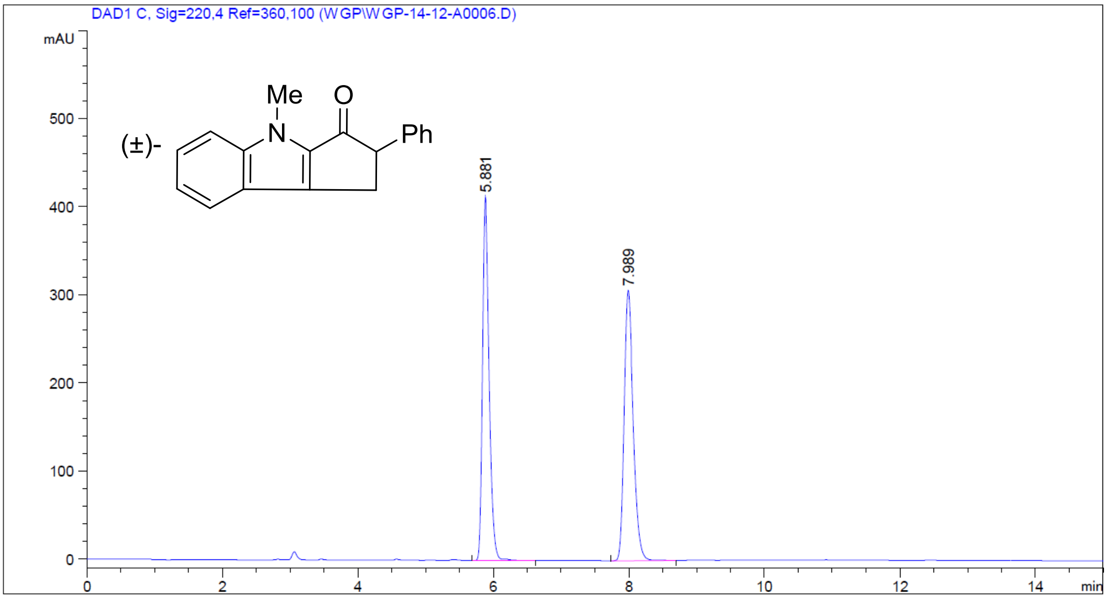

Peak #	RetTime [min]	Type	Width [min]	Area [mAU*s]	Height [mAU]	Area %
1	5.881	BB	0.1069	2860.16919	412.65671	49.9539
2	7.989	BB	0.1428	2865.44922	307.12250	50.0461

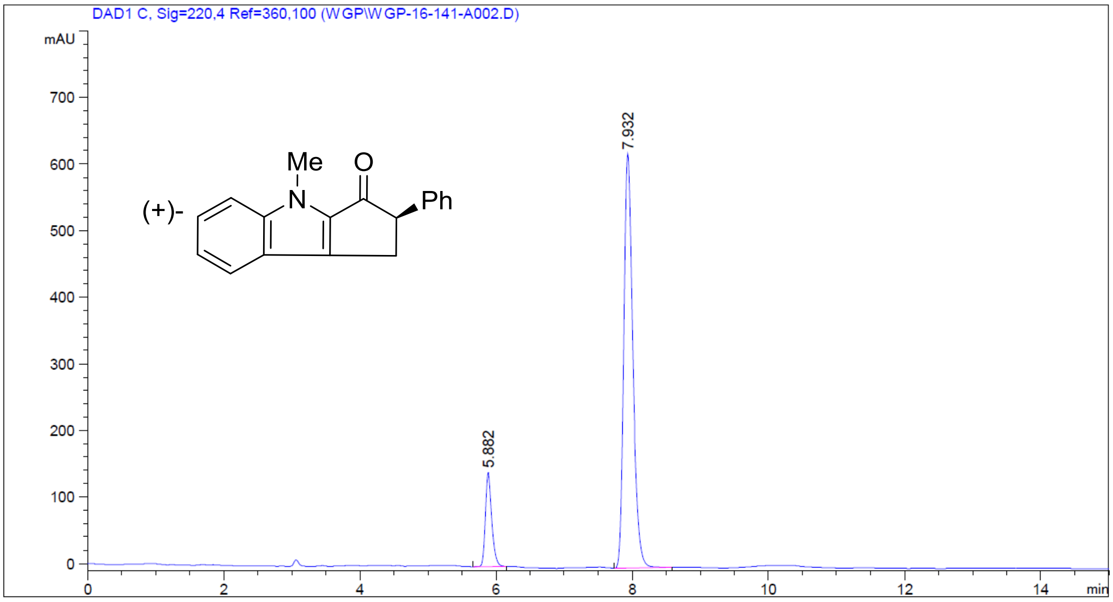

Peak #	RetTime [min]	Type	Width [min]	Area [mAU*s]	Height [mAU]	Area %
1	5.882	BB	0.1012	938.11768	141.71437	14.1556
2	7.932	BB	0.1407	5689.06592	621.92834	85.8444

(+)-2-(3-methoxyphenyl)-4-methyl-1,4-dihydrocyclopenta[b]indol-3(2H)-one(3l)

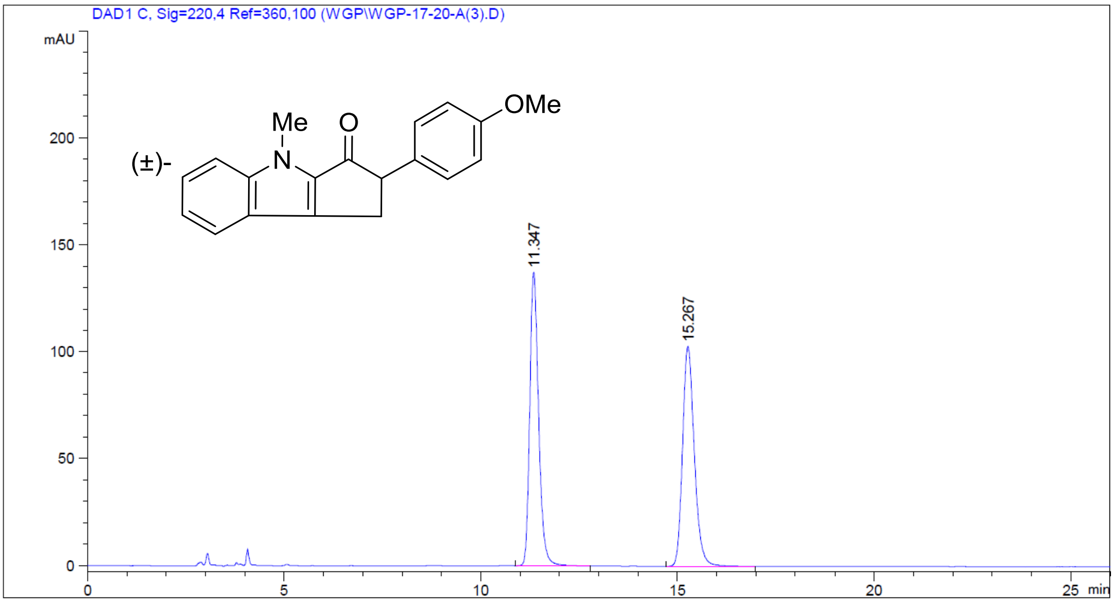

Peak #	RetTime [min]	Type	Width [min]	Area [mAU*s]	Height [mAU]	Area %
1	11.347	BB	0.2389	2151.22803	137.24571	50.1445
2	15.267	BB	0.3186	2138.83081	102.84928	49.8555

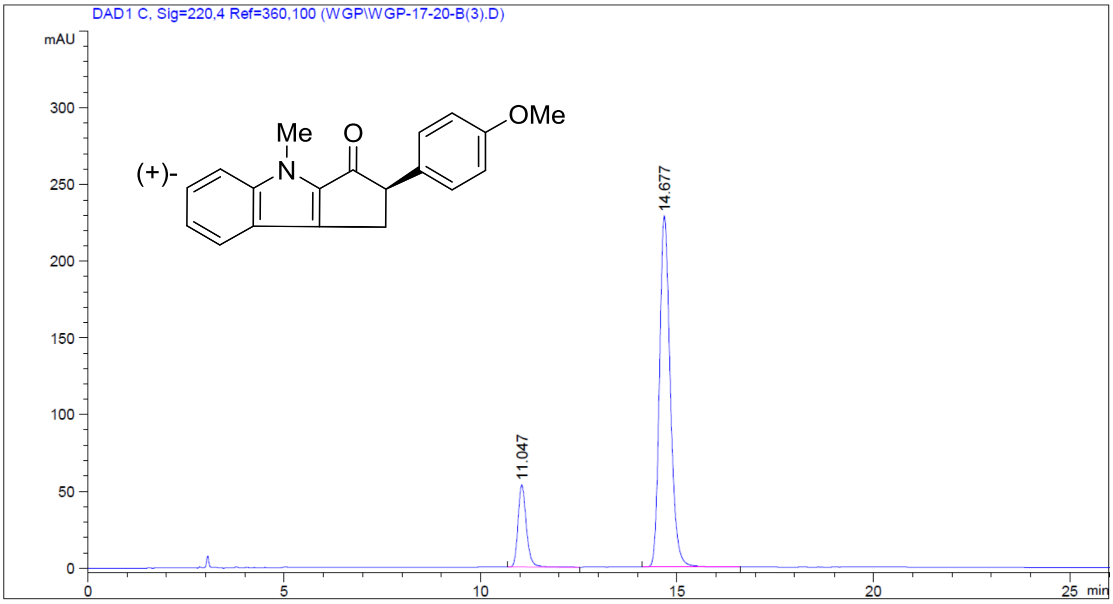

Peak #	RetTime [min]	Type	Width [min]	Area [mAU*s]	Height [mAU]	Area %
1	11.047	BB	0.2236	789.98444	53.69997	15.3006
2	14.677	BB	0.2929	4373.09863	228.85213	84.6994

(+)-2-(3-fluorophenyl)-4-methyl-1,4-dihydrocyclopenta[b]indol-3(2H)-one (3m)

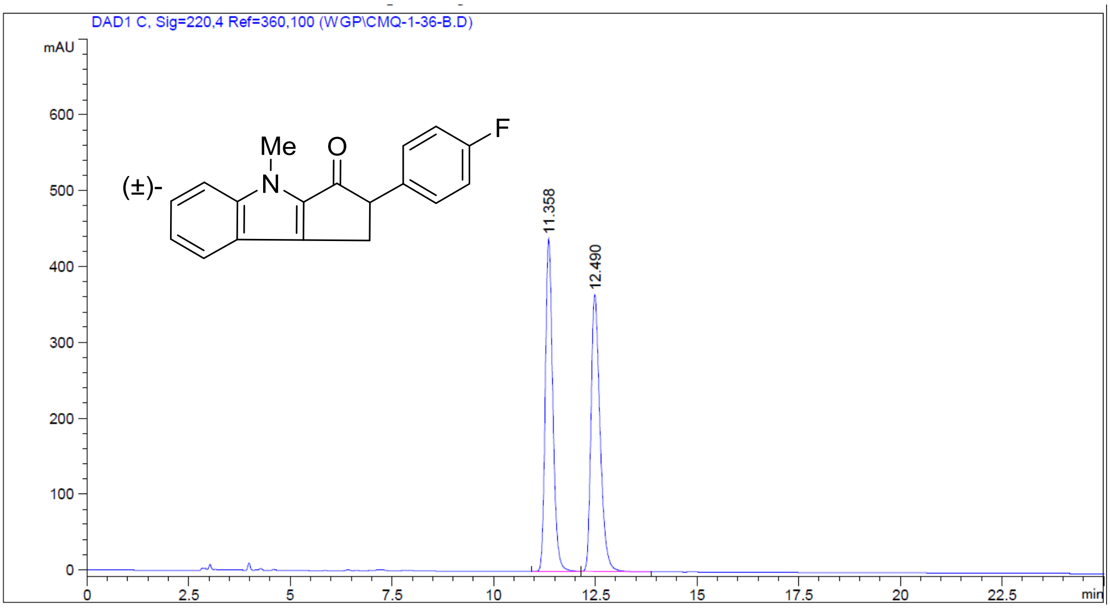

Peak #	RetTime [min]	Type	Width [min]	Area [mAU*s]	Height [mAU]	Area %
1	11.358	BB	0.1983	5653.51416	438.91394	50.0182
2	12.490	BB	0.2365	5649.40723	365.24557	49.9818

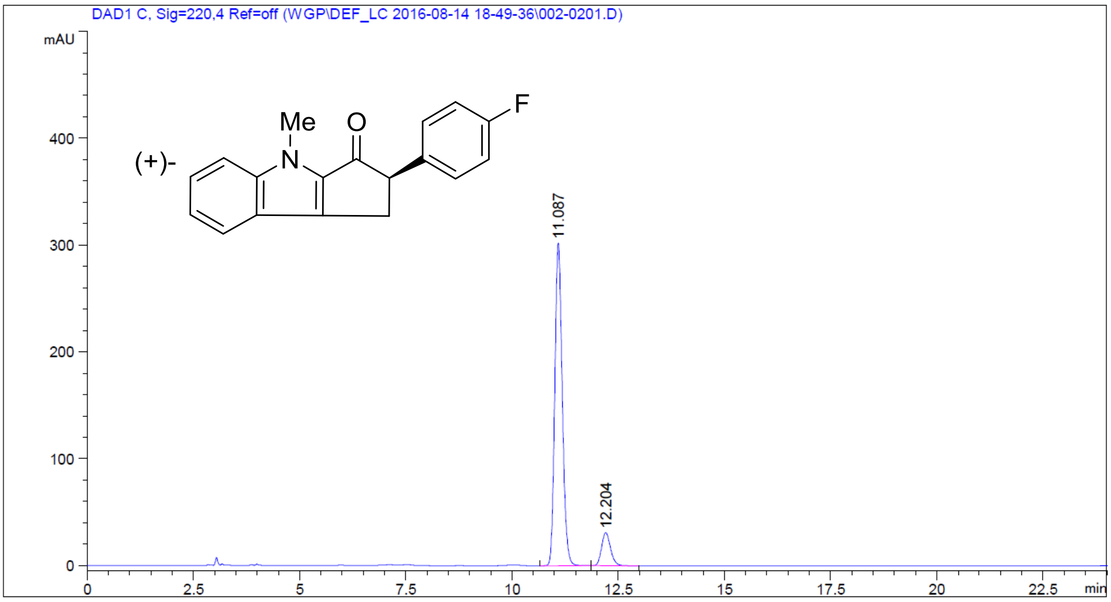

Peak #	RetTime [min]	Type	Width [min]	Area [mAU*s]	Height [mAU]	Area %
1	11.087	BB	0.1890	3699.49390	301.81158	88.9780
2	12.204	BB	0.2261	458.26645	31.07299	11.0220

(+)-2-(3-chlorophenyl)-4-methyl-1,4-dihydrocyclopenta[b]indol-3(2H)-one (3n)

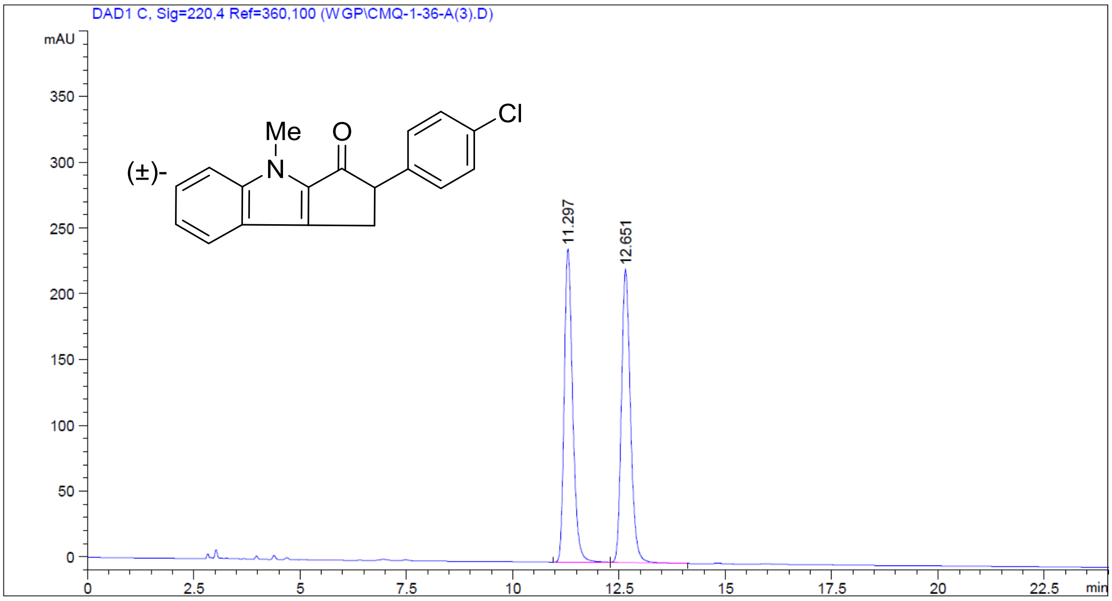

Peak #	RetTime [min]	Type	Width [min]	Area [mAU*s]	Height [mAU]	Area %
1	11.297	BB	0.2112	3289.29663	238.03419	50.0363
2	12.651	BB	0.2261	3284.52832	222.70454	49.9637

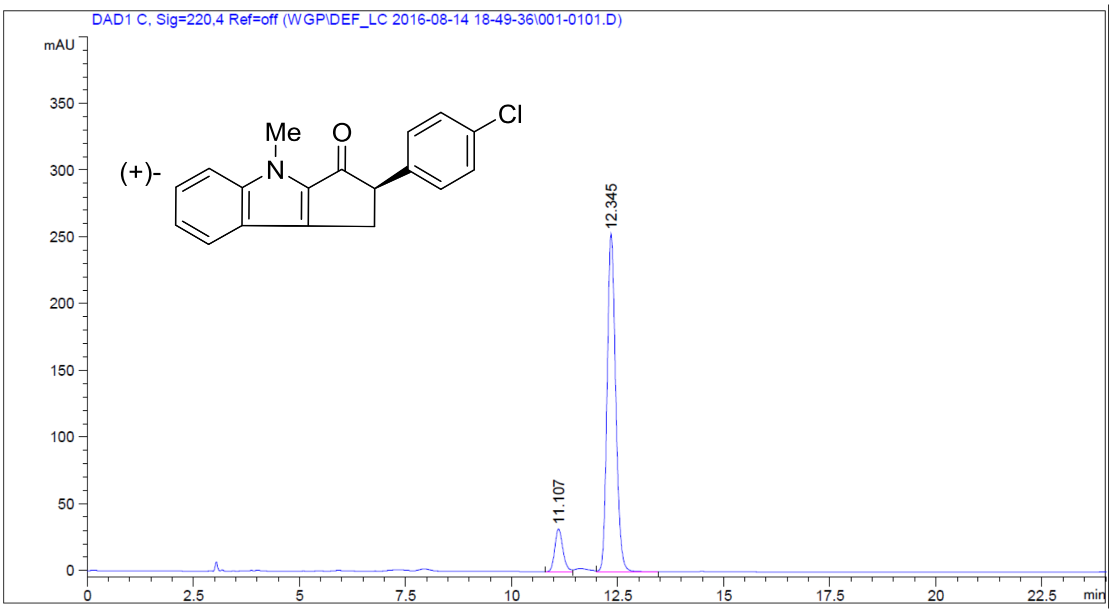

Peak #	RetTime [min]	Type	Width [min]	Area [mAU*s]	Height [mAU]	Area %
1	11.107	BV	0.2056	427.84143	32.07363	10.7141
2	12.345	VB	0.2182	3565.42065	253.40332	89.2859

(+)-2-(benzo[d][1,3]dioxol-5-yl)-4-methyl-1,4-dihydrocyclopenta[b]indol-3(2H)-one (3o)

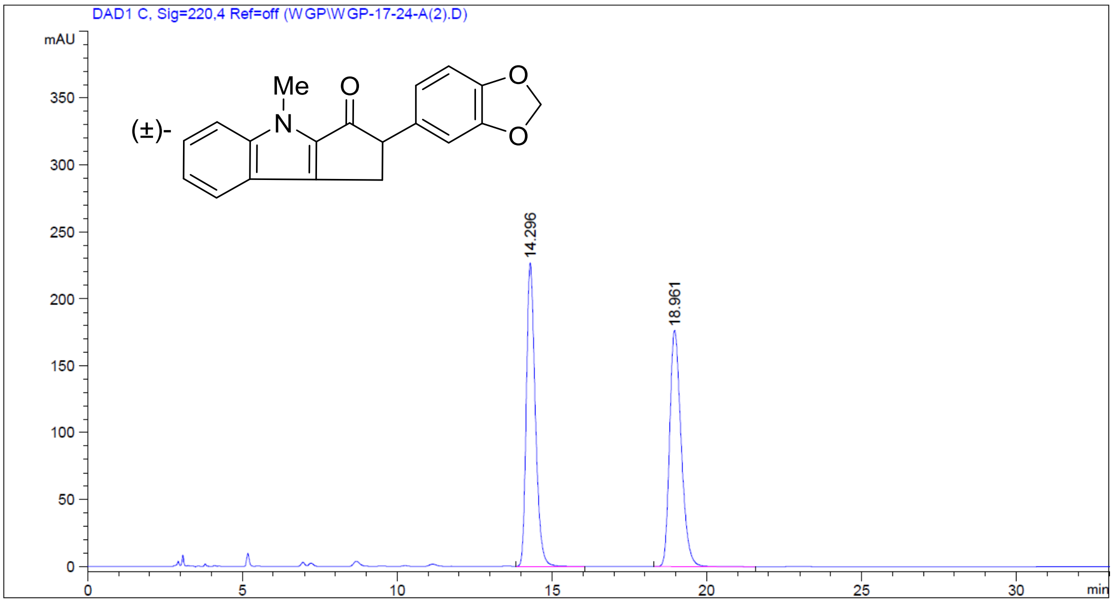

Peak #	RetTime [min]	Type	Width [min]	Area [mAU*s]	Height [mAU]	Area %
1	14.296	BB	0.3086	4557.24170	226.62152	49.8959
2	18.961	BB	0.3983	4576.25244	176.58572	50.1041

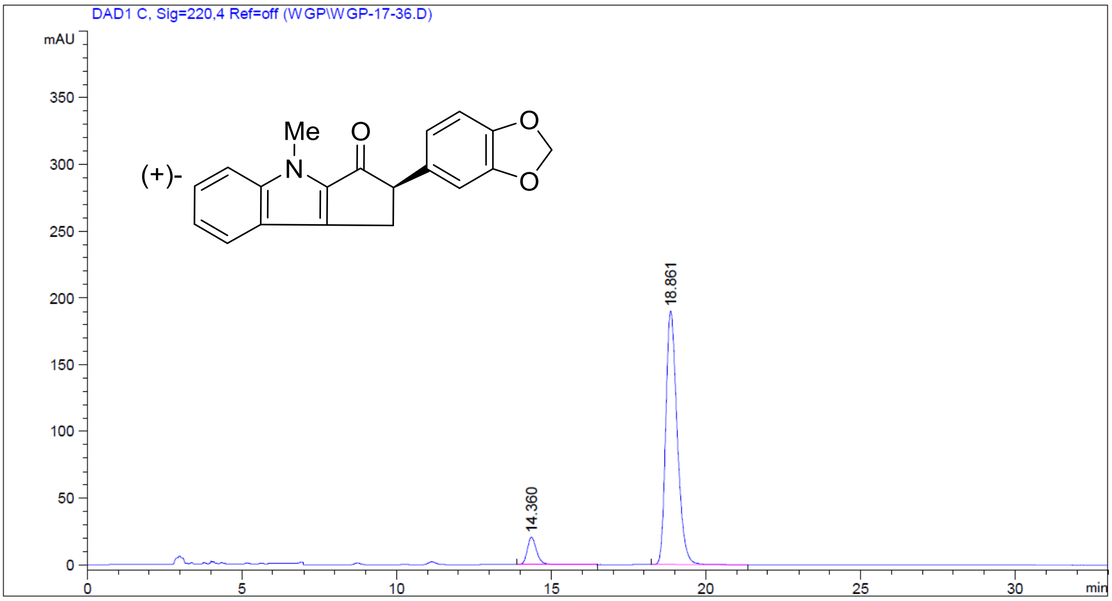

Peak #	RetTime [min]	Type	Width [min]	Area [mAU*s]	Height [mAU]	Area %
1	14.360	BB	0.3181	430.48038	20.57157	8.1107
2	18.861	BB	0.3953	4877.05273	190.11383	91.8893

(+)-2-isopropyl-4,7-dimethyl-1,4-dihydrocyclopenta[b]indol-3(2H)-one (3p)

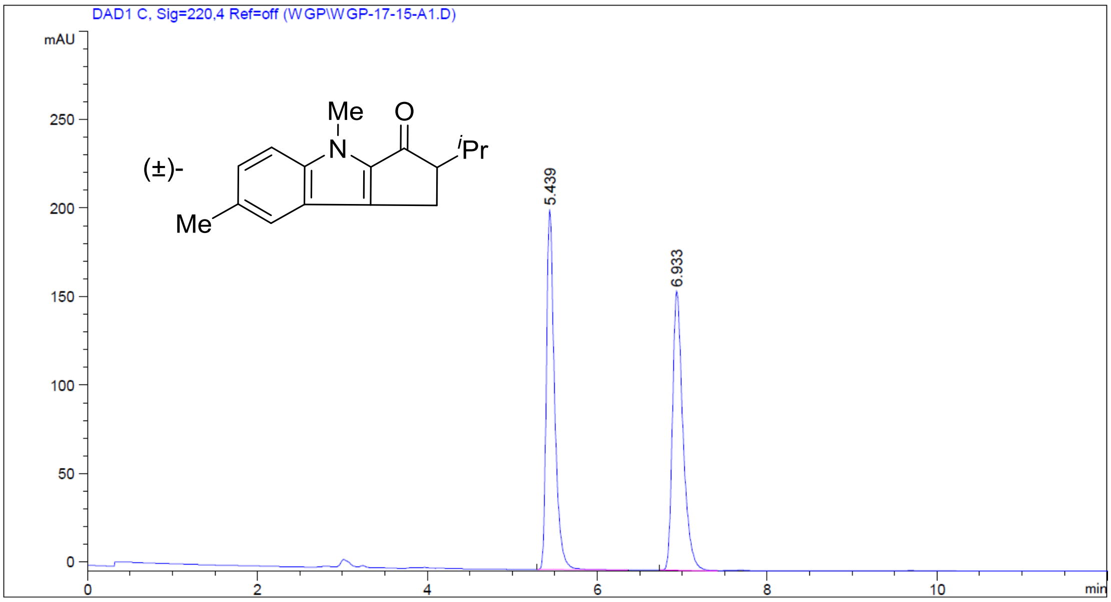

Peak #	RetTime [min]	Type	Width [min]	Area [mAU*s]	Height [mAU]	Area %
1	5.439	BB	0.0998	1358.27197	203.56589	50.0870
2	6.933	BB	0.1301	1353.55603	157.78421	49.9130

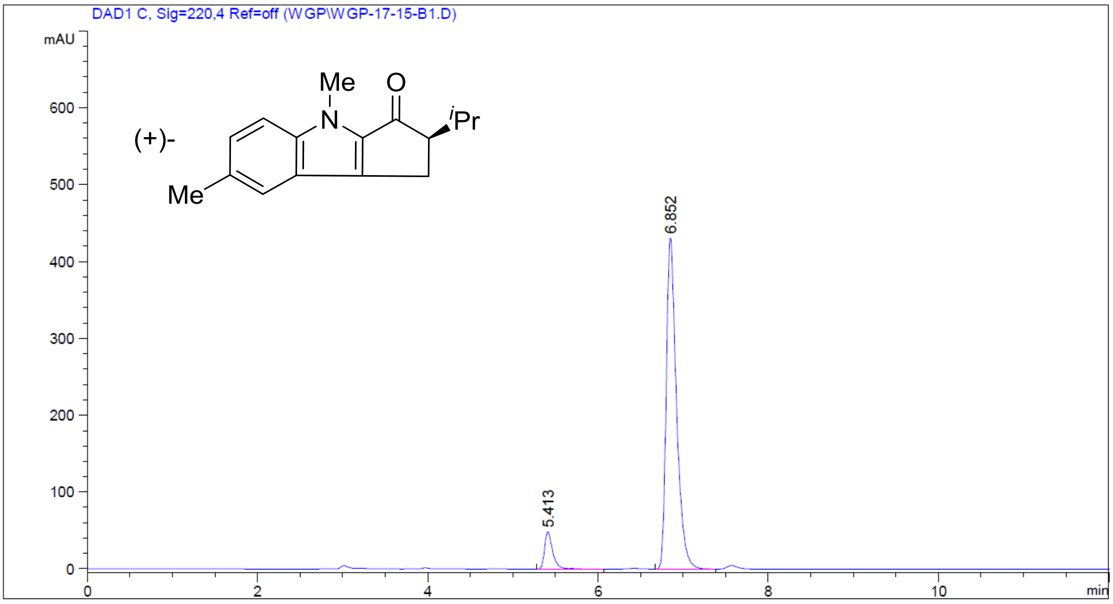

Peak #	RetTime [min]	Type	Width [min]	Area [mAU*s]	Height [mAU]	Area %
1	5.413	BB	0.0989	321.01346	48.69363	8.2061
2	6.852	BB	0.1251	3590.88110	431.42349	91.7939

(+)-7-fluoro-2-isopropyl-4-methyl-1,4-dihydrocyclopenta[b]indol-3(2H)-one (3q)

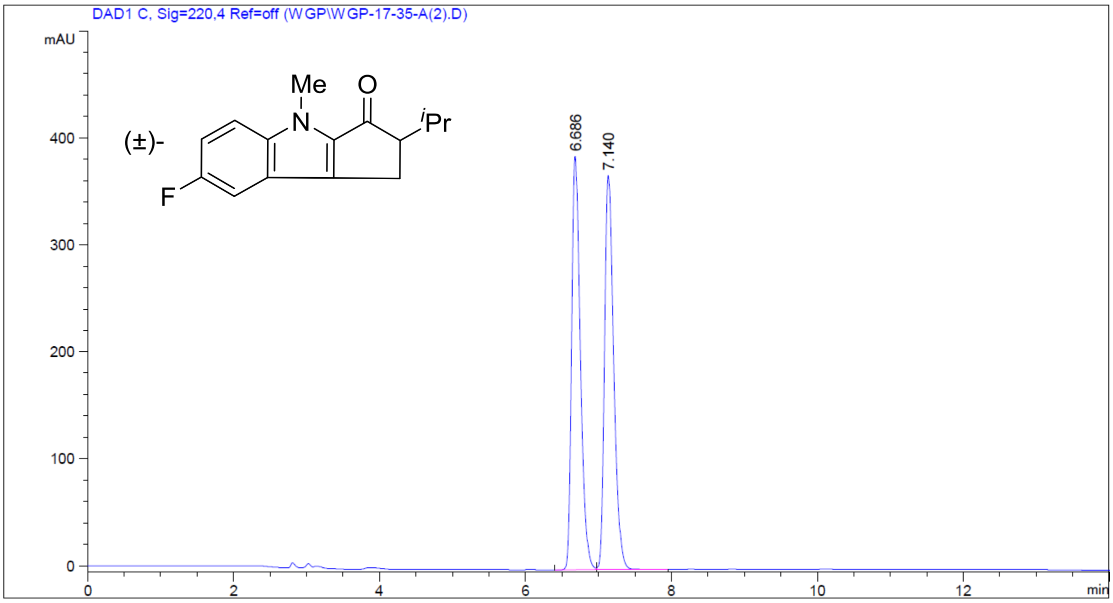

Peak #	RetTime [min]	Type	Width [min]	Area [mAU*s]	Height [mAU]	Area %
1	6.686	BV	0.1246	3208.80200	387.34805	49.8647
2	7.140	VB	0.1339	3226.21753	369.28119	50.1353

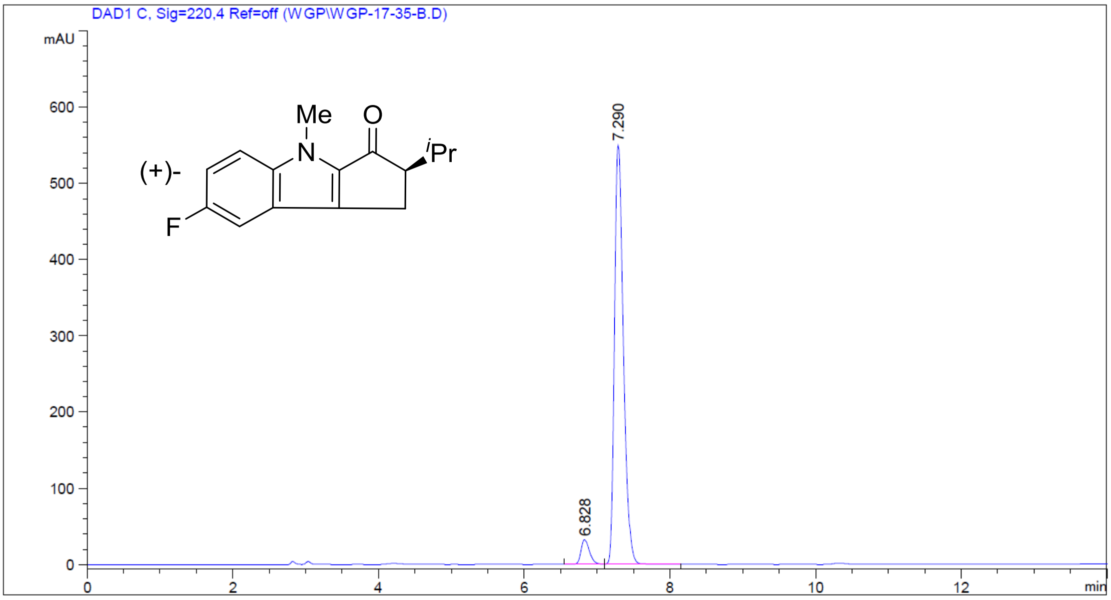

Peak #	RetTime [min]	Type	Width [min]	Area [mAU*s]	Height [mAU]	Area %
1	6.828	BV	0.1224	264.10773	32.64989	5.3645
2	7.290	VB	0.1289	4659.09668	549.42303	94.6355

(*R*)-(+)-7-chloro-2-isopropyl-4-methyl-1,4-dihydrocyclopenta[b]indol-3(2H)-one (3r)

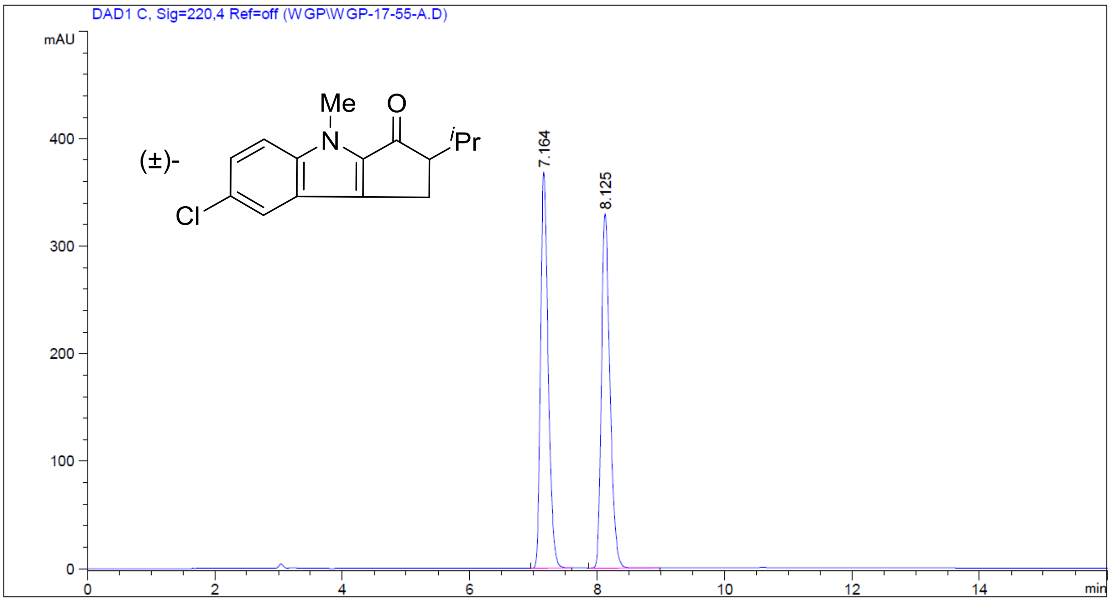

Peak #	RetTime [min]	Type	Width [min]	Area [mAU*s]	Height [mAU]	Area %
1	7.164	BB	0.1246	3054.42822	368.89114	49.8458
2	8.125	BB	0.1407	3073.32520	329.81360	50.1542

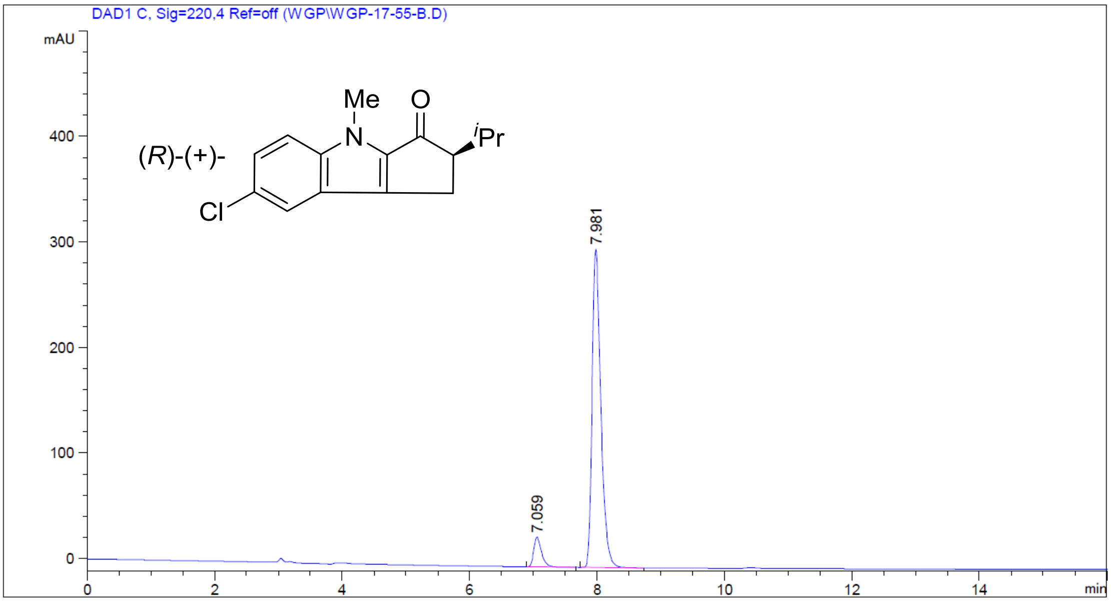

Peak #	RetTime [min]	Type	Width [min]	Area [mAU*s]	Height [mAU]	Area %
1	7.059	BB	0.1267	239.48936	28.29431	7.7251
2	7.981	BB	0.1447	2860.64624	301.52655	92.2749

(+)-8-fluoro-2-isopropyl-4-methyl-1,4-dihydrocyclopenta[b]indol-3(2H)-one (3s)

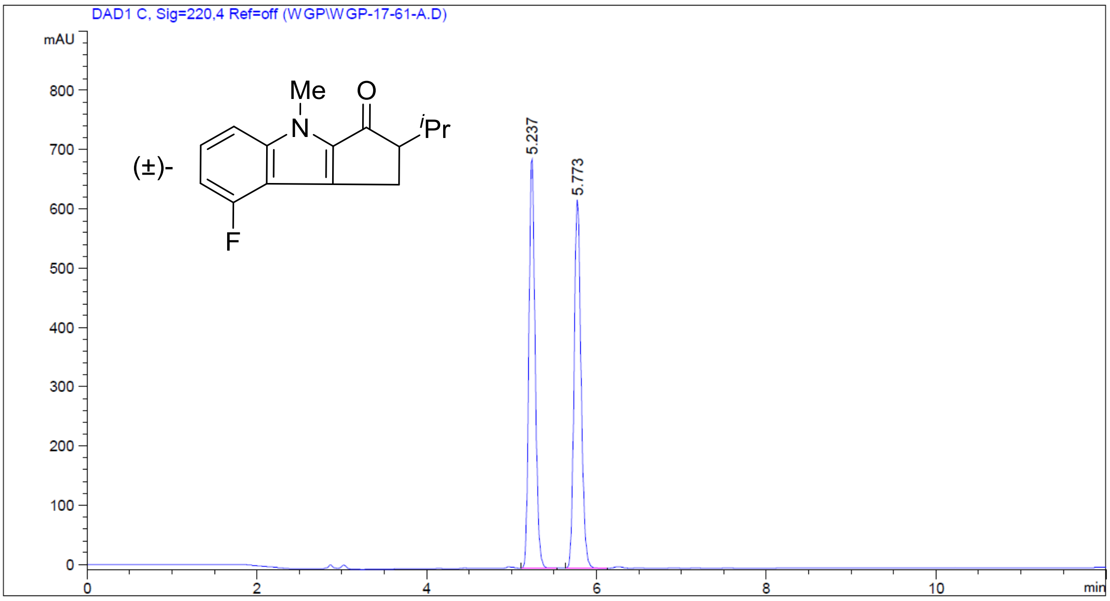

Peak #	RetTime [min]	Type	Width [min]	Area [mAU*s]	Height [mAU]	Area %
1	5.237	VV	0.0799	3547.43384	691.16223	50.1052
2	5.773	VB	0.0883	3532.53467	622.04254	49.8948

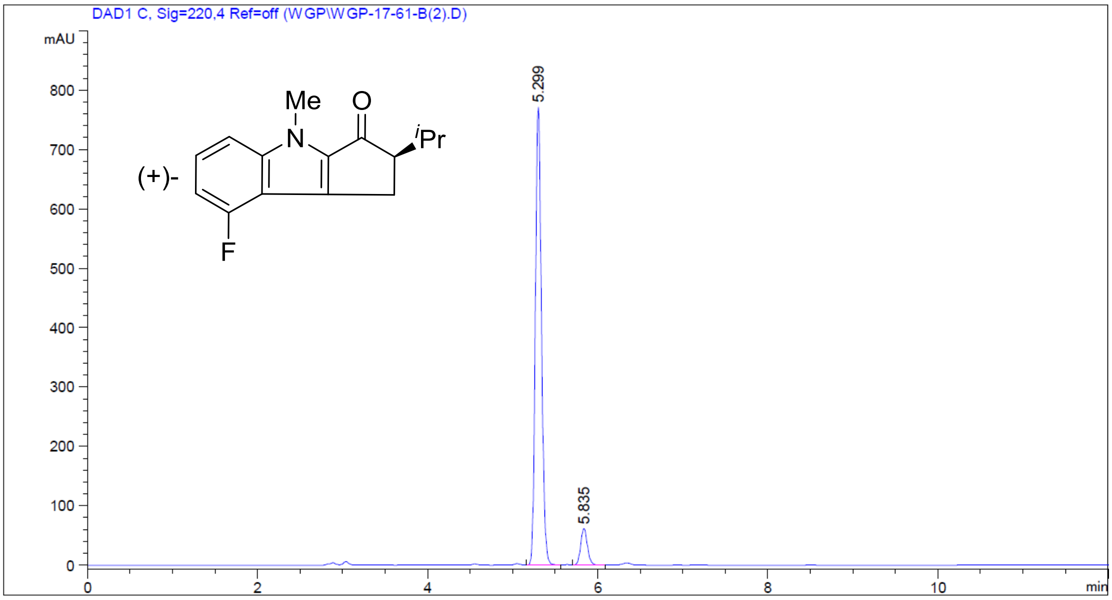

Peak #	RetTime [min]	Type	Width [min]	Area [mAU*s]	Height [mAU]	Area %
1	5.299	BV	0.0760	3831.00122	771.13232	91.8609
2	5.835	VB	0.0842	339.43655	61.64135	8.1391

(-)4-(4-methoxyphenyl)-2-methyl-1,2,3,4-tetrahydrocyclopenta[b] indol-3-ol (4)

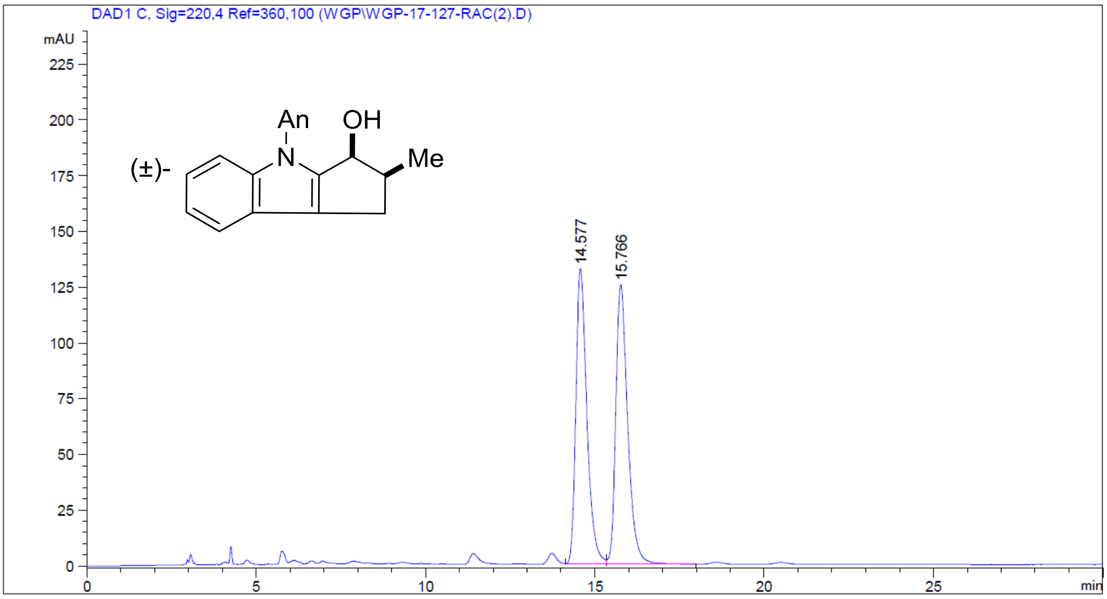

Peak #	RetTime [min]	Type	Width [min]	Area [mAU*s]	Height [mAU]	Area %
1	14.577	VV	0.3438	2995.34644	132.46559	49.4498
2	15.766	VB	0.3709	3062.00269	125.37962	50.5502

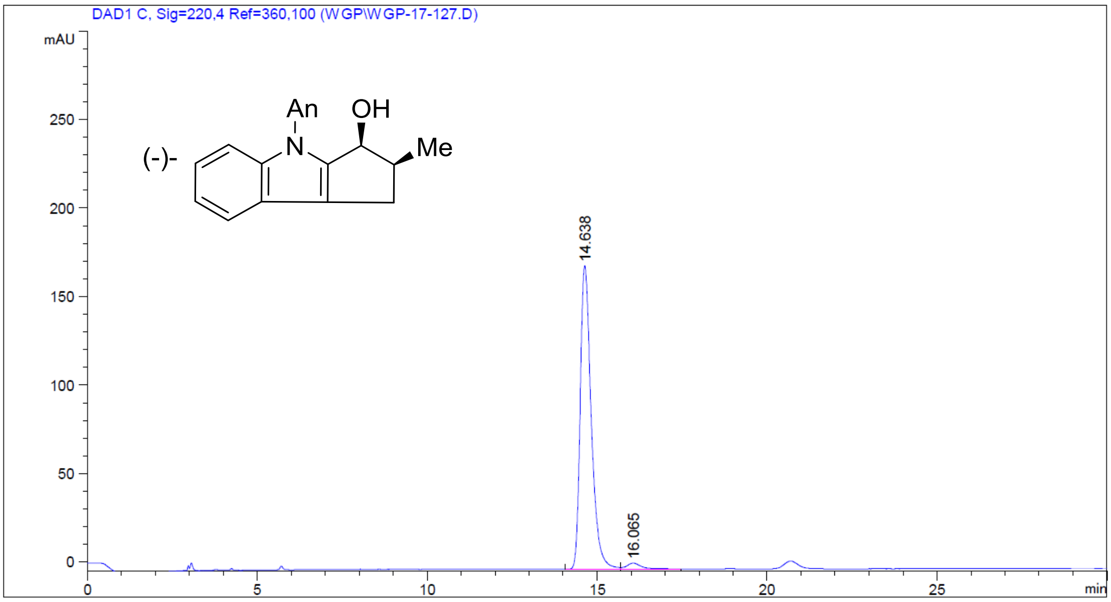

Peak #	RetTime [min]	Type	Width [min]	Area [mAU*s]	Height [mAU]	Area %
1	14.638	BV	0.3421	3851.26782	171.45178	97.5308
2	16.065	VB	0.4261	97.50146	3.32474	2.4692

10. References

(1) W. L. F. Armarego and C. L. L. Chai, *Purification of Laboratory Chemicals-Six Edition*; Elsevier Inc., London, 2009.

(2) C.-W. Zheng, Y.-P. Lu, L.-K. Zhang, X.-K. Chen, Z. Chai, W.-Y. Ma and G. Zhao, *Chem. Eur. J.*, 2010, **16**, 5853.

(3) D. Song, A. Rostami and F. G. West, *J. Am. Chem. Soc.*, 2007, **129**, 12019.

(4) Y. Wang, B. D. Schill, A. M. Arif and F. G. West, *Org. Lett.*, 2003, **5**, 2747.

(5) H. J. Johnston, M. S. McWhinnie, F. Landi and A. N. Hulme, *Org. Lett.*, 2014, **16**, 4778.

(6) S. Qi, C.-Y. Liu, J.-Y. Ding and F.-S. Han, *Chem. Commun.*, 2014, **50**, 8605.

(7) J. A. Malona, J. Colbourne and A. J. Frontier, *Org. Lett.*, 2006, **8**, 5661.

(8) J. T. S. Yeoman, V. W. Mak and S. E. Reisman, *J. Am. Chem. Soc.*, 2013, **135**, 11764.
