# Supplementary material for: Enantioselective Nazarov cyclization of indole enones cooperatively catalyzed by Lewis acids and chiral Brønsted acids
Source: Chem Sci. 2017 Aug 29;8(10):7197–202. doi: 10.1039/c7sc03183a (PMC5633839; doi:10.1039/c7sc03183a)
Supplement: Supplementary file 2 [file SC-008-C7SC03183A-s002.pdf]

Electronic Supplementary Material (ESI) for Chemical Science.
This journal is © The Royal Society of Chemistry 2017

Supplementary Information for:

Enantioselective Nazarov cyclization of indole enones cooperatively catalyzed by Lewis acids and chiral Brønsted acids

Guo-Peng Wang,[a] Meng-Qing Chen,[a] Shou-Fei Zhu,*,[a] and Qi-Lin Zhou*,[a,b]

[a] *State Key Laboratory and Institute of Elemento-Organic Chemistry, College of Chemistry, Nankai University, Tianjin 300071, China*

[b] *Collaborative Innovation Center of Chemical Science and Engineering (Tianjin), Nankai University, Tianjin 300071, China*

Computational Methods

DFT Methods

All DFT theoretical calculations have been carried out using the *Gaussian 09* program package. The B3LYP[1] method with DEF2SVP[2] basis set has been selected for geometry optimizations and calculation of Gibbs energy corrections at 298 K temperature. Final energies were retrieved from single-point calculations at the B3LYP/DEF2TZVP[2] level, including the D3BJ dispersion correction scheme developed by Grimme[3]. All structures have been optimized considering solvent effects using the SMD[4] model for 1,2-dichloroethane. Reaction paths were traced by the intrinsic reaction coordinate method for all transition states. All energetics reported throughout the text are in kcal/mol. Structures were generated using CYLview[5].

Complete Citation for Gaussian 09

Gaussian 09, Revision D.01,
M. J. Frisch, G. W. Trucks, H. B. Schlegel, G. E. Scuseria, M. A. Robb, J. R. Cheeseman, G. Scalmani, V. Barone, B. Mennucci, G. A. Petersson, H. Nakatsuji, M. Caricato, X. Li, H. P. Hratchian, A. F. Izmaylov, J. Bloino, G. Zheng, J. L. Sonnenberg, M. Hada, M. Ehara, K. Toyota, R. Fukuda, J. Hasegawa, M. Ishida, T. Nakajima, Y. Honda, O. Kitao, H. Nakai, T. Vreven, J. A. Montgomery, Jr., J. E. Peralta, F. Ogliaro, M. Bearpark, J. J. Heyd, E. Brothers, K. N. Kudin, V. N.

Staroverov, T. Keith, R. Kobayashi, J. Normand, K. Raghavachari, A. Rendell, J. C. Burant, S. S. Iyengar, J. Tomasi, M. Cossi, N. Rega, J. M. Millam, M. Klene, J. E. Knox, J. B. Cross, V. Bakken, C. Adamo, J. Jaramillo, R. Gomperts, R. E. Stratmann, O. Yazyev, A. J. Austin, R. Cammi, C. Pomelli, J. W. Ochterski, R. L. Martin, K. Morokuma, V. G. Zakrzewski, G. A. Voth, P. Salvador, J. J. Dannenberg, S. Dapprich, A. D. Daniels, O. Farkas, J. B. Foresman, J. V. Ortiz, J. Cioslowski and D. J. Fox, *Gaussian 09*, Revision D.01; Gaussian, Inc.: Wallingford, CT, 2013.

Table S1. Sum of computed energies of stationary points (For Figure S1 and Figure S2)

**Structure**	**ΔG_{sol}**	**ΔH_{sol}**
Substrate	-633.29770445	-633.24290745
$ZnCl_2$	-2700.08507295	-2700.05343195
(*R*)-TRIP-SPA	-2469.22291068	-2469.08284268
INT I	-3333.37398600	-3333.30254300
TS I	-5802.56558656	-5802.37975856
INT II	-5802.60318700	-5802.41996000
CP II-Pre	-3102.52107157	-3102.35154957
TS II	-3102.49799870	-3102.33011570
CP II-Post	-3102.51322113	-3102.34268313
Enol	-633.29051703	-633.23724603
CP III-R-Pre	-3102.51322752	-3102.34109552
CP III-S-Pre	-3102.51387608	-3102.34194508
TS III-R	-3102.50432525	-3102.33562925
TS III-S	-3102.50742828	-3102.33908928
CP III-R-Post	-3102.54344755	-3102.37144155
CP III-S-Post	-3102.54550249	-3102.37255949
Product-R	-633.31653363	-633.26406363
Product-S	-633.31653194	-633.26406394

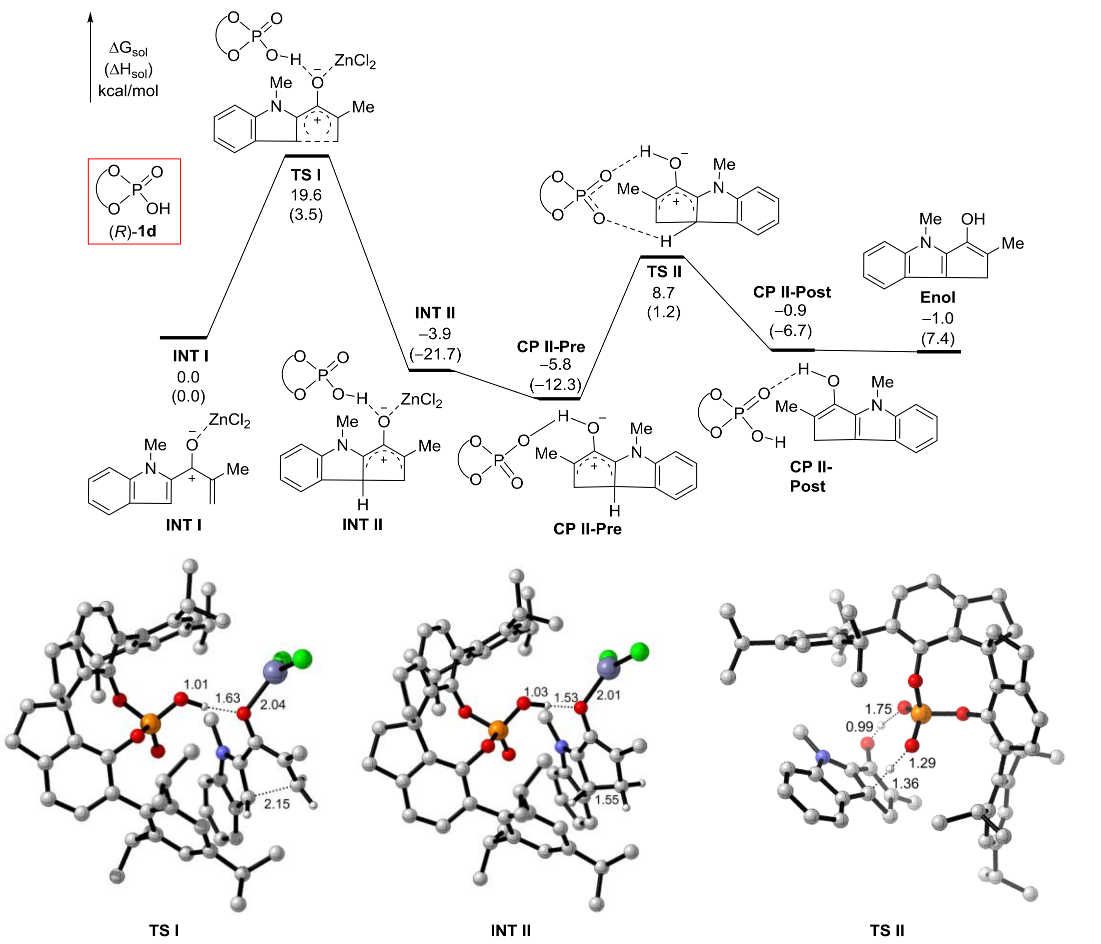

Figure S1. The computed energy surfaces for the co-catalyzed Nazarov cyclization (Ring-closing TS and [1,4]-proton transfer TS)

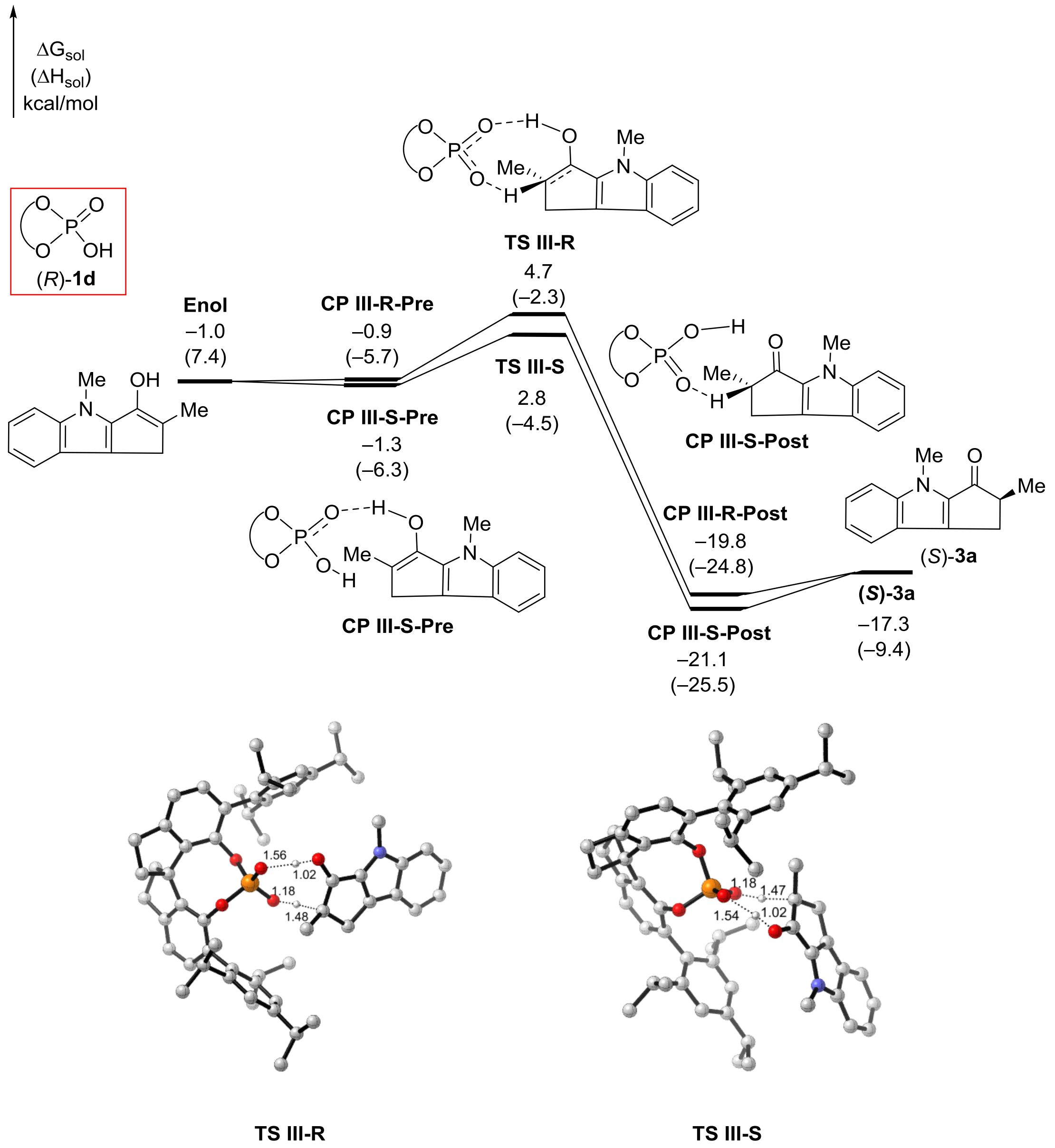

Figure S2. The computed energy surfaces for the co-catalyzed Nazarov cyclization ([1,3]-proton transfer TS)

Table S2. Comparision of energy barrier of the transition states with $ZnCl_2$ (SPA) and without $ZnCl_2$ (SPA)

Structure	ΔG_{sol}	ΔH_{sol}
TS I’	-3333.33560805	-3333.26641105
INT II’	-3333.36808853	-3333.29857453
CP II-Pre’	-5802.60321019	-5802.41996619
TS II’	-5802.57222496	-5802.39167696
CP II-Post’	-5802.59336534	-5802.40666734

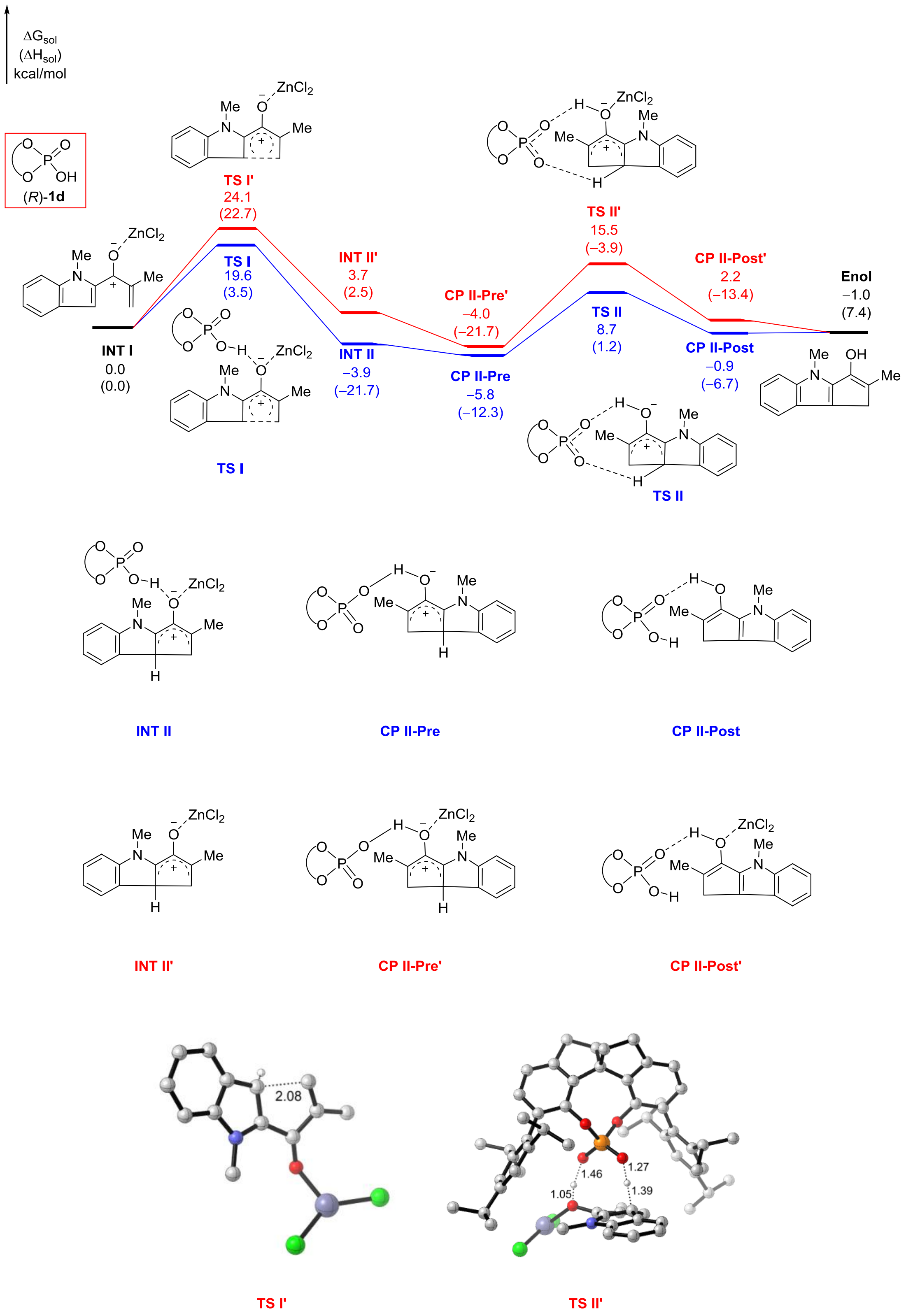

Figure S3. The computed energy surfaces for the transition states with $ZnCl_2$ (SPA) and without $ZnCl_2$ (SPA)

Table S3. Comparision of energy barrier of the [1,3]-proton transfer transition state promoted by other species

Structure	ΔG_{sol}	ΔH_{sol}
TS IV	-633.19775174	-633.14573474
CP V-Pre	-709.75277097	-709.69040197
TS V	-709.70654475	-709.65130975
CP V-Post	-709.77521984	-709.71542384
CP VI-Pre	-1266.57262375	-1266.48263675
TS VI	-1266.54231105	-1266.45709605
CP VI-Post	-1266.62191002	-1266.53092602

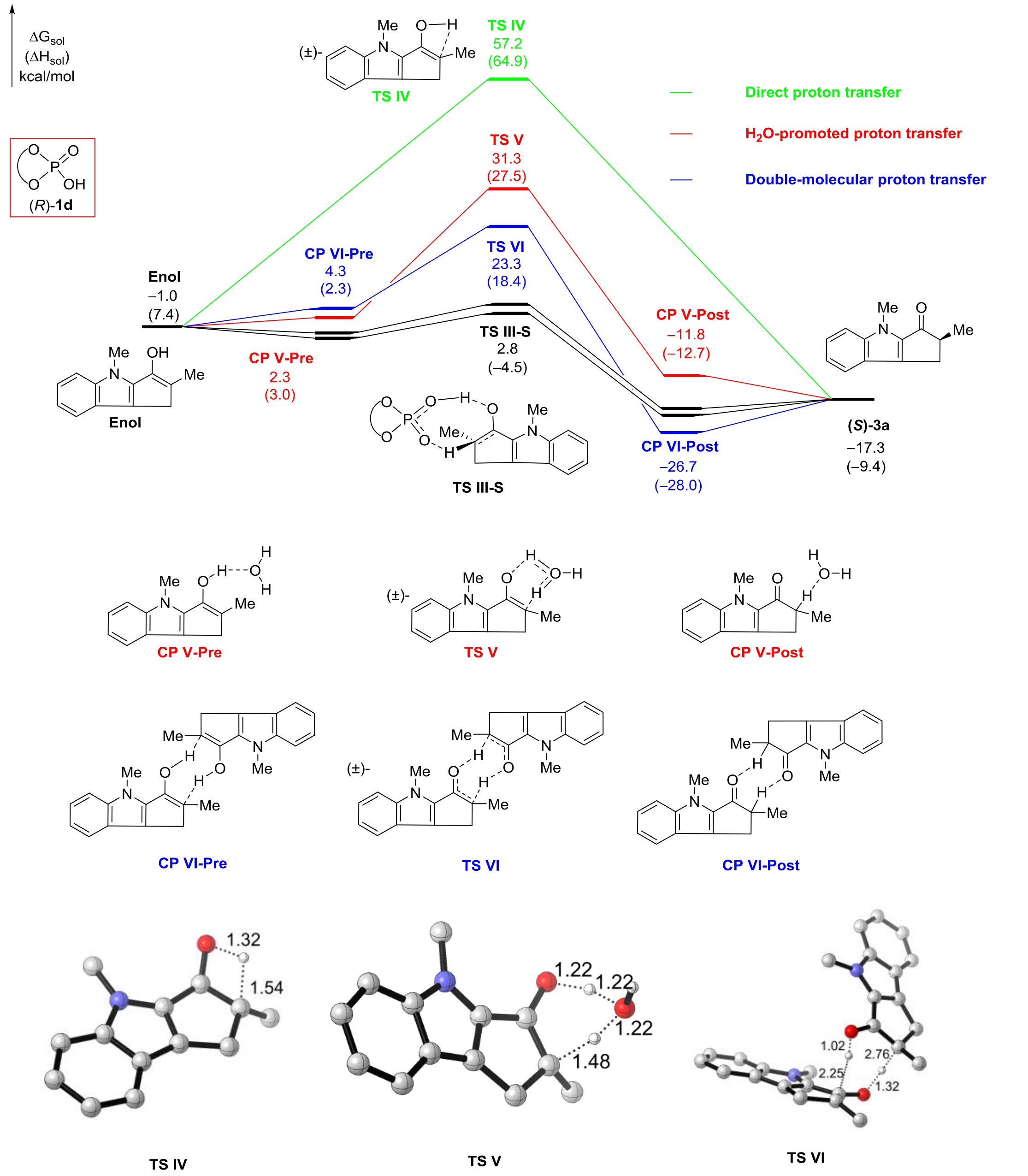

Figure S4. The computed energy surfaces for the [1,3]-proton transfer transition state promoted by other species

The Cartesian coordinates (Å) and energies at 298 K for the optimized structures.

Substrate

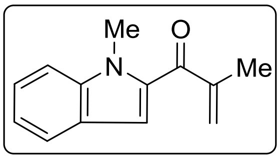

SCF energy[B3LYP-D3BJ/DEF2TZVP/SMD(1,2-dichloroethane)]: -633.48569745 a.u.
Thermal correction to Gibbs free energy at 298 K: 0.18799300 a.u.

Gibbs free energy at 298 K [B3LYP-D3BJ/DEF2TZVP/SMD(1,2-dichloroethane)]: -633.29770445 a.u.

C	-1.40754000	-0.93011300	0.16190300
C	-1.61509300	0.46635000	-0.02751800
C	-2.90332800	1.01358100	-0.13298300
C	-3.97913000	0.14372400	-0.02502100
C	-3.79318000	-1.24331100	0.17774500
C	-2.52198000	-1.78524300	0.27168800
C	0.00077600	-1.12601100	0.19039200
H	-3.05825900	2.07646700	-0.29040600
H	-4.98927100	0.53779000	-0.09674300
H	-4.66339300	-1.88868600	0.25781100
H	-2.38180400	-2.85249000	0.42232800
H	0.52434600	-2.05562900	0.35712800
N	-0.38910800	1.08880300	-0.08096800
C	-0.22466200	2.50817100	-0.36762900
H	0.81330200	2.78243300	-0.19869100
H	-0.51155800	2.73085700	-1.40278200
H	-0.86058900	3.09112400	0.30660100
C	0.60550700	0.11304800	0.03207200
C	2.04368100	0.42612500	0.12555300
O	2.44989700	1.54301100	0.45449000
C	3.04878000	-0.65438800	-0.16190300
C	4.35543100	-0.52166300	0.57821200
H	4.78982000	0.46755200	0.40266600
H	4.20511700	-0.60856900	1.66152400
H	5.06816400	-1.28890900	0.26276500
C	2.83623600	-1.58080900	-1.10820400
H	3.60484300	-2.30593600	-1.36541800
H	1.91086800	-1.62847100	-1.67176700

$ZnCl_2$

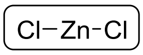

SCF energy[B3LYP-D3BJ/DEF2TZVP/SMD(1,2-dichloroethane)]: -2700.06118295 a.u.
Thermal correction to Gibbs free energy at 298 K: -0.02389000 a.u.

Gibbs free energy at 298 K [B3LYP-D3BJ/DEF2TZVP/SMD(1,2-dichloroethane)]: -2700.08507295 a.u.

Zn	1.55000000	0.31545741	0.00000000
Cl	-0.69000000	0.31545741	0.00000000
Cl	3.79000000	0.31545741	0.00000000

(*R*)-TRIP-SPA

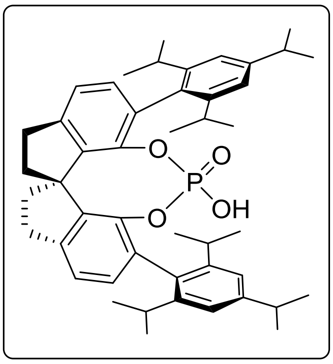

SCF energy[B3LYP-D3BJ/DEF2TZVP/SMD(1,2-dichloroethane)]: -2470.08964368 a.u.
Thermal correction to Gibbs free energy at 298 K: 0.86673300 a.u.

Gibbs free energy at 298 K [B3LYP-D3BJ/DEF2TZVP/SMD(1,2-dichloroethane)]: -2469.22291068 a.u.

P	-0.07818800	0.26266300	-0.33847000
O	1.00768700	-0.63435700	0.47017700
O	-1.24855400	-0.79229700	-0.76055500
O	-0.63987400	1.07508300	0.92715400
O	0.36095400	1.01193200	-1.52883700
C	1.75791600	-1.55268800	-0.26931300
C	3.04546700	-1.18706700	-0.71471800
C	3.73207500	-2.11205700	-1.52056200
C	3.14883900	-3.31496700	-1.92802200
C	1.88078500	-3.64911700	-1.45865700
C	1.03381400	-4.85847200	-1.78833500
C	-0.35026100	-4.45969700	-1.23787600

C	-0.03364500	-3.52393800	-0.02549900
C	1.20125100	-2.79970100	-0.56675600
C	0.33529400	-4.39451400	1.22439100
C	-1.02062600	-4.81258600	1.82472300
C	-1.90413000	-3.63594700	1.47792900
C	-3.14729500	-3.28904800	2.00203900
C	-3.75215300	-2.09671100	1.59380800
C	-3.12194400	-1.21342900	0.70084300
C	-1.88318600	-1.61833200	0.15490800
C	-1.28684800	-2.82972100	0.50479600
H	-1.44495900	1.57810900	0.71279400
H	4.73821500	-1.85788500	-1.85877400
H	3.68622000	-3.98221800	-2.60720900
H	1.42054600	-5.76648500	-1.29201000
H	1.01857900	-5.07487100	-2.86846800
H	-0.97721100	-5.31883100	-0.95680300
H	-0.90054200	-3.87795900	-1.99462200
H	0.98684100	-5.24341300	0.97102200
H	0.87976500	-3.76073800	1.94264200
H	-1.39740300	-5.74343800	1.36401400
H	-0.97248500	-4.99667600	2.90977000
H	-3.64082900	-3.92900400	2.73823700
H	-4.72574300	-1.82047100	2.00217000
C	-3.71323100	0.13050700	0.38011400
C	-3.83877200	1.12035000	1.39789900
C	-4.13826500	0.43360700	-0.94208000
C	-4.35130600	2.38207600	1.05380300
C	-4.64757400	1.70844800	-1.22068500
C	-4.75755600	2.70456800	-0.24448100
H	-4.44662200	3.14363100	1.83207900
H	-4.97029600	1.92802200	-2.24125200
C	3.68706400	0.11919000	-0.34116700
C	4.08699200	0.35147800	1.00219400
C	3.94580400	1.10450100	-1.32965500
C	4.73330200	1.55170800	1.32293800
C	4.60285300	2.28401000	-0.94924000
C	5.00495000	2.53453800	0.36475700
H	5.04090400	1.71940500	2.35848900
H	4.80361900	3.04454800	-1.70852100
C	3.55206900	0.93645600	-2.79919600
H	2.87773300	0.07111100	-2.86626500
C	4.78310900	0.64958900	-3.68024200
H	5.33886500	-0.23880900	-3.34227300
H	4.48015700	0.48043100	-4.72692200

H	5.48565500	1.49953000	-3.66993300
C	2.76815000	2.14227900	-3.34656500
H	3.38308400	3.05677800	-3.37850200
H	2.43921700	1.93686300	-4.37886000
H	1.87429500	2.32927200	-2.73754900
C	3.87335000	-0.67165700	2.11916900
H	3.36439500	-1.54411600	1.68664100
C	2.96439300	-0.12644000	3.23495700
H	1.99777500	0.20535000	2.82872500
H	2.76984700	-0.90583900	3.99087000
H	3.42812300	0.72753700	3.75574400
C	5.21137800	-1.18211400	2.68510500
H	5.84500900	-1.60964400	1.89191200
H	5.78428400	-0.37465600	3.16979400
H	5.03928300	-1.96579200	3.44167200
C	5.71057900	3.83427600	0.73138300
H	5.80189500	4.41887300	-0.20074900
C	4.88737600	4.67774700	1.72031400
H	5.38252700	5.64283500	1.91871900
H	3.88001900	4.88496600	1.32673600
H	4.76792600	4.16103300	2.68691100
C	7.13515300	3.59015300	1.25838000
H	7.12316100	3.02449600	2.20474800
H	7.73625100	3.01681200	0.53530700
H	7.65107100	4.54562200	1.45019600
C	-3.48988500	0.86164500	2.86874600
H	-2.91310800	-0.07226100	2.91849800
C	-4.77124300	0.66404500	3.70334300
H	-4.52052900	0.39859700	4.74355800
H	-5.37364600	1.58713900	3.72925800
H	-5.41192600	-0.13227300	3.29523000
C	-2.61456800	1.95590800	3.50620800
H	-3.13004000	2.92860600	3.55400500
H	-2.36171200	1.67493100	4.54137700
H	-1.66757200	2.09198700	2.96450600
C	-4.11308800	-0.59604800	-2.07213600
H	-3.61590700	-1.49953800	-1.69462200
C	-5.54241700	-1.01159600	-2.46876400
H	-6.10208000	-1.40369400	-1.60480100
H	-6.11287300	-0.16178000	-2.87845800
H	-5.51575900	-1.79756800	-3.24147000
C	-3.31069600	-0.11406700	-3.29351200
H	-3.79297100	0.74594300	-3.78713100
H	-2.28975600	0.18069300	-3.01068800

H	-3.23814400	-0.91917100	-4.04314700
C	-5.30178300	4.08947900	-0.57266900
H	-5.29997200	4.66271100	0.37090900
C	-4.39811600	4.83988400	-1.56647800
H	-4.77570900	5.86025700	-1.74388200
H	-3.36639500	4.91975600	-1.18979000
H	-4.35772200	4.32601400	-2.54083700
C	-6.75616200	4.03512700	-1.07145400
H	-6.83481900	3.49088700	-2.02683700
H	-7.41108000	3.53010500	-0.34420800
H	-7.14963400	5.05151100	-1.23651200

INT I

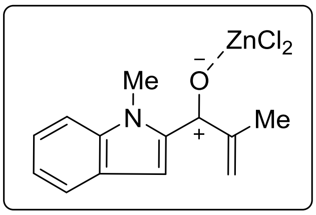

SCF energy[B3LYP-D3BJ/DEF2TZVP/SMD(1,2-dichloroethane)]: -3333.55485300 a.u.
Thermal correction to Gibbs free energy at 298 K: 0.18086700 a.u.

Gibbs free energy at 298 K [B3LYP-D3BJ/DEF2TZVP/SMD(1,2-dichloroethane)]: -3333.37398600 a.u.

C	3.56598100	0.67089200	0.17811300
C	3.10981900	-0.64201900	-0.16383300
C	4.02048900	-1.70547200	-0.33070300
C	5.36794800	-1.43518300	-0.14528900
C	5.83421100	-0.13937100	0.19996300
C	4.94875700	0.91011700	0.36382700
C	2.41461200	1.49014900	0.25860200
H	3.68813600	-2.71250000	-0.58652200
H	6.09237500	-2.24487500	-0.26386700
H	6.90576400	0.02223700	0.33732400
H	5.30297800	1.90910400	0.62954100
H	2.37424200	2.53938500	0.53694600
N	1.74124500	-0.61990700	-0.27261700
C	0.91852000	-1.76202900	-0.65546000
H	0.28164300	-2.11079800	0.16819400
H	0.26079500	-1.50077500	-1.49583400
H	1.57934800	-2.57518800	-0.97807000
C	1.30548800	0.68463600	-0.02195500

C	-0.09702600	1.04971100	0.06947900
O	-0.96054000	0.15490500	0.18796100
C	-0.50030500	2.48692700	0.07252000
C	-1.64720100	2.85840700	0.97630300
H	-2.57735900	2.37050400	0.64167700
H	-1.45710500	2.53609400	2.01352700
H	-1.80748400	3.94564500	0.97239700
C	0.07737300	3.35069800	-0.78301000
H	-0.25914800	4.39022800	-0.83695600
H	0.86203300	3.04350500	-1.47698000
Zn	-2.83416700	-0.65955600	0.05056800
Cl	-2.52347200	-2.75470800	0.51081300
Cl	-4.33756300	0.77644000	-0.56287900

TS I

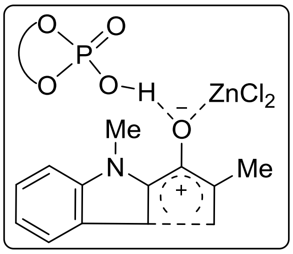

SCF energy[B3LYP-D3BJ/DEF2TZVP/SMD(1,2-dichloroethane)]: -5803.63914156 a.u.
Thermal correction to Gibbs free energy at 298 K: 1.07355500 a.u.

Gibbs free energy at 298 K [B3LYP-D3BJ/DEF2TZVP/SMD(1,2-dichloroethane)]: -5802.56558656 a.u.

C	1.72014400	-3.87808900	2.54108100
C	0.45294300	-3.33271800	2.88295700
C	-0.00665700	-3.31278600	4.20611700
C	0.83075900	-3.84673000	5.18315300
C	2.09695500	-4.38137600	4.86343800
C	2.55174400	-4.39433100	3.54869200
C	1.87766900	-3.68727000	1.11727800
H	-0.97609100	-2.88770900	4.46895600
H	0.50104500	-3.84330000	6.22499900
H	2.72527700	-4.78408600	5.66094000
H	3.53328900	-4.80553700	3.30075000
H	2.83909400	-3.43260400	0.67409300
N	-0.17345200	-2.86520400	1.72904400
C	-1.51641200	-2.29993100	1.69183400
H	-1.47665600	-1.23035600	1.94295600

H	-1.94886700	-2.43573500	0.69538900
H	-2.15163200	-2.83063000	2.41316700
C	0.64278600	-3.09578900	0.66955100
C	0.36806900	-3.05341800	-0.74510200
O	-0.45761700	-2.23371700	-1.32267600
C	1.18810100	-4.00620000	-1.40660900
C	1.54640500	-3.87530100	-2.85813800
H	0.70102800	-4.16362100	-3.50699400
H	1.78186100	-2.83168400	-3.11915100
H	2.39830400	-4.52207000	-3.11422600
C	1.69979200	-5.02273300	-0.56406600
H	2.60780300	-5.55700500	-0.87305800
H	1.04489000	-5.60278300	0.08517900
Zn	-1.92701300	-2.98938000	-2.52597700
Cl	-3.30253100	-4.03190100	-1.15488400
Cl	-1.51858600	-3.03403600	-4.66839800
P	0.12331700	1.09612200	0.14208900
O	1.20265600	2.00907100	-0.67481000
O	-0.93444600	2.17887400	0.73814500
O	-0.59695400	0.37582100	-1.06866500
O	0.65051900	0.24568200	1.23517000
C	2.02013500	2.92092800	-0.01707400
C	3.34099000	2.55348900	0.31989500
C	4.12436300	3.50434000	0.99927900
C	3.60922200	4.73696200	1.40386400
C	2.30161500	5.06756300	1.05879600
C	1.51391000	6.30630000	1.41986200
C	0.07551700	5.90620800	1.04106400
C	0.24753500	4.91296900	-0.15729800
C	1.52059000	4.19166700	0.28300400
C	0.48455500	5.72615200	-1.47395500
C	-0.92335100	6.18194600	-1.90314800
C	-1.80180800	5.06825100	-1.37925300
C	-3.13917900	4.80738200	-1.66463800
C	-3.74732400	3.68653300	-1.09499000
C	-3.03066500	2.76971300	-0.30577800
C	-1.67536600	3.06412400	-0.03577400
C	-1.07996200	4.23924700	-0.50085700
H	-0.49926700	-0.62510300	-1.07645200
H	5.15601000	3.24402700	1.24358600
H	4.22645300	5.42752200	1.98469200
H	1.85660000	7.18242300	0.84065600
H	1.62035100	6.57293000	2.48356400
H	-0.57003200	6.76179300	0.79470800

H	-0.39481200	5.36472400	1.87752100
H	1.19352600	6.55590600	-1.33830500
H	0.90703500	5.04820800	-2.23288400
H	-1.19128200	7.15206100	-1.44770300
H	-1.01917800	6.31083400	-2.99314100
H	-3.70935300	5.46672600	-2.32443300
H	-4.80287500	3.48809400	-1.29007200
C	-3.70275900	1.53010700	0.20701300
C	-4.18981800	0.55259900	-0.70568000
C	-3.89623500	1.33768200	1.59842700
C	-4.81950800	-0.59077600	-0.20094200
C	-4.54437600	0.17504100	2.04635500
C	-5.01179000	-0.80735800	1.16944600
H	-5.16892600	-1.35649500	-0.89773900
H	-4.69236700	0.04680900	3.12017600
C	3.94281100	1.21633800	-0.00452800
C	4.21245700	0.85137600	-1.35022900
C	4.33965100	0.34962200	1.04806300
C	4.89091000	-0.34764200	-1.60892500
C	5.01977300	-0.83513400	0.72894800
C	5.32030700	-1.20149400	-0.58650600
H	5.10960600	-0.60847100	-2.64752600
H	5.34116000	-1.49443000	1.54102600
C	4.06743500	0.65133300	2.52398700
H	3.45518100	1.56188200	2.57121200
C	5.36838400	0.92449800	3.30121800
H	5.95344900	1.74261900	2.85290300
H	5.14436200	1.20196300	4.34449800
H	6.01690000	0.03303600	3.32818200
C	3.24681100	-0.45797400	3.20264700
H	3.79388600	-1.41439400	3.23590200
H	3.02005300	-0.17720300	4.24470300
H	2.29570800	-0.60701200	2.67406700
C	3.83635400	1.73666300	-2.53898200
H	3.34050100	2.63498900	-2.14753700
C	2.83497800	1.04227600	-3.47925100
H	1.92236500	0.74890400	-2.94034800
H	2.54007200	1.71971700	-4.29762600
H	3.26920100	0.14013000	-3.94155700
C	5.08157500	2.22105700	-3.30445400
H	5.78429800	2.74493300	-2.63724000
H	5.62637900	1.38474900	-3.77250400
H	4.79368700	2.91797900	-4.10882300
C	6.14171200	-2.45343300	-0.87442000

H	6.18689400	-3.02713100	0.06866700
C	5.50659400	-3.36523300	-1.93544500
H	6.08729700	-4.29529800	-2.04931900
H	4.47403800	-3.63728700	-1.66602800
H	5.47180100	-2.87889500	-2.92366600
C	7.58957800	-2.09214100	-1.25502500
H	7.61942500	-1.51501900	-2.19391800
H	8.06649600	-1.48087200	-0.47330400
H	8.19861500	-3.00021400	-1.39855400
C	-4.08782300	0.70395200	-2.22615900
H	-3.47389000	1.58803800	-2.44227900
C	-5.47442800	0.93326300	-2.85729400
H	-5.38491800	1.11033000	-3.94170000
H	-6.12711300	0.05664500	-2.71443700
H	-5.98700600	1.80025200	-2.41209100
C	-3.38735700	-0.48281800	-2.90624800
H	-3.93890400	-1.42448500	-2.74866400
H	-3.30715600	-0.33436700	-3.99369400
H	-2.35913500	-0.57394200	-2.52222900
C	-3.48579900	2.37850500	2.64212500
H	-2.96924800	3.19570700	2.12077800
C	-4.72032900	3.00156400	3.32058200
H	-5.40210800	3.44798700	2.57956500
H	-5.29293000	2.25182400	3.89082400
H	-4.41710300	3.79424200	4.02476700
C	-2.50171000	1.81621300	3.68289400
H	-2.96148400	1.01777800	4.28873100
H	-1.60115200	1.41104800	3.19883300
H	-2.18410900	2.61039000	4.37895700
C	-5.74636700	-2.06481700	1.62703000
H	-5.36672300	-2.88687900	0.99475800
C	-5.49229900	-2.45026000	3.08899200
H	-5.95334800	-3.42551200	3.31190700
H	-4.41656900	-2.52846200	3.31257500
H	-5.92744500	-1.71834400	3.78949300
C	-7.25948200	-1.94086000	1.36033600
H	-7.69731600	-1.12459900	1.95915900
H	-7.46585600	-1.72507900	0.30104400
H	-7.78256000	-2.87504200	1.62350700

INT II

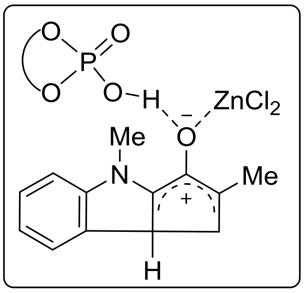

SCF energy[B3LYP-D3BJ/DEF2TZVP/SMD(1,2-dichloroethane)]: -5803.68160200 a.u.
Thermal correction to Gibbs free energy at 298 K: 1.07841500 a.u.

Gibbs free energy at 298 K [B3LYP-D3BJ/DEF2TZVP/SMD(1,2-dichloroethane)]: -5802.60318700 a.u.

Zn	-2.15212200	-2.95278000	-2.45166400
Cl	-3.44981300	-3.92592000	-0.95668700
Cl	-1.89585100	-3.12606800	-4.61729300
P	0.17935500	1.07522000	0.00073200
O	1.31760200	2.00579800	-0.71247700
O	-0.81813400	2.15506200	0.70573000
O	-0.56338900	0.51521200	-1.26797000
O	0.66638700	0.10301200	1.01294000
C	2.13995000	2.85064200	0.02086300
C	3.43457200	2.42130800	0.38751600
C	4.23184700	3.31642400	1.12406700
C	3.75242000	4.55439800	1.55476600
C	2.46732300	4.94392100	1.18755900
C	1.71550800	6.19697700	1.57354800
C	0.27199100	5.85555700	1.16045800
C	0.43030400	4.89740200	-0.06875200
C	1.67602600	4.12481900	0.36176100
C	0.69654200	5.74589300	-1.35628800
C	-0.69681200	6.25390400	-1.77642200
C	-1.61018600	5.15213400	-1.28735600
C	-2.95603200	4.94270900	-1.57555200
C	-3.59941300	3.82743400	-1.03387100
C	-2.91081000	2.86315200	-0.27676100
C	-1.54297800	3.10009200	-0.01265100
C	-0.91357300	4.27216900	-0.43905400
H	-0.57652500	-0.50842300	-1.34442800
H	5.24465900	3.00747100	1.39023200
H	4.37800500	5.20202700	2.17476200

H	2.09614700	7.07696200	1.02465900
H	1.81335400	6.42986000	2.64594100
H	-0.34316000	6.73854200	0.93405900
H	-0.22751800	5.30187800	1.97166200
H	1.42674500	6.55155800	-1.19024900
H	1.10464600	5.08199600	-2.13519500
H	-0.93865400	7.21873600	-1.29582500
H	-0.78419400	6.41545500	-2.86279300
H	-3.50688600	5.63930100	-2.21311100
H	-4.66268500	3.67297200	-1.22579600
C	-3.62785500	1.64247700	0.22182500
C	-4.17747100	0.70438600	-0.69743200
C	-3.80711900	1.43488400	1.61338600
C	-4.85255600	-0.41600000	-0.19914400
C	-4.50592900	0.29920800	2.05405800
C	-5.03460400	-0.64530800	1.17052000
H	-5.24947900	-1.15265700	-0.90146800
H	-4.64407500	0.16175000	3.12806100
C	3.99300600	1.07134600	0.04094900
C	4.29676900	0.73661400	-1.30258200
C	4.31045200	0.15237200	1.07916700
C	4.92648300	-0.48781900	-1.57831900
C	4.93654400	-1.05556400	0.74432700
C	5.27279200	-1.39474700	-0.57314800
H	5.17119600	-0.72117400	-2.61612900
H	5.19673400	-1.75378900	1.54646900
C	4.02105700	0.43204000	2.55702800
H	3.47943100	1.38520400	2.61755900
C	5.31970500	0.58805100	3.37009800
H	5.97560400	1.36773800	2.95204700
H	5.09209400	0.86161100	4.41360800
H	5.89823300	-0.35049600	3.39305500
C	3.10159900	-0.62662400	3.18828600
H	3.56794200	-1.62603300	3.19914800
H	2.88142900	-0.36260000	4.23604600
H	2.14996400	-0.67563800	2.64141300
C	4.01720600	1.68041700	-2.47265400
H	3.54361600	2.58576400	-2.07007300
C	3.03331900	1.07053300	-3.48670300
H	2.08266300	0.80095600	-3.00454200
H	2.81140800	1.79356100	-4.28883300
H	3.44762600	0.16769100	-3.96540900
C	5.31961800	2.13475400	-3.15748100
H	6.00804600	2.60572100	-2.43794300

H	5.85255300	1.29162500	-3.62706500
H	5.10196900	2.86937500	-3.95022600
C	6.06104800	-2.67335400	-0.84490800
H	5.69990000	-3.42805100	-0.12148500
C	5.86703200	-3.25321700	-2.25173600
H	6.37121900	-4.22885100	-2.33778200
H	4.80285100	-3.39895400	-2.49529900
H	6.29809100	-2.59829900	-3.02595900
C	7.55964100	-2.45570300	-0.55477800
H	7.98300000	-1.70416000	-1.24136700
H	7.72284800	-2.09641000	0.47279300
H	8.12702900	-3.39237400	-0.68391800
C	-4.10362300	0.88013000	-2.21721700
H	-3.43412000	1.72384800	-2.43134700
C	-5.48854600	1.21874400	-2.80226400
H	-5.41534200	1.42097900	-3.88347100
H	-6.19296100	0.38188800	-2.66598600
H	-5.93342400	2.10310900	-2.32001400
C	-3.50903900	-0.33726100	-2.94147100
H	-4.10095800	-1.25068200	-2.76407000
H	-3.48197400	-0.18416800	-4.03100600
H	-2.46843300	-0.49440900	-2.62283600
C	-3.32307000	2.43046800	2.66942000
H	-2.78530200	3.23750700	2.15394400
C	-4.50810200	3.09002400	3.39941000
H	-5.19160200	3.58178900	2.68929900
H	-5.09602600	2.35350900	3.97131500
H	-4.15048700	3.85352600	4.11035600
C	-2.33521500	1.79713700	3.66511000
H	-2.81469400	1.00824300	4.26840600
H	-1.47270000	1.36069400	3.14027600
H	-1.95508100	2.55745900	4.36763100
C	-5.81932000	-1.87446700	1.62102800
H	-5.48435000	-2.70310200	0.97247800
C	-5.56201200	-2.29165200	3.07374200
H	-6.06461500	-3.24752600	3.29076200
H	-4.48779000	-2.42372700	3.27928900
H	-5.95141700	-1.55031700	3.79119400
C	-7.32882000	-1.67923000	1.37817100
H	-7.72273400	-0.85524600	1.99669900
H	-7.53978200	-1.43639900	0.32564600
H	-7.88900600	-2.59416100	1.63244400
C	1.72768000	-5.02929200	4.43225000
C	0.50448600	-4.47479500	4.82446700

C	-0.25070200	-3.68771000	3.94308300
C	0.26589800	-3.49115700	2.66733900
C	1.50044000	-4.03600000	2.25448900
C	2.24182200	-4.80296600	3.14493100
N	-0.33023500	-2.78571800	1.57913000
C	0.46456900	-2.86633500	0.53054100
C	-1.64510800	-2.16036500	1.63433000
C	0.30117300	-2.72507900	-0.89585700
C	1.26769300	-3.53530500	-1.46806700
C	2.11493700	-4.24965700	-0.43466700
C	1.45359600	-3.74349100	-2.92970700
H	2.29145000	-5.64396200	5.13765700
H	0.12658700	-4.65836900	5.83288800
H	-1.20511500	-3.25698400	4.24882400
H	3.20420200	-5.22914800	2.85151700
H	-1.91533500	-1.81536000	0.63352600
H	-2.38982900	-2.89802900	1.96182100
H	-1.61286900	-1.30769200	2.32676700
H	3.18721800	-4.22804300	-0.68600300
H	1.81887100	-5.31072300	-0.36267800
H	0.64951200	-3.28019200	-3.51841700
H	2.41737400	-3.30471900	-3.24909500
H	1.49934000	-4.81724400	-3.18008600
O	-0.64921300	-2.02474900	-1.49043500
C	1.76586100	-3.49853900	0.87142100
H	2.48871300	-2.66325400	0.97556200

TS I’

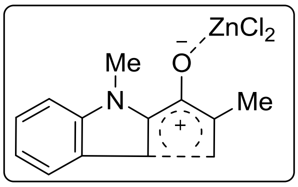

SCF energy[B3LYP-D3BJ/DEF2TZVP/SMD(1,2-dichloroethane)]: -3333.51682905 a.u.

Thermal correction to Gibbs free energy at 298 K: 0.18122100 a.u.

Gibbs free energy at 298 K [B3LYP-D3BJ/DEF2TZVP/SMD(1,2-dichloroethane)]: -3333.33560805 a.u.

C	-3.45478600	0.66067000	-0.12736200
C	-3.09514100	-0.71097300	-0.04627900
C	-4.03963800	-1.70934800	0.21982500
C	-5.36523000	-1.30848600	0.38567100

C	-5.74463600	0.04504500	0.28513300
C	-4.79825300	1.03419300	0.02456600
C	-2.23455000	1.39365000	-0.43484900
H	-3.75635500	-2.76041900	0.29412500
H	-6.12741900	-2.06316800	0.59445200
H	-6.79416800	0.31841300	0.41474200
H	-5.09483000	2.08343500	-0.04917800
H	-2.25378700	2.22427000	-1.14045100
N	-1.72139200	-0.84102100	-0.26859300
C	-0.98345200	-2.09022000	-0.11421600
H	-1.38022500	-2.84137400	-0.81398400
H	0.07946800	-1.93043800	-0.32877200
H	-1.08859700	-2.47285300	0.91336100
C	-1.19513300	0.39882700	-0.45187600
C	0.18773400	0.83092400	-0.42013900
O	1.16240300	0.11368500	-0.82157100
C	0.20903300	2.17993500	0.04145600
C	1.36099800	3.09139200	-0.25265000
H	2.26491500	2.74595200	0.28077900
H	1.62504400	3.07038500	-1.32351500
H	1.15191900	4.12641800	0.05399300
C	-0.97736800	2.58801100	0.71275400
H	-1.21328500	3.66125400	0.74171700
H	-1.34491700	2.04964400	1.58733700
Zn	2.90142000	-0.48743200	-0.05929200
Cl	2.74920700	-2.66065200	0.12158800
Cl	4.33448000	1.07049800	0.47104000

INT II’

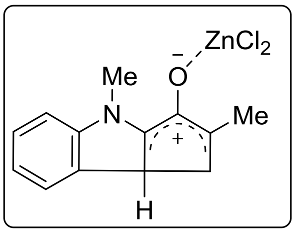

SCF energy[B3LYP-D3BJ/DEF2TZVP/SMD(1,2-dichloroethane)]: -3333.55119753 a.u.

Thermal correction to Gibbs free energy at 298 K: 0.18310900 a.u.

Gibbs free energy at 298 K [B3LYP-D3BJ/DEF2TZVP/SMD(1,2-dichloroethane)]: -3333.36808853 a.u.

C	-5.60030100	0.04829300	-0.40948400
C	-5.31576000	-1.25409800	0.01445200

C	-3.99621800	-1.66954300	0.24622900
C	-2.98883200	-0.73194400	0.04375100
C	-3.25615100	0.58743800	-0.38494200
C	-4.56830300	0.97677900	-0.62425800
N	-1.58627300	-0.89174200	0.25175500
C	-0.98425400	0.26646000	0.00521800
C	-0.96936800	-2.08958900	0.81456700
C	0.26466500	0.85638300	0.42257000
C	0.04407800	2.23704700	0.34797500
C	-1.36687900	2.59587700	-0.06087500
C	1.07172700	3.24097700	0.73309700
H	-6.63760500	0.34612800	-0.57801800
H	-6.13281100	-1.96219200	0.17097800
H	-3.77756800	-2.68525700	0.57940400
H	-4.79857500	1.98836000	-0.96745600
H	0.12300300	-1.99312300	0.77304200
H	-1.30263300	-2.23123800	1.85474000
H	-1.26881200	-2.96396100	0.21827200
H	-1.40128000	3.42093200	-0.79233500
H	-1.96054300	2.91907100	0.81266800
H	2.08084000	2.81056300	0.64730800
H	1.00277500	4.14349800	0.10286600
H	0.93214600	3.57490200	1.77935100
O	1.28336200	0.22162700	0.88890600
Zn	2.86687000	-0.49370600	-0.01781100
Cl	2.55714000	-2.64448000	-0.34378700
Cl	4.33705600	1.04252000	-0.50857700
C	-1.91703200	1.25198500	-0.60657200
H	-1.73360200	1.24959000	-1.70021600

CP II-Pre

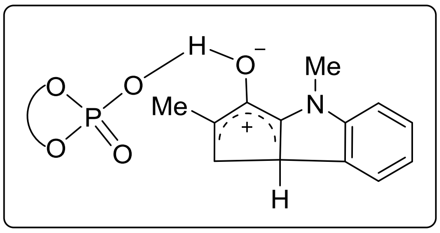

SCF energy[B3LYP-D3BJ/DEF2TZVP/SMD(1,2-dichloroethane)]: -3103.60340257 a.u.

Thermal correction to Gibbs free energy at 298 K: 1.08233100 a.u.

Gibbs free energy at 298 K [B3LYP-D3BJ/DEF2TZVP/SMD(1,2-dichloroethane)]: -3102.52107157 a.u.

C	-1.88576500	-4.28156300	-0.85761700
C	-0.50029100	-4.07313500	-1.00747500
C	0.24658200	-4.68401600	-2.00885400
C	-0.43625700	-5.54619500	-2.87871500
C	-1.81089600	-5.77033400	-2.74375500
C	-2.54983000	-5.12861800	-1.73666000
C	-2.34395800	-3.37241400	0.25200000
H	1.31722500	-4.50588300	-2.11795900
H	0.11772400	-6.04845400	-3.67512700
H	-2.31661900	-6.44926600	-3.43411100
H	-3.62568300	-5.29746700	-1.64778500
H	-2.86025100	-2.50331200	-0.20604600
N	-0.03535100	-3.20560500	0.02493700
C	1.36793000	-2.85741200	0.19579200
H	1.74193400	-2.40949200	-0.73423100
H	1.46796700	-2.11677900	0.99288100
H	1.94933000	-3.76049600	0.43335300
C	-1.05324200	-2.85707000	0.78819900
C	-1.21177200	-2.37682800	2.14482100
O	-0.35606600	-1.68224300	2.86713700
C	-2.39343500	-2.92952600	2.59951000
C	-2.89186800	-2.78610200	3.99441200
H	-2.22592300	-2.13733300	4.57898700
H	-3.90681500	-2.35041100	3.99897600
H	-2.96873500	-3.76617200	4.49835400
C	-3.10288700	-3.75260600	1.54728900
H	-4.18271700	-3.53737100	1.50859400
H	-2.99439600	-4.83280400	1.74892900
P	0.25865800	0.58748400	0.17382600
O	-0.68116500	1.91327600	0.51480400
O	1.55324400	1.27197200	-0.60612000
O	0.71941200	0.10746800	1.54438900
O	-0.39529700	-0.34447100	-0.79603800
C	-1.17197800	2.72711000	-0.48069200
C	-2.48077700	2.53298800	-0.97958500
C	-2.93388600	3.40084500	-1.99016600
C	-2.11379600	4.38329100	-2.54785400
C	-0.82630000	4.54897700	-2.04368200
C	0.25331500	5.50118600	-2.50564800
C	1.50680000	4.94203600	-1.80786000
C	0.97174400	4.30665900	-0.47962200
C	-0.37026300	3.76580300	-0.96918900
C	0.78435800	5.42791000	0.59489400
C	2.20196500	5.67207300	1.14978500

C	2.85385400	4.31538400	0.99571100
C	4.05618900	3.85747700	1.52808500
C	4.44840500	2.54063900	1.27522600
C	3.62865200	1.63754600	0.57521200
C	2.40671800	2.12662700	0.05674000
C	2.06162400	3.47525800	0.19402700
H	0.09680300	-0.91072200	2.31620300
H	-3.95248200	3.27480900	-2.36330800
H	-2.47997700	5.00664000	-3.36811300
H	0.03795900	6.53597500	-2.18285100
H	0.34747300	5.52890900	-3.60327700
H	2.28922800	5.69495800	-1.63185900
H	1.94817800	4.14112900	-2.42256100
H	0.30849900	6.33200300	0.18666200
H	0.13221200	5.03845700	1.39290500
H	2.73795800	6.44311700	0.56744300
H	2.19916300	6.01725000	2.19640600
H	4.69013200	4.51437400	2.12989000
H	5.40906100	2.18624100	1.65227000
C	4.08322800	0.21885000	0.38092400
C	4.29525900	-0.63222900	1.50039400
C	4.38155100	-0.26428200	-0.92276500
C	4.81239600	-1.91973500	1.28686800
C	4.88933600	-1.56245800	-1.07430800
C	5.12519600	-2.40815300	0.01511700
H	4.98824900	-2.56763700	2.15022400
H	5.13069200	-1.91190900	-2.08166900
C	-3.40688300	1.45878800	-0.48531900
C	-3.94486100	1.51446600	0.82449100
C	-3.83036900	0.42658600	-1.36812600
C	-4.91666000	0.57623400	1.21008900
C	-4.79945200	-0.48498300	-0.92831400
C	-5.37852200	-0.42013000	0.34655100
H	-5.33733000	0.65292200	2.21428900
H	-5.13937200	-1.26454300	-1.61775000
C	-3.29096800	0.27800500	-2.79336000
H	-2.49641100	1.02377000	-2.92620700
C	-4.37987600	0.56245900	-3.84500100
H	-4.83515500	1.55594900	-3.70886500
H	-3.95515600	0.52245600	-4.86179100
H	-5.19244100	-0.18174100	-3.79655800
C	-2.63864700	-1.09365000	-3.03462400
H	-3.36151100	-1.92068400	-2.92960900
H	-2.23512900	-1.14586900	-4.05972800

H	-1.80656600	-1.23866100	-2.33229600
C	-3.54876000	2.59226600	1.83435700
H	-2.80377500	3.24199700	1.35722600
C	-2.88494000	1.99119600	3.08590600
H	-1.99690600	1.40086700	2.81818400
H	-2.56339300	2.79236600	3.77211200
H	-3.58083900	1.34136100	3.64314300
C	-4.74337100	3.48907100	2.20852200
H	-5.19609700	3.94824200	1.31534100
H	-5.53328100	2.92410000	2.73062600
H	-4.42025400	4.30135400	2.88047200
C	-6.52479000	-1.36509800	0.70089500
H	-6.27959000	-2.34631900	0.25231100
C	-6.72914300	-1.58327900	2.20490000
H	-7.49397800	-2.35632300	2.38175600
H	-5.80146800	-1.90306700	2.70466800
H	-7.07573100	-0.66501000	2.70583600
C	-7.83787100	-0.88916500	0.04745700
H	-8.13436100	0.09451100	0.44742400
H	-7.73067100	-0.78521800	-1.04295800
H	-8.65861500	-1.59896000	0.24474900
C	4.01065500	-0.20182500	2.94258200
H	3.43721600	0.73405500	2.90038100
C	5.31626000	0.06688000	3.71698100
H	5.09562400	0.44362000	4.72950600
H	5.90973400	-0.85621100	3.82753000
H	5.95518200	0.80806400	3.21420100
C	3.14847600	-1.21199200	3.71885000
H	3.66344700	-2.17707100	3.85858100
H	2.92546400	-0.81831000	4.72406400
H	2.18803400	-1.39874900	3.22224300
C	4.22480300	0.58999700	-2.18243400
H	3.83186700	1.56913300	-1.88049200
C	5.58301900	0.85141600	-2.85946300
H	6.29486000	1.32034500	-2.16167000
H	6.04317900	-0.07929000	-3.23053900
H	5.46021800	1.52601200	-3.72304500
C	3.21107300	-0.01514800	-3.16968700
H	3.55175800	-0.99078200	-3.55635900
H	2.22959300	-0.14860600	-2.69201600
H	3.07635400	0.65091800	-4.03827600
C	5.74939900	-3.78858700	-0.15827200
H	5.71485300	-4.27896000	0.83047800
C	4.97302700	-4.68270200	-1.13899900

H	5.42012500	-5.68914300	-1.18864400
H	3.92083400	-4.79720300	-0.83258200
H	4.98217900	-4.26852300	-2.16056600
C	7.23087100	-3.68515000	-0.56498500
H	7.33920400	-3.20429900	-1.55120000
H	7.80159100	-3.08591300	0.16124400
H	7.69484200	-4.68383900	-0.62505100

TS II

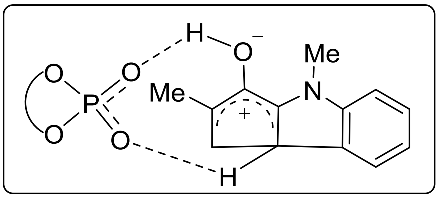

SCF energy[B3LYP-D3BJ/DEF2TZVP/SMD(1,2-dichloroethane)]: -3103.57777370 a.u.
Thermal correction to Gibbs free energy at 298 K: 1.07977500 a.u.

Gibbs free energy at 298 K [B3LYP-D3BJ/DEF2TZVP/SMD(1,2-dichloroethane)]: -3102.49799870 a.u.

C	-0.17981300	-3.92844400	1.20962300
C	-1.54161700	-3.92785100	0.79938300
C	-2.57051000	-4.40241000	1.61295600
C	-2.21544100	-4.92398200	2.86092400
C	-0.87480300	-4.95456100	3.27709600
C	0.14636700	-4.45016700	2.46558400
C	0.56427300	-3.24355100	0.14110100
H	-3.61309300	-4.37192400	1.29371000
H	-2.99509600	-5.31319800	3.52003100
H	-0.62676800	-5.37348800	4.25541600
H	1.18313500	-4.46464500	2.80748400
H	0.53130500	-1.92762400	0.46372300
N	-1.62624000	-3.40254800	-0.51053900
C	-2.84515400	-3.31179300	-1.29693300
H	-3.53025300	-2.56431900	-0.87111300
H	-2.58126200	-3.01506500	-2.31810400
H	-3.34609000	-4.29201700	-1.31926600
C	-0.38887800	-3.07966700	-0.90555400
C	0.26739300	-2.69680400	-2.13795900
O	-0.34322400	-2.11177800	-3.19416000
C	1.60338900	-2.93636600	-1.97703900
C	2.64629300	-2.65507500	-3.00166700
H	2.18770000	-2.28599900	-3.92927800

H	3.35931900	-1.89902500	-2.63232000
H	3.23215300	-3.56195600	-3.23218600
C	1.90755300	-3.42851800	-0.57401500
H	2.74704500	-2.86325700	-0.13837600
H	2.20320000	-4.49328800	-0.57681000
P	0.09747900	0.42980700	-0.29258000
O	1.34766100	1.49040000	-0.47876600
O	-1.02999600	1.29358100	0.54962900
O	-0.38531400	0.07826600	-1.66829700
O	0.52448500	-0.65791100	0.70772500
C	1.98803300	2.13480000	0.56448600
C	3.25223900	1.68709600	1.01452400
C	3.85879900	2.39031300	2.07293400
C	3.22929400	3.45190300	2.72109800
C	1.99187800	3.88063700	2.25104500
C	1.11925100	4.99233300	2.78606500
C	-0.20625600	4.75978600	2.04048800
C	0.21633300	4.12147900	0.67362000
C	1.39878200	3.27241300	1.13040800
C	0.67943700	5.24994900	-0.30623700
C	-0.63209700	5.85481600	-0.84259200
C	-1.58465700	4.68189900	-0.78297900
C	-2.86917000	4.57636900	-1.30777400
C	-3.57157800	3.38627000	-1.12098800
C	-2.99475900	2.25336500	-0.51386300
C	-1.67571200	2.37944800	-0.01903000
C	-1.01717600	3.61401700	-0.06656300
H	-0.45431600	-1.17121100	-2.89082400
H	4.84761100	2.06807300	2.40485000
H	3.70694400	3.94067700	3.57443700
H	1.54782700	5.98349700	2.55225700
H	1.01103600	4.94900300	3.88187400
H	-0.80915400	5.67067900	1.91342800
H	-0.82111200	4.02874700	2.58958500
H	1.33971400	5.98571700	0.17603000
H	1.24421500	4.78575400	-1.13054500
H	-0.98428300	6.68888600	-0.20942800
H	-0.53268600	6.25864900	-1.86316200
H	-3.33092900	5.41230500	-1.83999400
H	-4.60043700	3.31059100	-1.47605900
C	-3.85082100	1.02412400	-0.39780300
C	-4.39966100	0.43287200	-1.56909300
C	-4.26204800	0.54641500	0.87702400
C	-5.37456900	-0.56734200	-1.43435300

C	-5.22406600	-0.47165200	0.94824100
C	-5.81863600	-1.02633500	-0.19074600
H	-5.81591600	-1.00064300	-2.33679300
H	-5.54483300	-0.81402100	1.93561300
C	4.01175400	0.53429200	0.42542500
C	4.53269700	0.61094200	-0.89253300
C	4.34415300	-0.58254700	1.24115300
C	5.37806400	-0.40990400	-1.35606200
C	5.18602200	-1.57543000	0.72137800
C	5.73319500	-1.50739500	-0.56500100
H	5.79230700	-0.32316500	-2.36291100
H	5.44817700	-2.42907100	1.35409300
C	3.84931000	-0.74260600	2.68241100
H	3.19952400	0.11148700	2.91228700
C	5.01321200	-0.72120300	3.69071000
H	5.62607300	0.18829400	3.59080700
H	4.62977400	-0.75825100	4.72372300
H	5.68363000	-1.58597000	3.55575000
C	2.98688400	-2.00079600	2.87232100
H	3.54354000	-2.92370700	2.63717700
H	2.64804900	-2.07682200	3.91877700
H	2.09452000	-1.95255300	2.23535600
C	4.27090300	1.80243300	-1.81556500
H	3.63713300	2.51539200	-1.27251800
C	3.50192300	1.39626000	-3.08496600
H	2.53919000	0.92931000	-2.83182600
H	3.29254400	2.28236100	-3.70688500
H	4.08098800	0.69067200	-3.70404100
C	5.57307200	2.54794700	-2.16148500
H	6.10636100	2.86662600	-1.25178300
H	6.26250300	1.92102800	-2.75046700
H	5.35441000	3.44810700	-2.75957400
C	6.74555700	-2.55692300	-1.01467300
H	6.51315100	-3.48013500	-0.45384000
C	6.68070500	-2.90051400	-2.50881300
H	7.36792600	-3.72972700	-2.74160500
H	5.66783000	-3.20389900	-2.81348400
H	6.98009100	-2.04752900	-3.13948100
C	8.17275100	-2.13157700	-0.61619900
H	8.46535000	-1.20725700	-1.14136000
H	8.24642400	-1.93719200	0.46493900
H	8.90475400	-2.91496400	-0.87431400
C	-3.97966400	0.83767400	-2.98534500
H	-3.16896200	1.57288300	-2.89544900

C	-5.12931100	1.49325300	-3.77237200
H	-4.77680100	1.82937500	-4.76138400
H	-5.95753900	0.78521800	-3.94072600
H	-5.54571900	2.36737600	-3.24890100
C	-3.40238700	-0.34859800	-3.77522100
H	-4.15650300	-1.13268900	-3.95729300
H	-3.03774100	-0.01196200	-4.75946200
H	-2.55641400	-0.80070300	-3.24339900
C	-3.75471600	1.13932900	2.19299200
H	-3.06904700	1.96133500	1.95222900
C	-4.89943700	1.75403400	3.01986100
H	-5.45650200	2.50576100	2.43828900
H	-5.62034600	0.99179800	3.35767700
H	-4.49977400	2.24971700	3.91998500
C	-2.95429500	0.11332600	3.01445300
H	-3.57474200	-0.75219400	3.30138900
H	-2.08583400	-0.25400400	2.44930700
H	-2.58053200	0.57383300	3.94417400
C	-6.94476000	-2.05034900	-0.09331600
H	-7.18957300	-2.35176400	-1.12698900
C	-6.53864600	-3.32272000	0.66830200
H	-7.36952000	-4.04673400	0.69073600
H	-5.67753400	-3.81677100	0.19155000
H	-6.26467500	-3.10078300	1.71264100
C	-8.21654300	-1.42878200	0.51223700
H	-8.05004700	-1.11489400	1.55581600
H	-8.53501300	-0.54084200	-0.05558100
H	-9.04782500	-2.15321500	0.50919500

CP II-Post

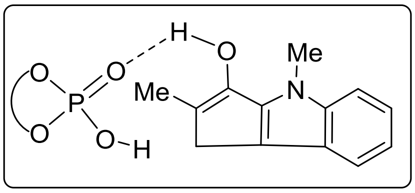

SCF energy[B3LYP-D3BJ/DEF2TZVP/SMD(1,2-dichloroethane)]: -3103.59500213 a.u.
Thermal correction to Gibbs free energy at 298 K: 1.08178100 a.u.

Gibbs free energy at 298 K [B3LYP-D3BJ/DEF2TZVP/SMD(1,2-dichloroethane)]: -3102.51322113 a.u.

C	1.45612300	-4.50031900	1.17408900
C	0.28124600	-3.75305000	1.49654700

C	-0.30461600	-3.80900600	2.76020800
C	0.30490600	-4.61086600	3.73133400
C	1.47299900	-5.33781000	3.43919400
C	2.05471900	-5.28969300	2.17219800
C	1.72407300	-4.22809600	-0.21118400
H	-1.20876200	-3.24320300	2.99480000
H	-0.13419100	-4.67063800	4.73008800
H	1.92934200	-5.95321000	4.21886300
H	2.95902600	-5.86340500	1.95498500
H	0.29268100	-1.27460400	0.74334400
N	-0.13531100	-3.00176500	0.36080100
C	-1.56905000	-2.84887300	0.07617700
H	-2.07771700	-2.39265200	0.93513200
H	-1.70325400	-2.18860600	-0.78694500
H	-2.03411800	-3.82537200	-0.13727900
C	0.76022500	-3.36716100	-0.66217800
C	0.95492800	-3.06590800	-2.08451300
O	0.19198800	-2.26513300	-2.85506800
C	2.03542900	-3.78617800	-2.51352200
C	2.57756700	-3.80074900	-3.90539500
H	2.04124600	-3.07263900	-4.53094500
H	3.65337100	-3.55107700	-3.93102900
H	2.47466200	-4.79458600	-4.37973800
C	2.62380800	-4.58383700	-1.35904000
H	3.68006400	-4.30735400	-1.18444000
H	2.62014500	-5.67097500	-1.56284400
P	-0.20798500	0.61483000	-0.22959800
O	0.84406300	1.81417600	-0.48203600
O	-1.46676800	1.29837300	0.54111900
O	-0.53973500	-0.04931500	-1.51976400
O	0.39057600	-0.29377000	0.92119200
C	1.34838200	2.64241800	0.52102100
C	2.65731500	2.42206500	1.00036500
C	3.11446900	3.27012800	2.02589200
C	2.30319300	4.25258700	2.59640000
C	1.02541500	4.45608500	2.08263600
C	-0.02897000	5.44123300	2.53291600
C	-1.28499200	4.94816100	1.79107000
C	-0.73861600	4.30315400	0.47250600
C	0.56161800	3.69512900	0.99429200
C	-0.45801500	5.42532600	-0.58158800
C	-1.84090800	5.74649300	-1.18259100
C	-2.56605800	4.42440200	-1.06600500
C	-3.77157800	4.03180200	-1.64103200

C	-4.24607100	2.73886200	-1.40733000
C	-3.50444300	1.78701400	-0.68410100
C	-2.27556500	2.21373500	-0.13654000
C	-1.84536100	3.53697700	-0.24737000
H	-0.05297500	-1.43720500	-2.37763500
H	4.13308100	3.13300200	2.39237700
H	2.67493200	4.85775100	3.42749100
H	0.23772100	6.47210900	2.23837700
H	-0.15303900	5.45092600	3.62761900
H	-2.02409700	5.73925400	1.59808400
H	-1.78446700	4.16402100	2.38253000
H	0.04734800	6.29897500	-0.14460600
H	0.20288600	5.01454500	-1.36162700
H	-2.35831100	6.53757600	-0.61096000
H	-1.78351600	6.10039000	-2.22435400
H	-4.34836400	4.72410100	-2.26004100
H	-5.21241100	2.43973900	-1.81529100
C	-4.05763900	0.40412900	-0.48720100
C	-4.26963500	-0.45597200	-1.59874800
C	-4.45470200	-0.02094000	0.80994800
C	-4.87711500	-1.70212700	-1.38269400
C	-5.05439400	-1.27872800	0.96312600
C	-5.28516300	-2.13629200	-0.11827600
H	-5.04533700	-2.36176000	-2.23800700
H	-5.36980100	-1.58793000	1.96302400
C	3.58265300	1.37617400	0.44732000
C	4.07492000	1.49001900	-0.88109000
C	4.05470000	0.32655400	1.27978600
C	5.03311400	0.57327300	-1.33255300
C	5.01584500	-0.55955000	0.77202100
C	5.52953500	-0.45387100	-0.52282000
H	5.41581800	0.67873700	-2.35085400
H	5.38460600	-1.36429800	1.41409700
C	3.57142500	0.11806700	2.71746100
H	2.72257300	0.79536200	2.88754000
C	4.66531300	0.47052500	3.74390500
H	5.02411900	1.50496000	3.63067600
H	4.28400100	0.35618500	4.77215600
H	5.53882900	-0.19357100	3.63718400
C	3.05785900	-1.31047800	2.96432000
H	3.86486400	-2.05785900	2.90210600
H	2.61766800	-1.39031600	3.97141100
H	2.28516600	-1.59188900	2.23728400
C	3.64685200	2.60519200	-1.83704400

H	2.90060000	3.22661900	-1.32458200
C	2.97628700	2.05088200	-3.10661300
H	2.10987700	1.42132000	-2.85700000
H	2.62410200	2.87617800	-3.74780600
H	3.67661000	1.44570300	-3.70547600
C	4.82414700	3.53489500	-2.18569500
H	5.28078900	3.96204300	-1.27876600
H	5.61527400	3.00211600	-2.73814600
H	4.48302200	4.36991600	-2.81988700
C	6.61793100	-1.40291400	-1.00932600
H	6.73405200	-2.17756100	-0.23103600
C	6.24403000	-2.11977300	-2.31643600
H	7.01716400	-2.85596500	-2.59132400
H	5.28391200	-2.64974600	-2.22176800
H	6.15124300	-1.41196200	-3.15624200
C	7.97206300	-0.68196200	-1.14069500
H	7.92970400	0.10633000	-1.91046200
H	8.26551800	-0.20596800	-0.19200700
H	8.76756400	-1.38907600	-1.42908400
C	-3.89213100	-0.07583300	-3.03353400
H	-3.24540100	0.81162400	-2.98278400
C	-5.14111600	0.28600600	-3.86219700
H	-4.85319500	0.61073200	-4.87562300
H	-5.80722200	-0.58625000	-3.96797000
H	-5.73045600	1.09411400	-3.40390000
C	-3.09163200	-1.16906800	-3.76232100
H	-3.69858500	-2.07046800	-3.94775400
H	-2.76180600	-0.79740000	-4.74603600
H	-2.19404900	-1.47221600	-3.20911600
C	-4.31113000	0.85792500	2.05495500
H	-3.83746700	1.80179200	1.75388400
C	-5.68423100	1.22772400	2.64610500
H	-6.32240100	1.72234600	1.89697600
H	-6.22539000	0.33947100	3.01068100
H	-5.56551100	1.91629000	3.49904600
C	-3.39890800	0.21722200	3.11595600
H	-3.81945600	-0.72777300	3.49827700
H	-2.39985000	0.01089100	2.70462100
H	-3.27526700	0.89311700	3.97835700
C	-5.99797700	-3.47299800	0.05287600
H	-5.91500400	-4.00144000	-0.91282400
C	-5.35138900	-4.36833600	1.12183800
H	-5.85373500	-5.34847800	1.16328200
H	-4.28508400	-4.54421400	0.91091800

H	-5.42480500	-3.92057900	2.12647200
C	-7.49823800	-3.26990800	0.33486600
H	-7.65549200	-2.74809200	1.29343600
H	-7.97503700	-2.66759900	-0.45408400
H	-8.02271800	-4.23821500	0.38981000

CP II-Pre'

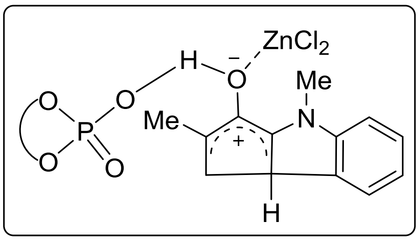

SCF energy[B3LYP-D3BJ/DEF2TZVP/SMD(1,2-dichloroethane)]: -5803.68160419 a.u.

Thermal correction to Gibbs free energy at 298 K: 1.07839400 a.u.

Gibbs free energy at 298 K [B3LYP-D3BJ/DEF2TZVP/SMD(1,2-dichloroethane)]: -5802.60321019 a.u.

C	1.49911200	-4.03556900	2.25509500
C	0.26450500	-3.49063800	2.66762600
C	-0.25245500	-3.68718200	3.94322100
C	0.50246100	-4.47430800	4.82481000
C	1.72573300	-5.02886100	4.43292200
C	2.24021900	-4.80257100	3.14573100
C	1.76490600	-3.49812400	0.87209000
H	-1.20694700	-3.25644700	4.24870200
H	0.12427000	-4.65787800	5.83312200
H	2.28929000	-5.64355600	5.13847500
H	3.20264900	-5.22881500	2.85256800
H	2.48775500	-2.66287400	0.97639200
N	-0.33128600	-2.78510200	1.57929300
C	-1.64607600	-2.15958500	1.63427700
H	-1.61430200	-1.30812900	2.32821800
H	-1.91526000	-1.81276000	0.63382800
H	-2.39131800	-2.89763100	1.95972800
C	0.46372800	-2.86583400	0.53088500
C	0.30072900	-2.72462800	-0.89553300
O	-0.64942800	-2.02426600	-1.49050500
C	1.26731700	-3.53492800	-1.46749800
C	1.45385500	-3.74291200	-2.92908600
H	0.64884500	-3.28153700	-3.51804600
H	2.41659700	-3.30170000	-3.24830900

H	1.50241200	-4.81654800	-3.17934500
C	2.11426900	-4.24928600	-0.43387700
H	3.18661600	-4.22773500	-0.68493300
H	1.81811500	-5.31033700	-0.36197600
Zn	-2.15226900	-2.95221600	-2.45204600
Cl	-3.44952000	-3.92548000	-0.95669600
Cl	-1.89596000	-3.12562300	-4.61764100
P	0.17973700	1.07497600	0.00043900
O	1.31801600	2.00564400	-0.71264500
O	-0.81774300	2.15481000	0.70562500
O	-0.56294900	0.51510200	-1.26825000
O	0.66675900	0.10274100	1.01264100
C	2.14047900	2.85021100	0.02083100
C	3.43505000	2.42067100	0.38748700
C	4.23242800	3.31562400	1.12411500
C	3.75316400	4.55361500	1.55495700
C	2.46809600	4.94330600	1.18784100
C	1.71640800	6.19638800	1.57399200
C	0.27286000	5.85517100	1.16084400
C	0.43108300	4.89715300	-0.06847900
C	1.67672300	4.12439000	0.36193700
C	0.69742900	5.74576600	-1.35591400
C	-0.69585300	6.25402200	-1.77595600
C	-1.60936700	5.15229900	-1.28704100
C	-2.95525600	4.94314000	-1.57521400
C	-3.59880300	3.82791500	-1.03364700
C	-2.91032700	2.86341300	-0.27669900
C	-1.54243400	3.10006900	-0.01259200
C	-0.91286500	4.27212100	-0.43886300
H	-0.57651600	-0.50874800	-1.34473300
H	5.24521200	3.00653100	1.39023400
H	4.37884800	5.20110300	2.17500000
H	2.09715100	7.07641000	1.02523400
H	1.81426000	6.42911300	2.64641900
H	-0.34220900	6.73824100	0.93456100
H	-0.22670500	5.30143800	1.97197600
H	1.42776800	6.55128800	-1.18979000
H	1.10540800	5.08187600	-2.13489500
H	-0.93756200	7.21879900	-1.29517400
H	-0.78323900	6.41578900	-2.86229300
H	-3.50601300	5.63989800	-2.21267600
H	-4.66211100	3.67366600	-1.22554600
C	-3.62763900	1.64280500	0.22168000
C	-4.17727100	0.70487900	-0.69774100

C	-3.80720500	1.43513500	1.61319200
C	-4.85266100	-0.41542000	-0.19967600
C	-4.50637900	0.29958900	2.05363200
C	-5.03505400	-0.64477300	1.16992900
H	-5.24955100	-1.15196800	-0.90213300
H	-4.64476500	0.16207700	3.12759800
C	3.99326500	1.07062800	0.04091300
C	4.29690300	0.73578700	-1.30260200
C	4.31064600	0.15163300	1.07915500
C	4.92640400	-0.48877300	-1.57834100
C	4.93652400	-1.05639900	0.74432200
C	5.27264600	-1.39570300	-0.57317200
H	5.17104200	-0.72215900	-2.61616100
H	5.19664300	-1.75461800	1.54649000
C	4.02136100	0.43138600	2.55702800
H	3.47991500	1.38464700	2.61757100
C	5.32006700	0.58718600	3.37003600
H	5.97611100	1.36670800	2.95189300
H	5.09256100	0.86086900	4.41353500
H	5.89840400	-0.35147500	3.39303400
C	3.10170400	-0.62708200	3.18835000
H	3.56790600	-1.62655600	3.19930700
H	2.88157200	-0.36295700	4.23609400
H	2.15007300	-0.67600300	2.64145700
C	4.01752500	1.67962700	-2.47269200
H	3.54377100	2.58491200	-2.07016000
C	3.03393700	1.06974800	-3.48703100
H	2.08324200	0.79991300	-3.00509300
H	2.81204300	1.79287500	-4.28907400
H	3.44850500	0.16707300	-3.96582700
C	5.32007800	2.13411000	-3.15716200
H	6.00829900	2.60506400	-2.43741800
H	5.85318400	1.29105100	-3.62667900
H	5.10257900	2.86878300	-3.94990000
C	6.06066900	-2.67448700	-0.84484800
H	5.69906600	-3.42917800	-0.12164100
C	5.86697300	-3.25411900	-2.25180500
H	6.37108300	-4.22979300	-2.33785400
H	4.80284500	-3.39974100	-2.49567600
H	6.29830000	-2.59913300	-3.02581900
C	7.55922100	-2.45726000	-0.55421900
H	7.98296900	-1.70576700	-1.24062300
H	7.72223700	-2.09808300	0.47342100
H	8.12640600	-3.39406600	-0.68326200

C	-4.10305200	0.88068800	-2.21750500
H	-3.43328600	1.72424100	-2.43145500
C	-5.48774800	1.21969400	-2.80286500
H	-5.41421000	1.42201200	-3.88403200
H	-6.19242200	0.38301200	-2.66685300
H	-5.93251100	2.10412700	-2.32063700
C	-3.50865000	-0.33685200	-2.94164700
H	-4.10087100	-1.25009600	-2.76431500
H	-3.48133700	-0.18382800	-4.03118400
H	-2.46813900	-0.49424900	-2.62281600
C	-3.32299000	2.43041400	2.66942900
H	-2.78502700	3.23743500	2.15412700
C	-4.50786200	3.09011400	3.39954700
H	-5.19127200	3.58212200	2.68951800
H	-5.09593300	2.35366400	3.97138400
H	-4.15006600	3.85344700	4.11058100
C	-2.33528800	1.79661400	3.66497400
H	-2.81498600	1.00773100	4.26812000
H	-1.47290500	1.36005000	3.14002900
H	-1.95491100	2.55667600	4.36764700
C	-5.82013000	-1.87378800	1.62018000
H	-5.48536900	-2.70240000	0.97149200
C	-5.56299700	-2.29134100	3.07281800
H	-6.06587500	-3.24711500	3.28963700
H	-4.48881900	-2.42377400	3.27836700
H	-5.95221600	-1.55003800	3.79040500
C	-7.32956600	-1.67805400	1.37729000
H	-7.72326200	-0.85407300	1.99596100
H	-7.54039000	-1.43494300	0.32480200
H	-7.89004200	-2.59286700	1.63135400

TS II'

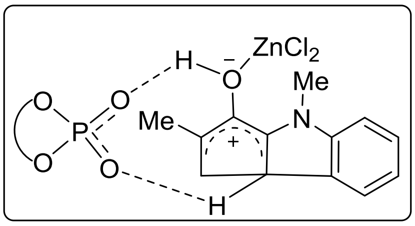

SCF energy[B3LYP-D3BJ/DEF2TZVP/SMD(1,2-dichloroethane)]: -5803.64774096 a.u.

Thermal correction to Gibbs free energy at 298 K: 1.07551600 a.u.

Gibbs free energy at 298 K [B3LYP-D3BJ/DEF2TZVP/SMD(1,2-dichloroethane)]: -5802.57222496 a.u.

C	0.17110800	-2.67593200	3.41284700
C	-1.23993200	-2.51804600	3.50514400
C	-1.91497800	-2.49194800	4.72647400
C	-1.15457500	-2.66852400	5.88527500
C	0.23573900	-2.85703600	5.81604800
C	0.90688700	-2.85319200	4.59006700
C	0.49830600	-2.53565300	1.98685600
H	-2.99554500	-2.34906600	4.78047900
H	-1.65163000	-2.66380200	6.85815600
H	0.80273000	-3.00432000	6.73838900
H	1.98852900	-2.99023500	4.55287700
H	0.69080200	-1.18655700	1.71225200
N	-1.78107500	-2.42516100	2.20312900
C	-3.19924500	-2.30906300	1.89842100
H	-3.55246900	-1.28543100	2.08798900
H	-3.37859800	-2.55778400	0.84568100
H	-3.76332000	-3.01504300	2.52466600
C	-0.76667800	-2.50531100	1.33510200
C	-0.58186100	-2.63697800	-0.09228800
O	-1.45411300	-2.12482600	-1.01681400
C	0.69438200	-3.05894400	-0.32711500
C	1.31863600	-3.24055400	-1.66583600
H	0.58529600	-3.12756800	-2.47625500
H	2.13098400	-2.50794400	-1.81168400
H	1.77866200	-4.23994100	-1.74605900
C	1.45210600	-3.19270500	0.98154400
H	2.44499900	-2.72232600	0.90011600
H	1.61977300	-4.25451000	1.23679900
Zn	-2.69781200	-3.02788200	-2.40504100
Cl	-4.58979400	-3.51766300	-1.42205500
Cl	-1.62401600	-3.41939200	-4.25272500
P	0.46865300	0.77344700	0.08171500
O	1.79526500	1.39646800	-0.64875600
O	-0.34226300	2.08031600	0.64583000
O	-0.31020900	0.08044100	-1.01070300
O	0.88521800	0.01432500	1.35227400
C	2.76386400	2.15489200	-0.00284000
C	4.02191400	1.58128300	0.29636300
C	4.96503700	2.39111100	0.95796900
C	4.66448800	3.68125300	1.39457700
C	3.42376700	4.22347500	1.07462800
C	2.86715000	5.58195000	1.43716000
C	1.39562100	5.47073200	0.99461300

C	1.42618200	4.45980500	-0.19772600
C	2.50390000	3.49736200	0.29613200
C	1.92484700	5.19586000	-1.48829000
C	0.68207000	5.93075900	-2.01859500
C	-0.44185900	5.02454600	-1.57332500
C	-1.77416700	5.04017500	-1.97207100
C	-2.63769200	4.07328900	-1.45945500
C	-2.19654200	3.04066000	-0.61032600
C	-0.84044200	3.05902600	-0.21017200
C	0.01689300	4.07807100	-0.63902200
H	-1.10708100	-1.14299700	-1.11759900
H	5.95303600	1.97349200	1.15942200
H	5.39893100	4.25583600	1.96522900
H	3.39485300	6.38898000	0.89841700
H	2.97688600	5.80077600	2.51161000
H	0.94256400	6.43476700	0.72102200
H	0.78941100	5.03593300	1.80540200
H	2.78209700	5.85631600	-1.29399400
H	2.24951100	4.43671500	-2.21795800
H	0.58472100	6.93744700	-1.57387300
H	0.69776800	6.07130200	-3.11123300
H	-2.14197600	5.79302700	-2.67419400
H	-3.69100800	4.08902800	-1.74425400
C	-3.20708900	2.01198100	-0.19241400
C	-3.83091600	1.19288300	-1.17767400
C	-3.65773900	1.94675100	1.14959700
C	-4.87597900	0.34691100	-0.79012000
C	-4.73106500	1.09869700	1.47344100
C	-5.36599400	0.29659700	0.52146300
H	-5.34450400	-0.29578400	-1.53995700
H	-5.08598400	1.08627400	2.50564100
C	4.41427700	0.17729600	-0.05995400
C	4.50057000	-0.23026800	-1.41920800
C	4.81551800	-0.72652600	0.96266100
C	4.97253300	-1.51648000	-1.71922700
C	5.27637100	-2.00124200	0.60070300
C	5.37532200	-2.42039500	-0.72958800
H	5.05395000	-1.80762900	-2.76928500
H	5.59253400	-2.69206100	1.38821400
C	4.80092800	-0.37176500	2.45337100
H	4.40705200	0.64677800	2.55635600
C	6.21828200	-0.37919300	3.05522800
H	6.90279300	0.28035100	2.49893500
H	6.19514900	-0.03679400	4.10292200

H	6.65889100	-1.38962200	3.04742600
C	3.85736800	-1.27317500	3.26275500
H	4.14900000	-2.33543300	3.20671600
H	3.86726200	-0.98189000	4.32596600
H	2.82603400	-1.17201000	2.90011600
C	4.18064800	0.70397800	-2.58770700
H	3.82943900	1.65730100	-2.17294000
C	3.05338100	0.16227300	-3.48379000
H	2.13273300	-0.00625200	-2.90676500
H	2.82049300	0.88439600	-4.28353600
H	3.33715400	-0.78442100	-3.97267600
C	5.44482900	1.02211900	-3.40866700
H	6.24004500	1.44536300	-2.77459500
H	5.85154800	0.12265800	-3.89920600
H	5.21585700	1.75470700	-4.20020000
C	5.98488500	-3.77659400	-1.06789100
H	5.99445700	-4.36122300	-0.13085300
C	5.17655700	-4.57760900	-2.09905200
H	5.62510700	-5.57230700	-2.25300000
H	4.13548700	-4.72326300	-1.77278800
H	5.15243000	-4.07647200	-3.08026600
C	7.44946700	-3.61821600	-1.51957600
H	7.51381600	-3.04059100	-2.45653300
H	8.04675700	-3.08911800	-0.76083300
H	7.91482800	-4.60186100	-1.69744100
C	-3.46698100	1.23985800	-2.66746600
H	-2.60020200	1.90291200	-2.78796300
C	-4.62412600	1.82525700	-3.50113900
H	-4.31996000	1.94901000	-4.55339600
H	-5.50425700	1.16191100	-3.48505200
H	-4.94733600	2.80760400	-3.12411200
C	-3.04707100	-0.12355900	-3.24281500
H	-3.82029400	-0.89157200	-3.06050400
H	-2.90913100	-0.06852800	-4.33370600
H	-2.08311100	-0.44333900	-2.82631900
C	-3.06265400	2.81101500	2.26255800
H	-2.28212000	3.44212100	1.81760000
C	-4.10757700	3.76611200	2.86835900
H	-4.56923300	4.39852000	2.09359900
H	-4.91735500	3.22001200	3.37915400
H	-3.63739300	4.42975700	3.61268600
C	-2.38484400	1.95893900	3.34991200
H	-3.10925000	1.30453700	3.86321600
H	-1.59041000	1.33065100	2.92143700

H	-1.92551800	2.60425400	4.11705600
C	-6.57688300	-0.58259700	0.82737600
H	-6.38710200	-1.55126700	0.33102200
C	-6.80783100	-0.85777500	2.31774000
H	-7.63629400	-1.57168300	2.44822500
H	-5.91919600	-1.28703100	2.80590800
H	-7.08068000	0.05977400	2.86507200
C	-7.85278300	0.01099400	0.19694200
H	-8.09390000	0.98770100	0.64889200
H	-7.73755200	0.16352400	-0.88664100
H	-8.71429100	-0.65881200	0.35336800

CP II-Post'

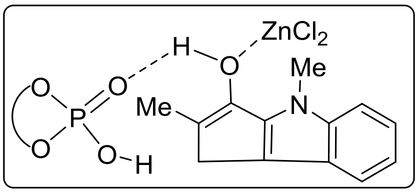

SCF energy[B3LYP-D3BJ/DEF2TZVP/SMD(1,2-dichloroethane)]: -5803.66830134 a.u.
Thermal correction to Gibbs free energy at 298 K: 1.07493600 a.u.

Gibbs free energy at 298 K [B3LYP-D3BJ/DEF2TZVP/SMD(1,2-dichloroethane)]: -5802.59336534 a.u.

C	0.19641300	-4.98355900	1.98450000
C	-1.11764900	-4.59862500	2.42575300
C	-1.69027800	-5.14028000	3.58420300
C	-0.95102200	-6.07827300	4.30331500
C	0.33707800	-6.47265800	3.88282200
C	0.91369500	-5.93610200	2.73577500
C	0.43586700	-4.22785400	0.79557100
H	-2.68449400	-4.83879600	3.92056000
H	-1.37774400	-6.51472400	5.20988200
H	0.88803200	-7.21182900	4.46999300
H	1.91166100	-6.25010400	2.41895600
H	0.91129700	-0.83981100	0.77769000
N	-1.64225000	-3.66383000	1.53906000
C	-2.92814400	-3.00554900	1.66189600
H	-2.88244400	-2.14629800	2.35321900
H	-3.26598200	-2.65751100	0.67829800
H	-3.67569800	-3.71988100	2.03624700
C	-0.68520700	-3.45180400	0.57564200
C	-0.47736800	-2.62509700	-0.60987700

O	-1.42613200	-1.74896700	-1.10893000
C	0.75552900	-2.88372700	-1.13824600
C	1.34752300	-2.31891700	-2.38854900
H	0.64562200	-1.65402400	-2.90703800
H	2.27977500	-1.76529700	-2.18478900
H	1.60448100	-3.12858100	-3.09326900
C	1.44109200	-3.94517100	-0.28441400
H	2.41255200	-3.58343000	0.09758200
H	1.66745000	-4.83161300	-0.90560400
Zn	-2.73488500	-2.33718900	-2.64062500
Cl	-4.64902600	-2.92310600	-1.77890500
Cl	-1.66971700	-2.37058300	-4.53100100
P	0.45190300	1.11307200	-0.01774400
O	1.82677800	1.81297500	-0.49420900
O	-0.33499900	2.20651700	0.86386300
O	-0.25390900	0.55170400	-1.20765000
O	0.80034200	0.06214300	1.13403800
C	2.73706300	2.37716500	0.41011500
C	3.89862800	1.65382800	0.75547000
C	4.77656900	2.25309000	1.67682700
C	4.49394700	3.47592600	2.28672400
C	3.34121000	4.16340600	1.91897100
C	2.81536300	5.47573900	2.45255900
C	1.37808500	5.50590300	1.90263800
C	1.45465000	4.71330600	0.55370700
C	2.48374900	3.64909300	0.92862300
C	1.99016900	5.65344100	-0.57659100
C	0.76124800	6.48006700	-1.00474800
C	-0.38475500	5.53109000	-0.73523200
C	-1.71663600	5.63070400	-1.12854300
C	-2.60509900	4.61077500	-0.78214400
C	-2.18584900	3.44050800	-0.12125000
C	-0.82647300	3.37085100	0.24723000
C	0.05370200	4.42960600	0.02087900
H	-1.02474500	-0.82619500	-1.19828900
H	5.69685700	1.72486500	1.93013200
H	5.17401600	3.88602200	3.03784900
H	3.41225100	6.32542200	2.07573500
H	2.85664000	5.52688600	3.55216500
H	0.97592100	6.52103700	1.77324900
H	0.70433300	4.96703100	2.58799700
H	2.84132300	6.26741200	-0.24793000
H	2.33470800	5.03059300	-1.41764600
H	0.66500700	7.40352800	-0.40648700

H	0.80316700	6.79402400	-2.05984400
H	-2.06926400	6.49587900	-1.69607400
H	-3.65810700	4.69765100	-1.05515300
C	-3.18264100	2.35760500	0.17102300
C	-3.84356000	1.68504000	-0.89548300
C	-3.53863000	2.05697700	1.51078200
C	-4.82678800	0.73733400	-0.58949100
C	-4.54198100	1.10576800	1.75457900
C	-5.20730600	0.43627800	0.72469400
H	-5.32398300	0.20499200	-1.40403700
H	-4.81752500	0.89828900	2.78995800
C	4.26568300	0.31835500	0.17244400
C	4.64753800	0.22091600	-1.19303000
C	4.35672300	-0.82800300	1.00862200
C	5.12148400	-1.00421900	-1.68207200
C	4.84476400	-2.02497300	0.46237600
C	5.24291300	-2.13948100	-0.87318100
H	5.42740400	-1.06400900	-2.72936800
H	4.93346900	-2.90298000	1.10819100
C	3.97910700	-0.81237300	2.49394800
H	3.45833000	0.13443400	2.69685800
C	5.22774700	-0.86827500	3.39594300
H	5.92963100	-0.04799600	3.18577300
H	4.94144200	-0.80422400	4.45845500
H	5.77514000	-1.81453200	3.25428600
C	3.01484000	-1.94652300	2.88500800
H	3.48517200	-2.93932600	2.80518500
H	2.69103400	-1.82626700	3.93100200
H	2.10841500	-1.96229700	2.26354300
C	4.62056300	1.41493400	-2.14909300
H	4.25228500	2.28696800	-1.59213600
C	3.65559500	1.19237000	-3.32684900
H	2.63545500	0.98498100	-2.97216300
H	3.61612000	2.08949300	-3.96654500
H	3.97458500	0.35029800	-3.96289500
C	6.03395100	1.77707400	-2.64175200
H	6.71579400	1.97066400	-1.79859200
H	6.47509000	0.97059900	-3.24975800
H	6.00282800	2.68323600	-3.26879300
C	5.83466100	-3.44256500	-1.39783300
H	5.65784700	-4.20571000	-0.61968200
C	5.16034500	-3.93761800	-2.68708500
H	5.56115100	-4.92148300	-2.97985100
H	4.07155000	-4.03827000	-2.56089500

H	5.33615000	-3.24975500	-3.53025100
C	7.35913900	-3.32294300	-1.58129500
H	7.60843200	-2.57549400	-2.35258700
H	7.85250600	-3.01480000	-0.64629600
H	7.79315700	-4.28651900	-1.89515600
C	-3.56086800	1.97266600	-2.37403900
H	-2.71178200	2.66731600	-2.43215600
C	-4.76602100	2.65403100	-3.05020200
H	-4.52625600	2.92383100	-4.09175700
H	-5.64188000	1.98554400	-3.07191600
H	-5.06773300	3.57213700	-2.52222400
C	-3.14590100	0.71913200	-3.16128400
H	-3.93029500	-0.05657800	-3.13447700
H	-2.97243500	0.95487400	-4.22290200
H	-2.20070000	0.31464600	-2.77461300
C	-2.92621200	2.77314700	2.71602500
H	-2.15835500	3.46707000	2.34767400
C	-3.97240400	3.63103300	3.45186000
H	-4.44584000	4.35631900	2.77146200
H	-4.77316900	3.01188600	3.88775900
H	-3.50266400	4.19379800	4.27566200
C	-2.22506300	1.79881400	3.67909300
H	-2.93756000	1.09158200	4.13468300
H	-1.45006500	1.21583000	3.15995600
H	-1.74116000	2.35082100	4.50194100
C	-6.34526600	-0.55132400	0.96328700
H	-6.19759900	-1.37012700	0.23816400
C	-6.36411900	-1.17114400	2.36539400
H	-7.13334000	-1.95746700	2.42237900
H	-5.39709400	-1.62818400	2.62632500
H	-6.60532000	-0.42681500	3.14253400
C	-7.70517600	0.10309900	0.64784100
H	-7.90727600	0.94021400	1.33706300
H	-7.73505000	0.50060600	-0.37813400
H	-8.52407500	-0.62779500	0.75076100

Enol

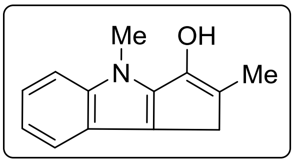

SCF energy[B3LYP-D3BJ/DEF2TZVP/SMD(1,2-dichloroethane)]: -633.480678032 a.u.

Thermal correction to Gibbs free energy at 298 K: 0.19016100 a.u.

Gibbs free energy at 298 K [B3LYP-D3BJ/DEF2TZVP/SMD(1,2-dichloroethane)]:
-633.29051703 a.u.

C	3.52787200	-1.38395200	0.00016200
C	3.85386000	-0.01142800	0.00026700
C	2.85818300	0.96571800	0.00018400
C	1.52334400	0.54309300	0.00009600
C	1.16881500	-0.85206400	-0.00014700
C	2.20224200	-1.80982900	-0.00007300
N	0.36157700	1.30672800	0.00005200
C	-0.69336000	0.42337300	-0.00043600
C	-0.26096200	-0.88463900	-0.00058500
C	0.29647100	2.75218900	-0.00025500
C	-2.14874000	0.47208400	0.00001200
C	-2.63153100	-0.80476200	0.00013800
C	-1.46410900	-1.78746700	-0.00014300
C	-4.06537300	-1.23151000	0.00023600
H	4.33246900	-2.12389400	0.00021800
H	4.90369900	0.29258700	0.00037900
H	3.11698900	2.02710600	0.00016800
H	1.96452000	-2.87679900	-0.00023100
H	-0.75625600	3.06002000	0.00283500
H	0.79103800	3.17259700	0.89187200
H	0.78566500	3.17236400	-0.89550300
H	-1.50247000	-2.45137500	-0.88429100
H	-1.50180100	-2.45095500	0.88436900
H	-4.75883100	-0.37254300	-0.00045100
H	-4.31687000	-1.84312500	-0.88545300
H	-4.31721300	-1.84197800	0.88663200
O	-2.81329700	1.65672200	0.00022600
H	-3.76586700	1.49029000	0.00054200

CP III-R-Pre

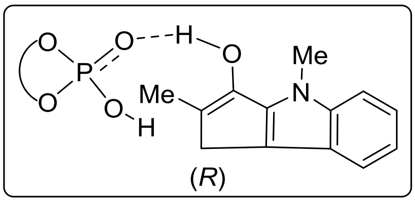

SCF energy[B3LYP-D3BJ/DEF2TZVP/SMD(1,2-dichloroethane)]: -3103.59363952 a.u.
Thermal correction to Gibbs free energy at 298 K: 1.08041200 a.u.

Gibbs free energy at 298 K [B3LYP-D3BJ/DEF2TZVP/SMD(1,2-dichloroethane)]:

-3102.51322752 a.u.

C	-0.54211300	7.74096900	2.03132100
C	0.84345900	7.66619700	2.28795200
C	1.62329100	6.64437800	1.74690100
C	0.98899200	5.69047800	0.94060000
C	-0.42274700	5.74976300	0.66498200
C	-1.17750200	6.79746600	1.22876500
N	1.53172800	4.58653000	0.29582200
C	0.49963300	3.97038600	-0.37351000
C	-0.69344100	4.63318100	-0.18659700
C	2.91724100	4.17119100	0.34145500
C	0.30474600	2.78573200	-1.20408800
C	-1.03180200	2.69650800	-1.52289900
C	-1.77472300	3.88733500	-0.91509600
C	-1.67591900	1.78949800	-2.52956900
H	-1.12349100	8.55558900	2.47100100
H	1.31456200	8.42192600	2.92169200
H	2.69546900	6.59239100	1.94962600
H	-2.25127300	6.86822000	1.03634600
H	3.03111500	3.24576000	-0.23630300
H	3.57892700	4.94366900	-0.08612300
H	3.23485800	3.98042100	1.38033700
H	-2.59954500	3.56412900	-0.25558200
H	-2.24158600	4.48841500	-1.71795000
H	-2.06113200	2.37485200	-3.38501700
H	-0.97396500	1.04274900	-2.92369800
H	-2.53902300	1.24363800	-2.11170900
O	1.34096800	2.00132000	-1.55342600
H	1.04262200	1.09225700	-1.79235400
P	-0.05437300	-0.91319500	-0.31254500
O	1.03019800	-1.60838400	0.67078900
O	-1.28649000	-1.96315400	-0.40804800
O	-0.53106600	0.29780700	0.60429500
O	0.42105800	-0.56455400	-1.68026200
C	1.69670700	-2.75111100	0.20729400
C	2.98253200	-2.62922300	-0.36107100
C	3.57482400	-3.80580100	-0.85550100
C	2.90678100	-5.03216600	-0.86308300
C	1.64350700	-5.11672900	-0.28491200
C	0.71817300	-6.30918800	-0.18998700
C	-0.60834900	-5.66876400	0.26407200
C	-0.17448500	-4.42172700	1.10360900
C	1.05972600	-3.98856800	0.31800000

C	0.22130300	-4.87544200	2.54925700
C	-1.11872600	-5.00617500	3.29635800
C	-1.97782500	-3.96930200	2.60965100
C	-3.20894700	-3.45675500	3.00841400
C	-3.81634000	-2.47210800	2.22780200
C	-3.19430900	-1.92635300	1.08993200
C	-1.94614600	-2.46932100	0.71430200
C	-1.36729100	-3.52491200	1.42305400
H	-0.71247500	1.11568100	0.07617500
H	4.58408700	-3.74093800	-1.26412600
H	3.37775400	-5.91114900	-1.31112400
H	1.08758300	-7.04370300	0.54773300
H	0.62902300	-6.84585600	-1.14797700
H	-1.25857100	-6.35247300	0.82917700
H	-1.17507300	-5.31899900	-0.61367000
H	0.82134100	-5.79697900	2.55244100
H	0.82982700	-4.08140200	3.01089800
H	-1.54948800	-6.01735600	3.18440400
H	-1.02682700	-4.82339000	4.37887300
H	-3.69849400	-3.81839700	3.91645200
H	-4.79604500	-2.08652200	2.51411500
C	-3.89142100	-0.83101800	0.33474100
C	-4.13825200	0.42211400	0.95926500
C	-4.40261100	-1.07510300	-0.96845400
C	-4.88823400	1.38574100	0.26802200
C	-5.14390500	-0.07303200	-1.60875500
C	-5.40827900	1.16408300	-1.01040800
H	-5.08802400	2.34620400	0.75114900
H	-5.54310900	-0.27700500	-2.60556800
C	3.76043500	-1.34403200	-0.41439800
C	4.27601500	-0.78351300	0.78071900
C	4.07928500	-0.74233600	-1.66464100
C	5.11729100	0.33822500	0.70498700
C	4.92092300	0.37554900	-1.67642000
C	5.46964000	0.92680200	-0.51130700
H	5.51435700	0.74932500	1.63462500
H	5.16523700	0.83970200	-2.63567900
C	3.55666500	-1.27680700	-3.00209700
H	2.71961400	-1.95512000	-2.78209300
C	4.63813800	-2.07299500	-3.75940900
H	5.02368200	-2.92085400	-3.17544200
H	4.23411500	-2.47260500	-4.70435400
H	5.49639100	-1.42733000	-4.00938500
C	3.00612900	-0.17249100	-3.92256900

H	3.81046700	0.46366400	-4.32669700
H	2.48928000	-0.62539800	-4.78395400
H	2.29288700	0.48005900	-3.40496100
C	3.98782500	-1.36788000	2.16473200
H	3.35102900	-2.25354300	2.03443900
C	3.20829400	-0.37912500	3.05037000
H	2.26982100	-0.07080900	2.56675300
H	2.95708700	-0.84193600	4.01937100
H	3.79865600	0.52775900	3.26184900
C	5.27240900	-1.85282200	2.86131600
H	5.81126000	-2.58774000	2.24253700
H	5.96488600	-1.02092600	3.06969600
H	5.03255000	-2.33133200	3.82535400
C	6.44586400	2.09582200	-0.62278200
H	6.02830500	2.78099500	-1.38321400
C	6.62534500	2.89906800	0.67056600
H	7.24839500	3.78833500	0.48498600
H	5.66254700	3.23999200	1.08090300
H	7.13144700	2.30683800	1.45056800
C	7.81285300	1.61405500	-1.14823800
H	8.28179400	0.91910400	-0.43191700
H	7.71415300	1.08543000	-2.10855000
H	8.49908100	2.46425100	-1.29636500
C	-3.66077000	0.76028700	2.37561100
H	-2.97952000	-0.03847700	2.69993400
C	-4.83976600	0.80340400	3.36799800
H	-4.47741900	0.98234200	4.39369100
H	-5.54056900	1.61609700	3.11589400
H	-5.41441300	-0.13518700	3.37207900
C	-2.86875400	2.07684400	2.45328300
H	-3.48966100	2.95127700	2.20053000
H	-2.49373200	2.23273400	3.47762600
H	-1.99922800	2.07111300	1.78279500
C	-4.23258600	-2.41436900	-1.68829200
H	-3.64129500	-3.07565800	-1.04072400
C	-5.58829000	-3.11252300	-1.90488700
H	-6.12584100	-3.25017100	-0.95329700
H	-6.24292200	-2.53400100	-2.57697300
H	-5.44304500	-4.10648600	-2.35932100
C	-3.45908100	-2.26957500	-3.01057900
H	-4.00402400	-1.64176700	-3.73471300
H	-2.46926100	-1.82060800	-2.84345200
H	-3.30975500	-3.25668500	-3.47881200
C	-6.26368400	2.22097000	-1.69935200

H	-6.23461900	3.11923500	-1.05798300
C	-5.71569800	2.62499500	-3.07786500
H	-6.31882900	3.43904400	-3.51200400
H	-4.67330100	2.97205100	-3.01026000
H	-5.74114900	1.78218400	-3.78806000
C	-7.73442400	1.77625200	-1.79582900
H	-7.83952300	0.88145500	-2.43144400
H	-8.14453200	1.53055800	-0.80378400
H	-8.35693200	2.57314700	-2.23518300

CP III-S-Pre

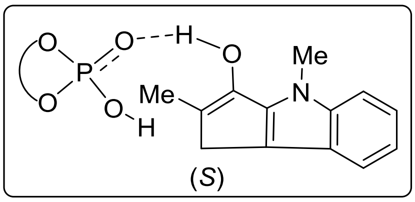

SCF energy[B3LYP-D3BJ/DEF2TZVP/SMD(1,2-dichloroethane)]: -3103.59416008 a.u.
Thermal correction to Gibbs free energy at 298 K: 1.08028400 a.u.

Gibbs free energy at 298 K [B3LYP-D3BJ/DEF2TZVP/SMD(1,2-dichloroethane)]: -3102.51387608 a.u.

C	6.31888500	4.88264200	-1.82445600
C	6.92115600	4.53752200	-0.59564000
C	6.17800300	3.96857600	0.43820400
C	4.81201400	3.74463400	0.21941700
C	4.17707900	4.09601900	-1.02442100
C	4.96124200	4.66903400	-2.04552900
N	3.86610300	3.19479800	1.07339800
C	2.66714100	3.20770900	0.39814700
C	2.79960500	3.74755300	-0.86230300
C	4.09980100	2.74642700	2.43001200
C	1.29538200	2.77575700	0.63467800
C	0.55725000	3.03814200	-0.50392400
C	1.46360700	3.70129300	-1.54600800
C	-0.93954700	3.09110100	-0.62702000
H	6.93131700	5.32754400	-2.61297800
H	7.98886900	4.72072300	-0.45030800
H	6.64998800	3.70819000	1.38824800
H	4.50605000	4.94469800	-3.00033000
H	3.17061100	2.31609000	2.82231500
H	4.88784300	1.97690600	2.45793400
H	4.40830000	3.58345100	3.07963500

H	1.07758000	4.70737100	-1.79558800
H	1.48585000	3.13938100	-2.49822100
H	-1.45159300	2.55895200	0.18586900
H	-1.29230900	4.13866300	-0.59715600
H	-1.30099900	2.66905400	-1.57965000
O	0.93092600	2.25362100	1.81543100
H	0.18599800	1.60701000	1.72296700
P	-0.71181500	-0.53016900	0.14394400
O	-2.11851200	-0.80018400	-0.62144600
O	-0.11370000	-1.99538200	0.47633400
O	0.16580400	0.06904600	-1.03589700
O	-0.80678100	0.27688200	1.39223100
C	-3.16668800	-1.40619700	0.07908000
C	-4.18240800	-0.60925600	0.64801900
C	-5.17880300	-1.27439000	1.38545700
C	-5.14769400	-2.65345200	1.60519400
C	-4.14241000	-3.41056200	1.00997400
C	-3.88697100	-4.89752900	1.10834800
C	-2.47183000	-5.03415500	0.51360300
C	-2.36637600	-3.87099600	-0.52968700
C	-3.17987000	-2.79783600	0.18847700
C	-3.05599600	-4.30028300	-1.86766500
C	-2.00042300	-5.15832500	-2.59181100
C	-0.69917900	-4.56455100	-2.10088300
C	0.59238700	-4.77525900	-2.57648100
C	1.65741400	-4.11051600	-1.96461100
C	1.46292900	-3.16812900	-0.93835900
C	0.13674900	-2.95565300	-0.50606000
C	-0.92845800	-3.70167100	-1.01445700
H	0.46584800	1.00371800	-0.86233400
H	-5.98927000	-0.67988800	1.81016800
H	-5.90992900	-3.12717100	2.22945400
H	-4.62988300	-5.46862700	0.52325500
H	-3.95388000	-5.26620600	2.14433700
H	-2.27282200	-6.02008800	0.06868000
H	-1.71912300	-4.86676600	1.30067200
H	-4.01018500	-4.82163500	-1.70216400
H	-3.26962300	-3.39401500	-2.45678100
H	-2.08228700	-6.22378000	-2.31178800
H	-2.09252100	-5.11540400	-3.68887200
H	0.77769900	-5.46026100	-3.40808500
H	2.67803700	-4.30734700	-2.29677900
C	2.65497600	-2.49125500	-0.32355900
C	3.46883600	-1.61898500	-1.09758300

C	3.03545000	-2.79996700	1.00771900
C	4.64013200	-1.10456600	-0.52837700
C	4.22100800	-2.25458300	1.52406200
C	5.04825900	-1.41520100	0.77423000
H	5.26490900	-0.43177400	-1.12234900
H	4.50551400	-2.51063900	2.54616000
C	-4.28364300	0.87769000	0.45197000
C	-4.65267000	1.39346600	-0.81860900
C	-4.11627600	1.76645700	1.54713800
C	-4.85951500	2.77233100	-0.96077000
C	-4.34231900	3.13582100	1.34484900
C	-4.72133200	3.66466700	0.10754100
H	-5.15361600	3.15716400	-1.94036600
H	-4.21956700	3.82144700	2.18729900
C	-3.71676100	1.29236500	2.94666300
H	-3.34267000	0.26283000	2.85245100
C	-4.92340200	1.27915900	3.90576400
H	-5.74543800	0.64970900	3.53459300
H	-4.62725500	0.89584000	4.89626700
H	-5.32495000	2.29636200	4.04766200
C	-2.57760700	2.12363400	3.56319800
H	-2.90033400	3.14876900	3.80812500
H	-2.24162200	1.65831100	4.50401800
H	-1.70955300	2.18868000	2.89513900
C	-4.87374000	0.50239800	-2.04286800
H	-4.71350100	-0.54066000	-1.73701000
C	-3.86003100	0.80268300	-3.16155800
H	-2.82803700	0.68268600	-2.80013700
H	-4.00496500	0.11413700	-4.01076200
H	-3.97208300	1.82953700	-3.54721700
C	-6.32144300	0.58834900	-2.55952200
H	-7.04496200	0.33993300	-1.76702600
H	-6.56596000	1.59726300	-2.92983800
H	-6.47667700	-0.11564900	-3.39379700
C	-5.01206800	5.15329600	-0.04462500
H	-4.65628800	5.64106800	0.87975300
C	-4.26114300	5.79866100	-1.22002000
H	-4.43756700	6.88645100	-1.24228900
H	-3.17541200	5.63205200	-1.14391000
H	-4.59512000	5.39361100	-2.18941000
C	-6.52632300	5.41536100	-0.14522500
H	-6.94954400	4.95120700	-1.05145200
H	-7.06259500	5.00049700	0.72251700
H	-6.73633400	6.49682500	-0.19208700

C	3.12440400	-1.21835200	-2.53437000
H	2.09606300	-1.55071900	-2.73569600
C	4.05104700	-1.90258200	-3.55779300
H	3.76296100	-1.62875700	-4.58633700
H	5.09894900	-1.59279500	-3.41157100
H	4.01838400	-2.99979000	-3.48027700
C	3.15145500	0.30584300	-2.74267400
H	4.17212600	0.71534000	-2.68437000
H	2.75449000	0.56047200	-3.73907400
H	2.54495300	0.82567000	-1.98903500
C	2.23510000	-3.74445400	1.90641900
H	1.35837500	-4.09033700	1.34265100
C	3.04372600	-5.00352500	2.27005300
H	3.39100100	-5.53124300	1.36762700
H	3.93168200	-4.75919600	2.87588900
H	2.42645700	-5.70399500	2.85672400
C	1.70873600	-3.03309800	3.16591200
H	2.53284300	-2.69316600	3.81473000
H	1.09813800	-2.15732800	2.90181800
H	1.08268900	-3.71783800	3.76215600
C	6.37834200	-0.87837200	1.29482800
H	6.49081400	0.13846800	0.87699100
C	6.45693700	-0.76123100	2.82212600
H	7.38589600	-0.24927600	3.12002200
H	5.60781100	-0.19388100	3.23445100
H	6.46410000	-1.75027300	3.30880800
C	7.55144400	-1.72072500	0.75576300
H	7.49316700	-2.75593600	1.13134400
H	7.54318100	-1.76465100	-0.34411600
H	8.51917300	-1.29749500	1.07241000

TS III-R

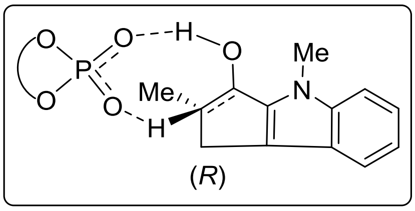

SCF energy[B3LYP-D3BJ/DEF2TZVP/SMD(1,2-dichloroethane)]: -3103.58294325 a.u.
Thermal correction to Gibbs free energy at 298 K: 1.07861800 a.u.

Gibbs free energy at 298 K [B3LYP-D3BJ/DEF2TZVP/SMD(1,2-dichloroethane)]: -3102.50432525 a.u.

C	3.85836200	6.81611500	1.85367300
C	5.07030400	6.34012800	1.29549400
C	5.09684400	5.26190000	0.41979800
C	3.87313200	4.64448000	0.10252400
C	2.62889000	5.12267900	0.64730300
C	2.64619300	6.22096400	1.53775700
N	3.63327600	3.56510000	-0.72279800
C	2.26741500	3.37332600	-0.73901200
C	1.61657600	4.29487000	0.07747900
C	4.61266900	2.85438100	-1.52140700
C	1.33988200	2.44849000	-1.29828400
C	-0.00332100	2.75624600	-0.76558400
C	0.14091200	4.04445400	0.07685400
C	-1.20456200	2.62441700	-1.70057100
H	3.88463700	7.66572800	2.53972500
H	6.00850200	6.83439000	1.56030700
H	6.03815200	4.90749000	-0.00405200
H	1.71377300	6.59495500	1.96720300
H	4.16275500	1.92745700	-1.89694900
H	4.95002700	3.46381900	-2.37711500
H	5.48600000	2.59223500	-0.90691900
H	-0.26740000	3.91419600	1.09215000
H	-0.41585900	4.88227300	-0.37865200
H	-1.27684500	3.46972400	-2.40562400
H	-1.15001200	1.69309100	-2.28048800
H	-2.12811500	2.58564500	-1.10787200
O	1.63567100	1.45334100	-2.05196800
H	0.90805000	0.63629200	-2.00009800
P	-0.52730700	-0.66556500	-0.33972200
O	0.20848400	-1.86406500	0.51891200
O	-2.06052100	-1.23204500	-0.56452600
O	-0.51779400	0.50560400	0.60118700
O	0.06403900	-0.50134100	-1.74129300
C	0.47636900	-3.09464100	-0.03728200
C	1.78615500	-3.39824100	-0.47378100
C	1.98431500	-4.64627500	-1.09070300
C	0.93319400	-5.53096000	-1.34083100
C	-0.33847000	-5.21492400	-0.87126300
C	-1.62720800	-5.99410800	-1.01343900
C	-2.68537200	-4.99415300	-0.50389100
C	-1.91849800	-4.11319200	0.53638200
C	-0.55282100	-4.03445600	-0.14107000
C	-1.79327000	-4.89659700	1.88832200

C	-3.14328800	-4.67765200	2.59232400
C	-3.55149700	-3.31206600	2.08998600
C	-4.55942600	-2.48167800	2.57044900
C	-4.74826400	-1.23844400	1.96683900
C	-3.91095600	-0.76921300	0.93749400
C	-2.89072400	-1.63156600	0.46889700
C	-2.75442400	-2.92133100	0.99821900
H	-0.13897600	1.89800100	-0.04113900
H	2.99404700	-4.91569300	-1.40239500
H	1.11642700	-6.46260700	-1.88291200
H	-1.61013500	-6.91416600	-0.40216700
H	-1.80641100	-6.31399300	-2.05275000
H	-3.57674000	-5.47709700	-0.07669700
H	-3.02010300	-4.34662800	-1.32995800
H	-1.53712500	-5.95603600	1.74191700
H	-0.98514500	-4.43871100	2.48110300
H	-3.88149300	-5.44684300	2.30159400
H	-3.06791000	-4.71537900	3.69106300
H	-5.19593100	-2.79490500	3.40236300
H	-5.55410500	-0.58814000	2.31312800
C	-4.16813000	0.60364700	0.38720000
C	-4.00513800	1.74320000	1.21960600
C	-4.68587900	0.76603000	-0.92516800
C	-4.36135000	3.00448600	0.71868500
C	-5.02707200	2.05050500	-1.37042900
C	-4.87986900	3.18645600	-0.56677000
H	-4.24042700	3.88193200	1.36094200
H	-5.43657400	2.16156800	-2.37770600
C	2.96189800	-2.48631600	-0.26613900
C	3.38593400	-2.15667600	1.05046400
C	3.72888200	-2.02943600	-1.37519100
C	4.55015600	-1.39571100	1.22628200
C	4.88762800	-1.27704700	-1.13518500
C	5.32509500	-0.95027400	0.15234400
H	4.86719200	-1.16358600	2.24525900
H	5.48165500	-0.93747000	-1.98899400
C	3.36819900	-2.34126100	-2.83190500
H	2.35777000	-2.77258500	-2.84029200
C	4.33514600	-3.37151800	-3.44813700
H	4.38541600	-4.30205900	-2.86342900
H	4.02240500	-3.63304100	-4.47267300
H	5.35930600	-2.96671000	-3.50587300
C	3.31877700	-1.08710800	-3.72164600
H	4.31669500	-0.63633700	-3.85497700

H	2.94692000	-1.35056600	-4.72533800
H	2.65363000	-0.32211000	-3.30374500
C	2.66828300	-2.64410200	2.31118900
H	1.78927100	-3.22269100	2.00153900
C	2.15731000	-1.47672900	3.17396900
H	1.49162100	-0.82078700	2.59444400
H	1.58997800	-1.85982300	4.03863600
H	2.98822900	-0.86962100	3.57076700
C	3.55578900	-3.60156200	3.12898200
H	3.88684400	-4.45953100	2.52234500
H	4.45758800	-3.09730900	3.51341100
H	3.00117300	-3.99383800	3.99757900
C	6.63527900	-0.19646200	0.35193900
H	6.83668000	0.34179900	-0.59226700
C	6.58172200	0.84813300	1.47676600
H	7.50788300	1.44573400	1.49550400
H	5.73021600	1.53643800	1.35612500
H	6.48416200	0.37620200	2.46763900
C	7.80408600	-1.17635400	0.56956100
H	7.65962200	-1.75678700	1.49568500
H	7.88386100	-1.89282600	-0.26251600
H	8.76335700	-0.63832600	0.65249700
C	-3.48664000	1.65637800	2.65833100
H	-3.18475500	0.61678200	2.84277600
C	-4.58716000	2.00963600	3.67689200
H	-4.22029200	1.87162600	4.70754200
H	-4.90739000	3.06002300	3.57637800
H	-5.48173700	1.38033700	3.55036300
C	-2.23355500	2.51611100	2.89433700
H	-2.43213700	3.58951100	2.73558100
H	-1.88355300	2.39841200	3.93332600
H	-1.42342100	2.19365000	2.22541100
C	-4.94614100	-0.41633900	-1.86033600
H	-4.65031800	-1.33314100	-1.33318800
C	-6.44484000	-0.56039800	-2.18463900
H	-7.04729100	-0.64782000	-1.26654400
H	-6.82696200	0.30294000	-2.75402600
H	-6.62252400	-1.46188700	-2.79429700
C	-4.09995600	-0.34368300	-3.14339300
H	-4.34307100	0.54959200	-3.74291900
H	-3.02643900	-0.32331000	-2.90628200
H	-4.28852000	-1.22614800	-3.77728800
C	-5.30475000	4.56977000	-1.04552000
H	-4.95348600	5.28941600	-0.28501300

C	-4.65866900	4.96602200	-2.38292800
H	-4.92750400	6.00019200	-2.65464400
H	-3.56057700	4.90084100	-2.33392700
H	-4.99462900	4.31340300	-3.20529500
C	-6.83819200	4.69165600	-1.11378500
H	-7.25907300	3.99805200	-1.86047500
H	-7.30070800	4.45531400	-0.14278700
H	-7.14049400	5.71360100	-1.39739400

TS III-S

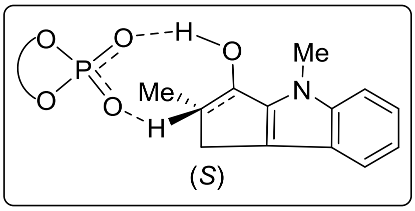

SCF energy[B3LYP-D3BJ/DEF2TZVP/SMD(1,2-dichloroethane)]: -3103.58656328 a.u.
Thermal correction to Gibbs free energy at 298 K: 1.07913500 a.u.

Gibbs free energy at 298 K [B3LYP-D3BJ/DEF2TZVP/SMD(1,2-dichloroethane)]: -3102.50742828 a.u.

C	-6.51516000	4.48904600	1.55071000
C	-7.00670200	4.33186700	0.23444100
C	-6.18762800	3.88845100	-0.80025000
C	-4.84943700	3.59367900	-0.49527400
C	-4.32792100	3.75746100	0.83650300
C	-5.18949000	4.20863000	1.85786800
N	-3.84538900	3.13399200	-1.32904800
C	-2.70913400	3.02778400	-0.55535100
C	-2.94656600	3.40449200	0.75403100
C	-3.95440200	2.90101800	-2.75539400
C	-1.35592100	2.56345200	-0.70667400
C	-0.70014900	2.61585900	0.55502700
C	-1.69546200	3.23378700	1.56035800
C	0.76839600	2.99880700	0.67368000
H	-7.19133500	4.83910700	2.33439600
H	-8.05383300	4.56420500	0.02420800
H	-6.57767500	3.77244400	-1.81350000
H	-4.81548600	4.33709800	2.87661400
H	-3.01029700	2.47501200	-3.11556600
H	-4.76878700	2.19253400	-2.97332300
H	-4.15309700	3.84041700	-3.29886900

H	-1.31060700	4.20020700	1.93368400
H	-1.84979200	2.59938700	2.45041200
H	1.38984500	2.51705600	-0.09277100
H	0.90166900	4.09084800	0.57062600
H	1.17705900	2.70598100	1.65276300
O	-0.89300800	2.12498800	-1.85321800
H	-0.11253000	1.47684600	-1.73257400
P	0.70393600	-0.51538300	-0.17071400
O	2.14072700	-0.81166300	0.55107900
O	0.15559100	-1.98924000	-0.59799900
O	-0.18108500	0.05755200	0.96427600
O	0.83866500	0.30305700	-1.42633500
C	3.20045400	-1.31049300	-0.20240100
C	4.19439700	-0.42910700	-0.68215800
C	5.21238700	-0.97887800	-1.48194300
C	5.22043800	-2.32427100	-1.85613500
C	4.23636600	-3.16980100	-1.35209300
C	4.02034200	-4.64151400	-1.62438900
C	2.61855700	-4.88888600	-1.03413700
C	2.49999100	-3.86007300	0.13937400
C	3.26146500	-2.68199500	-0.46359400
C	3.24728800	-4.41580200	1.39832800
C	2.24840100	-5.39604900	2.04162500
C	0.91322600	-4.79318600	1.66810300
C	-0.35061000	-5.09902000	2.16538800
C	-1.45384200	-4.38670400	1.69173100
C	-1.32380000	-3.32006300	0.78335000
C	-0.02740000	-3.03210800	0.30216900
C	1.07446400	-3.80307100	0.68247700
H	-0.52887400	1.17674200	0.79026900
H	6.00480900	-0.31737800	-1.83542400
H	5.99461600	-2.70344500	-2.52858300
H	4.78671900	-5.25818700	-1.12153800
H	4.08205900	-4.88081700	-2.69817300
H	2.45440700	-5.92577600	-0.70599000
H	1.84870000	-4.65151600	-1.78571000
H	4.21595900	-4.87252900	1.14800300
H	3.44329200	-3.57765200	2.08609100
H	2.35640600	-6.41469900	1.62747200
H	2.37690300	-5.48641500	3.13219000
H	-0.48323800	-5.88604500	2.91256900
H	-2.45300000	-4.63887100	2.05172000
C	-2.54987000	-2.56197300	0.36450100
C	-3.29595500	-1.82446200	1.32605500

C	-3.02344400	-2.64275100	-0.96894200
C	-4.48294300	-1.20027900	0.92623900
C	-4.22720600	-2.00475900	-1.30885700
C	-4.98137600	-1.28419400	-0.37996700
H	-5.05172600	-0.62769500	1.66468500
H	-4.58335700	-2.09079000	-2.33680300
C	4.23982800	1.03500300	-0.34712400
C	4.52532800	1.45128400	0.98099400
C	4.10018400	2.01195100	-1.36935000
C	4.67906300	2.81792500	1.24994000
C	4.27613300	3.36454500	-1.04151900
C	4.57224200	3.79511800	0.25451900
H	4.90808300	3.12506300	2.27366200
H	4.17859000	4.11619900	-1.82952900
C	3.77661300	1.65484400	-2.82292500
H	3.46047700	0.60245700	-2.84270600
C	5.01004600	1.80567700	-3.73432200
H	5.85815000	1.19378800	-3.39331500
H	4.76934200	1.50180100	-4.76657200
H	5.35210900	2.85357000	-3.76594700
C	2.60772900	2.47649600	-3.39441000
H	2.86683400	3.54192600	-3.50798200
H	2.33768000	2.10055500	-4.39474800
H	1.71531200	2.40650900	-2.75992200
C	4.71239900	0.46672500	2.13718900
H	4.59998600	-0.55028000	1.73790200
C	3.63549800	0.64629200	3.22216900
H	2.62742200	0.53158500	2.79791300
H	3.75762800	-0.10835200	4.01712400
H	3.70093000	1.63902800	3.69775400
C	6.12954000	0.54815400	2.73361500
H	6.89917300	0.38746100	1.96209700
H	6.32357700	1.52831100	3.19920800
H	6.26528800	-0.21952000	3.51345400
C	4.79595700	5.27282000	0.55430700
H	4.57645000	5.82170800	-0.37821000
C	3.84304600	5.80884200	1.63547800
H	3.98703900	6.89220600	1.78035300
H	2.79013200	5.63882100	1.36164500
H	4.01828500	5.32009200	2.60800600
C	6.26414900	5.55826300	0.91799300
H	6.55137400	5.04385600	1.84992500
H	6.94622300	5.21493500	0.12452900
H	6.42960200	6.63814700	1.06769000

C	-2.87654100	-1.70319900	2.79426500
H	-1.88454800	-2.16260600	2.90058700
C	-3.84311300	-2.46687600	3.71964900
H	-3.49098200	-2.43168400	4.76401000
H	-4.85376400	-2.02696700	3.69463100
H	-3.93943300	-3.52522700	3.43132400
C	-2.73105600	-0.24342000	3.25495300
H	-3.68231300	0.30944100	3.19074200
H	-2.40015100	-0.20840800	4.30625200
H	-1.98097000	0.27681200	2.64567500
C	-2.30611000	-3.44264700	-2.05741000
H	-1.39165200	-3.86569300	-1.62088500
C	-3.15542600	-4.63490200	-2.53595900
H	-3.43131000	-5.29344000	-1.69706700
H	-4.08859200	-4.30411800	-3.02063700
H	-2.59673200	-5.23771000	-3.27118000
C	-1.87145100	-2.55456500	-3.23664900
H	-2.73940800	-2.12397400	-3.76329600
H	-1.23066600	-1.72885100	-2.89487700
H	-1.30131300	-3.14501700	-3.97317800
C	-6.32465700	-0.63951600	-0.71265300
H	-6.32894600	0.35016200	-0.22086500
C	-6.56129600	-0.40787900	-2.20941100
H	-7.48225500	0.17617600	-2.36545500
H	-5.72730200	0.13969800	-2.67533800
H	-6.68273300	-1.35713200	-2.75648200
C	-7.48538000	-1.45361000	-0.10725800
H	-7.52966600	-2.46055400	-0.55471500
H	-7.36838400	-1.57826800	0.98003900
H	-8.45269300	-0.95663900	-0.28973100

CP III-R-Post

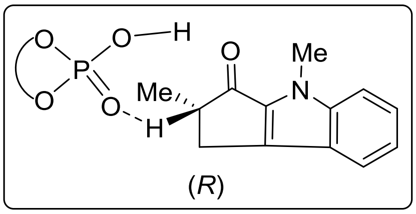

SCF energy[B3LYP-D3BJ/DEF2TZVP/SMD(1,2-dichloroethane)]: -3103.62470155 a.u.

Thermal correction to Gibbs free energy at 298 K: 1.08125400 a.u.

Gibbs free energy at 298 K [B3LYP-D3BJ/DEF2TZVP/SMD(1,2-dichloroethane)]: -3102.54344755 a.u.

C	7.67153300	4.53219900	1.09105700
C	8.29904700	3.65687600	0.17291700
C	7.57873700	2.70185400	-0.53615300
C	6.19368400	2.62875400	-0.31083900
C	5.53787700	3.51322300	0.61483300
C	6.30348900	4.46820000	1.31639000
N	5.26040200	1.78155900	-0.87413200
C	4.03678400	2.12060500	-0.33222100
C	4.15547000	3.15929000	0.57272700
C	5.50899000	0.77242400	-1.88544500
C	2.67121200	1.66261500	-0.44339000
C	1.82476700	2.57874700	0.44976200
C	2.83090100	3.52601900	1.17513100
C	0.76599800	3.31435600	-0.38339300
H	8.27739700	5.26681200	1.62657700
H	9.37822100	3.73387500	0.01735600
H	8.07593500	2.03351100	-1.24190100
H	5.82279800	5.14641900	2.02565900
H	4.57069700	0.24045800	-2.08498200
H	5.86963100	1.22985800	-2.82195600
H	6.26082800	0.04686200	-1.53601600
H	2.81889900	3.37064800	2.26734100
H	2.57934600	4.58733100	1.00920800
H	1.23815700	3.99963500	-1.10729600
H	0.14197700	2.60044300	-0.93875400
H	0.09743300	3.89662400	0.26635400
O	2.24265700	0.72878800	-1.12730100
H	0.78880100	0.06132400	-1.12019300
P	-0.95068100	-0.45994900	0.12395900
O	-0.91844500	-1.99505700	0.67956800
O	-2.44523900	-0.31128100	-0.51893200
O	-0.59772500	0.46242300	1.22838500
O	-0.10172800	-0.41023300	-1.20695000
C	-1.16046900	-3.11375300	-0.10212300
C	-0.06891000	-3.87642000	-0.57596900
C	-0.36280200	-5.00772900	-1.35808400
C	-1.66821800	-5.33560100	-1.73008100
C	-2.72090400	-4.56183900	-1.24967700
C	-4.19958700	-4.69115400	-1.53801200
C	-4.76330800	-3.36883200	-0.98388200
C	-3.80737600	-2.99876400	0.19974500
C	-2.47915300	-3.48895000	-0.37281000
C	-4.21302500	-3.81733100	1.47096400

C	-5.41078700	-3.04759800	2.06001100
C	-5.11548200	-1.62131700	1.65373200
C	-5.71068600	-0.44539700	2.10243500
C	-5.25110900	0.77709700	1.60831100
C	-4.15235600	0.86516700	0.73427400
C	-3.56153100	-0.34546100	0.31000500
C	-4.08238800	-1.58230600	0.70035200
H	1.29195900	1.92500600	1.16045200
H	0.46593100	-5.62718700	-1.70416800
H	-1.85458400	-6.18886800	-2.38772600
H	-4.63332300	-5.56555500	-1.02038900
H	-4.40543400	-4.82919800	-2.61162300
H	-5.81662300	-3.43343000	-0.67399400
H	-4.69015300	-2.58068100	-1.75040200
H	-4.43392100	-4.87057500	1.24369600
H	-3.37061100	-3.79905900	2.18090100
H	-6.36721600	-3.39610700	1.63058700
H	-5.49866300	-3.16103700	3.15248300
H	-6.52849300	-0.47308200	2.82751600
H	-5.73260700	1.70331700	1.92792000
C	-3.67811700	2.21654400	0.28377400
C	-3.16519300	3.14437300	1.22827400
C	-3.82187500	2.60933100	-1.07438100
C	-2.83041800	4.43621400	0.79476800
C	-3.46206200	3.91039300	-1.44971100
C	-2.97170900	4.84717900	-0.53324800
H	-2.44398600	5.15456200	1.52378500
H	-3.58599300	4.20488100	-2.49510800
C	1.36030400	-3.53083700	-0.27229500
C	1.84211800	-3.57384400	1.06464300
C	2.26090600	-3.22050700	-1.32767500
C	3.19350500	-3.30214100	1.31219700
C	3.60470100	-2.96416600	-1.01719100
C	4.09750600	-2.99671200	0.28939800
H	3.55243900	-3.34437500	2.34370800
H	4.29761000	-2.72996300	-1.83012400
C	1.84308900	-3.17501000	-2.80123900
H	0.74616400	-3.22462200	-2.84040700
C	2.39690400	-4.38404900	-3.58052100
H	2.09781000	-5.34269400	-3.13015600
H	2.03660600	-4.37316600	-4.62268800
H	3.49912400	-4.36625100	-3.60893200
C	2.24754400	-1.86872200	-3.50690500
H	3.34224200	-1.77373900	-3.60139900

H	1.83302000	-1.84946300	-4.52822400
H	1.87692100	-0.98681700	-2.97000600
C	0.96264700	-3.96391000	2.25358700
H	-0.06016400	-4.11470400	1.88501100
C	0.89841700	-2.85696300	3.32080300
H	0.55257700	-1.90732200	2.88785700
H	0.19830100	-3.13996600	4.12446700
H	1.88161300	-2.68584100	3.78999500
C	1.41106000	-5.30559700	2.86320500
H	1.41810500	-6.10748200	2.10786500
H	2.42541800	-5.24101100	3.29030200
H	0.72889300	-5.60956000	3.67463100
C	5.56818200	-2.72372600	0.58013200
H	6.04457900	-2.49132800	-0.38912100
C	5.76324400	-1.50123600	1.49341300
H	6.83477800	-1.28048000	1.63134900
H	5.28085600	-0.60478100	1.07373300
H	5.33049000	-1.67273900	2.49245300
C	6.28049900	-3.96269200	1.15078600
H	5.86371200	-4.24758700	2.13080600
H	6.17520200	-4.82922100	0.47938500
H	7.35655400	-3.76715800	1.29161900
C	-2.96436900	2.80734800	2.70843200
H	-3.18187700	1.73891100	2.84112600
C	-3.93507500	3.59698500	3.60640800
H	-3.81825800	3.29722100	4.66107500
H	-3.74611400	4.68177500	3.54722100
H	-4.98641900	3.43160500	3.32281400
C	-1.50872300	3.01137900	3.16304500
H	-1.19389400	4.06488900	3.07900700
H	-1.39888200	2.72080200	4.22123300
H	-0.83486400	2.38278300	2.56583200
C	-4.40429600	1.68765700	-2.14753400
H	-4.63860400	0.72391100	-1.67612400
C	-5.73072800	2.23634800	-2.70544300
H	-6.46321000	2.41031600	-1.90130400
H	-5.58690800	3.19083100	-3.23801300
H	-6.17405600	1.52315500	-3.42017900
C	-3.39684900	1.40382700	-3.27576200
H	-3.13056000	2.32301200	-3.82358200
H	-2.47303300	0.95960100	-2.87776700
H	-3.82598900	0.69835300	-4.00688700
C	-2.62845000	6.27261900	-0.95061300
H	-2.19394100	6.76607100	-0.06341200

C	-1.57095600	6.32428900	-2.06594800
H	-1.29442500	7.36716800	-2.29322900
H	-0.65650300	5.78376400	-1.77664800
H	-1.94429400	5.87201300	-2.99929600
C	-3.88743500	7.06641000	-1.34300500
H	-4.37146600	6.63206300	-2.23328000
H	-4.62854900	7.06809300	-0.52852200
H	-3.63474600	8.11379600	-1.57790700

CP III-S-Post

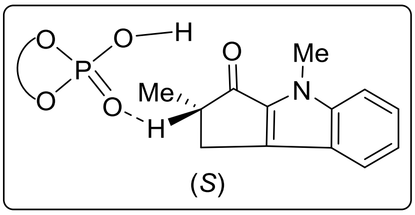

SCF energy[B3LYP-D3BJ/DEF2TZVP/SMD(1,2-dichloroethane)]: -3103.62568349 a.u.
Thermal correction to Gibbs free energy at 298 K: 1.08018100 a.u.

Gibbs free energy at 298 K [B3LYP-D3BJ/DEF2TZVP/SMD(1,2-dichloroethane)]: -3102.54550249 a.u.

C	8.03723200	3.69866900	-0.60048200
C	8.20809700	3.48876500	0.78892900
C	7.16593000	3.04459900	1.59505700
C	5.92332400	2.80694800	0.98338100
C	5.73034900	3.01607600	-0.42722900
C	6.81302600	3.46698600	-1.21167400
N	4.74238200	2.36862300	1.54709900
C	3.81572500	2.30215000	0.52678600
C	4.36615600	2.68314300	-0.68320900
C	4.51538100	2.03959800	2.94136900
C	2.43108400	1.92180600	0.39024000
C	2.06243600	2.11386200	-1.08751000
C	3.36311600	2.60715800	-1.79631900
C	0.85913600	3.05048700	-1.25562600
H	8.88450500	4.04844800	-1.19487100
H	9.18527500	3.68098200	1.23939100
H	7.31191600	2.88637100	2.66538300
H	6.68540900	3.63091100	-2.28448900
H	3.47265300	1.71839600	3.05550700
H	5.17834100	1.22065500	3.26477700
H	4.69436200	2.91466400	3.58767900

H	3.21014800	3.58728100	-2.27986800
H	3.67975000	1.91310100	-2.59299300
H	-0.00871500	2.68380700	-0.69063000
H	1.09508600	4.06902000	-0.90469300
H	0.56469600	3.10839700	-2.31420400
O	1.67655700	1.51580900	1.28186200
H	0.24255100	0.89621400	1.09901800
P	-0.93909800	-0.48545600	-0.15685800
O	-2.52630200	-0.35500300	-0.49340200
O	-0.81698600	-1.93584500	0.59405200
O	-0.13655900	-0.32880600	-1.39153300
O	-0.68836200	0.49584900	1.05230200
C	-3.51308700	-0.39940500	0.48158200
C	-4.08049000	0.81024500	0.94084500
C	-5.05936900	0.72167800	1.94613800
C	-5.41826500	-0.49719500	2.52706300
C	-4.84773100	-1.67140900	2.04303300
C	-5.04985200	-3.09114400	2.52327100
C	-3.90864900	-3.84837100	1.81674500
C	-3.68056600	-3.06311200	0.48206700
C	-3.94132600	-1.63737400	0.96790700
C	-4.74932100	-3.50543200	-0.57300000
C	-4.20185700	-4.82579600	-1.15066600
C	-2.70546300	-4.63593800	-1.03974500
C	-1.68211400	-5.37518900	-1.62820800
C	-0.35623600	-4.98841800	-1.41633200
C	-0.02269800	-3.83219200	-0.68794400
C	-1.08437800	-3.10261700	-0.11007000
C	-2.40881900	-3.53191000	-0.22174900
H	1.75922200	1.11950500	-1.45791000
H	-5.53058100	1.64259800	2.29333600
H	-6.14085600	-0.52311100	3.34715200
H	-6.03999200	-3.47798200	2.22204300
H	-5.00339700	-3.17219400	3.62115800
H	-4.12389200	-4.91381600	1.64858500
H	-2.98879800	-3.78565000	2.41998000
H	-5.75721100	-3.59645200	-0.14192500
H	-4.79377300	-2.74160400	-1.36582400
H	-4.53930300	-5.69660300	-0.56009700
H	-4.52565500	-5.00684700	-2.18812800
H	-1.90700900	-6.24698700	-2.24850100
H	0.45299100	-5.57845400	-1.85111500
C	1.41459900	-3.42383600	-0.54127100
C	2.17173300	-3.04341500	-1.68071600

C	2.04315500	-3.46947400	0.73203100
C	3.52926700	-2.72977000	-1.51623800
C	3.40102800	-3.14146900	0.83488000
C	4.16889200	-2.77228100	-0.27485500
H	4.11545700	-2.44191900	-2.39355900
H	3.87740900	-3.19291200	1.81761200
C	-3.69763500	2.15093800	0.38063900
C	-4.00760100	2.47414300	-0.96836500
C	-3.08192700	3.12959000	1.20672500
C	-3.70080300	3.75227900	-1.45275900
C	-2.80218000	4.39357600	0.66626600
C	-3.10185700	4.73322700	-0.65547900
H	-3.95122600	3.98961100	-2.48994700
H	-2.33139400	5.14737700	1.30312400
C	-2.72168300	2.87886700	2.67384900
H	-2.81857600	1.80061900	2.86222500
C	-3.68639100	3.61501600	3.62434300
H	-4.73729600	3.34480500	3.44160400
H	-3.45447300	3.37664300	4.67577600
H	-3.60368100	4.70802400	3.50399200
C	-1.26877000	3.25809800	3.01121900
H	-1.09754600	4.34467500	2.93762700
H	-1.03395900	2.96360300	4.04741100
H	-0.55296200	2.75391000	2.34896600
C	-4.70935800	1.50032600	-1.91695300
H	-4.86730800	0.55591700	-1.37942800
C	-3.85248900	1.17743800	-3.15425700
H	-2.86746800	0.78564900	-2.86252800
H	-4.35117900	0.41760800	-3.77901500
H	-3.69694200	2.06856400	-3.78482500
C	-6.10461700	2.01339800	-2.31927100
H	-6.73233400	2.20532200	-1.43457700
H	-6.04442100	2.95153400	-2.89505300
H	-6.62320000	1.27139200	-2.94903600
C	-2.79986800	6.12693600	-1.19319500
H	-2.30176700	6.68032000	-0.37769700
C	-1.82819100	6.09586800	-2.38514600
H	-1.57869300	7.11863700	-2.71318700
H	-0.88992700	5.58254600	-2.12362800
H	-2.26640200	5.56909000	-3.24878200
C	-4.08622900	6.89431700	-1.54634900
H	-4.63157900	6.40422000	-2.36969000
H	-4.76742800	6.95151900	-0.68297000
H	-3.85381100	7.92336200	-1.86731600

C	1.58427500	-2.97426600	-3.09279300
H	0.49975200	-3.12883800	-3.01299000
C	2.14989600	-4.09186900	-3.98969300
H	1.67245000	-4.07145700	-4.98344700
H	3.23595200	-3.97419500	-4.14023100
H	1.98571700	-5.09112200	-3.55649400
C	1.77801200	-1.59630200	-3.74967500
H	2.84405300	-1.35250900	-3.89300100
H	1.30484200	-1.58428400	-4.74579300
H	1.30636200	-0.81566300	-3.13805500
C	1.31421500	-3.91469300	2.00118500
H	0.26800000	-4.12002400	1.73779300
C	1.89425300	-5.23118800	2.55075200
H	1.86503100	-6.02911500	1.79192900
H	2.94288600	-5.11346300	2.86985000
H	1.31753500	-5.57334000	3.42621000
C	1.29873600	-2.81597000	3.07850800
H	2.31487900	-2.58329800	3.43814200
H	0.85100400	-1.88970100	2.69063500
H	0.70834000	-3.14082800	3.95154700
C	5.65490100	-2.45716900	-0.15008000
H	5.99283300	-2.12012400	-1.14564200
C	5.93466400	-1.31244900	0.83767900
H	7.00778400	-1.06020100	0.85005900
H	5.37585700	-0.40357100	0.56652300
H	5.64805000	-1.58969400	1.86586700
C	6.47109300	-3.71156400	0.20991500
H	6.18563000	-4.10171500	1.20092400
H	6.30970000	-4.51698500	-0.52349600
H	7.54998900	-3.48501500	0.23747900

Product-R

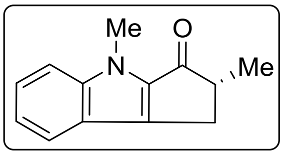

SCF energy[B3LYP-D3BJ/DEF2TZVP/SMD(1,2-dichloroethane)]: -633.508416633 a.u.
Thermal correction to Gibbs free energy at 298 K: 0.19188300 a.u.

Gibbs free energy at 298 K [B3LYP-D3BJ/DEF2TZVP/SMD(1,2-dichloroethane)]: -633.31653363 a.u.

C	-3.46354700	-1.41656000	-0.05091000

C	-3.81262900	-0.04791700	-0.12264900
C	-2.84586200	0.95258900	-0.11058400
C	-1.50006400	0.56043600	-0.02384000
C	-1.12556800	-0.82669700	0.05238900
C	-2.13453300	-1.81150800	0.03559400
N	-0.36028300	1.34021500	0.00595600
C	0.71149000	0.47940100	0.10384100
C	0.30239900	-0.83664300	0.13200900
C	-0.29090300	2.78729400	-0.04727800
C	2.16187100	0.61290900	0.16962500
C	2.70030500	-0.83237100	0.33801500
C	1.46798200	-1.78260600	0.20840300
O	2.84338000	1.62045000	0.11124100
C	3.86130900	-1.12143100	-0.61359300
H	-4.25457300	-2.17019500	-0.06376200
H	-4.86757200	0.23033000	-0.18947700
H	-3.12777900	2.00607100	-0.16682400
H	-1.87183800	-2.87095300	0.09110400
H	0.76664800	3.08020100	-0.02687700
H	-0.75120700	3.17058300	-0.97295500
H	-0.80752200	3.24231900	0.81428800
H	3.08261800	-0.87709600	1.37442600
H	1.39317500	-2.48180000	1.05840500
H	1.54404200	-2.40644000	-0.69985800
H	4.63769000	-0.34886300	-0.50664500
H	4.31472600	-2.10360400	-0.40634800
H	3.52303400	-1.11702500	-1.66322200

Product-S

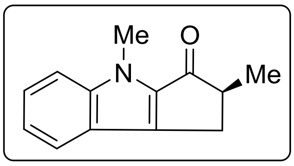

SCF energy[B3LYP-D3BJ/DEF2TZVP/SMD(1,2-dichloroethane)]: -633.508415944 a.u.
Thermal correction to Gibbs free energy at 298 K: 0.19188400 a.u.

Gibbs free energy at 298 K [B3LYP-D3BJ/DEF2TZVP/SMD(1,2-dichloroethane)]: -633.31653194 a.u.

C	-3.46357600	-1.41666100	0.05066400
C	-3.81271000	-0.04803700	0.12236200
C	-2.84596500	0.95249900	0.11055100

C	-1.50013700	0.56038900	0.02409200
C	-1.12559000	-0.82672200	-0.05216900
C	-2.13452500	-1.81156300	-0.03556500
N	-0.36035800	1.34018700	-0.00540000
C	0.71144500	0.47942300	-0.10330600
C	0.30238000	-0.83662400	-0.13163200
C	-0.29107100	2.78730700	0.04658900
C	2.16181000	0.61300300	-0.16919600
C	1.46803400	-1.78252200	-0.20790700
O	2.84328400	1.62057800	-0.11061300
C	3.86193900	-1.12152000	0.61239600
H	-4.25457600	-2.17032800	0.06332000
H	-4.86767800	0.23018800	0.18893100
H	-3.12793500	2.00597400	0.16669100
H	-1.87178900	-2.87100100	-0.09106200
H	0.76652600	3.08017500	0.02801500
H	-0.80591300	3.24160000	-0.81645300
H	-0.75327600	3.17139900	0.97097000
H	1.54439900	-2.40585200	0.70067000
H	1.39312900	-2.48212500	-1.05755600
H	3.52451500	-1.11735000	1.66231100
H	4.31511900	-2.10369300	0.40459200
H	4.63837300	-0.34903800	0.50511700
C	2.70024200	-0.83217500	-0.33819500
H	3.08167500	-0.87666300	-1.37494400

TS IV

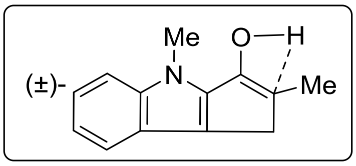

SCF energy[B3LYP-D3BJ/DEF2TZVP/SMD(1,2-dichloroethane)]: -633.38331274 a.u.
Thermal correction to Gibbs free energy at 298 K: 0.18556100 a.u.

Gibbs free energy at 298 K [B3LYP-D3BJ/DEF2TZVP/SMD(1,2-dichloroethane)]: -633.19775174 a.u.

C	3.46650400	-1.41930100	-0.06999200
C	3.82284000	-0.05599700	0.04919000
C	2.85903600	0.94486500	0.12662100
C	1.51019100	0.55913500	0.07497100
C	1.12716200	-0.82408100	-0.03222100

C	2.13400200	-1.80889000	-0.11072300
N	0.37352700	1.34613800	0.10861500
C	-0.70536300	0.48946800	0.07270100
C	-0.30216800	-0.83742900	-0.01701200
C	0.31436600	2.79092200	0.18780800
C	-2.12047100	0.54539600	-0.15286800
C	-2.67879900	-0.77582600	-0.20990500
C	-1.48420400	-1.75276800	-0.24436900
C	-3.85625200	-1.14211800	0.68676700
H	4.25488000	-2.17355600	-0.12810500
H	4.88042600	0.21821100	0.08076900
H	3.14691500	1.99424000	0.21865400
H	1.86522900	-2.86451100	-0.19817600
H	-0.72624800	3.10609000	0.03252500
H	0.65658900	3.16012600	1.16975900
H	0.93971300	3.24809800	-0.59561600
H	-1.39326400	-2.27459000	-1.21482900
H	-1.56595100	-2.54172300	0.52430300
H	-3.52511500	-1.44479100	1.70011100
H	-4.55730000	-0.30027100	0.79887300
H	-4.42677300	-1.98538500	0.26427600
O	-2.91194900	1.47791100	-0.57145900
H	-3.38925300	0.33156000	-1.00698200

H_2O

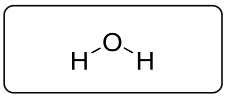

SCF energy[B3LYP-D3BJ/DEF2TZVP/SMD(1,2-dichloroethane)]: -76.47109086 a.u.
Thermal correction to Gibbs free energy at 298 K: 0.00357200 a.u.

Gibbs free energy at 298 K [B3LYP-D3BJ/DEF2TZVP/SMD(1,2-dichloroethane)]: -76.46751886 a.u.

O	0.00000000	0.00000000	0.12020700
H	0.00000000	0.75703900	-0.48082800
H	0.00000000	-0.75703900	-0.48082800

CP V-Pre

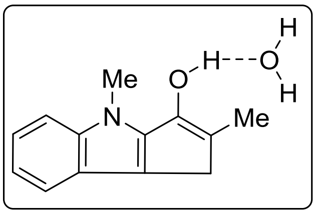

SCF energy[B3LYP-D3BJ/DEF2TZVP/SMD(1,2-dichloroethane)]: -709.96157397 a.u.

Thermal correction to Gibbs free energy at 298 K: 0.20880300 a.u.

Gibbs free energy at 298 K [B3LYP-D3BJ/DEF2TZVP/SMD(1,2-dichloroethane)]: -709.75277097 a.u.

C	-4.19763600	-0.94487200	0.00040300
C	-4.31666400	0.46040800	0.00048800
C	-3.18576000	1.27832700	0.00035000
C	-1.92884800	0.66233000	0.00016100
C	-1.78448100	-0.77063200	-0.00002700
C	-2.94942500	-1.56316100	0.00015400
N	-0.66619600	1.24387700	0.00010700
C	0.24748900	0.21364700	-0.00039300
C	-0.37541100	-1.01416000	-0.00043700
C	-0.38626900	2.66264800	-0.00097100
C	1.69869700	0.05735500	-0.00016200
C	1.98454300	-1.28046600	-0.00007700
C	0.68395900	-2.08085100	-0.00018800
C	3.32999000	-1.93662700	-0.00042700
H	-5.10313500	-1.55752000	0.00053400
H	-5.30968000	0.91720800	0.00065000
H	-3.28372100	2.36656800	0.00039200
H	-2.87319000	-2.65372100	0.00006500
H	0.70102400	2.80597600	-0.00038300
H	-0.80952800	3.15209700	-0.89507800
H	-0.81066200	3.15355300	0.89177800
H	0.62521700	-2.74381400	0.88390400
H	0.62557300	-2.74391900	-0.88423200
H	3.46794300	-2.58588100	-0.88533900
H	4.15102500	-1.20471100	0.00017300
H	3.46776500	-2.58729200	0.88345100
O	2.50427400	1.14259500	-0.00011500
H	3.45436500	0.90543300	0.00049000
O	5.27548100	0.94776300	0.00086800
H	5.60079100	1.43963400	0.76842200

H	5.60043600	1.44271000	-0.76485800

TS V

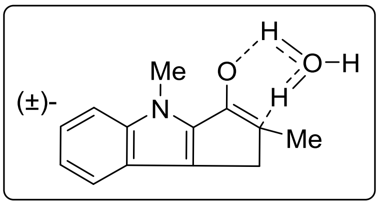

SCF energy[B3LYP-D3BJ/DEF2TZVP/SMD(1,2-dichloroethane)]: -709.91535875 a.u.
Thermal correction to Gibbs free energy at 298 K: 0.20881400 a.u.

Gibbs free energy at 298 K [B3LYP-D3BJ/DEF2TZVP/SMD(1,2-dichloroethane)]: -709.70654475 a.u.

C	-3.88035300	-1.30984600	0.28243800
C	-4.18207000	0.06729900	0.18177300
C	-3.18129300	1.02021300	0.01154900
C	-1.85341300	0.57084700	-0.05253300
C	-1.52640400	-0.82808100	0.03897700
C	-2.56813400	-1.76229400	0.21182200
N	-0.68811100	1.30222200	-0.20244000
C	0.34926800	0.39503100	-0.23315700
C	-0.10492500	-0.90318500	-0.09801100
C	-0.57855600	2.74072300	-0.33201800
C	1.79504600	0.42856200	-0.25364800
C	2.28264200	-0.91991800	-0.12962600
C	1.05928900	-1.85221400	-0.03841100
C	3.47511200	-1.38815000	-0.95931200
H	-4.69484000	-2.02613500	0.41527200
H	-5.22429700	0.39169400	0.23899700
H	-3.42561300	2.08186100	-0.06550900
H	-2.34372600	-2.82936600	0.28589200
H	0.48607000	3.00692100	-0.34822200
H	-1.05224000	3.09828300	-1.26216700
H	-1.05944900	3.24865700	0.52021800
H	1.05212100	-2.45126600	0.89099200
H	1.04911300	-2.58059400	-0.86947800
H	3.16506700	-1.67179000	-1.98246600
H	4.23824300	-0.60141500	-1.04869900
H	3.96439100	-2.26694400	-0.50795600
O	2.53289000	1.48323400	-0.17736000
H	3.41846900	1.09191600	0.56745500
O	3.95607800	0.34699200	1.37238100

H	3.12288700	-0.45200000	0.98964400
H	3.69158300	0.64890400	2.25387700

CP V-Post

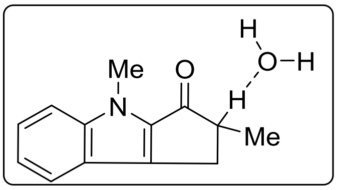

SCF energy[B3LYP-D3BJ/DEF2TZVP/SMD(1,2-dichloroethane)]: -709.98779584 a.u.
Thermal correction to Gibbs free energy at 298 K: 0.21257600 a.u.

Gibbs free energy at 298 K [B3LYP-D3BJ/DEF2TZVP/SMD(1,2-dichloroethane)]: -709.77521984 a.u.

C	-4.10754800	-1.05333400	-0.03423800
C	-4.28184200	0.34830300	-0.11692100
C	-3.19828000	1.22026500	-0.11326000
C	-1.91173600	0.66303700	-0.02440600
C	-1.71482500	-0.75972700	0.06129300
C	-2.83919800	-1.61127300	0.05398900
N	-0.68421900	1.29495000	-0.00229800
C	0.27179900	0.30644900	0.09714600
C	-0.30057900	-0.94902200	0.13604600
C	-0.43869800	2.72278300	-0.06577300
C	1.72022800	0.25310200	0.16300800
C	2.08253200	-1.24372400	0.31593700
C	0.73687300	-2.03132900	0.21882300
C	3.17290100	-1.68401800	-0.66509300
H	-4.98717900	-1.70132500	-0.04071500
H	-5.29377100	0.75576100	-0.18537300
H	-3.34655900	2.30013500	-0.17734900
H	-2.71065400	-2.69470000	0.11691100
H	0.64605100	2.88652300	-0.04198800
H	-0.84496900	3.15210100	-0.99637600
H	-0.89974500	3.24278900	0.79032300
H	0.58455900	-2.69842300	1.08408300
H	0.72306000	-2.67673600	-0.67681400
H	2.81414300	-1.61775600	-1.70578100
H	4.06052500	-1.04118200	-0.56600000
H	3.46967600	-2.72775300	-0.47544000
O	2.51330500	1.18611000	0.11849300
H	4.41440700	1.08949200	-0.03616400

O	5.36196000	0.85482500	-0.04865800
H	2.48315000	-1.33895000	1.34252500
H	5.52494800	0.58881300	0.86626200

CP VI-Pre

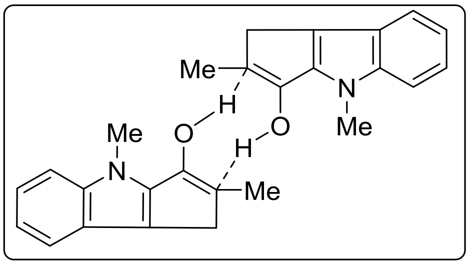

SCF energy[B3LYP-D3BJ/DEF2TZVP/SMD(1,2-dichloroethane)]: -1266.97145575 a.u.
Thermal correction to Gibbs free energy at 298 K: 0.39883200 a.u.

Gibbs free energy at 298 K [B3LYP-D3BJ/DEF2TZVP/SMD(1,2-dichloroethane)]: -1266.57262375 a.u.

C	5.49509500	-1.19606800	0.87496800
C	4.94931300	-2.25917000	0.12377300
C	3.76357400	-2.10273900	-0.59251100
C	3.12651300	-0.85561900	-0.54273700
C	3.66853400	0.24274400	0.21391700
C	4.86937700	0.04592200	0.92534100
N	1.94657600	-0.45001800	-1.15533800
C	1.75467300	0.86628800	-0.80647000
C	2.76026900	1.32922000	0.01498500
C	1.10802500	-1.26719200	-2.01151800
C	0.75856200	1.90714800	-1.01666400
C	1.13339600	3.02691100	-0.31984100
C	2.46104800	2.75515100	0.38761500
C	0.47670500	4.37397700	-0.34257600
H	6.42660100	-1.35385100	1.42452400
H	5.46508400	-3.22244800	0.10242100
H	3.34676700	-2.92895100	-1.17267400
H	5.30466600	0.86141400	1.50869000
H	0.22946500	-0.68322300	-2.30987800
H	1.65386100	-1.58012300	-2.91751300
H	0.76211500	-2.16815500	-1.47988800
H	2.38319300	2.90937800	1.48030000
H	3.23707700	3.46100500	0.03664500
H	1.13019800	5.13297900	-0.81027700
H	-0.46722400	4.36928300	-0.91217800

H	0.23360700	4.74101700	0.66943100
O	-0.31585300	1.68929100	-1.80951200
H	-0.95711600	2.40851800	-1.71761500
C	-3.10589600	-4.19866800	-0.52447200
C	-1.91674500	-4.40901500	0.20445400
C	-1.23524900	-3.34324500	0.79423400
C	-1.76762700	-2.05709900	0.63925000
C	-2.98073200	-1.82078300	-0.09875400
C	-3.64057100	-2.92216300	-0.67941300
N	-1.28783400	-0.84497200	1.12404200
C	-2.16409600	0.12911200	0.69851900
C	-3.20115000	-0.40972600	-0.03182300
C	-0.15067700	-0.68440700	2.00784000
C	-2.31930000	1.57971400	0.80135400
C	-3.47134100	1.93850900	0.16383200
C	-4.13940800	0.69904600	-0.42161700
C	-4.03763300	3.31825900	0.06258300
H	-3.61523100	-5.05512100	-0.97387800
H	-1.52206400	-5.42266300	0.31055600
H	-0.31468900	-3.51230800	1.35764000
H	-4.56380000	-2.77519100	-1.24595600
H	-0.15025200	0.33740300	2.40712000
H	-0.22252600	-1.38167300	2.85836200
H	0.80466900	-0.86463400	1.48802400
H	-4.26466200	0.80274200	-1.51587900
H	-5.15635900	0.55960300	-0.00945500
H	-4.99887400	3.40648700	0.60158700
H	-3.34623600	4.05597000	0.49593300
H	-4.24358100	3.60552500	-0.98455600
O	-1.47893600	2.42582500	1.46784100
H	-0.57552100	2.32438100	1.11534500

TS VI

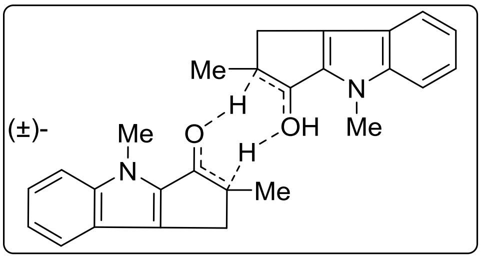

SCF energy[B3LYP-D3BJ/DEF2TZVP/SMD(1,2-dichloroethane)]: -1266.93973705 a.u.

Thermal correction to Gibbs free energy at 298 K: 0.39742600 a.u.

Gibbs free energy at 298 K [B3LYP-D3BJ/DEF2TZVP/SMD(1,2-dichloroethane)]:
-1266.54231105 a.u.

C	-5.73785400	-1.84820600	-0.93288100
C	-5.32115600	-2.73773900	0.08648500
C	-4.22637700	-2.45972100	0.89759900
C	-3.53474300	-1.25834600	0.67104400
C	-3.94760600	-0.33805200	-0.35638300
C	-5.06412200	-0.65700000	-1.15952600
N	-2.42110000	-0.76002300	1.31948100
C	-2.14052200	0.45976800	0.73800300
C	-3.04210900	0.75868500	-0.27751200
C	-1.69126200	-1.40899800	2.39268200
C	-1.11159100	1.44283100	0.77952700
C	-1.34224000	2.44559200	-0.23738700
C	-2.65794700	2.04152200	-0.94777500
C	-1.21181300	3.91809400	0.16088500
H	-6.60341900	-2.10860800	-1.54635800
H	-5.87409800	-3.66805100	0.23899400
H	-3.91602800	-3.15622500	1.67879000
H	-5.39041700	0.02779300	-1.94590800
H	-0.81549100	-0.79903100	2.64427800
H	-2.32087600	-1.51972000	3.29106000
H	-1.34509600	-2.40690800	2.07940900
H	-2.51957400	1.91242800	-2.03573600
H	-3.42913600	2.82211800	-0.82481400
H	-2.09597000	4.25844000	0.72878200
H	-0.32354400	4.09743400	0.77980000
H	-1.11434700	4.55174400	-0.73374700
O	-0.08015000	1.35953000	1.58074000
H	0.70511700	1.90416600	1.22371600
C	4.79720800	-3.15301400	0.77451900
C	4.11831400	-3.75354000	-0.30721500
C	3.22461000	-3.02465600	-1.09192900
C	3.01701800	-1.67637600	-0.77251800
C	3.70396900	-1.04231600	0.32444000
C	4.59949300	-1.81204700	1.09339100
N	2.18767700	-0.74651800	-1.38179300
C	2.34672600	0.44057100	-0.70181400
C	3.25566000	0.31670300	0.32384000
C	1.34904700	-0.96551900	-2.54084800
C	1.76840700	1.79225200	-0.75980600
C	2.32560500	2.50550800	0.31237900
C	3.35778400	1.63427100	1.04094100

C	2.34416600	4.00598500	0.41695300
H	5.49184500	-3.75299300	1.36838900
H	4.29626900	-4.80771100	-0.53535900
H	2.70270300	-3.49495600	-1.92875900
H	5.13587500	-1.35839800	1.93112100
H	0.85224100	-0.01730500	-2.78377000
H	1.94713500	-1.29054400	-3.40926500
H	0.58408800	-1.73622300	-2.34233000
H	3.16030300	1.54614900	2.12721000
H	4.36822000	2.07915000	0.95771400
H	3.36170300	4.40226400	0.23494900
H	1.68382700	4.45098400	-0.34160500
H	2.03824100	4.39230900	1.40714800
O	0.86542200	2.16268100	-1.62817800
H	-0.29976200	2.28229100	-1.01058300

CP VI-Post

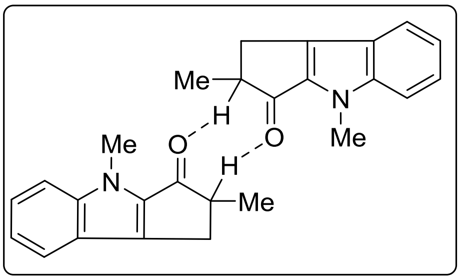

SCF energy[B3LYP-D3BJ/DEF2TZVP/SMD(1,2-dichloroethane)]: -1267.02183702 a.u.
Thermal correction to Gibbs free energy at 298 K: 0.39992700 a.u.

Gibbs free energy at 298 K [B3LYP-D3BJ/DEF2TZVP/SMD(1,2-dichloroethane)]: -1266.62191002 a.u.

C	6.59006900	-2.00430100	1.07410000
C	6.31551000	-2.78287400	-0.07417700
C	5.26960600	-2.46727800	-0.93582900
C	4.48666700	-1.34225200	-0.62939500
C	4.75022500	-0.53831800	0.53436600
C	5.81964100	-0.89033700	1.38311800
N	3.40491200	-0.81693800	-1.30840900
C	2.98656400	0.28985800	-0.59995200
C	3.76672900	0.50006600	0.51704800
C	2.81112200	-1.32659900	-2.52824800
C	1.93439700	1.28786600	-0.73514800
C	2.07380800	2.21277800	0.49586300
C	3.32563600	1.71391900	1.28485500

C	2.11127200	3.68957700	0.09548700
H	7.42122600	-2.28746500	1.72444500
H	6.94024400	-3.65359600	-0.28965600
H	5.06775600	-3.07631600	-1.81950800
H	6.03688800	-0.29293100	2.27217100
H	1.97837300	-0.66759300	-2.80534500
H	3.54633000	-1.34109100	-3.35000200
H	2.42698900	-2.35053300	-2.38545500
H	3.08171300	1.48073400	2.33510800
H	4.11476200	2.48604100	1.31310100
H	3.01947800	3.92415300	-0.48514300
H	1.23907500	3.93999300	-0.52686400
H	2.09827900	4.34072400	0.98384900
O	1.09632900	1.39337100	-1.61843400
H	-1.16117000	2.03855900	-1.09181900
C	-6.59128600	-2.00334200	-1.07355800
C	-6.31557700	-2.78316700	0.07359800
C	-5.26889800	-2.46841400	0.93461300
C	-4.48631900	-1.34301400	0.62864800
C	-4.75106200	-0.53777700	-0.53395000
C	-5.82127000	-0.88895900	-1.38206500
N	-3.40394700	-0.81837300	1.30721500
C	-2.98641000	0.28925500	0.59962600
C	-3.76771800	0.50073200	-0.51634400
C	-2.80875000	-1.32962600	2.52572400
C	-1.93384700	1.28683300	0.73469900
C	-2.07445100	2.21320200	-0.49499400
C	-3.32723800	1.71536700	-1.28328100
C	-2.11147500	3.68955200	-0.09270500
H	-7.42304700	-2.28585200	-1.72341500
H	-6.94004000	-3.65417300	0.28870400
H	-5.06618500	-3.07836000	1.81746800
H	-6.03947400	-0.29054600	-2.27020600
H	-1.97440000	-0.67213900	2.80159900
H	-3.54240200	-1.34326600	3.34889800
H	-2.42670100	-2.35413300	2.38161000
H	-3.08454300	1.48337100	-2.33406600
H	-4.11614300	2.48777700	-1.30966800
H	-3.01936100	3.92333400	0.48871900
H	-1.23889600	3.93895400	0.52952500
H	-2.09883800	4.34186100	-0.98020400
O	-1.09484100	1.39111300	1.61725400
H	1.15974800	2.03730200	1.09135500

References

[1] a) A. D. Becke, *J. Chem. Phys.*, 1993, **98**, 1372; b) A. D. Becke, *J. Chem. Phys.*, 1993, **98**, 5648.

[2] F. Weigend and R. Ahlrichs, *Phys.Chem. Chem. Phys.*, 2005, **7**, 3297.

[3] S. Grimme, J. Antony, S. Ehrlich and H. Krieg, *J. Chem. Phys.*, 2010, **132**, 154104.

[4] A. V. Marenich, C. J. Cramer and D. G. Truhlar, *J. Phys. Chem. B*, 2009, **113**, 6378.

[5] *CYLview, 1.0b*; C. Y. Legault, Université de Sherbrooke, 2009. (http://www.cylview.org).
